# FPW-YOLO11n: A Lightweight Frequency-Perception Framework for Lunar Impact Crater Detection

**DOI:** 10.3390/s26144344

**Published:** 2026-07-09

**Authors:** Jiarui Liang, Pengcheng Yan, Qi Wen, Qingjie Liu, Yikui Zhai, Xiaolin Tian

**Affiliations:** 1 Faculty of Innovation Engineering, Macau University of Science and Technology, Avenida Wai Long, N°S 100–460, Taipa, Macau, China; lianggary@163.com (J.L.); 3220004952@student.must.edu.mo (P.Y.); 2 Technology and Engineering Center for Space Utilization, Chinese Academy of Sciences, Beijing 100094, China; wenqi@csu.ac.cn; 3 The State Key Laboratory of Virtual Reality Technology and Systems, Beihang University, Beijing 100191, China; qingjie.liu@buaa.edu.cn; 4 The Department of Intelligent Manufacturing, Wuyi University, Jiangmen 529020, China; yikuizhai@163.com

**Keywords:** lunar impact crater detection, frequency-directional attention, local region self-attention, Inner-WIoU, digital elevation model

## Abstract

Automated detection of lunar impact craters from digital elevation model (DEM) data is important for lunar geological analysis, landing-site selection, and crater catalog updating. However, this task remains challenging because lunar craters exhibit large scale variations, weak or degraded rims, ambiguous boundaries, and complex topographic backgrounds. In addition, large-scale lunar remote sensing applications require detection models to achieve a reasonable balance among accuracy, model complexity, and inference efficiency. To address these challenges, this study proposes FPW-YOLO11n, a frequency-perception crater detection method developed based on YOLO11n. First, a Frequency-Directional Attention Module (FDA-Module) is introduced into the shallow stage of the backbone. This module combines frequency-aware channel attention and direction-aware spatial attention to enhance the representation of crater rim structures, elevation variations, and directional topographic cues in DEM data. Second, a C2PSA-LRSA module is designed by embedding Local Region Self-Attention into the C2PSA framework, thereby improving local contextual feature interaction while reducing the excessive cost associated with global self-attention. Third, Inner-WIoU is adopted to replace the original CIoU loss in YOLO11n. By combining the auxiliary-box mechanism of Inner-IoU with the sample-quality-aware weighting strategy of WIoU, Inner-WIoU provides a more flexible bounding-box regression objective for craters with weak rims, scale variations, and uncertain boundaries. A DEM-based lunar crater dataset was constructed from the Moon LRO LOLA–SELENE Kaguya TC DEM Merge 60N60S 59m product and the Robbins lunar crater catalog, covering the non-polar region from 60° S to 60° N and containing 4760 image tiles. Under the random data-splitting strategy, FPW-YOLO11n achieves 78.3% Precision, 66.2% Recall, 75.1% mAP@0.5, and 50.2% mAP@0.5:0.95, outperforming the YOLO11n baseline by 1.2, 2.0, 1.6, and 4.0 percentage points, respectively. Additional experiments based on geographically disjoint data splitting further show that the proposed method consistently performs better than YOLO11n on DEM data, indicating that the proposed structural improvements remain effective under a more rigorous spatially independent evaluation setting. Although the computational cost increases from 6.3 to 24.0 GFLOPs, FPW-YOLO11n maintains a compact parameter size of 2.59 M and a high inference speed, demonstrating an improved accuracy–efficiency trade-off for lunar crater detection from DEM data.

## 1. Introduction

Impact craters are among the most prominent geological features on the lunar surface. They are mainly formed by meteorite or asteroid impacts and typically exhibit a bowl-shaped morphology, with a relatively flat floor surrounded by elevated rim walls. The study of lunar impact craters has a long history and has provided important scientific support for understanding the geological evolution of the Moon. In addition, systematic investigations of lunar craters have offered valuable references for lunar landing site selection, surface exploration, and the potential exploitation of lunar resources [1,2,3].

According to their morphological characteristics, lunar impact craters can be classified into five representative categories: Albategnius C, Biot, Sosigenes, Triesneker, and Tycho [4]. With the development of orbital imaging technology, high-resolution lunar remote sensing images have been obtained by onboard cameras. These data mainly include charge-coupled device (CCD) images and digital elevation model (DEM) data, which provide rich visual and topographic information for crater analysis [5,6,7]. Based on these global lunar remote sensing datasets, various lunar impact crater databases have been progressively established through manual annotation, automated labeling, or a combination of both approaches [8,9,10]. These databases provide reliable coordinate and morphological information for crater-related studies and have become an important foundation for subsequent crater detection experiments.

However, lunar impact craters are not completely static geological structures. Their shapes, sizes, and surface appearances may gradually change under the influence of various geological and environmental processes, such as space weathering [11] and moonquakes [12]. By analyzing variations in crater morphology, researchers can estimate the ages of lunar impact craters and provide a scientific basis for determining the relative stratigraphic ages of newly observed craters [13]. Meanwhile, these analyses also contribute to the continuous updating and refinement of lunar crater databases. Therefore, pre-established crater databases not only provide coordinate information for known craters but also support further detection, classification, and evolutionary analysis of lunar impact craters.

Initial crater detection methods mainly relied on traditional image-processing techniques, including morphology-based algorithms, object-oriented flooding methods, template matching strategies, edge detection, and the Hough transform [14,15,16,17,18]. These methods provided useful early solutions for crater identification and offered interpretable detection procedures based on geometric or texture-related features. However, the lunar surface is characterized by complex and highly variable topography, uneven illumination, shadows, terrain degradation, and other environmental disturbances. Under such conditions, traditional image-processing methods often show limited robustness and usually require manual intervention when dealing with special or anomalous cases. This makes the detection process time-consuming, labor-intensive, and prone to errors. To improve detection efficiency and accuracy, researchers gradually introduced machine learning methods, such as support vector machines and random forests, into crater detection tasks [19,20].

In 2012, the convolutional neural network AlexNet achieved breakthrough performance in the ImageNet Large Scale Visual Recognition Challenge 2012 (ILSVRC-2012), significantly reducing the error rate in image classification [21]. This achievement marked an important milestone in the development of deep learning and demonstrated its strong potential in computer vision and image processing. In the field of object detection, deep learning-based detectors, such as Faster R-CNN, YOLO, SSD, RetinaNet, and EfficientDet, have become widely used because of their strong automatic feature learning capability and favorable real-time performance [22,23,24,25,26]. As a result, CNN-based methods have been increasingly adopted for lunar impact crater detection.

Using lunar remote sensing images acquired by the Lunar Reconnaissance Orbiter Camera (LROC) Narrow-Angle Camera (NAC) [27], Chen et al. [28] employed an improved Faster R-CNN to identify small lunar craters and estimate their ages, demonstrating the effectiveness of CNN models in small-crater detection. Jiang et al. [29] developed a multi-type landform detector based on a lightweight SSD model, which showed good robustness for landform detection under different environmental conditions. Bickel et al. [30] applied RetinaNet to detect lunar rockfalls and distinguish them from craters while also evaluating the detection speed and performance of the model.

Although these deep learning models have shown promising performance in crater detection, YOLO-based detectors have attracted particular attention because of their fast inference speed and relatively low false-positive rates. Different versions of the YOLO series have been investigated and applied in this research direction. Qiu et al. [31] constructed a small-scale CNN model based on YOLOv5, improving both processing speed and detection accuracy. Zhu et al. [32,33] proposed improved YOLOv7-based crater detection methods by introducing the convolutional block attention module (CBAM) and deformable convolutional modules, achieving better performance than the original YOLOv7 model. These studies indicate that YOLO models are effective for lunar impact crater detection, especially for small craters that are easily overlooked by other detection models. The ability of YOLOv7 to identify numerous small craters further suggests the potential of YOLO-based architectures for improving crater feature detection.

In addition to CNN-based methods, transformer-based models have recently become an emerging research direction for crater detection because of their ability to model global contextual information. Unlike CNNs, transformers rely mainly on attention mechanisms and do not depend on recurrent operations or convolutional structures [34]. Guo et al. [35] proposed Crater-DETR, the first transformer-based model applied to crater detection, and achieved encouraging results. Zhang et al. [36] adopted Swin Transformer [37] as a feature extractor for lunar impact crater detection. Although transformer-based models can achieve strong performance in many scenarios, they generally require more computational resources and longer processing time than CNN-based models due to their architectural design. Therefore, for practical lunar crater detection tasks, especially those requiring efficient inference on large-scale remote sensing images, it remains important to balance detection accuracy, model complexity, and computational efficiency.

To address the above challenges, this paper proposes FPW-YOLO11n, a frequency-perception framework built upon the YOLO11n architecture for lunar impact crater detection. The proposed model is designed to improve crater feature representation, enhance the detection of small and weak-boundary craters, and refine bounding-box regression under complex lunar topographic conditions. The main contributions of this work are summarized as follows:

1. Frequency-directional feature enhancement: a Frequency-Directional Attention Module (FDA-Module) is introduced to strengthen shallow feature representation. By combining frequency-aware channel attention and direction-aware spatial attention, the module helps the network capture crater rim structures, elevation variations, and directional shadow-related cues in lunar DEM data.

2. Local region self-attention: a C2PSA-LRSA module is introduced by embedding local region self-attention (LRSA) into the C2PSA framework. The module adaptively captures spatial dependencies within local regions, allowing the network to better represent crater rims, shadows, and nearby terrain structures. Compared with global self-attention, it limits attention computation to local windows, thereby reducing computational cost while retaining useful local contextual information. This helps improve the detection of small and weak-boundary lunar craters under complex terrain conditions.

3. Improved bounding-box regression: to better adapt the regression loss to the morphological characteristics of lunar craters, Inner-WIoU is adopted to replace the original CIoU loss in YOLO11n. By combining the auxiliary-box mechanism of Inner-IoU with the sample-quality-aware weighting strategy of Wise-IoU (WIoU), the proposed loss provides a more flexible localization constraint for craters with weak rims, scale variations, and uncertain boundaries.

4. A lunar impact crater detection dataset is constructed using the Moon LRO LOLA–SELENE Kaguya TC DEM Merge 60N60S 59m product and the Robbins lunar crater catalog. The study area covers non-polar lunar regions from 60° S to 60° N, and the global DEM is cropped into 4760 image tiles. This dataset provides a stable experimental basis for training, validating, and evaluating deep learning-based lunar crater detection models.

The remainder of this paper is organized as follows. Section 2 describes the data sources and dataset generation process, including the selection of lunar DEM data, the use of the Robbins crater catalog, coordinate conversion, image tiling, and label preparation. Section 3 introduces the proposed FPW-YOLO11n framework, with emphasis on the FDA-Module, the C2PSA-LRSA module, and the Inner-WIoU loss function. Section 4 presents the experimental settings, evaluation metrics, ablation studies, comparative experiments, training-curve analysis, and qualitative visualization results. Finally, Section 5 concludes the paper and discusses the limitations of the current study as well as possible directions for future work.

## 2. Materials and Methods

### 2.1. Data Sources and Catalog Preparation

To construct a reliable dataset for lunar impact crater detection, it is necessary to select image data with broad surface coverage, sufficient spatial resolution, and accurate topographic representation. At the same time, the corresponding crater catalog should provide abundant and trustworthy annotations across a wide range of crater diameters. Considering these requirements, this study uses the Moon LRO LOLA–SELENE Kaguya TC DEM Merge 60N60S 59m product as the basic topographic data source [38] (included in the Supplementary Material). This dataset, commonly referred to as SLDEM2015, is a merged lunar digital elevation model derived from two complementary sources: the Lunar Orbiter Laser Altimeter (LOLA; NASA Goddard Space Flight Center, Greenbelt, MD, USA) onboard NASA’s Lunar Reconnaissance Orbiter (LRO), and the Terrain Camera (TC; Japan Aerospace Exploration Agency/Institute of Space and Astronautical Science, Sagamihara City, Kanagawa, Japan) carried by JAXA’s SELENE/Kaguya mission. The LOLA measurements provide a precise geodetic reference framework, whereas the SELENE/Kaguya TC data contribute high-resolution stereo-derived terrain information. By integrating these two datasets, the merged DEM offers detailed and geometrically consistent lunar topographic information for crater morphology analysis.

**Figure 1:**
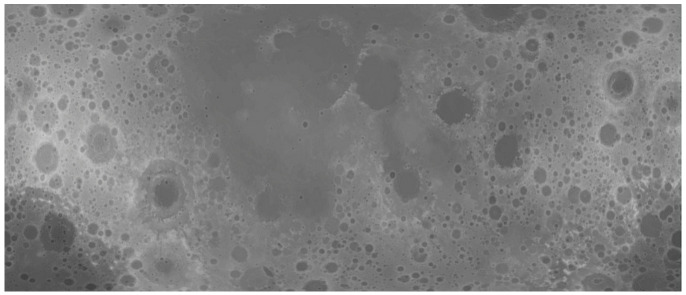
Sample of the Moon LRO LOLA–SELENE Kaguya TC DEM Merge 60N60S 59m product. The dataset is available from https://planetarymaps.usgs.gov/mosaic/LolaKaguya_Topo/Lunar_LRO_LOLAKaguya_DEMmerge_60N60S_512ppd.tif (accessed on 1 March 2025).

The selected DEM product covers the lunar region between 60° S and 60° N, with a spatial resolution of approximately 59 m per pixel. This resolution is suitable for extracting the morphological characteristics of impact craters in non-polar lunar regions. Compared with optical image mosaics, DEM data describe elevation changes directly and can more clearly represent crater-related terrain structures, including crater rims, floors, walls, and surrounding relief. Therefore, this DEM product provides an appropriate data foundation for constructing a crater detection dataset and for training deep learning models to learn topographic features associated with lunar craters. According to the product description, SLDEM2015 was generated by combining a large number of SELENE TC DEM tiles with LOLA elevation measurements, resulting in a near-global lunar DEM with an effective resolution of about 60 m near the equator and a typical vertical accuracy of approximately 3–4 m. An example is shown in Figure 1.

**Figure 2:**
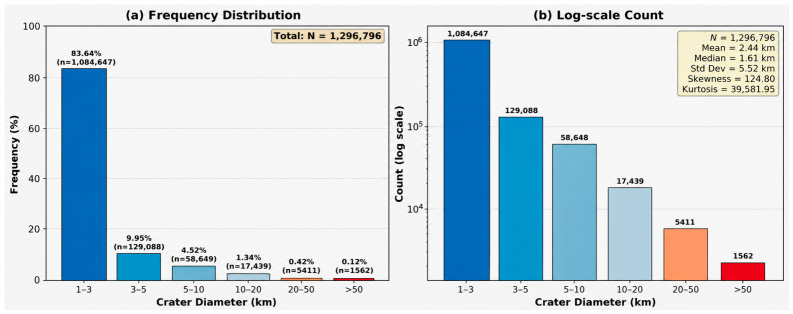
Crater diameter frequency distribution with summary statistics and log-scale view.

For ground-truth annotation, this study adopts the lunar impact crater catalog developed by Robbins [9]. This catalog was selected because of its broad spatial coverage, large number of crater records, and relatively high annotation reliability. It contains approximately 1.3 million lunar impact craters and is regarded as nearly complete for craters larger than about 1–2 km in diameter. Detailed information on the data distribution is presented in Figure 2. In addition, the crater entries were manually identified and measured using multiple lunar datasets, including LROC Wide-Angle Camera (WAC; Malin Space Science Systems, San Diego, CA, USA) images, Lunar Orbiter Laser Altimeter (LOLA; NASA Goddard Space Flight Center, Greenbelt, MD, USA) topographic data, and SELENE/Kaguya Terrain Camera (TC; Japan Aerospace Exploration Agency, Sagamihara, Kanagawa, Japan) images were used in this study, and the merged LOLA–TC digital terrain model. As a result, the catalog provides reliable crater location and size information for supervised model training. By combining the SLDEM2015 data with the Robbins crater catalog, this study establishes a stable data basis for crater sample generation, model training, validation, and performance evaluation. Moreover, since the Robbins catalog has been widely used in lunar geological research, it also provides a credible reference for comparing deep learning-based crater detection methods.

### 2.2. Dataset Generation and Split

After orthorectification, we converted the geographic coordinates of craters in the Robbins catalog into pixel coordinates in each image tile. We then cropped the global mosaic into tiles of 3° × 3°, and we generated the label files for each tile. For each tile, crater annotations were encoded in the YOLO format as [0, *x_center*, *y_center*, *w*, *h*], and all values were normalized to [0, 1].

**Figure 3:**
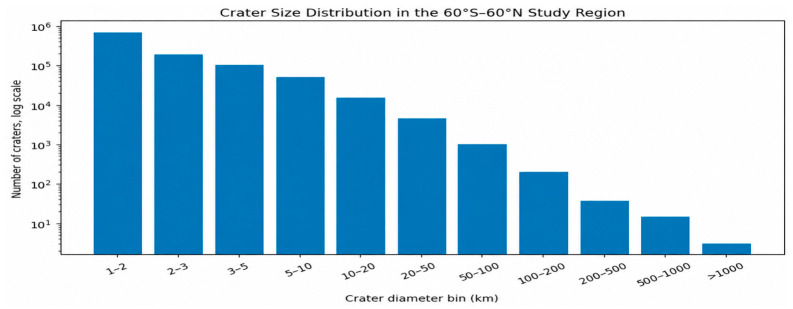
Crater size distribution within the 60° S–60° N study region. This figure shows the number of craters in different diameter intervals. A logarithmic *y*-axis is used because the crater population is highly dominated by small craters.

**Figure 4:**
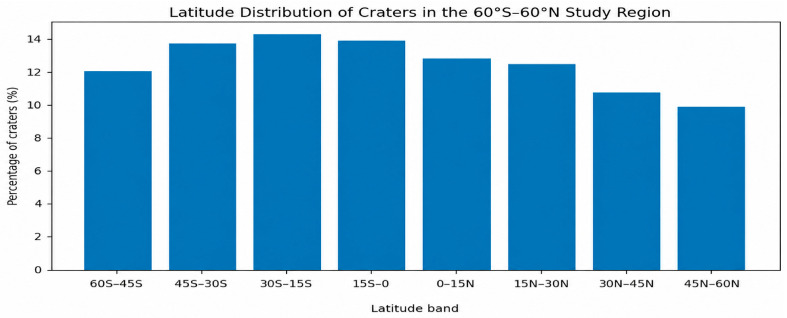
Latitude distribution of craters within the 60° S–60° N study region. This figure presents the proportion of craters in different latitude bands, illustrating the spatial coverage of the selected study area.

**Figure 5:**
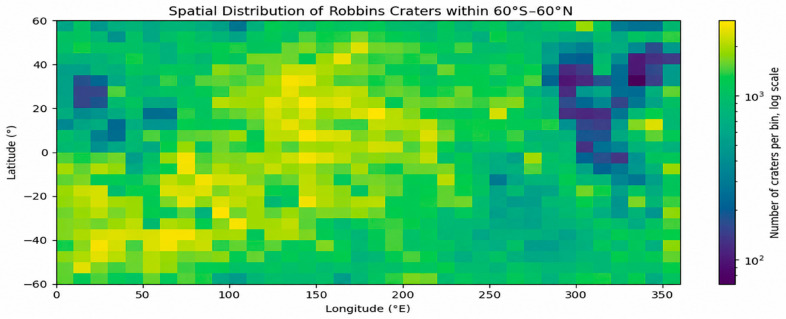
Longitude–latitude spatial distribution of Robbins craters within the 60° S–60° N study region. This figure visualizes the spatial density of crater centers after excluding polar regions outside 60° S–60° N.

To further characterize the generated dataset, we statistically analyzed the crater annotations derived from the Robbins catalog within the study region. Since the image tiles used in this study cover the latitude range from 60° S to 60° N, craters outside this range were excluded from the statistical analysis. After filtering, 1,042,701 craters were retained from the original 1,296,796 global crater records, accounting for approximately 80.41% of the Robbins catalog. The median and mean crater diameters within the study region are 1.61 km and 2.47 km, respectively. Craters smaller than 5 km account for 93.29% of the records, while craters smaller than 10 km account for 98.00%, indicating that the dataset is dominated by small craters but still covers a wide range of crater sizes. In addition, the spatial distribution of crater centers was analyzed in both latitude and longitude directions, showing that crater annotations are widely distributed across the selected 60° S–60° N region. The crater size and spatial distributions are shown in Figure 3, Figure 4 and Figure 5.

**Figure 6:**
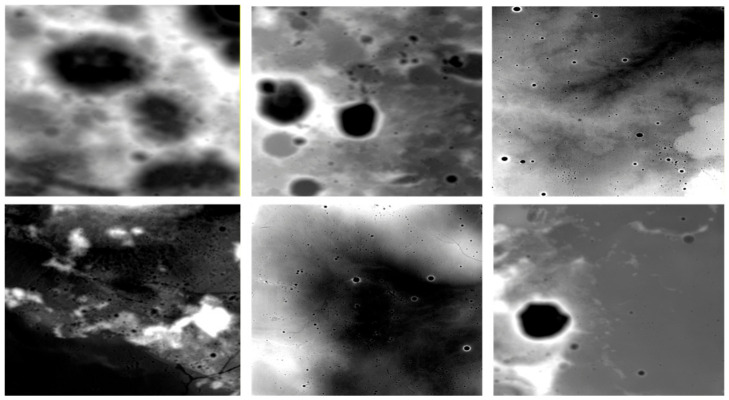
Sample cropped image patches used in building the dataset.

With this pipeline, we obtained a dataset with 4760 image tiles. We randomly split the tiles into training, validation, and test sets at a ratio of 8:1:1. Example tiles are shown in Figure 6. The numbers of tiles in the training, validation, and test sets are 3808, 476, and 476, respectively.

## 3. Methods

### 3.1. YOLO11n Architecture

Object detection is a fundamental task in computer vision. It aims not only to determine whether objects of interest are present in an image, but also to accurately predict their spatial locations. With the rapid development of deep learning, especially the widespread application of convolutional neural networks (CNNs), object detection methods have achieved substantial improvements in both accuracy and inference efficiency. Among existing detection frameworks, YOLO (You Only Look Once) has received considerable attention because of its end-to-end detection pipeline and strong real-time performance. By directly predicting object categories and bounding boxes from the input image in a single forward pass, YOLO has become one of the most widely used one-stage detection frameworks in practical applications.

**Figure 7:**
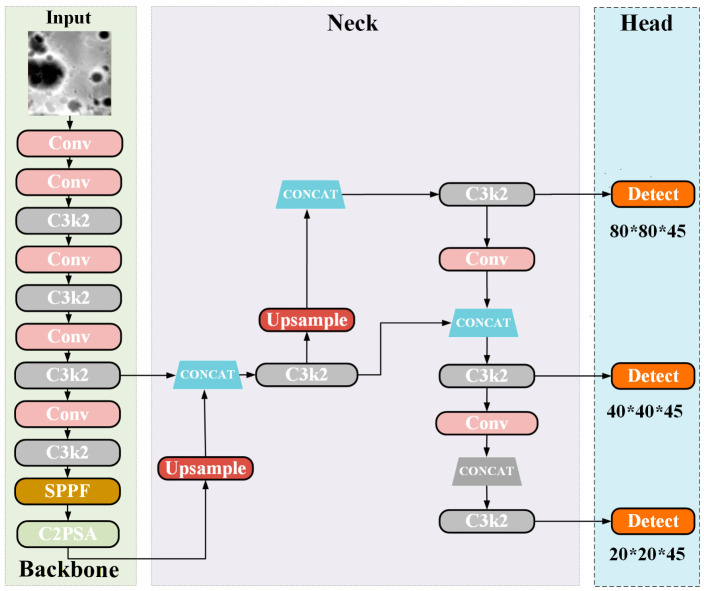
YOLO11n Network Architecture Diagram.

With continuous development, the YOLO family has undergone multiple iterations, and its network architecture, feature fusion strategy, and training mechanism have been gradually improved. Ultralytics released YOLO11 on 10 September 2024, and its overall architecture is shown in Figure 7. Compared with previous versions, YOLO11 further improves model performance and extends its applicability to multiple vision tasks. In addition to object detection, YOLO11 supports instance segmentation, image classification, pose estimation, and oriented bounding box (OBB) detection. These extensions enhance its generalization ability across different scenarios and make it more suitable for diverse deployment requirements.

Structurally, YOLO11 improves the backbone, neck, and detection head to strengthen feature extraction, multi-scale fusion, and prediction efficiency. In the backbone network, two important modules, C3K2 and C2PSA, are introduced. C3K2 is an enhanced convolutional module with higher configurability than C2f, allowing the model scale to be adjusted according to different task requirements. C2PSA combines partial self-attention with a feed-forward network to improve feature extraction and contextual modeling. In addition, the optional residual connection in C2PSA helps improve gradient propagation, which is beneficial for training stability and model convergence. These structural improvements provide stronger feature representations for subsequent feature aggregation and object prediction.

The neck of YOLO11 adopts a PAN-based structure to enhance the transfer and fusion of multi-scale features. By incorporating C3K2 into the neck, semantic information from different feature levels can be more effectively aggregated. This design is particularly useful for objects with large scale variations, as it enables the network to combine high-level semantic information with lower-level spatial details. Meanwhile, the detection head is optimized using depthwise separable convolutions, which reduce the number of parameters and computational cost while maintaining competitive detection accuracy. Through these improvements, YOLO11 achieves a more balanced trade-off between detection accuracy and inference speed in complex visual scenes.

In addition to architectural improvements, YOLO11 also modifies the training and loss design to improve detection performance. The traditional objectness branch is removed, simplifying the prediction structure. For classification, binary cross-entropy (BCE) loss is still used to supervise category prediction. For bounding-box regression, YOLO11 combines Distribution Focal Loss (DFL) with Complete IoU (CIoU) loss. DFL helps improve the modeling of bounding-box distributions, while CIoU provides geometric constraints by considering overlap area, center-point distance, and aspect-ratio consistency. This combination is helpful for improving localization accuracy, especially in cases where object boundaries are unclear or the background is complex.

To satisfy different accuracy and deployment requirements, the YOLO11 series provides five model scales with different depths and widths. Among them, YOLO11n is the nano-scale version, designed for scenarios requiring fast inference and low computa-tional cost. It has fewer parameters and lower FLOPs while still maintaining reasonable detection capability. Considering the characteristics of the lunar impact crater dataset used in this study, YOLO11n is selected as the baseline model. Lunar crater detection requires the model to identify numerous small- and medium-sized targets from complex terrain backgrounds. Therefore, a compact detector with real-time potential and good feature extraction ability is suitable for this task. Based on these considerations, YOLO11n provides an appropriate foundation for subsequent structural optimization and loss function improvement in this study.

### 3.2. Improved FPW-YOLO11n for Lunar Crater Detection

**Figure 8:**
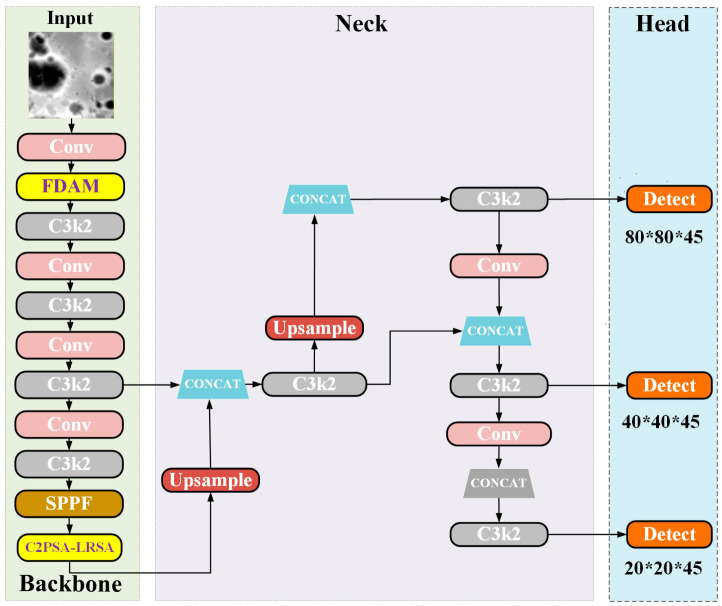
FPW-YOLO11n network architecture diagram.

To better adapt YOLO11n to the characteristics of lunar crater detection, this study proposes an improved model named FPW-YOLO11n. The model is designed to strengthen feature representation and reduce missed detections of small craters while maintaining a compact parameter size and relatively high inference speed. Through these modifications, FPW-YOLO11n aims to improve detection accuracy under complex lunar terrain conditions. The overall architecture of the improved FPW-YOLO11n network is shown in Figure 8.

#### 3.2.1. Frequency-Directional Attention Module

**Figure 9:**
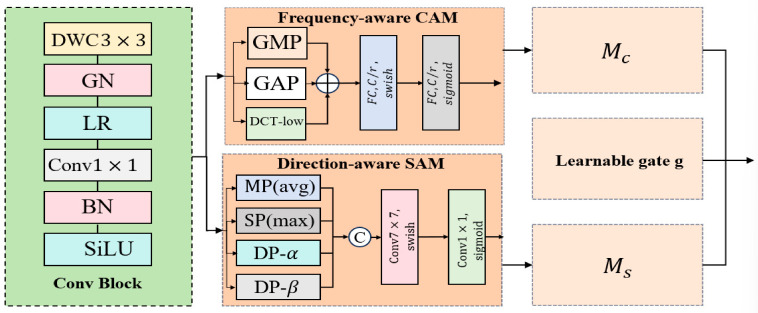
Architecture of the Frequency-Directional Attention Module (FDA).

To address the imaging characteristics of lunar crater detection—namely, the substantial scale variation among targets, the highly regularized annular morphology of craters, and the constraint imposed by a single illumination direction—we propose the Frequency-Directional Attention Module (FDA-Module). The detailed architecture is illustrated in Figure 9. The proposed module extends the conventional channel–spatial attention paradigm along two complementary dimensions: a frequency-domain branch that captures the periodic spectral signature of crater rims, and a directional pooling branch that exploits the consistent shadow orientation induced by the solar azimuth. A learnable gate is further introduced to adaptively balance the channel and spatial responses, allowing the module to accommodate craters of diverse scales.

Given an input feature map $X\in R^{C\times H\times W}$, the FDA-Module first applies a convolutional block (Conv block) to extract local representations. The Conv Block adopts an inverted-bottleneck structure consisting of a 3 × 3 depth-wise convolution (DWC) followed by group normalization (GN) and a LeakyReLU activation (LR), and a 1 × 1 point-wise convolution followed by Batch Normalization (BN) and a SiLU activation. The hybrid normalization design—GN at the depth-wise stage and BN at the point-wise stage—stabilizes statistical estimation under small training batches while preserving the channel-wise discriminability of point-wise features:

(1) $$F=SiLU\left(BN\left(f_{1\times 1}\left(LR\left(GN\left(f_{3\times 3}^{dw}\left(X\right)\right)\right)\right)\right)\right),$$

where X denotes the input feature map and F denotes the output feature map. $f_{3\times 3}^{dw}$ represents a 3 × 3 depth-wise convolution, which is used to extract local spatial features. GN denotes Group Normalization, LR denotes the Leaky ReLU activation function, and *f*_1 × 1_ represents a 1 × 1 convolution for channel information fusion. BN denotes Batch Normalization and SiLU denotes the SiLU activation function. Overall, this computational process first extracts local features through depth-wise convolution and then combines normalization, nonlinear activation, and 1 × 1 convolution to achieve feature enhancement and channel fusion.

Frequency-aware Channel Attention. The annular shadow–highlight structure of lunar craters exhibits a pronounced low-frequency concentration in the spectral domain, which conventional global pooling operators are unable to capture explicitly. To this end, we augment the channel attention pathway with a low-frequency Discrete Cosine Transform (DCT) branch in parallel with Global Average Pooling (GAP) and Global Maximum Pooling (GMP). For the c-th channel of F, the three descriptors are computed as

(2) $$\begin{array}{c} z_{c}^{GAP}=\frac{1}{HW}\sum_{i=1}^{H}\;\sum_{j=1}^{W}\;F_{c,i,j},\;\;z_{c}^{GMP}=\underset{i,j}{max}\;F_{c,i,j}, \end{array}$$

(3) $$\begin{array}{c} z_{c}^{DCT}=\sum_{i=0}^{H-1}\;\sum_{j=0}^{W-1}\;F_{c,i,j}B_{i,j}^{u,v},B_{i,j}^{u,v}=\cos\left(\frac{\pi \left(2i+1\right)u}{2H}\right)\cos\left(\frac{\pi \left(2j+1\right)v}{2W}\right), \end{array}$$

where (u,v) indexes the selected low-frequency component of the DCT-II basis. The three descriptors are aggregated and passed through a two-layer bottleneck network with a reduction ratio r to produce the channel attention weight:

(4) $$\begin{array}{c} M_{c}=\sigma \left(W_{2}\delta \left(W_{1}\left(z^{GAP}+z^{GMP}+z^{DCT}\right)\right)\right), \end{array}$$

where $W_{1}\in R^{(C/r)\times C}$ and $W_{2}\in RC\times (C/r)$ are the weights of the bottleneck layers, δ(⋅) denotes the swish activation, and σ(⋅) is the sigmoid function. The resulting weight $M_{c}\in R^{C\times 1\times 1}$ emphasizes channels carrying salient spectral signatures of crater rims.

Direction-aware Spatial Attention. Within a single lunar image, the solar azimuth remains constant, resulting in highly consistent shadow–highlight orientations across all craters in the frame. We exploit this physical prior by introducing two directional pooling operators, DP-α and DP-β, alongside the conventional mean and maximum pooling. DP-α is implemented as a 1×k horizontal strip pooling that aggregates contextual information along the rows of the feature map, while DP−β s implemented as a k×1 vertical strip pooling along the columns. Together, they encode the directional gradient pattern produced by the unidirectional illumination. Let $S^{avg},S^{max},S^{\alpha },\;and\;S^{\beta }$ denote the four pooled descriptors. The directional spatial attention weight is obtained as

(5) $$\begin{array}{c} S=\mathrm{Concat}\left(S^{avg},S^{max},S^{\alpha },S^{\beta }\right), \end{array}$$

(6) $$\begin{array}{c} M_{s}=\sigma \left(f_{1\times 1}\left(\delta \left(f_{7\times 7}\left(S\right)\right)\right)\right), \end{array}$$

where $f_{k\times k}(\cdot )$ denotes a convolution with kernel size k × k. The resulting weight $M_{s}\in R^{1\times H\times W}$ is sensitive to directional gradients, which facilitates accurate localization of crater rims under the low-contrast conditions characteristic of lunar surface imagery.

Gated Parallel Fusion: Rather than applying channel and spatial attention sequentially as in conventional designs [ref], we fuse the two attention maps in parallel through a learnable scalar gate g ∈ (0,1), which is implemented as a sigmoid-activated learnable parameter $g=\sigma \left(\hat{g}\right)\;with\;\hat{g}\in R.$ The gate adaptively balances the two attention pathways according to the dominant target scale: small craters rely more heavily on spatial cues, whereas large craters benefit from channel-wise discrimination. The final output of the FDA-Module is computed with a residual connection as

(7) $$\begin{array}{c} X_{out}=F⊗\left(g\cdot M_{c}\right)⊗\left(\left(1-g\right)\cdot M_{s}\right)⊕X, \end{array}$$

where ⊗ denotes broadcasted element-wise multiplication and ⊕ denotes element-wise addition. The residual path preserves the original feature flow and stabilizes optimization at the shallow stage of the backbone.

Module Placement. The FDA-Module is inserted at the P2 stage of the YOLO11 backbone, immediately after the first downsampling layer. At this stage, the feature resolution remains sufficiently high to retain the edge and texture cues of small craters before they are attenuated by successive downsampling operations, while the computational overhead of the attention pathways remains tractable. By embedding both spectral and directional priors into the early feature representation, the FDA-Module provides discriminative cues for subsequent multi-scale aggregation and detection heads.

#### 3.2.2. Local Region Self-Attention Module

**Figure 10:**
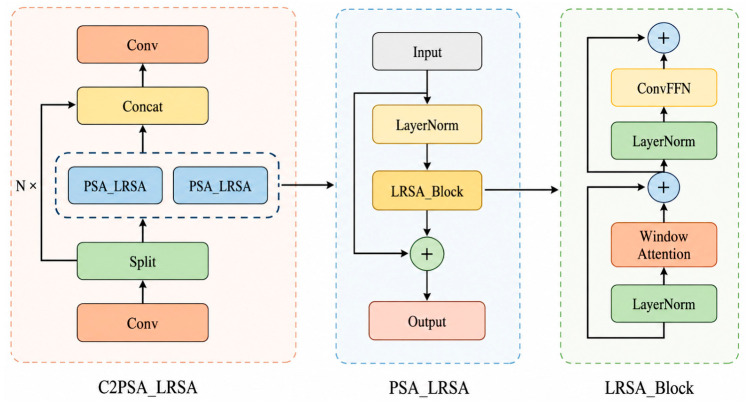
Architecture of the C2PSA-LRSA.

To enhance local feature representation, this study introduces the C2PSA_LRSA module by embedding the Local Region Self-Attention (LRSA) mechanism into the C2PSA framework. The LRSA mechanism establishes attention-based interactions among different spatial positions within a local region, allowing the network to measure the relevance between neighboring features. As a result, the module can emphasize informative local structures and suppress less relevant responses, thereby improving the utilization of discriminative regional information. The architecture of the C2PSA_LRSA module is illustrated in Figure 10.

First, a normalization operation is applied to the input feature map to reduce scale differences across channels, which provides a more stable feature representation for subsequent attention computation. The normalized features are then fed into the Window Attention module. In this module, the feature map is partitioned into several local windows. For each window, the features are projected into query, key, and value representations using the shared projection matrices $W_{Q},W_{K},\;and\;W_{V}$, respectively. Self-attention is then calculated within each local window, enabling the model to capture local spatial dependencies among neighboring pixels.

After the window-based attention operation, a residual connection is used to merge the attention-enhanced features with the original input, which helps preserve feature information during local representation refinement. The fused feature map is subsequently normalized again before being passed into the convolutional feed-forward network. Finally, the ConvFFN layer performs channel expansion and compression to further enhance feature transformation, while the final residual connection maintains smooth information flow and improves training stability. The process can be formulated as follows:

(8) $$\begin{array}{c} X_{out}=MSA\left(X_{0}W_{Q},X_{0}W_{K},X_{0}W_{V}\right) \end{array}$$

In the above equations, $W_{Q},W_{K},\;and{\;W}_{V}$ denote the learnable projection matrices for query, key, and value generation, respectively, while MSA represents the multi-head self-attention operation.

In object detection tasks, conventional convolutional operations are constrained by fixed receptive fields, which limits their ability to capture fine edge textures and local structural details of small objects. Although global self-attention can model long-range dependencies among different spatial positions, its computational complexity increases quadratically with the spatial size of the feature map. This makes it less suitable for real-time detection scenarios, especially when processing high-resolution features. To address these limitations, this study proposes a hybrid attention enhancement module named C2PSA_LRSA, which aims to improve channel, spatial, and local feature modeling while maintaining a lightweight computational design.

Specifically, a 1 × 1 convolution is first used to reduce the channel dimension of the input feature map. The resulting feature is then divided into two branches. The main branch contains several PSA_LRSA submodules, which combine lightweight local-region self-attention, ConvFFN, and residual connections to enhance multi-scale spatial representations. The other branch serves as a shortcut path and preserves local details through identity mapping. Afterward, the features from the two branches are concatenated and passed through another 1 × 1 convolution to restore the channel dimension.

Compared with the original C2PSA structure, the proposed C2PSA_LRSA replaces standard multi-head self-attention with the lightweight LRSA mechanism. This design reduces computational complexity and improves inference efficiency while retaining competitive detection accuracy. Moreover, because attention computation is restricted to local regions, the module is more suitable for high-resolution feature maps and can better support small-object detection tasks.

#### 3.2.3. Loss Function for Inner-WIoU

In the baseline YOLO11 detector, the Complete IoU (CIoU) loss is adopted for bounding-box regression. Compared with the standard IoU loss, CIoU considers not only the overlap area between the predicted bounding box and the ground-truth box, but also the normalized center distance and the aspect-ratio consistency. Therefore, CIoU provides a more comprehensive geometric constraint for object localization. Given a predicted bounding box B=(x,y,w,h), Bgt = $\left(x^{gt},y^{gt},w^{gt},h^{gt}\right)$, the CIoU loss can be formulated as follows:

(9) $$\begin{array}{c} L_{CIoU}=1-IoU+\frac{\rho^{2}\left(b,b^{gt}\right)}{c^{2}}+\alpha v \end{array}$$

where

(10) $$\begin{array}{c} IoU=\frac{\left|B\cap B^{gt}\right|}{\left|B\cup B^{gt}\right|+\varepsilon } \end{array}$$

(11) $$\begin{array}{c} v=\frac{4}{\pi^{2}}{\left(\arctan\frac{w^{gt}}{h^{gt}}-\arctan\frac{w}{h}\right)}^{2} \end{array}$$

(12) $$\begin{array}{c} \alpha =\frac{v}{1-IoU+v+\varepsilon } \end{array}$$

In these equations, IoU denotes the intersection-over-union between the predicted and ground-truth boxes. b = (x, y) and $b^{gt}=\left(x^{gt},{\;y}^{gt}\right)$ are the center points of the two boxes. $\rho^{2}(b,b^{gt})$ is the squared Euclidean distance between the two center points. c denotes the diagonal length of the smallest enclosing box covering both B and $B^{gt}$. The term v measures the aspect-ratio consistency, while α is an adaptive weighting factor. ε is a small constant used to avoid numerical instability.

Although CIoU is effective for general object detection, it may not be fully suitable for Lunar impact crater detection. Lunar craters usually exhibit approximately circular or bowl-shaped structures, and their boundaries are often degraded by illumination changes, shadows, surface erosion, and complex terrain. Under such conditions, the aspect-ratio penalty in CIoU may provide limited additional discrimination, because most crater boxes have similar width-to-height ratios. Moreover, CIoU treats different regression samples in a relatively uniform manner. For low-quality samples, such as heavily shadowed craters, partially occluded craters, or craters with ambiguous rims, excessive regression constraints may introduce unstable gradients. For high-quality samples, CIoU may also lack a more flexible mechanism to accelerate fine-grained localization. Therefore, a more adaptive bounding-box regression loss is required to improve localization robustness for craters with weak edges, varying scales, and uncertain boundaries.

To improve the adaptability of bounding-box regression, Wise-IoU (WIoU) introduces a distance attention term and a dynamic focusing mechanism. Instead of uniformly strengthening all samples, WIoU assigns different gradient gains according to the quality of anchor boxes. This strategy reduces the adverse influence of low-quality samples and avoids overemphasizing high-quality samples, allowing the model to focus more on ordinary-quality samples that are more beneficial for training. The basic form of WIoU can be expressed as:

(13) $$\begin{array}{c} L_{WIoU}^{v1}=R_{WIoU}L_{IoU} \end{array}$$

where

(14) $$\begin{array}{c} L_{IoU}=1-IoU \end{array}$$

(15) $$\begin{array}{c} R_{WIoU}=\exp\left(\frac{\rho^{2}\left(b,b^{gt}\right)}{c^{2}}\right) \end{array}$$

Here, $R_{WIoU}$ is the distance attention term. $\rho^{2}(b,b^{gt})$ denotes the squared center distance between the predicted and ground-truth boxes, and $c^{2}$ denotes the squared diagonal length of the smallest enclosing box.

In WIoU v3, a dynamic non-monotonic focusing coefficient is further introduced. The outlier degree β is defined as

(16) $$\begin{array}{c} \beta =\frac{L_{IoU}^{*}}{\overset{¯}{L_{IoU}}} \end{array}$$

where $L_{IoU}^{*}$ denotes the IoU loss detached from gradient propagation, and $\overset{¯}{L_{IoU}}$ is the running mean of the IoU loss during training. The dynamic focusing coefficient is formulated as

(17) $$\begin{array}{c} r_{f}=\frac{\beta }{\delta \alpha^{\beta -\delta }} \end{array}$$

where α and δ are hyperparameters controlling the non-monotonic focusing curve. Therefore, the WIoU v3 loss can be written as

(18) $$\begin{array}{c} L_{WIoU}^{v3}=r_{f}R_{WIoU}L_{IoU} \end{array}$$

Compared with CIoU, WIoU reduces the reliance on aspect-ratio constraints and introduces sample-quality-aware gradient allocation. This is more suitable for Lunar crater detection, where crater boundaries may be ambiguous and the quality of bounding-box samples varies significantly.

Although WIoU improves the sample weighting strategy, its localization term is still based on the original bounding boxes. To further improve the flexibility of bounding-box regression, Inner-IoU introduces auxiliary bounding boxes controlled by a scale ratio. Instead of calculating IoU directly from the original boxes, Inner-IoU computes the overlap between the scaled auxiliary boxes. This design allows the regression process to be adjusted according to different detection tasks and sample qualities. For high-IoU samples, a smaller auxiliary box can provide stricter localization supervision, while for low-IoU samples, a larger auxiliary box can offer more tolerant and stable regression guidance.

Given the predicted box B=(x,y,w,h), the auxiliary predicted box $B_{r}$ is defined as

(19) $$\begin{array}{c} B_{r}=\left(x-\frac{rw}{2},y-\frac{rh}{2},x+\frac{rw}{2},y+\frac{rh}{2}\right) \end{array}$$

Similarly, for the ground-truth box $B^{gt}=(x^{gt},y^{gt},w^{gt},h^{gt})$, the auxiliary ground-truth box $B_{r}^{gt}$ is defined as

(20) $$\begin{array}{c} B_{r}^{gt}=\left(x^{gt}-\frac{rw^{gt}}{2},y^{gt}-\frac{rh^{gt}}{2},x^{gt}+\frac{rw^{gt}}{2},y^{gt}+\frac{rh^{gt}}{2}\right) \end{array}$$

where r is the scaling ratio used to control the size of the auxiliary boxes. The Inner-IoU is then calculated as

(21) $$\begin{array}{c} IoU_{inner}=\frac{\left|B_{r}\cap B_{r}^{gt}\right|}{\left|B_{r}\cup B_{r}^{gt}\right|+\varepsilon } \end{array}$$

and the corresponding Inner-IoU loss is

(22) $$\begin{array}{c} L_{Inner-IoU}=1-IoU_{inner} \end{array}$$

where r < 1 produces smaller auxiliary boxes and r > 1 produces larger auxiliary boxes. In Lunar crater detection, this flexible auxiliary-box mechanism is useful because craters may have weak rims, irregular shadows, and different degrees of boundary degradation.

Based on the above analysis, this study further combines the adaptive sample-quality weighting ability of WIoU with the auxiliary-box regression mechanism of Inner-IoU. The resulting Inner-WIoU loss replaces the original IoU term in WIoU with $IoU_{inner}$, enabling the detector to perform more flexible and quality-aware bounding-box regression for Lunar impact craters.

(23) $$\begin{array}{c} L_{Inner-WIoU}^{v3}=\frac{\beta_{inner}}{\delta \alpha^{\beta_{inner}-\delta }}\cdot \exp\left(\frac{\rho^{2}\left(b,b^{gt}\right)}{c^{2}}\right)\left(1-IoU_{inner}\right) \end{array}$$

Through this formulation, Inner-WIoU inherits the distance-aware and sample-quality-aware properties of WIoU, while also benefiting from the auxiliary-box mechanism of Inner-IoU. This makes the regression loss more suitable for Lunar impact crater detection, where targets often exhibit circular shapes, weak boundaries, scale variations, and uncertain edge responses.

To make the present study easier to reproduce, we have provided the detailed parameter settings for the improved components described in Section 3.2.1, Section 3.2.2 and Section 3.2.3. The specific parameters are presented in Table 1.

## 4. Experimental Result and Analysis

### 4.1. Experimental Setups

#### 4.1.1. Experimental Environment

All experiments were conducted using the PyTorch framework version 2.2.2 on an Ubuntu 22.04 system. The main hardware configuration and experimental settings are summarized in Table 2.

#### 4.1.2. Training Parameters

During training, all input images were uniformly resized to 640 × 640 pixels. The detailed hyperparameter settings are provided in Table 3.

### 4.2. Evaluation Metrics

To evaluate the detection performance of the proposed model, Precision (P), Recall (R), and mAP@0.5 are used as the main accuracy metrics, as defined in Equations (24)–(27). In addition, the number of parameters and GFLOPs are reported to assess model complexity and computational cost, respectively. Frames per second (FPS) is also adopted to evaluate inference efficiency. Together, these metrics provide a comprehensive assessment of detection accuracy, model complexity, and real-time performance.

(24) $$\begin{array}{c} P=\frac{TP}{TP+FP} \end{array}$$

(25) $$\begin{array}{c} R=\frac{TP}{TP+FN} \end{array}$$

(26) $$\begin{array}{c} AP=\int_{0}^{1}\;P\left(R\right)dR \end{array}$$

(27) $$\begin{array}{c} mAP@0.5=\frac{1}{C}\sum_{c=1}^{C}\;AP_{0.5}^{\left(c\right)} \end{array}$$

where *TP* represents true positives; *FP* represents false positives; *FN* represents false negatives; *AP_i_* indicates the average precision of the *i*-th category; the area under the *PR* curve corresponds to the *AP* value; C is the total number of categories.

### 4.3. Ablation Experiments

In this experiment, the dataset remains consistent with that described in Section 2.1 and Section 2.2. The research area is restricted to 60° N to 60° S, and the minimum selected crater diameter is set to 1 km. To further evaluate the contribution of each proposed component, an extended ablation study was conducted, as shown in Table 4. In addition to Precision, Recall, mAP@0.5, mAP@0.5:0.95, Params, and GFLOPs, FPS is also reported to evaluate the inference speed of different model variants.

Setting A denotes the baseline YOLO11n model without any additional modules. The baseline achieves 77.1% Precision, 64.2% Recall, 73.5% mAP@0.5, and 46.2% mAP@0.5:0.95, with 2.58 M parameters and 6.3 GFLOPs. Its inference speed reaches 128.46 FPS, indicating that the baseline model has high computational efficiency and fast inference capability.

When the FDA module is introduced individually in Setting B, the detection performance improves across all accuracy metrics. Specifically, Precision increases from 77.1% to 77.7%, Recall improves from 64.2% to 65.4%, mAP@0.5 rises from 73.5% to 74.4%, and mAP@0.5:0.95 increases from 46.2% to 49.4%. The improvement in mAP@0.5:0.95 is particularly evident, suggesting that the FDA module helps improve localization accuracy under stricter IoU thresholds. The number of parameters only slightly increases from 2.58 M to 2.59 M. However, GFLOPs increase from 6.3 to 24.0, and FPS decreases from 128.46 to 125.76. This indicates that the FDA module improves detection accuracy and localization quality at the cost of increased computational complexity, although the actual decrease in inference speed remains limited.

With the C2PSA-LRSA module added individually in Setting C, the model also obtains stable performance improvements. Precision and Recall increase to 77.6% and 65.0%, respectively, while mAP@0.5 and mAP@0.5:0.95 reach 74.1% and 49.0%. Compared with the baseline, C2PSA-LRSA improves mAP@0.5:0.95 by 2.8 percentage points. Meanwhile, GFLOPs remain unchanged at 6.3, and FPS only slightly decreases from 128.46 to 127.34. This result suggests that C2PSA-LRSA can effectively enhance feature representation and localization ability while introducing very limited computational overhead.

Setting D evaluates the effect of the Inner-WIoU loss function. After replacing the original regression loss with Inner-WIoU, the model achieves 77.4% Precision, 64.4% Recall, 73.9% mAP@0.5, and 46.6% mAP@0.5:0.95. Since the number of parameters and GFLOPs remain unchanged, the performance gain mainly comes from improved bounding-box regression optimization rather than increased model complexity. In addition, the FPS remains almost unchanged, decreasing only slightly from 128.46 to 128.12. This shows that Inner-WIoU can improve localization constraints with almost no additional inference cost, although its independent performance gain is relatively moderate.

Settings E, F, and G further evaluate the combined effects of two components. When the FDA module and C2PSA-LRSA are combined in Setting E, the model achieves 77.9% Precision, 65.4% Recall, 74.9% mAP@0.5, and 49.7% mAP@0.5:0.95. These results are higher than those obtained by using either FDA or C2PSA-LRSA alone in most metrics, indicating that the two structural modules can complement each other in feature extraction and multi-scale representation. However, due to the introduction of the FDA module, GFLOPs remain at 24.0, and FPS decreases to 124.87.

When the FDA module and Inner-WIoU are combined in Setting F, the model achieves 77.9% Precision, 65.4% Recall, 74.7% mAP@0.5, and 49.3% mAP@0.5:0.95. Compared with using the FDA module alone, the addition of Inner-WIoU provides a slight improvement in mAP@0.5 and maintains stable Recall. The FPS is 125.21, which is close to Setting B, indicating that Inner-WIoU does not introduce additional computational burden during inference.

In Setting G, C2PSA-LRSA and Inner-WIoU are combined without the FDA module. The model achieves 77.8% Precision, 65.2% Recall, 74.3% mAP@0.5, and 49.2% mAP@0.5:0.95, with 2.60 M parameters and 6.3 GFLOPs. The FPS reaches 126.86, which is only slightly lower than that of the baseline. This result further confirms that C2PSA-LRSA and Inner-WIoU can improve detection performance while maintaining high inference efficiency.

Finally, when all three components are integrated, the proposed model achieves the best overall detection performance. Compared with the baseline, Precision improves from 77.1% to 78.3%, Recall increases from 64.2% to 66.2%, mAP@0.5 rises from 73.5% to 75.1%, and mAP@0.5:0.95 improves from 46.2% to 50.2%. These results correspond to gains of 1.2, 2.0, 1.6, and 4.0 percentage points, respectively. The final model maintains a compact parameter size of 2.59 M, while GFLOPs increase to 24.0. In terms of inference speed, FPS decreases from 128.46 to 124.23, corresponding to a reduction of approximately 3.29%. Although the computational complexity increases substantially in terms of GFLOPs, the actual FPS reduction is relatively limited under the experimental hardware environment.

Overall, the extended ablation results demonstrate that the FDA module, C2PSA-LRSA, and Inner-WIoU contribute to crater detection from different perspectives. The FDA module mainly improves feature extraction and localization accuracy, especially under stricter IoU thresholds, but it is also the main source of the increased GFLOPs. C2PSA-LRSA enhances feature representation with limited computational cost and only a slight decrease in FPS. Inner-WIoU improves bounding-box regression without increasing model parameters or GFLOPs. Their combination achieves the best detection accuracy, while maintaining acceptable inference speed. These results indicate that the proposed model provides a reasonable trade-off between detection accuracy, model complexity, and inference efficiency for crater detection tasks.

### 4.4. Comparative Experiment and Analysis of Test Results with Different Network Models

Table 5 presents the comparison results between the proposed method and several representative object detection models under the same evaluation settings. The compared models include lightweight YOLO-series detectors, such as YOLOv6n, YOLOv8n, YOLOv9t, YOLOv12n, and YOLO11n, as well as the transformer-based detector RT-DETR-R18. To comprehensively evaluate detection accuracy, model complexity, and inference efficiency, the number of parameters, GFLOPs, Precision, Recall, mAP@0.5, mAP@0.5:0.95, and FPS are reported.

As shown in Table 4, the proposed method achieves the best detection accuracy among all compared models. Compared with the YOLO11n baseline, the proposed method improves Precision from 77.1% to 78.3% and Recall from 64.2% to 66.2%. In terms of mAP, mAP@0.5 increases from 73.5% to 75.1%, while mAP@0.5:0.95 increases from 46.2% to 50.2%. The improvement of 4.0 percentage points in mAP@0.5:0.95 indicates that the proposed method provides a clear benefit for more accurate bounding-box localization under stricter IoU thresholds. Meanwhile, the number of parameters increases only slightly from 2.58 M to 2.59 M, suggesting that the performance gain is not mainly achieved by increasing the model size.

Compared with other lightweight YOLO detectors, the proposed method also demonstrates strong competitive advantages. For example, YOLOv8n achieves 73.4% mAP@0.5 and 44.4% mAP@0.5:0.95, while YOLOv12n obtains 73.2% and 44.0% on the two metrics, respectively. In contrast, the proposed method reaches 75.1% mAP@0.5 and 50.2% mAP@0.5:0.95, outperforming these lightweight baselines in both overall detection accuracy and high-precision localization quality. In particular, the significant improvement in mAP@0.5:0.95 suggests that the proposed structural improvements and regression optimization strategy are beneficial for precise crater localization.

In terms of inference efficiency, the proposed method achieves 124.23 FPS. Although this is slightly lower than YOLO11n with 128.46 FPS and YOLOv12n with 129.64 FPS, the proposed method still maintains a high inference speed and can satisfy real-time or near-real-time detection requirements. Compared with YOLO11n, the FPS decreases by only 4.23, approximately 3.3%, while Precision, Recall, mAP@0.5, and mAP@0.5:0.95 are all improved. Therefore, the proposed method achieves a reasonable balance between detection accuracy and inference efficiency.

Compared with RT-DETR-R18, the proposed method shows clear advantages in accuracy, parameter size, computational cost, and inference speed. RT-DETR-R18 contains 19.87 M parameters, requires 56.9 GFLOPs, and achieves 82.42 FPS, while its mAP@0.5 and mAP@0.5:0.95 are only 66.3% and 40.3%, respectively. By contrast, the proposed method uses only 2.59 M parameters and 24.0 GFLOPs, while achieving 75.1% mAP@0.5 and 50.2% mAP@0.5:0.95, with an FPS of 124.23. This comparison indicates that, for the lunar crater detection task evaluated in this study, the proposed method is more lightweight and efficient than the transformer-based detector while also providing superior detection performance.

It should also be noted that the proposed method introduces a higher computational cost than the original YOLO11n baseline, with GFLOPs increasing from 6.3 to 24.0. This increase is mainly caused by the additional feature enhancement modules and attention-related components. Nevertheless, the proposed method maintains an almost unchanged parameter size and still achieves an inference speed above 120 FPS, while obtaining clear improvements in Precision, Recall, and mAP, especially under the stricter mAP@0.5:0.95 metric. Overall, the comparison results in Table 4 demonstrate that the proposed method effectively improves lunar crater detection accuracy while preserving a lightweight parameter scale and high inference speed, providing a favorable trade-off between detection performance and computational complexity.

As shown in Table 4, the proposed method achieves the best overall detection accuracy among the compared models, with 78.3% Precision, 66.2% Recall, 75.1% mAP@0.5, and 50.2% mAP@0.5:0.95. Compared with the YOLO11n baseline, the proposed method improves Precision by 1.2 percentage points, Recall by 2.0 percentage points, mAP@0.5 by 1.6 percentage points, and mAP@0.5:0.95 by 4.0 percentage points. The most significant improvement is observed under the stricter mAP@0.5:0.95 metric, indicating that the proposed method enhances the bounding-box localization accuracy for lunar crater detection. Meanwhile, the parameter size increases only slightly from 2.58 M to 2.59 M, while the FPS decreases from 128.46 to 124.23. This suggests that the proposed method achieves higher detection accuracy while maintaining a lightweight model scale and a high inference speed.

**Figure 11:**
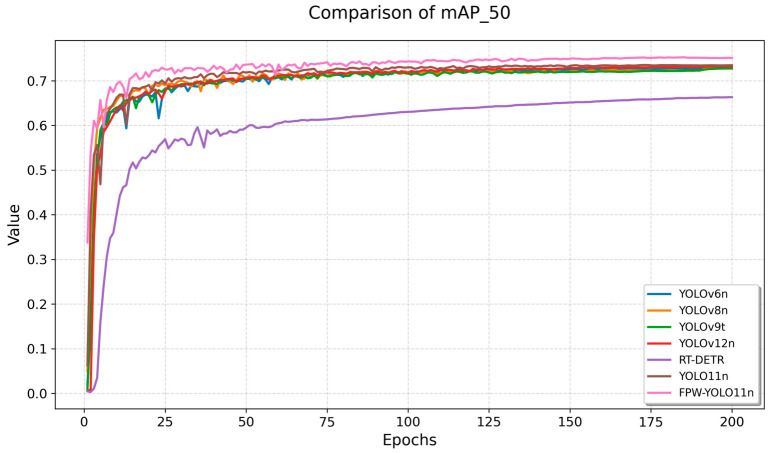
Comparison of mAP curves between different algorithms.

To further analyze the training behavior of different detectors, the mAP@0.5 curves of representative YOLO-series models and the DETR-based method are plotted over the training epochs under the same experimental environment and hyperparameter settings, as shown in Figure 11. These curves provide a more intuitive comparison of convergence speed and training stability. In addition to the final quantitative results in Table 4, the training curves help reveal how rapidly each model improves during training and whether its performance remains stable after convergence. Combined with the results in Table 4, the proposed method not only obtains the highest final mAP@0.5, but also maintains a favorable balance among detection accuracy, model complexity, and inference efficiency.

As shown in Figure 11, the mAP@0.5 values of all models increase rapidly during the early training stage and then gradually become stable. The proposed method, denoted as FPW-YOLO11n in the figure, maintains a consistently higher curve than the compared models, especially in the middle and later training stages. Compared with lightweight YOLO detectors, including YOLOv6n, YOLOv8n, YOLOv9t, YOLOv12n, and YOLO11n, the proposed method not only converges to a higher final mAP@0.5, but also shows relatively stable performance after convergence, indicating good training stability and a higher detection performance ceiling.

Combined with the quantitative results in Table 4, the proposed method achieves a final mAP@0.5 of 75.1%, outperforming YOLO11n with 73.5%, YOLOv8n with 73.4%, YOLOv12n with 73.2%, YOLOv6n with 73.0%, and YOLOv9t with 72.7%. From the trend of the curves in Figure 11, the proposed method gradually establishes a stable advantage after approximately 20 epochs. This suggests that the improved structure can more effectively learn discriminative features of lunar craters and continuously enhance detection performance during training. In particular, the FPW-YOLO11n curve still shows a slight upward trend in the later training stage, while most YOLO baselines tend to reach a performance plateau, further demonstrating the stronger feature representation capability and optimization potential of the proposed method.

Compared with the transformer-based RT-DETR-R18, the advantage of the proposed method is more evident. The mAP@0.5 curve of RT-DETR-R18 increases more slowly and finally stabilizes at around 0.66, which is consistent with its mAP@0.5 of 66.3% reported in Table 4. In contrast, the proposed method reaches 75.1% mAP@0.5 and remains clearly higher than RT-DETR-R18 throughout the training process. This indicates that, for the lunar crater detection task investigated in this study, the proposed method achieves faster convergence and higher detection accuracy than RT-DETR-R18.

Although the computational cost of the proposed method increases from 6.3 GFLOPs in YOLO11n to 24.0 GFLOPs, its parameter size only slightly increases from 2.58 M to 2.59 M, and the FPS remains high at 124.23, only slightly lower than the 128.46 FPS of YOLO11n. Therefore, the results in Figure 11, together with Table 4, demonstrate that the proposed method achieves higher and more stable mAP@0.5 performance while maintaining a lightweight parameter scale and high inference speed. The training curves further verify the comprehensive advantages of the improved algorithm in terms of detection accuracy, convergence stability, and practical inference efficiency.

### 4.5. Visualization of Detection Results

**Figure 12:**
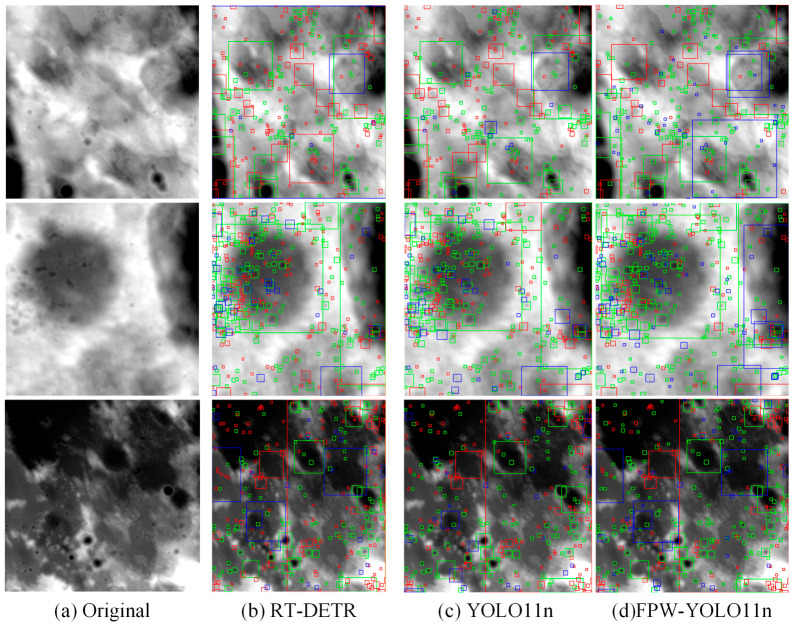
Qualitative comparison of crater detection results on representative Lunar tiles (green boxes indicate correctly detected targets, blue boxes indicate false detections, and red boxes indicate missed detections).

To further evaluate the effectiveness of the improved model, three image tiles from different regions were selected, and three detection algorithms were used for qualitative comparison. The detailed visual comparison results are shown in Figure 12. Each image tile contains a certain number of medium-to-large craters as well as multiple small craters, which is consistent with the typical spatial distribution characteristics of lunar impact craters. In the figure, green boxes indicate correctly detected targets, blue boxes indicate false detections, and red boxes indicate missed detections.

Through visual inspection, it can be observed that all three algorithms achieve satisfactory detection performance for medium-to-large craters. However, their detection capabilities differ when dealing with small craters. Since small craters are more numerous and usually have weak boundaries, small scales, and are easily affected by complex terrain, missed detections and false detections of these small craters can significantly influence the final detection accuracy. Therefore, the qualitative results in Figure 12 further indicate that differences in the ability to recognize small craters are one of the important reasons for the overall performance gap among different algorithms.

Quantitative analysis is an important way to evaluate the detection performance of different algorithms. Taking the image tile in the first row as an example, FPW-YOLO11n correctly detects 166 craters, whereas YOLO11n and RT-DETR detect only 120 and 101 craters, respectively. Recall is used to measure the ability of a model to identify true crater targets. The calculated Recall of FPW-YOLO11n reaches 74.44%, which is clearly higher than 53.81% for YOLO11n and 45.29% for RT-DETR. This result indicates that FPW-YOLO11n has a stronger target discovery capability in representative complex regions, especially in reducing missed detections of small craters and craters with weak boundaries.

At the same time, we also observe that the improved algorithm may focus more on small-crater features. As a result, in some medium-to-large crater regions, FPW-YOLO11n may still produce missed detections even when YOLO11n and RT-DETR successfully detect these craters. Therefore, how to further improve the detection performance for small craters while maintaining stable detection capability for medium-to-large craters remains a challenging and interesting direction for future research.

### 4.6. Evaluation Using Spatially Independent Data Splits

In the earlier experiments, the dataset was split into training, validation, and testing subsets at an 8:1:1 ratio based on a fixed random seed. Nevertheless, remote sensing datasets often present intrinsic spatial distribution patterns. When random partitioning is applied, spatial dependence may exist between the training and testing samples, which can result in potential spatial information leakage and an overly optimistic estimation of model generalization. Therefore, additional experiments were designed to provide a more rigorous and objective assessment of the proposed improvements.

For consistency with the previous experimental settings, the study area remained limited to the lunar non-polar region from 60° N to 60° S, and only craters with diameters of at least 1 km were considered. The data split ratio for the training, validation, and testing subsets was also maintained at 8:1:1.

The main difference in this experiment is the use of a geographically separated data partitioning strategy. This approach ensures spatial independence among the training, validation, and testing subsets, thereby minimizing the possibility of spatial information leakage. The hardware platform, software environment, and hyperparameter settings were kept the same as those described in Section 4.1. The corresponding experimental results are reported in Table 6.

According to the results presented in Table 5, the proposed method still outperforms the YOLO11n baseline under the geographically disjoint data splitting strategy. Specifically, the Precision increases from 76.4% to 77.8%, corresponding to an improvement of 1.4 percentage points, which indicates a better ability to suppress false detections. The Recall improves from 62.9% to 65.1%, with a gain of 2.2 percentage points, suggesting that the proposed method can detect more true crater targets.

In terms of overall detection performance, consistent improvements are also observed. The mAP@0.5 increases from 72.1% to 73.9%, showing a gain of 1.8 percentage points. Meanwhile, the stricter mAP@0.5:0.95 improves from 44.8% to 48.9%, corresponding to an increase of 4.1 percentage points. The more pronounced improvement in mAP@0.5:0.95 demonstrates that the proposed method enhances not only crater recognition capability but also bounding-box localization accuracy.

Compared with the results obtained under the random data splitting strategy, the overall metrics change to some extent after adopting the spatially independent partitioning scheme. Nevertheless, the proposed method consistently maintains its advantage over YOLO11n. This indicates that even when the training, validation, and test sets are spatially independent, the proposed method can still achieve better detection performance, thereby reducing the potential influence of spatial information leakage caused by random data partitioning.

It should be noted that the computational cost of the proposed method increases from 6.3 GFLOPs in YOLO11n to 24.0 GFLOPs, while the FPS decreases from 131.47 to 126.82. However, the method still maintains a high inference speed. In addition, the parameter size only slightly increases from 2.58 M to 2.59 M. Overall, the experiment based on spatially disjoint data splitting further confirms that the proposed method performs better than YOLO11n on DEM data, with only limited changes in the overall results. Therefore, at least for the DEM data used in this study, the proposed structural improvements are effective and feasible.

## 5. Conclusions and Future Work

### 5.1. Conclusions

This study proposes FPW-YOLO11n, a frequency-perception object detection method for the automated detection of lunar impact craters. The proposed method is developed based on YOLO11n and aims to address several challenges in lunar crater detection, including large scale variations, weak or degraded crater rims, complex lunar topographic backgrounds, and the requirement for efficient detection on large-scale remote sensing data. Rather than simply increasing the model size, FPW-YOLO11n improves detection performance mainly by enhancing feature representation and optimizing bounding-box regression, while maintaining a compact parameter scale and a relatively high inference speed.

To enhance the representation of crater-related features, a Frequency-Directional Attention Module, namely the FDA-Module, is introduced into the shallow stage of the backbone. This module combines frequency-aware channel attention and direction-aware spatial attention, enabling the network to capture crater rim structures, elevation variations, and directional topographic cues more effectively from lunar DEM data. In addition, a C2PSA-LRSA module is designed by embedding Local Region Self-Attention into the C2PSA structure. By modeling feature interactions within local regions, this module enhances the representation of local geomorphological details while avoiding the excessive computational burden associated with global self-attention. Furthermore, Inner-WIoU is adopted to replace the original CIoU loss in YOLO11n. By integrating the auxiliary-box mechanism of Inner-IoU with the sample-quality-aware weighting strategy of WIoU, Inner-WIoU provides a more flexible bounding-box regression constraint for craters with weak boundaries, large scale variations, and uncertain edge responses.

The ablation experiments demonstrate that the three proposed components contribute to performance improvement to different extents. The YOLO11n baseline achieves 77.1% Precision, 64.2% Recall, 73.5% mAP@0.5, and 46.2% mAP@0.5:0.95. After introducing only the FDA-Module, mAP@0.5 and mAP@0.5:0.95 increase to 74.4% and 49.4%, respectively, indicating that frequency and directional information are effective for enhancing crater-related feature representation. When only C2PSA-LRSA is introduced, mAP@0.5:0.95 improves to 49.0% without increasing GFLOPs, suggesting that local region self-attention can effectively improve high-precision localization. The use of Inner-WIoU alone also improves detection performance, confirming that the modified bounding-box regression loss is beneficial for crater localization. When all three components are jointly applied, FPW-YOLO11n achieves the best overall performance, with 78.3% Precision, 66.2% Recall, 75.1% mAP@0.5, and 50.2% mAP@0.5:0.95. Compared with the YOLO11n baseline, these results represent improvements of 1.2, 2.0, 1.6, and 4.0 percentage points, respectively. The notable improvement in mAP@0.5:0.95 indicates that the proposed method provides better bounding-box localization under stricter IoU thresholds.

Comparative experiments with other advanced detection algorithms further verify the effectiveness of FPW-YOLO11n. Under the same experimental settings, the proposed method achieves 75.1% mAP@0.5 and 50.2% mAP@0.5:0.95, outperforming YOLOv6n, YOLOv8n, YOLOv9t, YOLOv12n, RT-DETR-R18, and YOLO11n. Compared with YOLO11n, the proposed method achieves higher detection accuracy while increasing the parameter size only slightly from 2.58 M to 2.59 M. Compared with RT-DETR-R18, FPW-YOLO11n shows clear advantages in parameter size, computational cost, inference speed, and detection accuracy. Specifically, RT-DETR-R18 contains 19.87 M parameters, requires 56.9 GFLOPs, and achieves 82.42 FPS, whereas FPW-YOLO11n contains 2.59 M parameters, requires 24.0 GFLOPs, and reaches 124.23 FPS, while also achieving higher detection accuracy. These results indicate that FPW-YOLO11n is more suitable for the lunar crater detection task investigated in this study.

It should be noted that although FPW-YOLO11n maintains a compact parameter size and a high inference speed, its computational cost is higher than that of the original YOLO11n baseline, with GFLOPs increasing from 6.3 to 24.0. This increase is mainly caused by the introduction of feature enhancement structures such as the FDA-Module. Therefore, the proposed method should not be regarded as lightweight in all aspects. Instead, it can be considered a detection framework that preserves a compact parameter scale and high inference efficiency while improving detection accuracy at the cost of increased computational complexity. Overall, FPW-YOLO11n achieves improved crater detection accuracy and provides a reasonable balance between parameter size, inference speed, and detection performance. Future work will focus on reducing the computational cost introduced by the FDA-Module and developing feature enhancement modules with lower GFLOPs, thereby further improving the practical deployment potential of the proposed method.

### 5.2. Limitations and Future Work

Although the proposed FPW-YOLO11n improves lunar crater detection performance, several limitations remain, and further improvements are still required in terms of data sources, computational efficiency, input resolution, and comparative evaluation.

First, this study mainly focuses on the non-polar lunar region between 60° S and 60° N. Lunar polar regions are not included because they usually involve more complex DEM projection distortion, illumination variations, and terrain occlusion effects. These factors may lead to different crater morphology representations and feature distributions compared with non-polar regions. Therefore, the generalization ability and robustness of the proposed model in lunar polar environments still require further validation. In future work, polar-region data will be introduced to evaluate the applicability of the proposed method under more challenging lunar surface conditions.

Second, the data currently used for training and testing are limited to DEM data. Although DEMs can effectively represent lunar topographic variations and provide important morphological information for crater identification, a single data source may not fully characterize crater features under different illumination conditions, texture patterns, slope distributions, and geological backgrounds. Future studies will gradually incorporate additional data sources, such as optical images, slope maps, shadow maps, terrain roughness maps, and multi-source fused data. This will allow a more comprehensive evaluation of the detection accuracy of the proposed algorithm under different data conditions and further verify its adaptability and generalization capability in multi-source planetary remote sensing scenarios.

Third, the current dataset mainly considers craters with diameters larger than 1 km. Due to the spatial resolution of the selected DEM data, sub-kilometer craters are difficult to represent clearly and were therefore not included in this study. Future work may incorporate higher-resolution lunar remote sensing data to improve the detection of small-scale craters, degraded craters, and craters with incomplete rims. In addition, all image patches in this study are resized to an input resolution of 640 × 640 pixels. Different input resolutions may affect the scale representation of craters, the preservation of fine-grained morphological details, and the final detection performance of the model. Therefore, future studies will further investigate the performance trends of different algorithms under various input resolutions, aiming to determine a more suitable input scale for lunar crater detection.

Fourth, although the FDA-Module and C2PSA-LRSA effectively enhance feature representation and improve detection accuracy in complex lunar surface environments, the proposed method still introduces a higher computational cost than the original YOLO11n baseline. In particular, the increase in GFLOPs indicates that the current feature enhancement design improves detection performance at the expense of additional computational complexity. Future work will focus on developing a more lightweight FDA module with lower GFLOPs to alleviate the current increase in computational cost and further reduce both parameter size and computational burden. In addition, model compression strategies, such as structural pruning, knowledge distillation, lightweight attention design, and quantization-aware training, will be considered to improve deployment efficiency while preserving detection accuracy as much as possible.

Finally, complex lunar landforms may still cause false detections or missed detections in challenging cases, especially for overlapping craters, heavily degraded crater rims, low-contrast regions, and terrain structures with crater-like appearances. The discrimination ability of the model under such complex geological conditions still needs to be further improved. After the algorithm becomes more stable, future work will compare the proposed method with more crater-specific detection models to provide a more comprehensive evaluation of its performance advantages. Furthermore, the proposed method may be extended to multi-scale crater classification, crater morphological parameter extraction, crater age estimation, and automatic updating of lunar crater databases, thereby further enhancing its value for lunar geological analysis and planetary remote sensing applications.

## Figures and Tables

**Table 1:** The detailed settings of the parameters.

Module	Parameter	Setting
FDA-Module	Selected DCT low-frequency component	DCT-II low-frequency component, (u,v) = (0,0)
FDA-Module	Directional pooling kernel size	7 × 7
Attention module	Reduction ratio	r = 16
LRSA	Window size	7 × 7
LRSA	Number of attention heads	2, implemented as self.c//64
Inner-IoU	Scale ratio	r = 0.7
WIoU	Hyperparameters	WIoU v3, α = 1.9, δ = 3.0, with non-monotonic dynamic focusing

**Table 2:** Experimental platform configuration.

Name	Details
System	Ubuntu 22.04
Graphics Processing Unit	NVIDIA GeForce RTX 4090
Central Processing Unit	Intel Core i9-14900K
GPU Memory	24 G
Memory	64 G (DDR 5 6000 MHZ)
Algorithmic Framework	PyTorch v2.2.2

**Table 3:** Model training hyperparameter settings.

Parameter	Value	Parameter	Value
epochs	200	optimizer	SGD
lr0	0.01	weight decay	0.0005
batch	16	momentum	0.937
imgsz	640	workers	8
lrf	0.01	close mosaic	0
NMS	0.7	confidence thresholds	0.25

**Table 4:** Detection results of the ablation experiment.

Model	FDA Module	C2PSA-LRSA	Inner-WIoU	Params	GFLOPs	P%	R%	mAP@0.5	mAP@0.5:0.95	FPS
A				2.58 M	6.3	77.1	64.2	0.735	0.462	128.46
B	**✓**			2.59 M	24.0	77.7	65.4	0.744	0.494	125.76
C		**✓**		2.60 M	6.3	77.6	65.0	0.741	0.490	127.34
D			**✓**	2.58 M	6.3	77.4	64.4	0.739	0.466	128.12
E	**✓**	**✓**		2.59 M	24.0	77.9	65.4	0.749	0.497	124.87
F	**✓**		**✓**	2.59 M	24.0	77.9	65.4	0.747	0.493	125.21
G		**✓**	**✓**	2.60 M	6.3	77.8	65.2	0.743	0.492	126.86
Ours	**✓**	**✓**	**✓**	2.59 M	24.0	78.3	66.2	0.751	0.502	124.23

**Table 5:** Comparison of Experimental Results of Different Advanced Detection Algorithms.

Model	Params (M)	GFLOPs	P%	R%	mAP@0.5	mAP@0.5:0.95	FPS
YOLOv6n	4.23	11.8	77.0	63.6	0.730	0.438	116.72
YOLOv8n	3.01	8.1	77.0	64.1	0.734	0.444	118.58
YOLOv9t	1.97	7.6	76.9	63.2	0.727	0.435	125.31
YOLOv12n	2.51	5.8	77.4	63.7	0.732	0.440	129.64
RT-DETR-R18	19.87	56.9	71.4	58.7	0.663	0.403	82.42
YOLO11n	2.58	6.3	77.1	64.2	0.735	0.462	128.46
Our Method	2.59	24.0	78.3	66.2	0.751	0.502	124.23

**Table 6:** Experimental Results under Geographically Disjoint Split.

Model	Params(M)	GFLOPs	P%	R%	mAP@0.5	mAP@0.5:0.95	FPS
YOLO11n	2.58	6.3	76.4	62.9	0.721	0.448	131.47
Our Method	2.59	24.0	77.8	65.1	0.739	0.489	126.82

## Data Availability

Data are reported within the article. Further inquiries can be directed to the corresponding authors.

## Supplementary Materials

The following supporting information can be downloaded at: https://www.mdpi.com/article/10.3390/s26144344/s1.
