# Supplementary figures and images for: FPW-YOLO11n: A Lightweight Frequency-Perception Framework for Lunar Impact Crater Detection (part 2 of 2)
Source: Sensors (Basel). 2026 Jul 9;26(14):4344. doi: 10.3390/s26144344 (PMC13417639; doi:10.3390/s26144344)

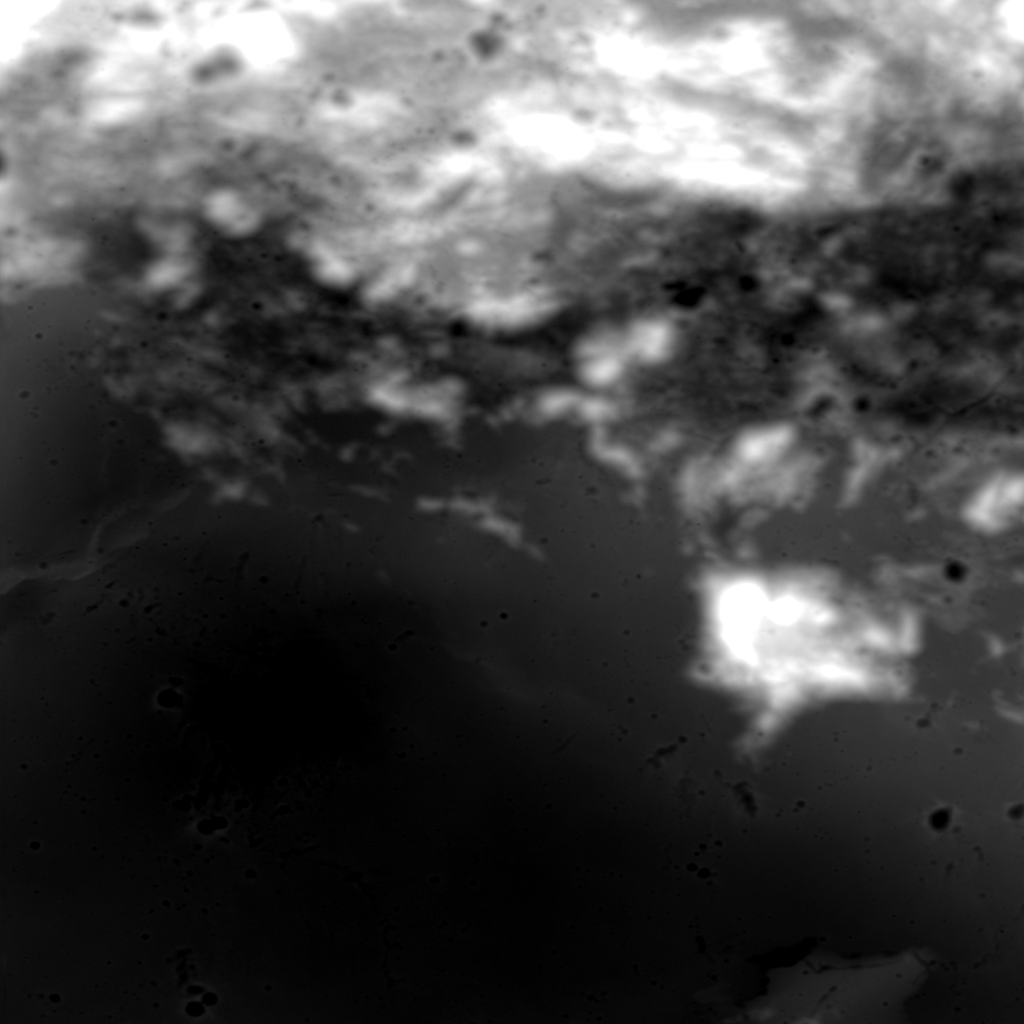

Supplement: Supplementary file 1 [file sensors-26-04344-s001.zip › data/images/test/tile_00905_lon15.0_lat36.0.png]

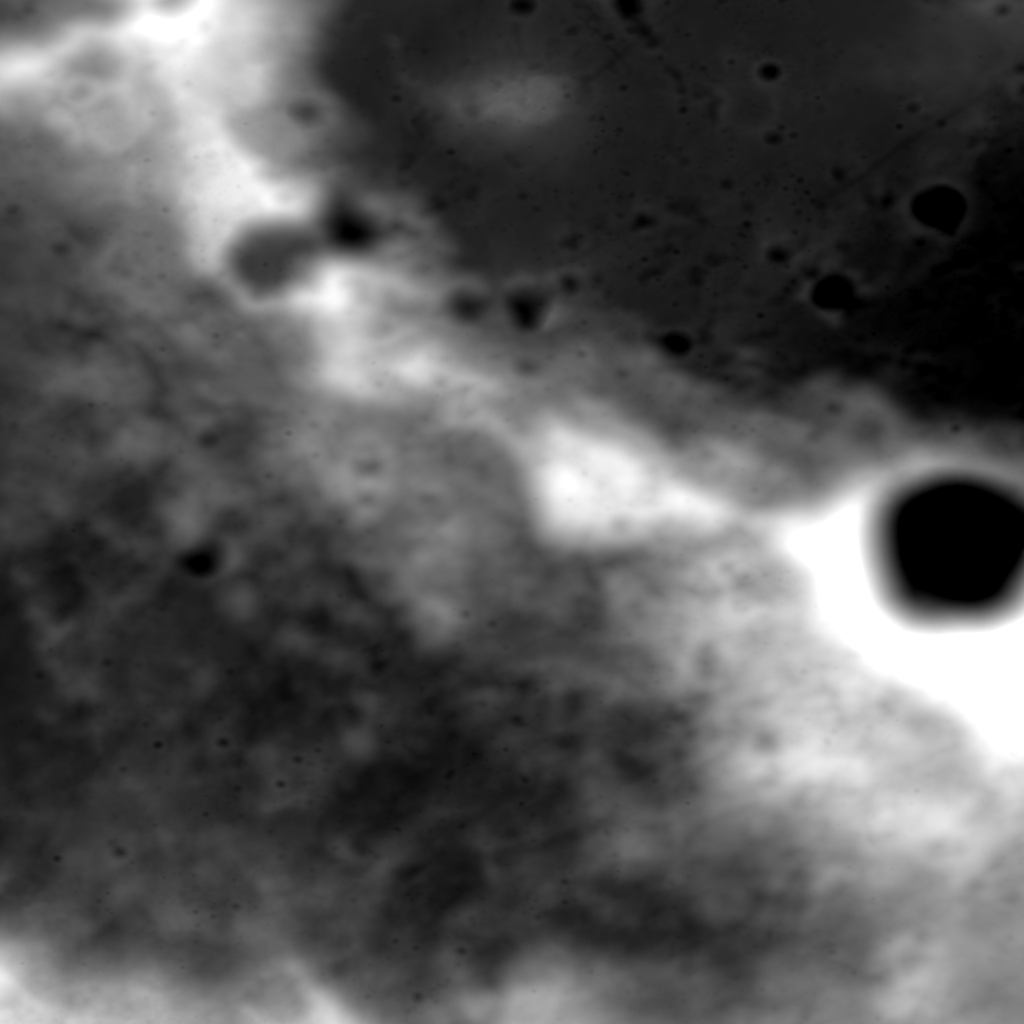

Supplement: Supplementary file 1 [file sensors-26-04344-s001.zip › data/images/test/tile_00919_lon57.0_lat36.0.png]

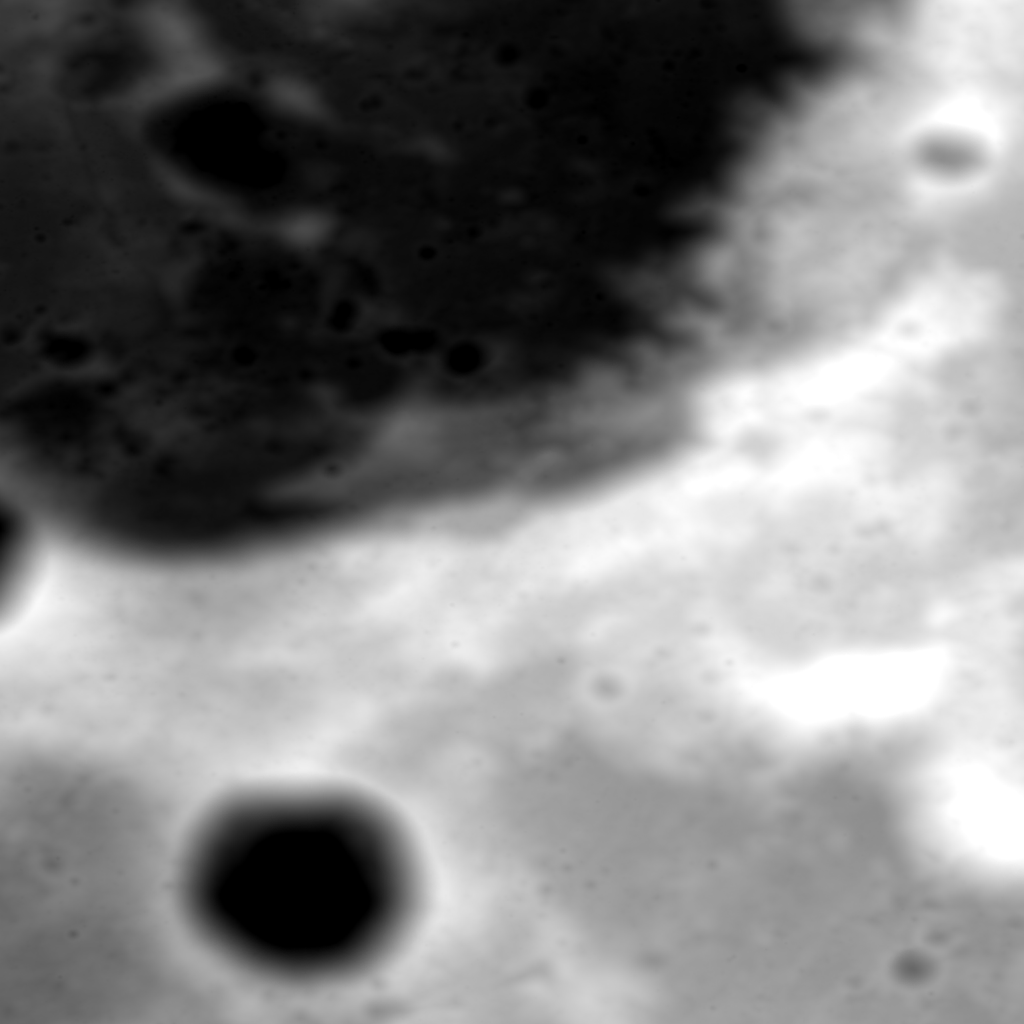

Supplement: Supplementary file 1 [file sensors-26-04344-s001.zip › data/images/test/tile_00920_lon60.0_lat36.0.png]

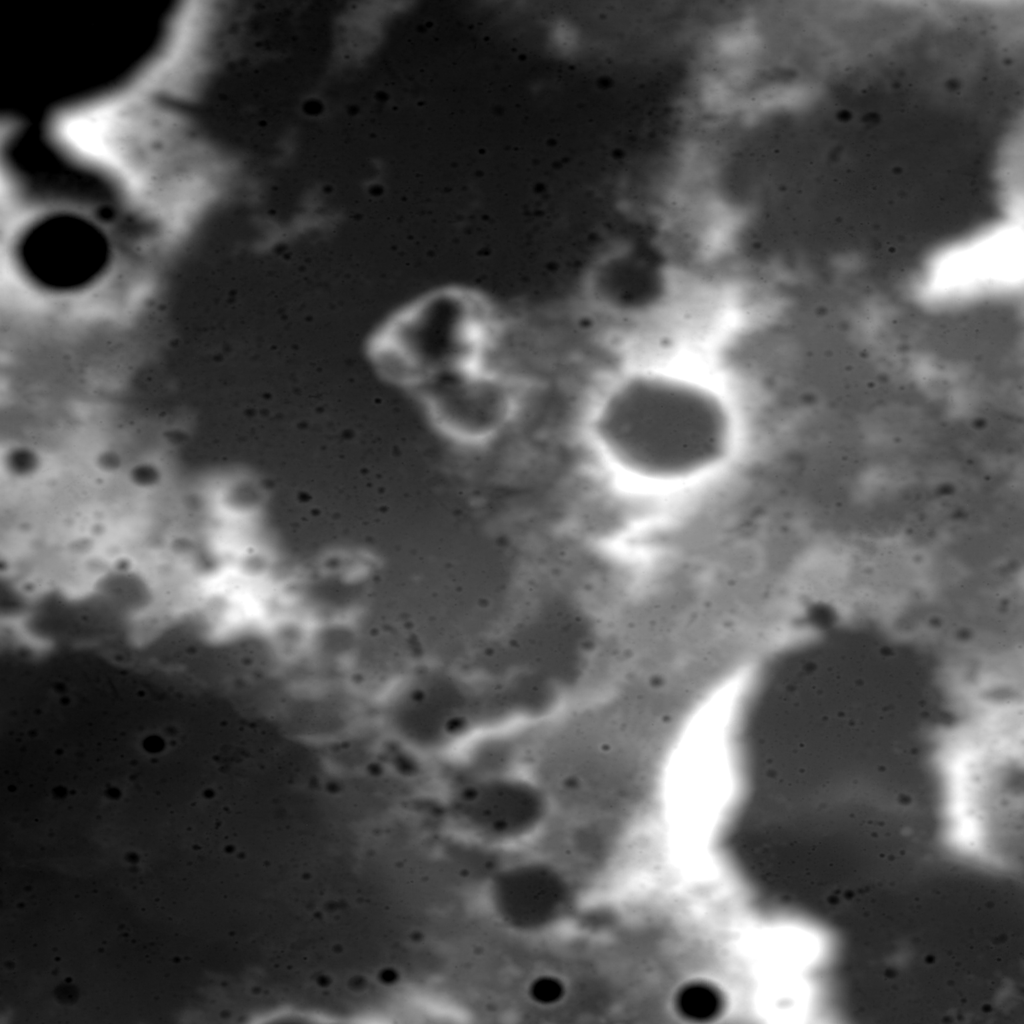

Supplement: Supplementary file 1 [file sensors-26-04344-s001.zip › data/images/test/tile_00923_lon69.0_lat36.0.png]

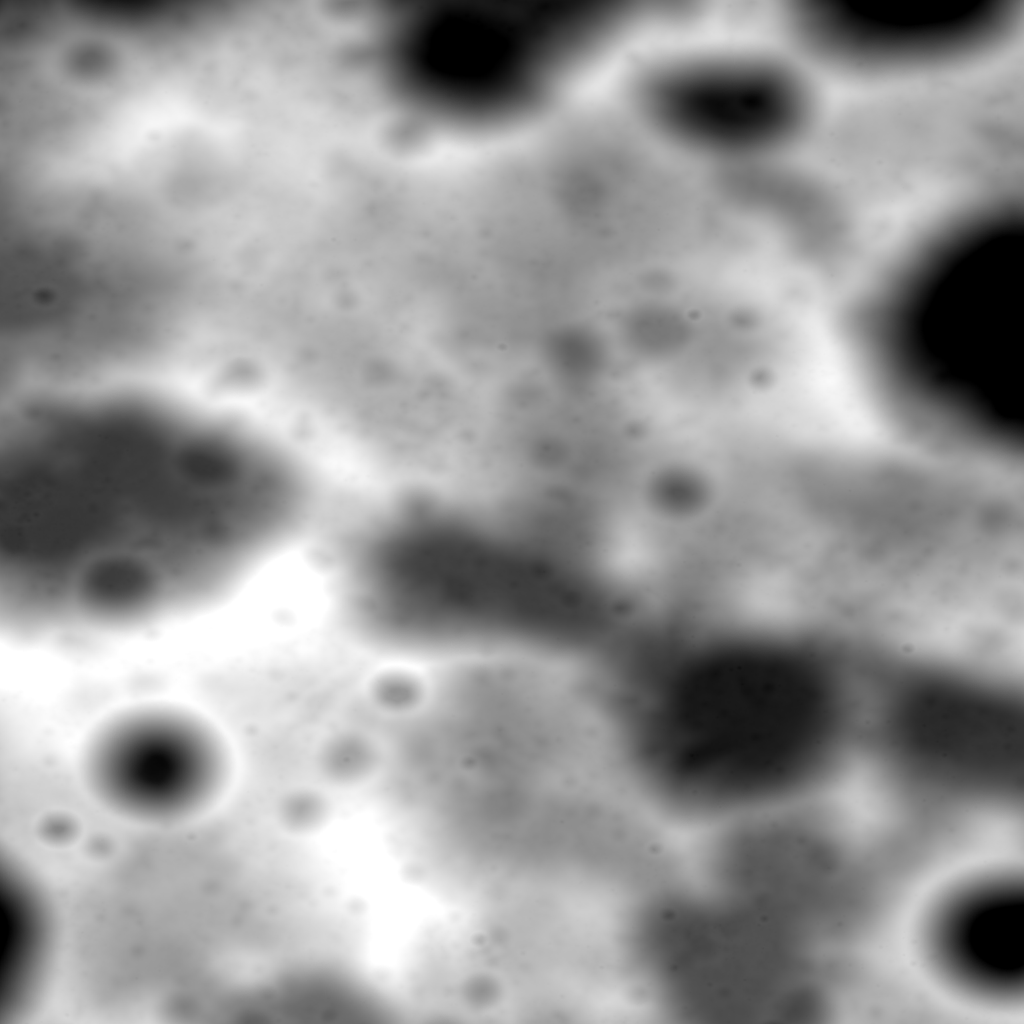

Supplement: Supplementary file 1 [file sensors-26-04344-s001.zip › data/images/test/tile_00945_lon135.0_lat36.0.png]

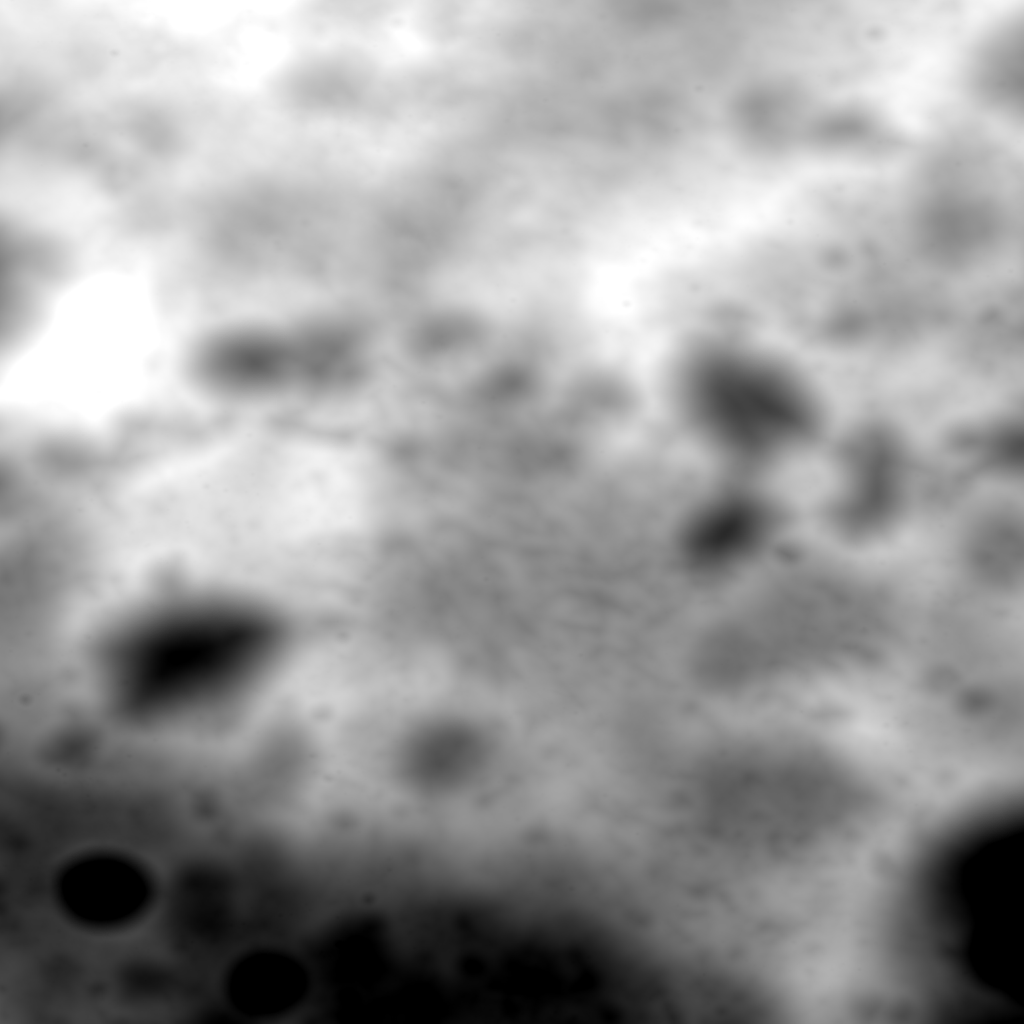

Supplement: Supplementary file 1 [file sensors-26-04344-s001.zip › data/images/test/tile_00951_lon153.0_lat36.0.png]

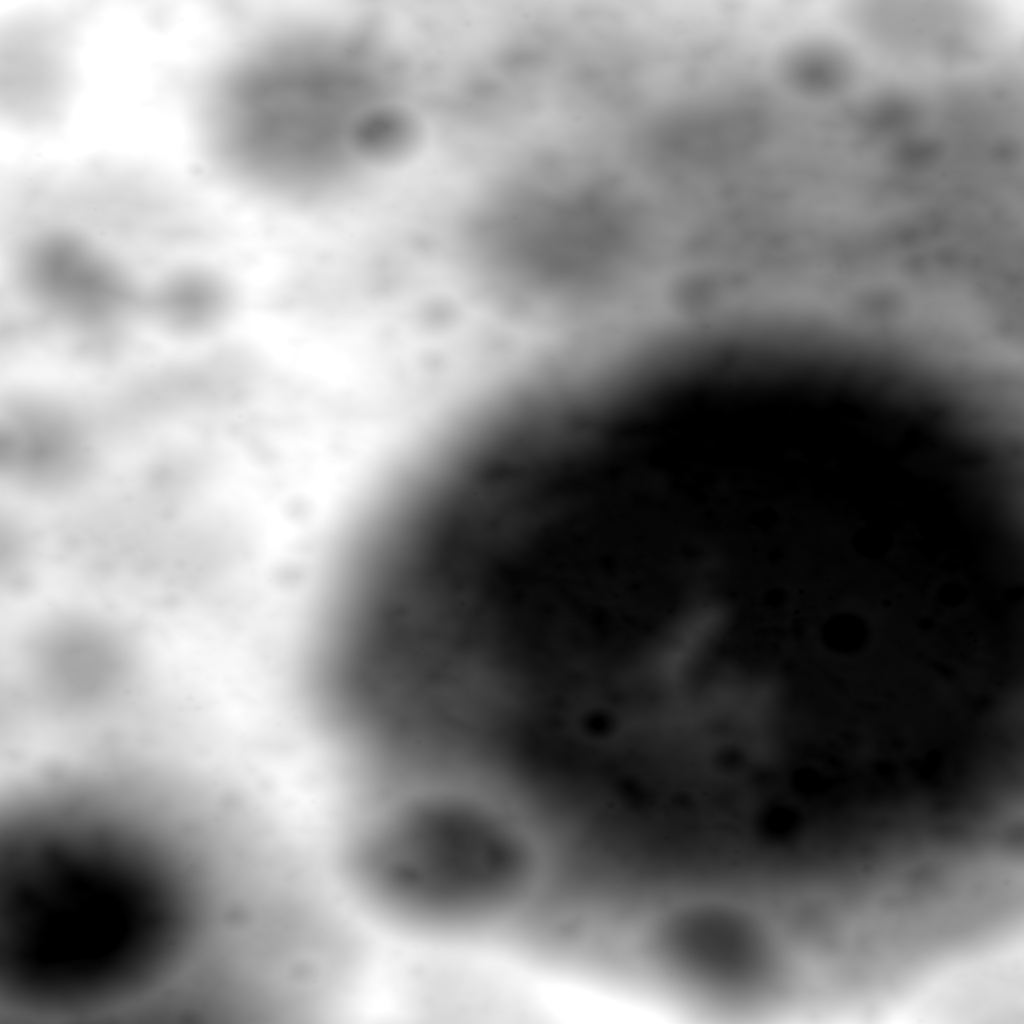

Supplement: Supplementary file 1 [file sensors-26-04344-s001.zip › data/images/test/tile_00952_lon156.0_lat36.0.png]

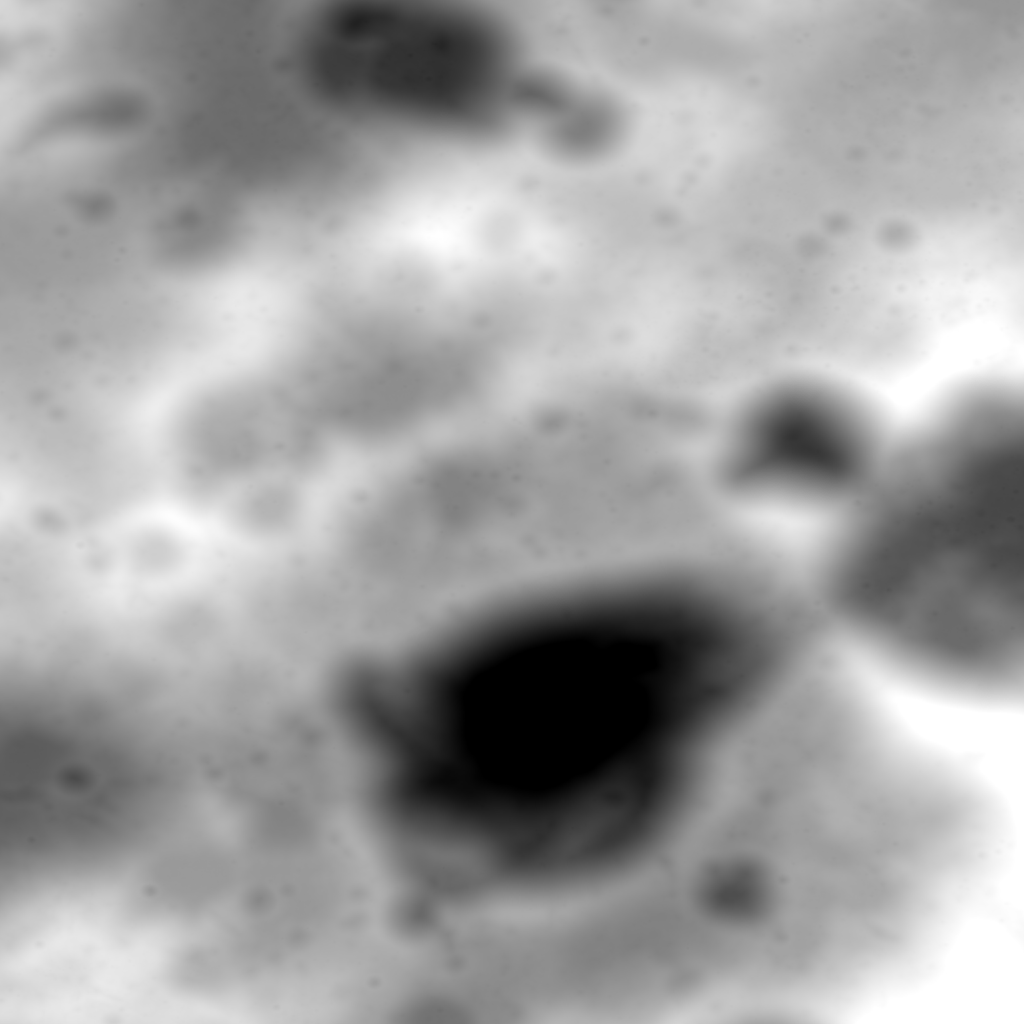

Supplement: Supplementary file 1 [file sensors-26-04344-s001.zip › data/images/test/tile_00954_lon162.0_lat36.0.png]

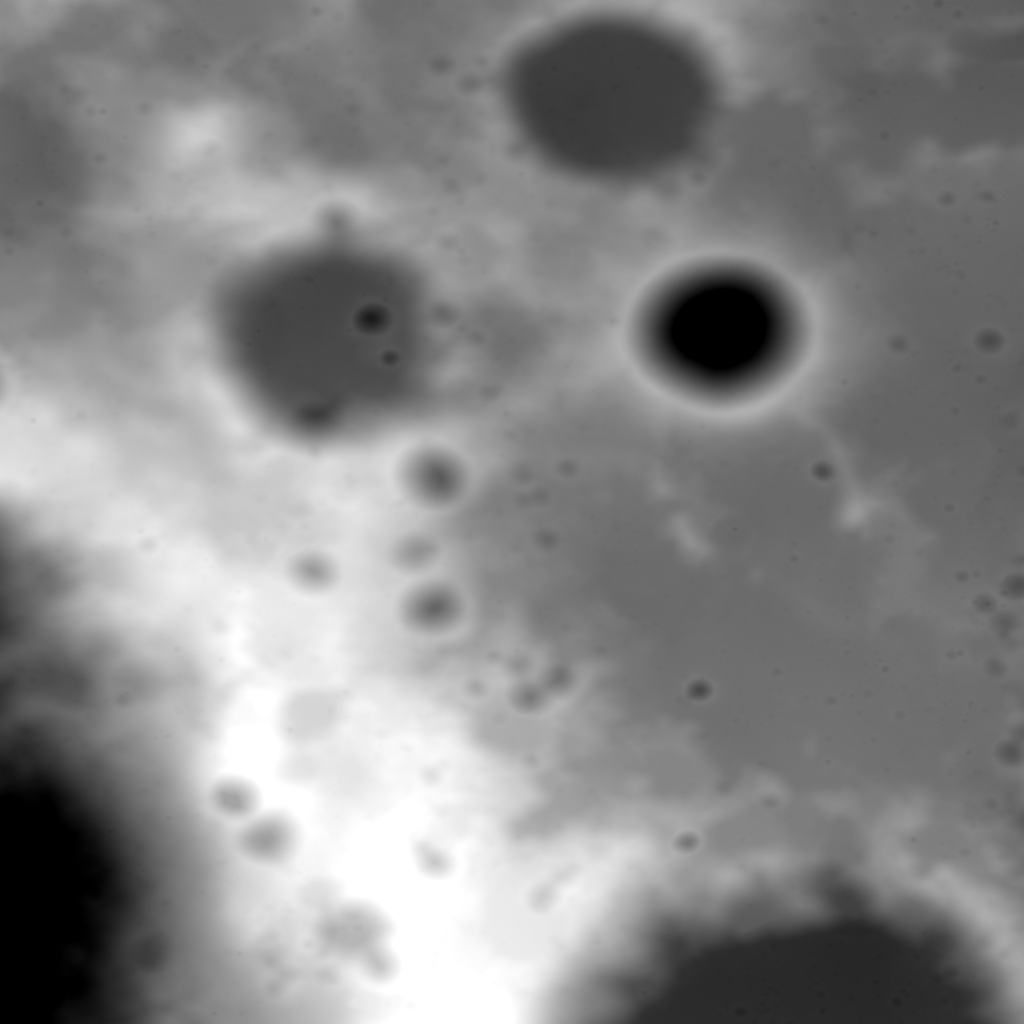

Supplement: Supplementary file 1 [file sensors-26-04344-s001.zip › data/images/test/tile_00990_lon-90.0_lat33.0.png]

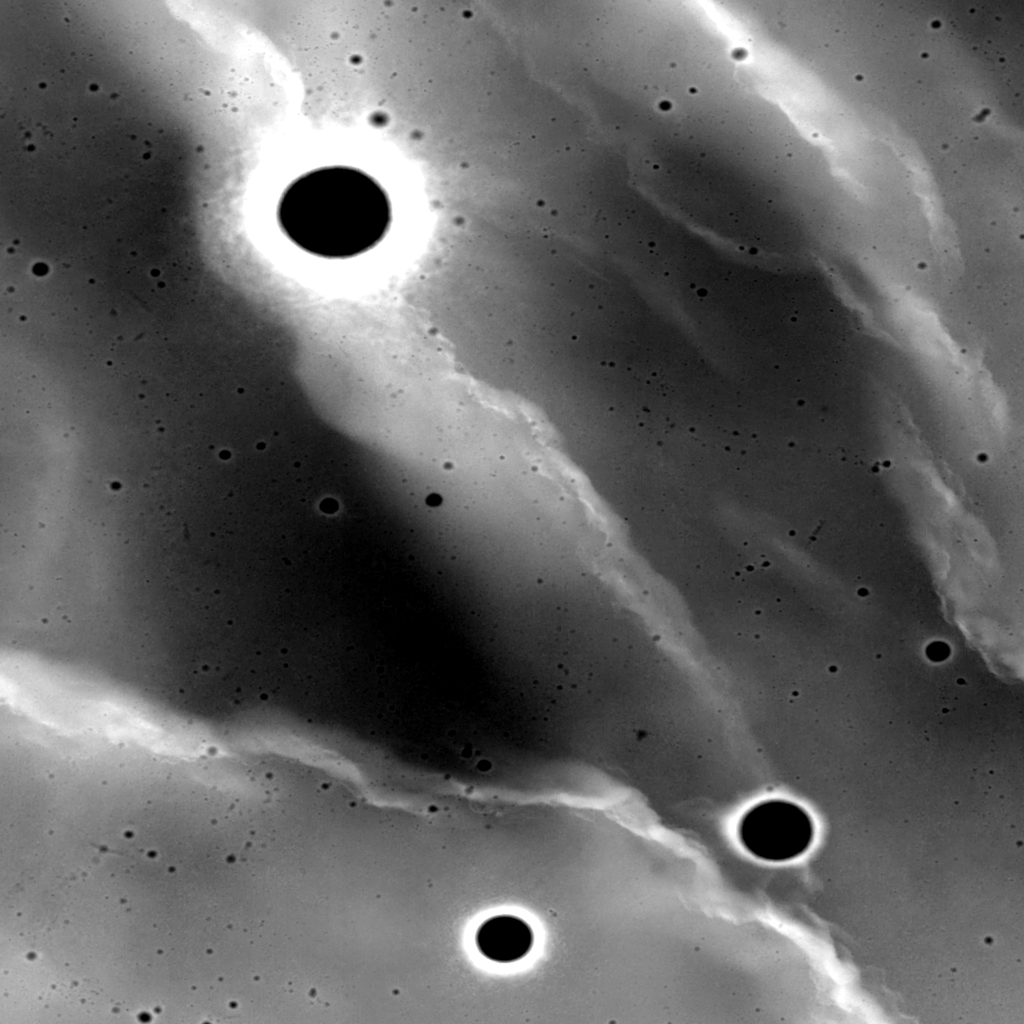

Supplement: Supplementary file 1 [file sensors-26-04344-s001.zip › data/images/test/tile_00999_lon-63.0_lat33.0.png]

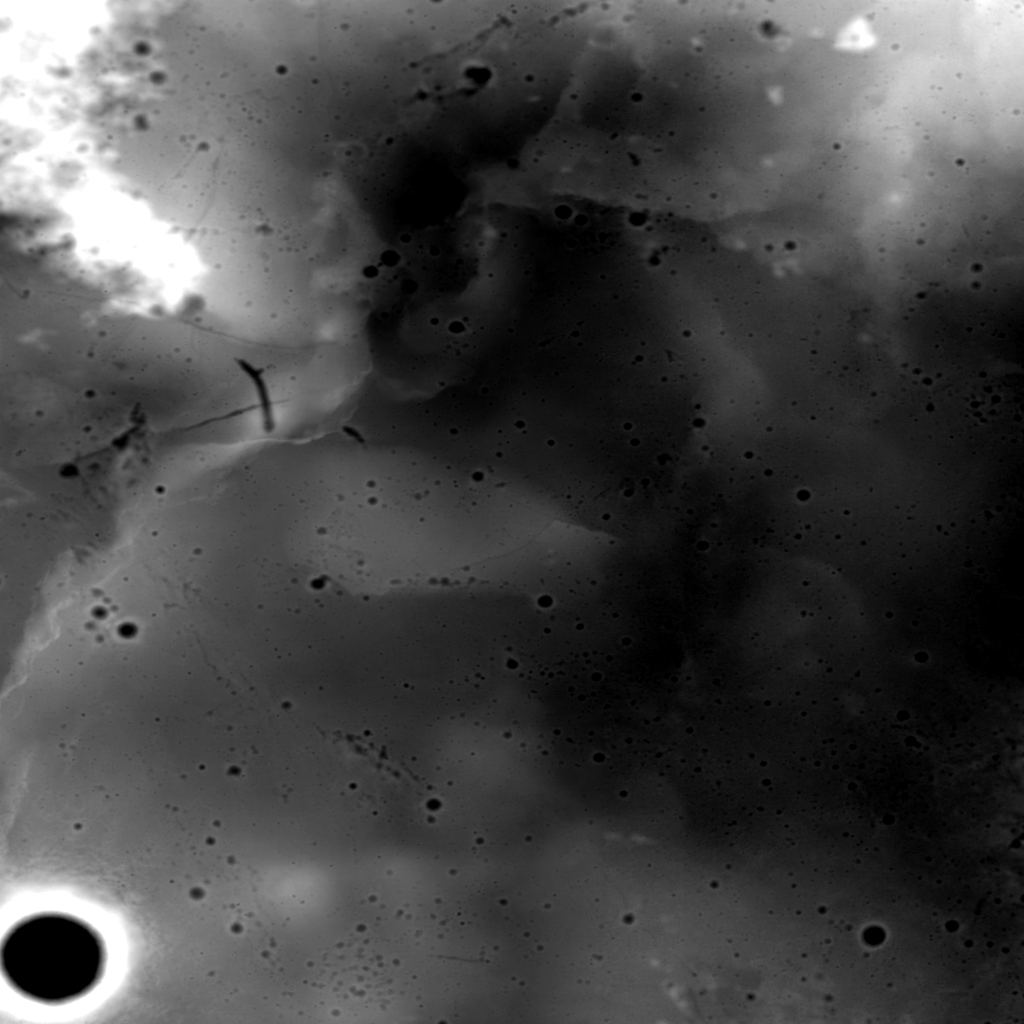

Supplement: Supplementary file 1 [file sensors-26-04344-s001.zip › data/images/test/tile_01028_lon24.0_lat33.0.png]

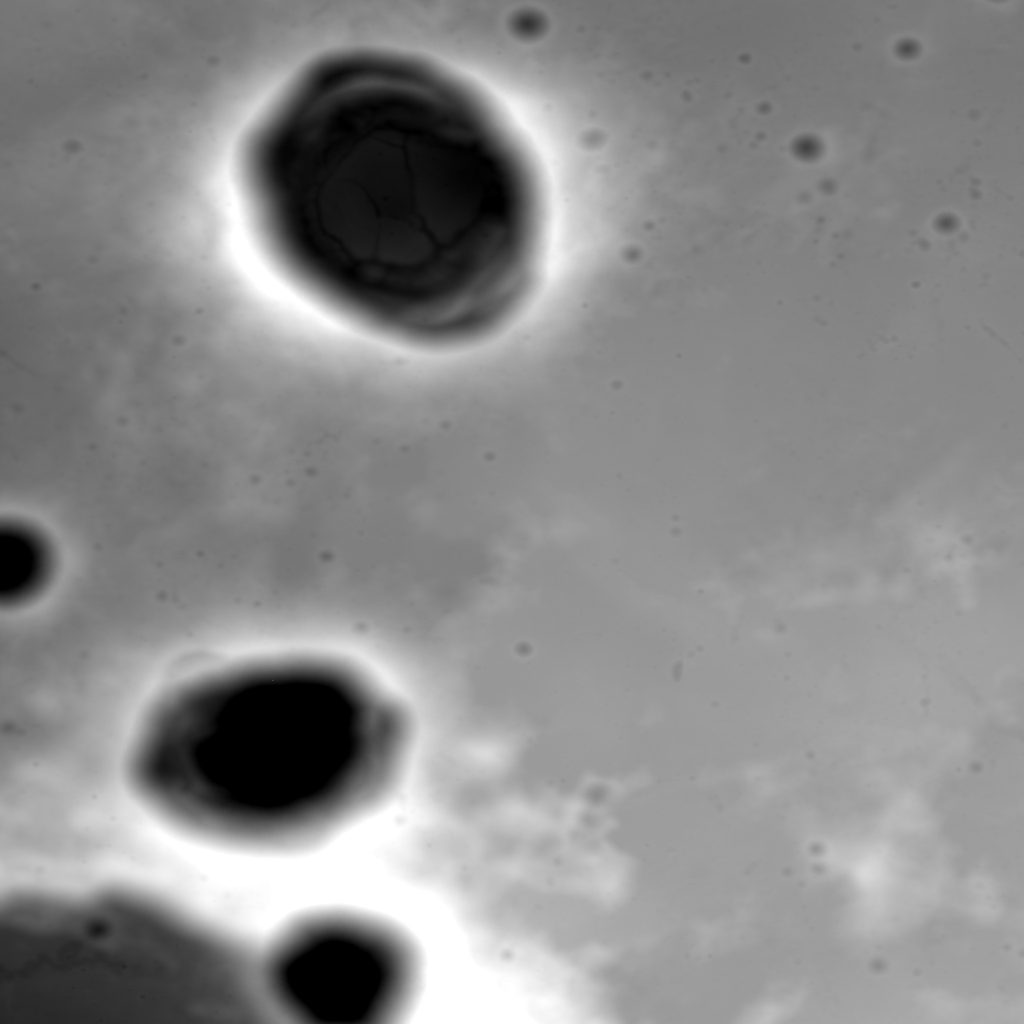

Supplement: Supplementary file 1 [file sensors-26-04344-s001.zip › data/images/test/tile_01030_lon30.0_lat33.0.png]

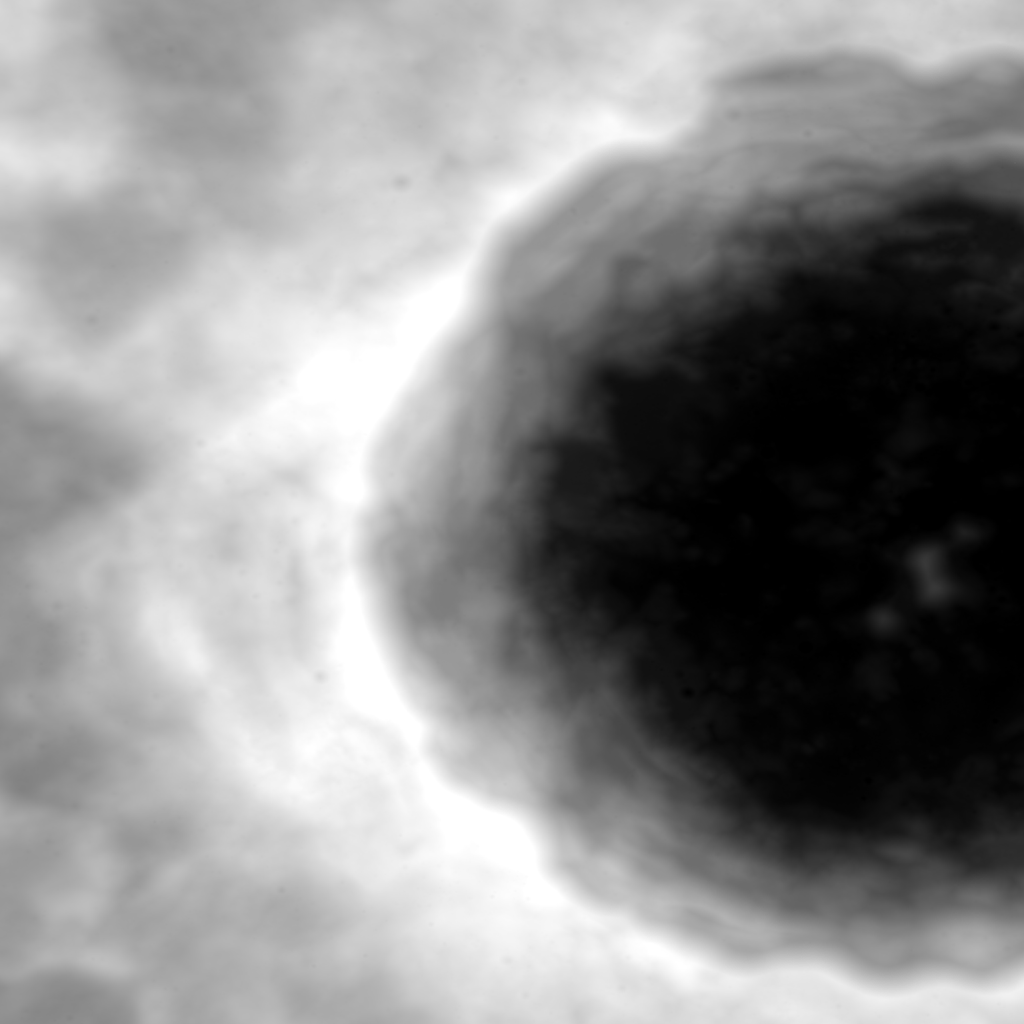

Supplement: Supplementary file 1 [file sensors-26-04344-s001.zip › data/images/test/tile_01038_lon54.0_lat33.0.png]

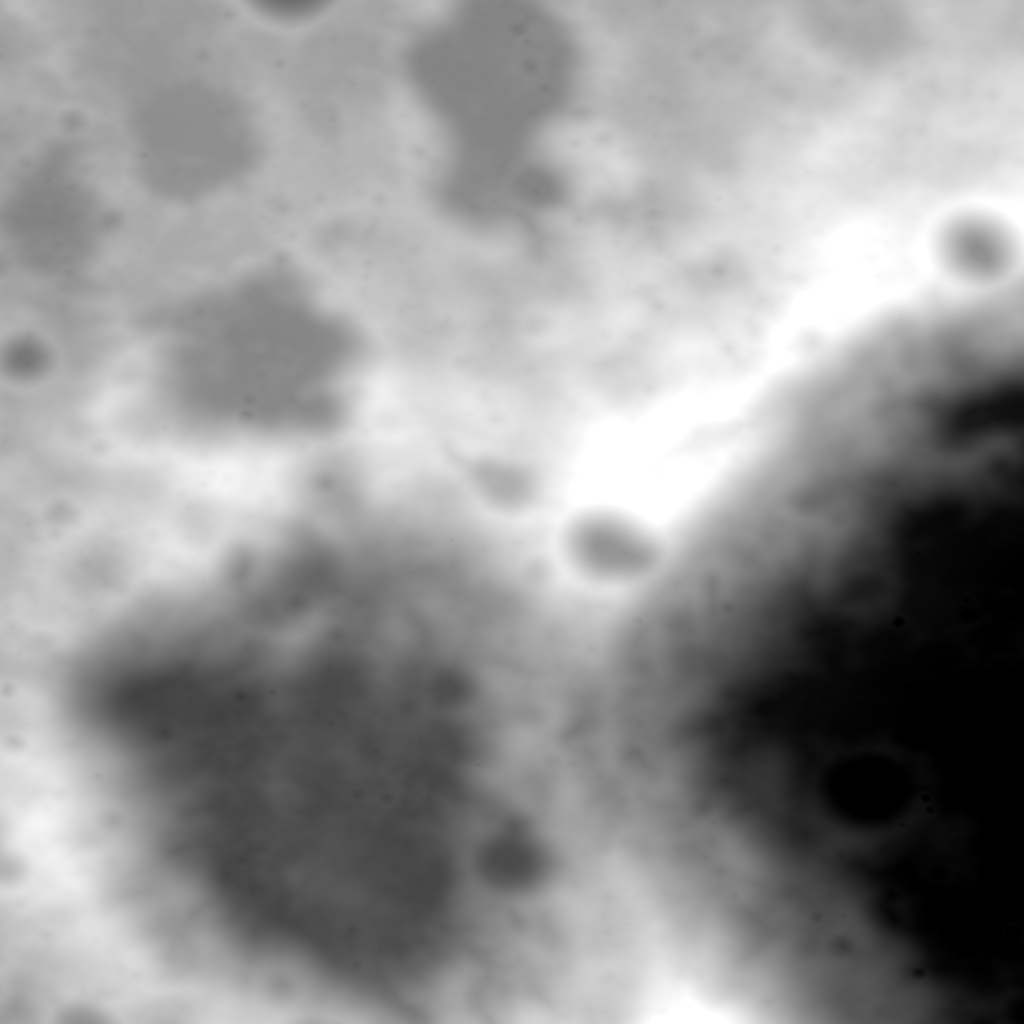

Supplement: Supplementary file 1 [file sensors-26-04344-s001.zip › data/images/test/tile_01050_lon90.0_lat33.0.png]

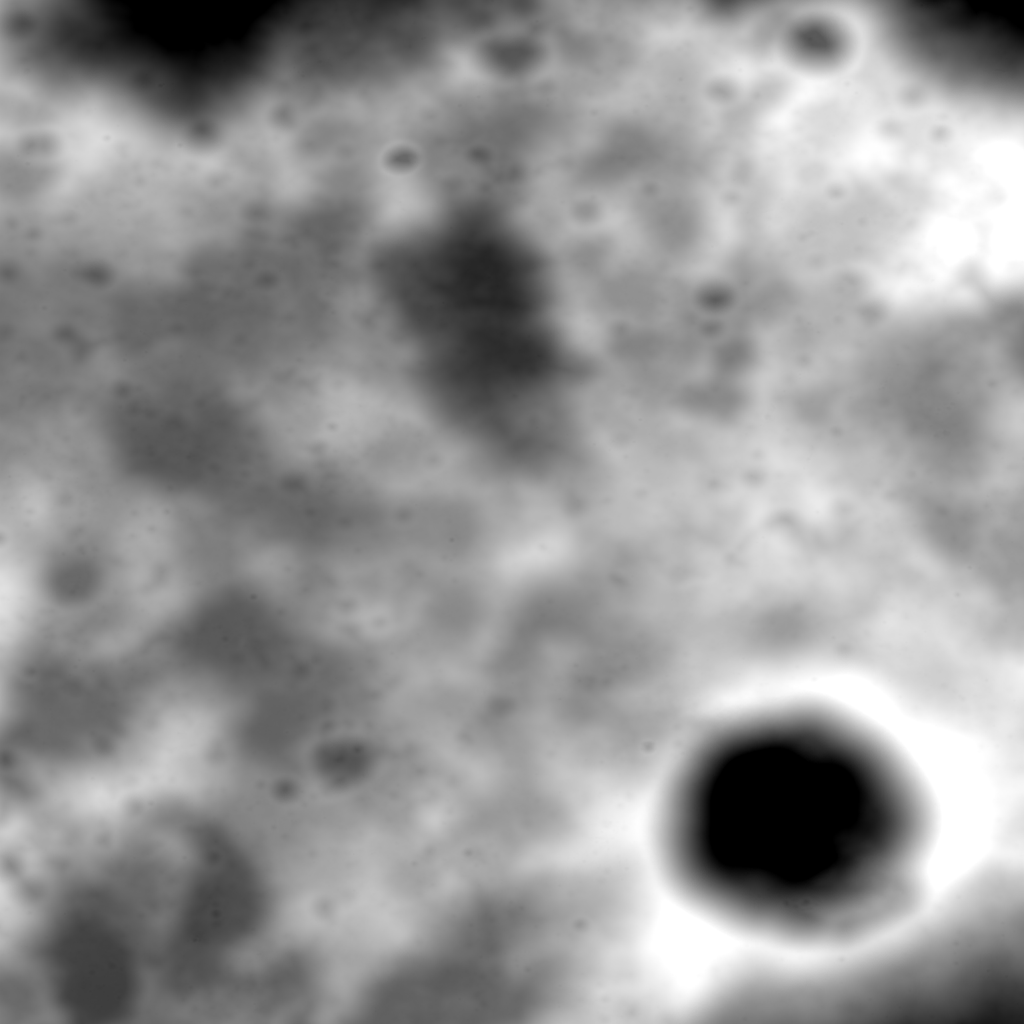

Supplement: Supplementary file 1 [file sensors-26-04344-s001.zip › data/images/test/tile_01052_lon96.0_lat33.0.png]

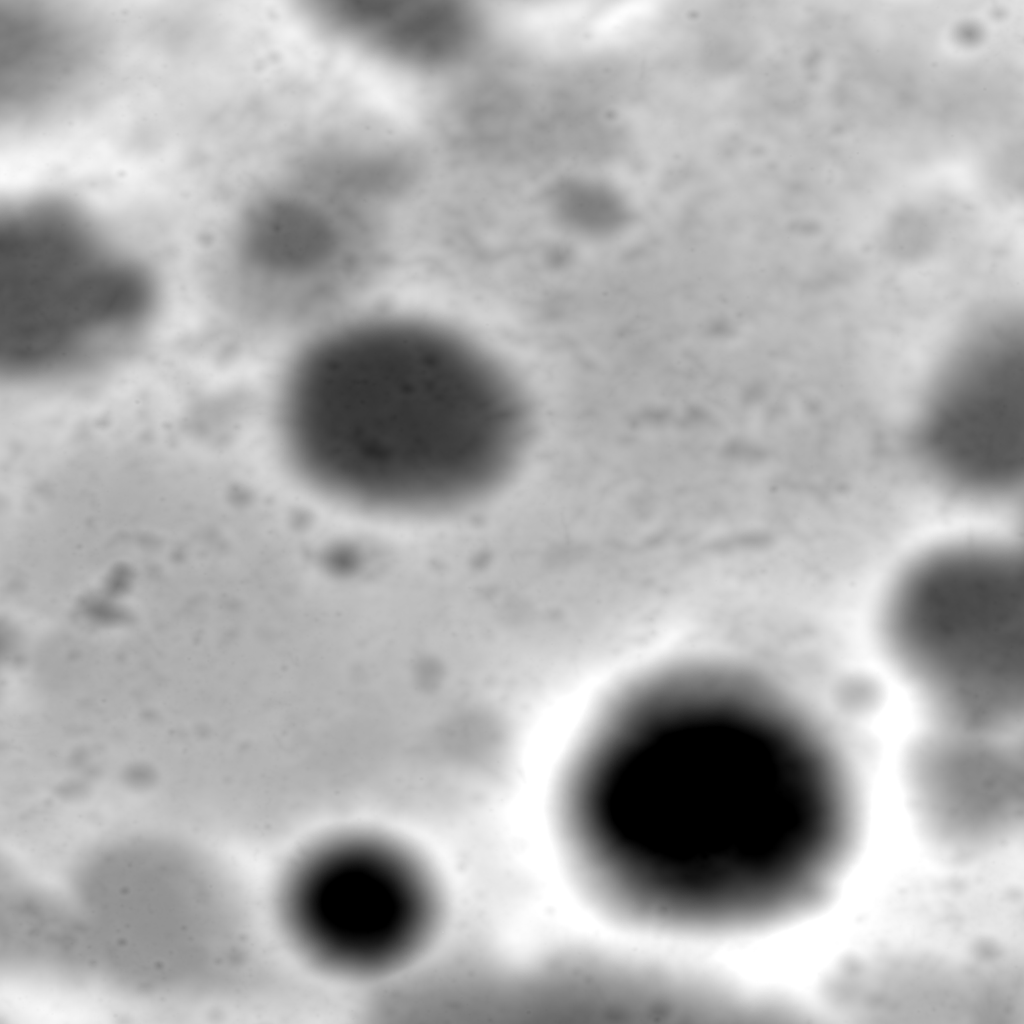

Supplement: Supplementary file 1 [file sensors-26-04344-s001.zip › data/images/test/tile_01059_lon117.0_lat33.0.png]

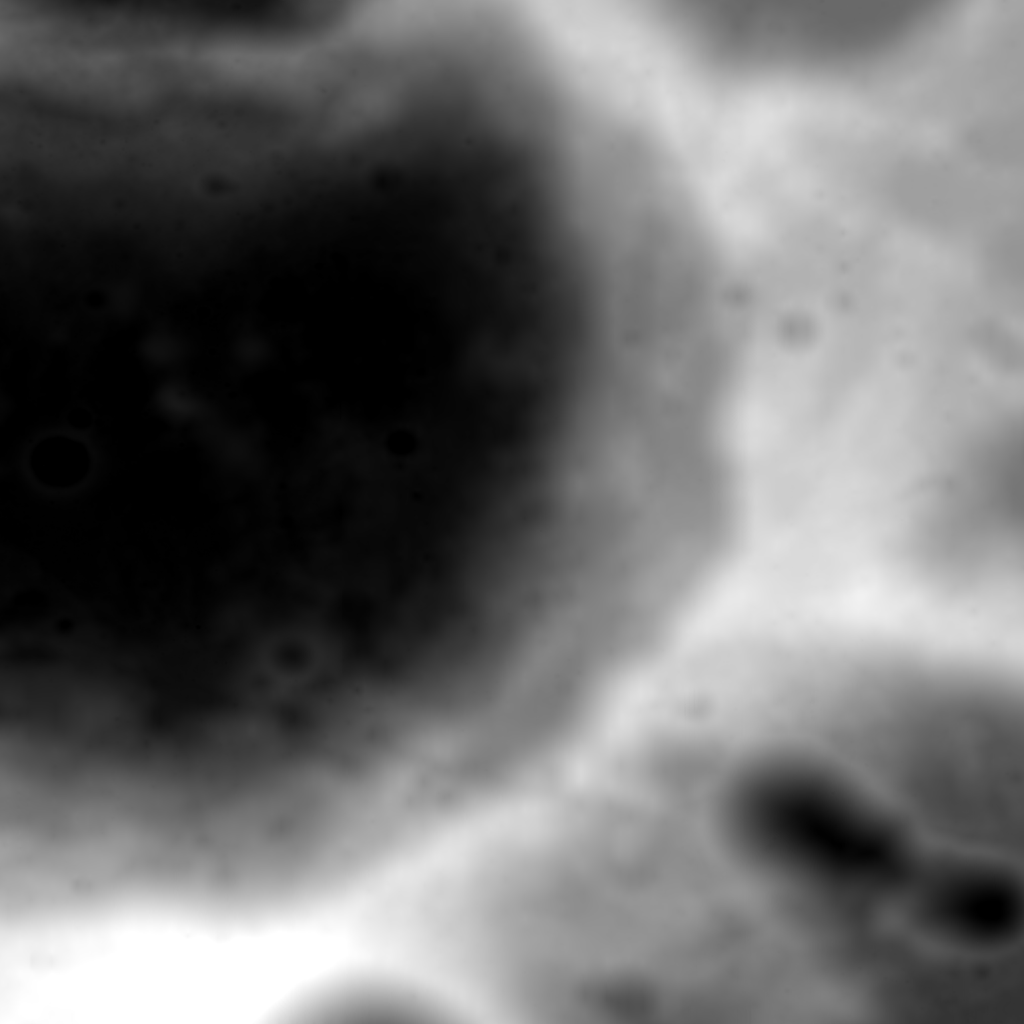

Supplement: Supplementary file 1 [file sensors-26-04344-s001.zip › data/images/test/tile_01080_lon-180.0_lat30.0.png]

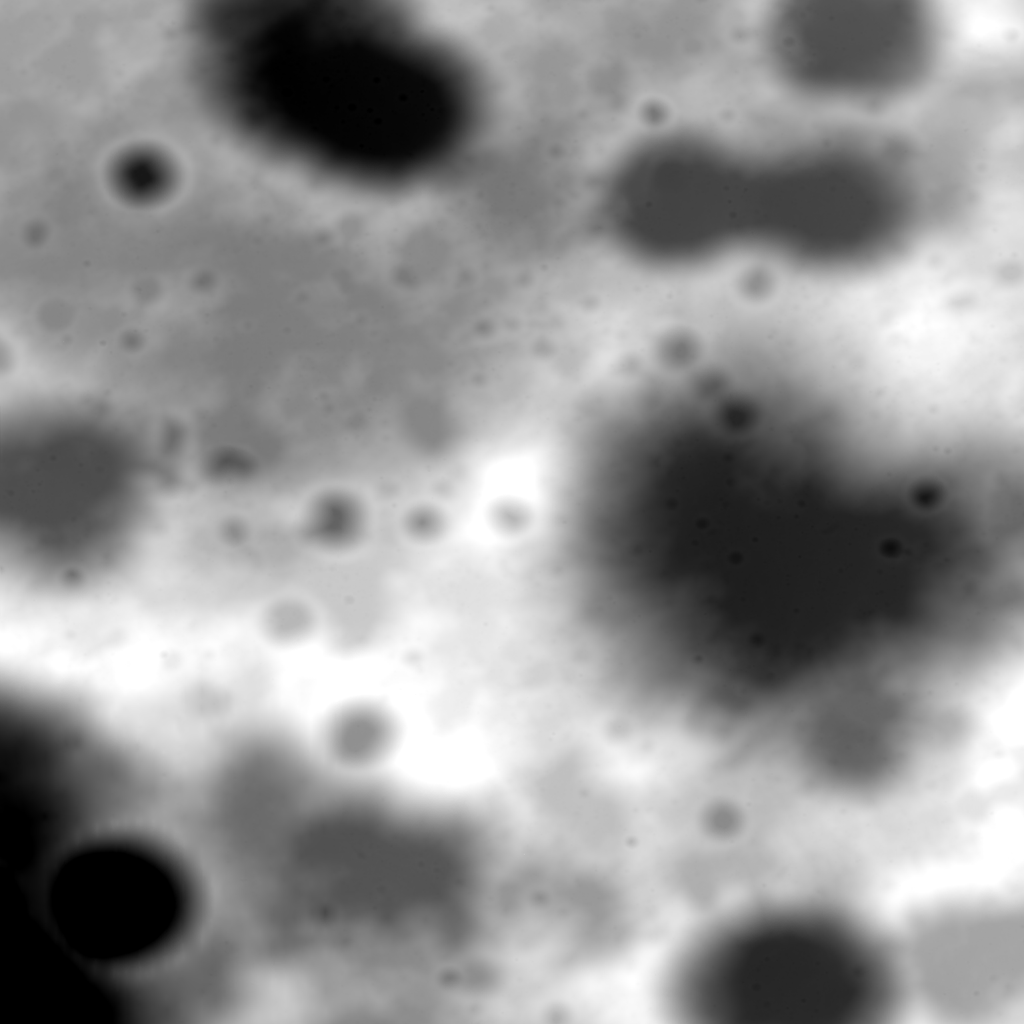

Supplement: Supplementary file 1 [file sensors-26-04344-s001.zip › data/images/test/tile_01081_lon-177.0_lat30.0.png]

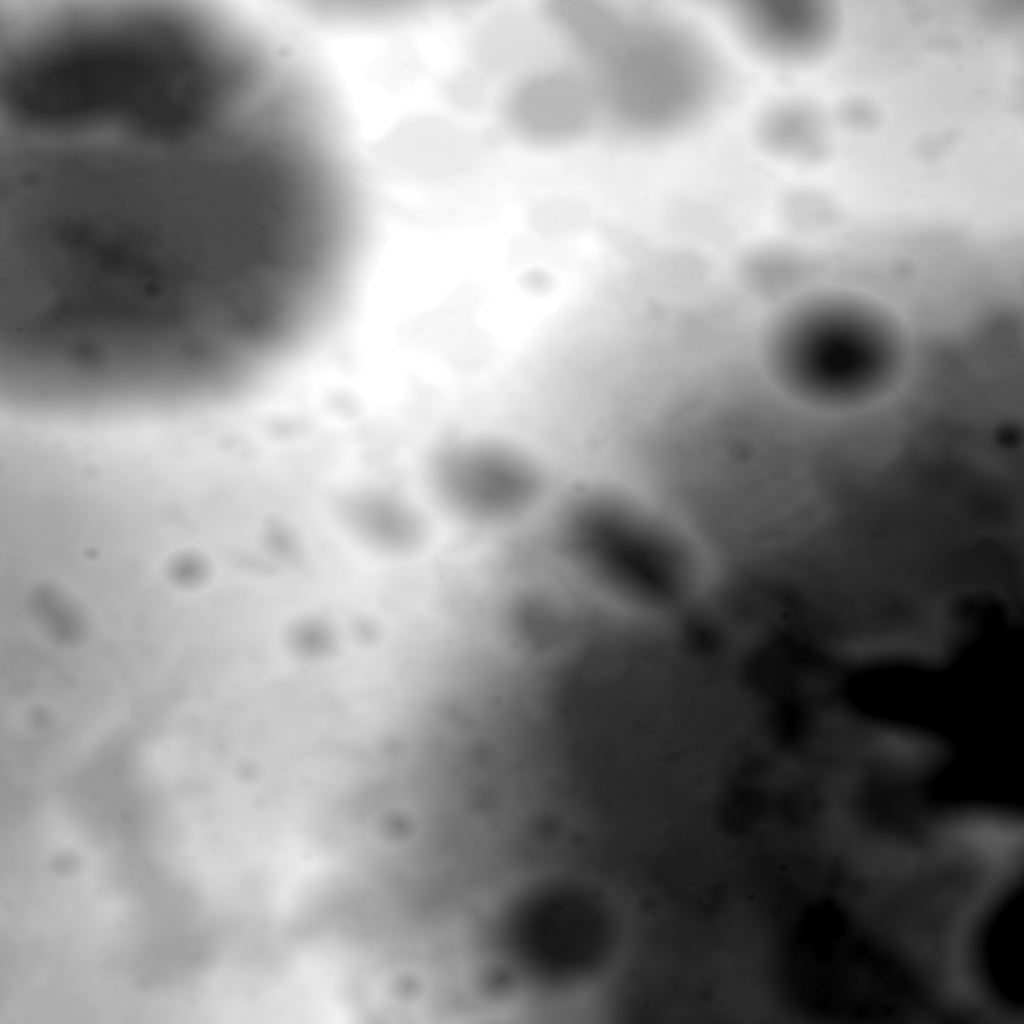

Supplement: Supplementary file 1 [file sensors-26-04344-s001.zip › data/images/test/tile_01092_lon-144.0_lat30.0.png]

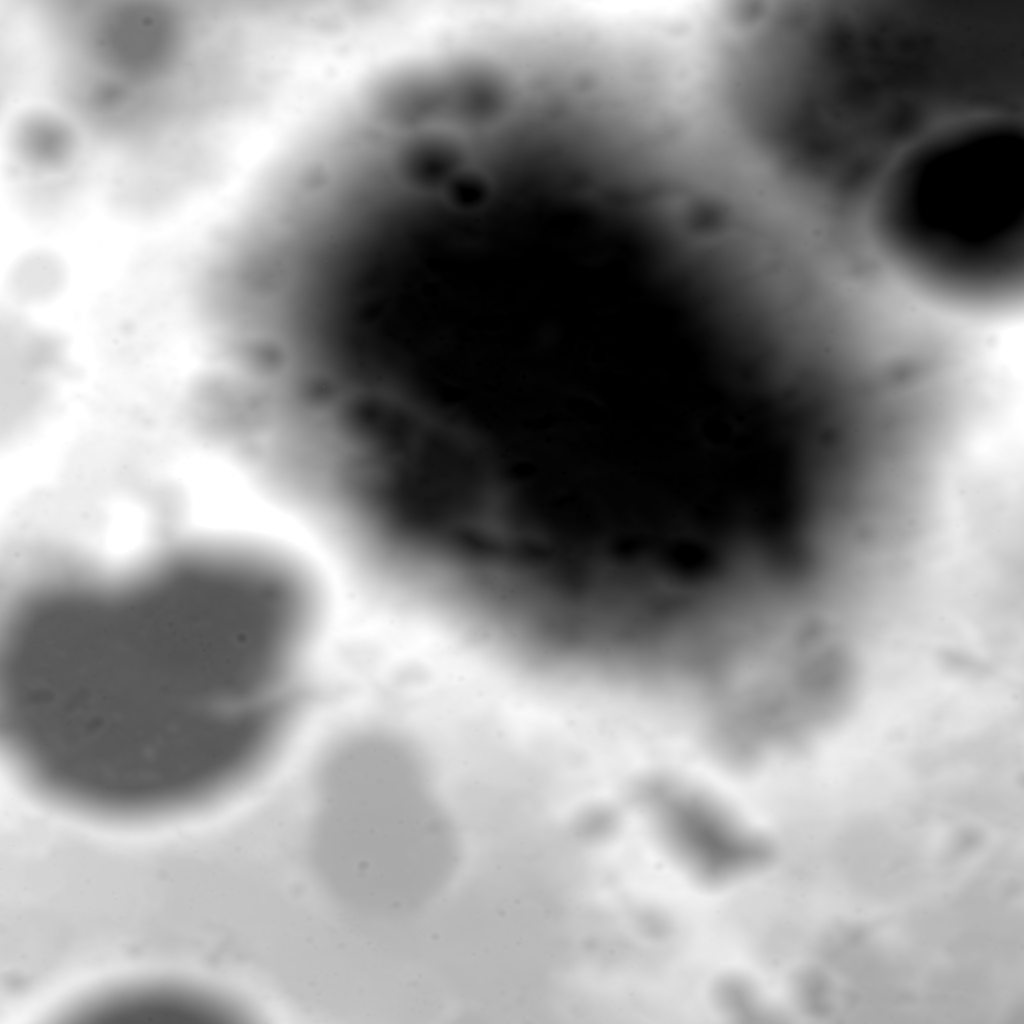

Supplement: Supplementary file 1 [file sensors-26-04344-s001.zip › data/images/test/tile_01094_lon-138.0_lat30.0.png]

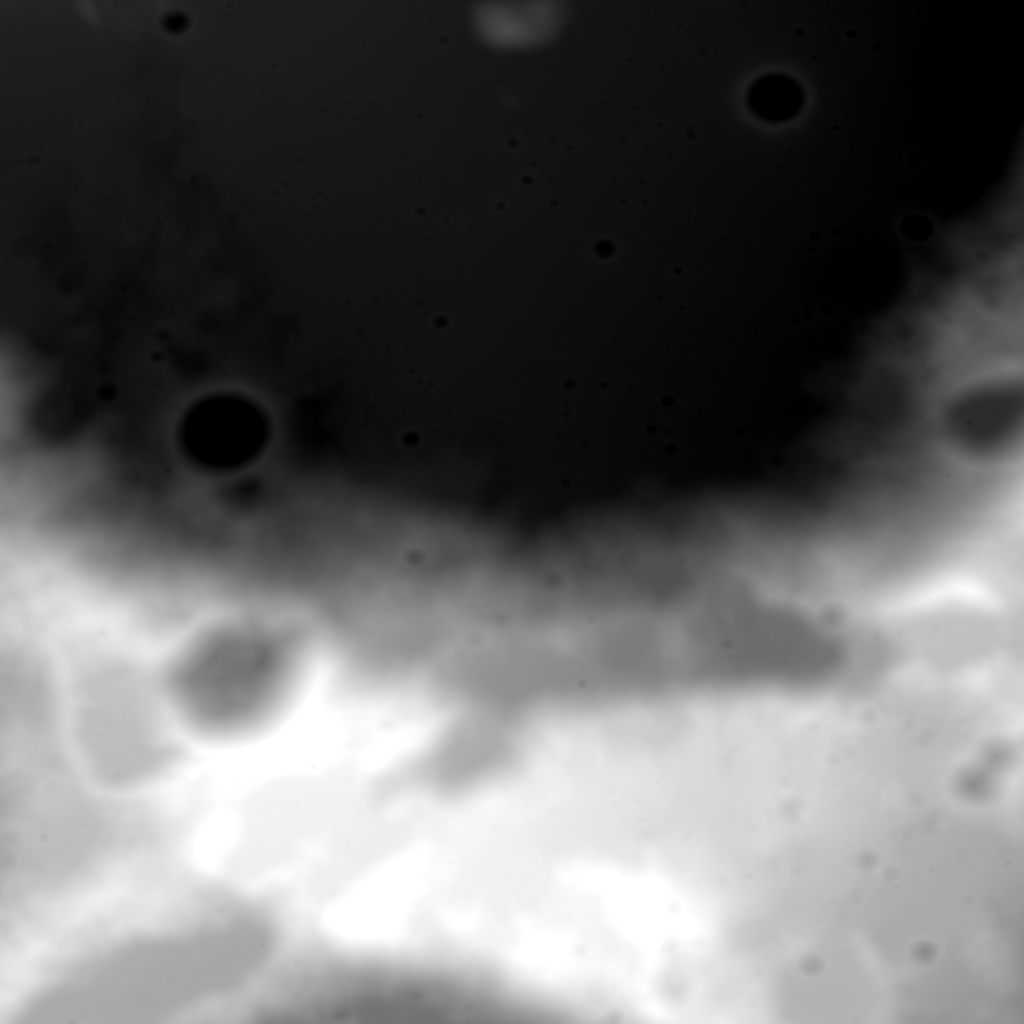

Supplement: Supplementary file 1 [file sensors-26-04344-s001.zip › data/images/test/tile_01109_lon-93.0_lat30.0.png]

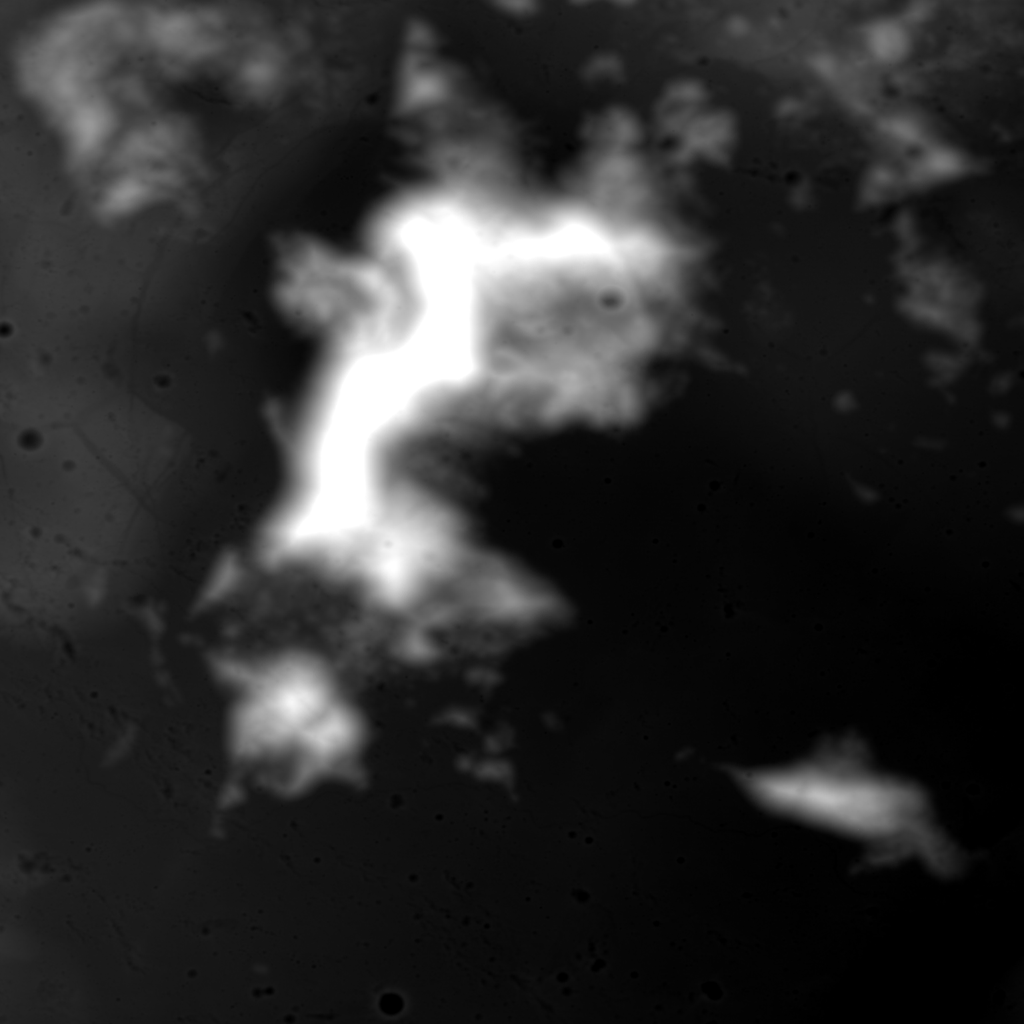

Supplement: Supplementary file 1 [file sensors-26-04344-s001.zip › data/images/test/tile_01142_lon6.0_lat30.0.png]

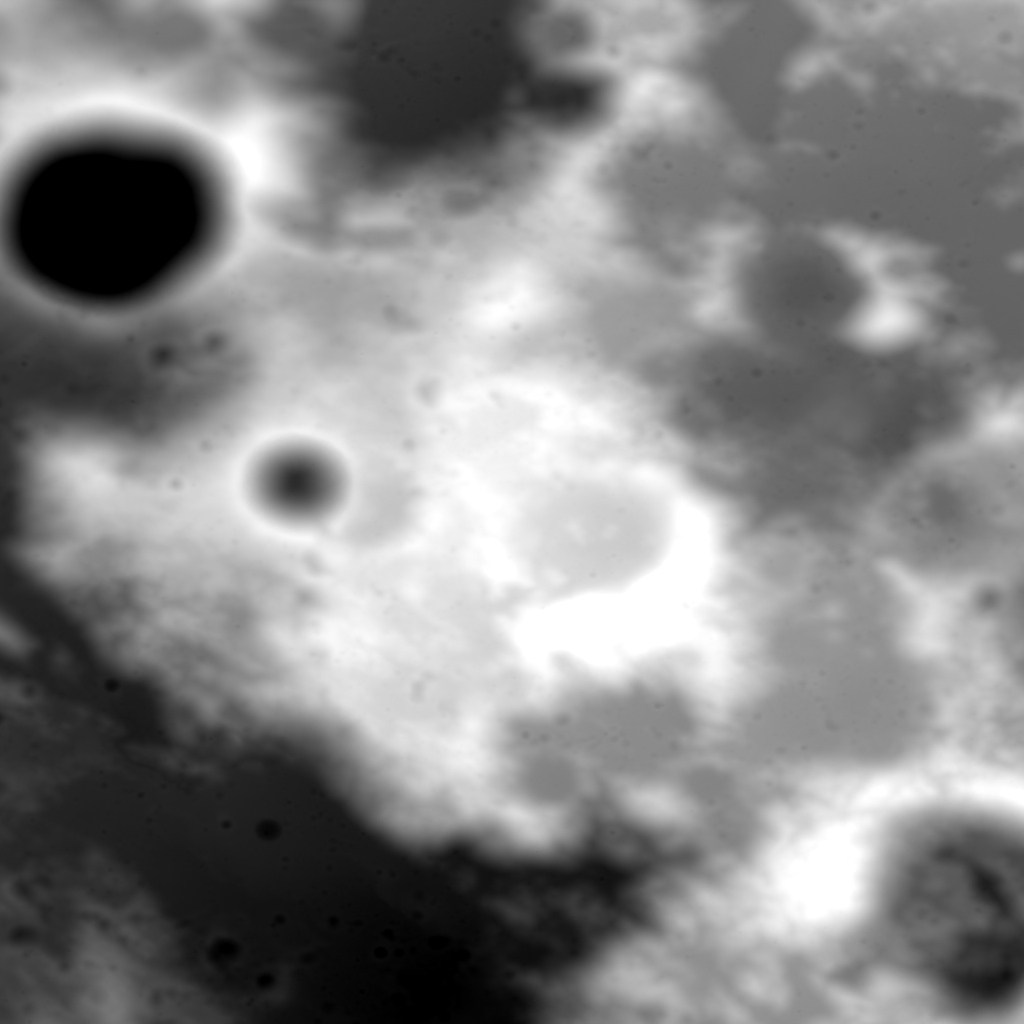

Supplement: Supplementary file 1 [file sensors-26-04344-s001.zip › data/images/test/tile_01152_lon36.0_lat30.0.png]

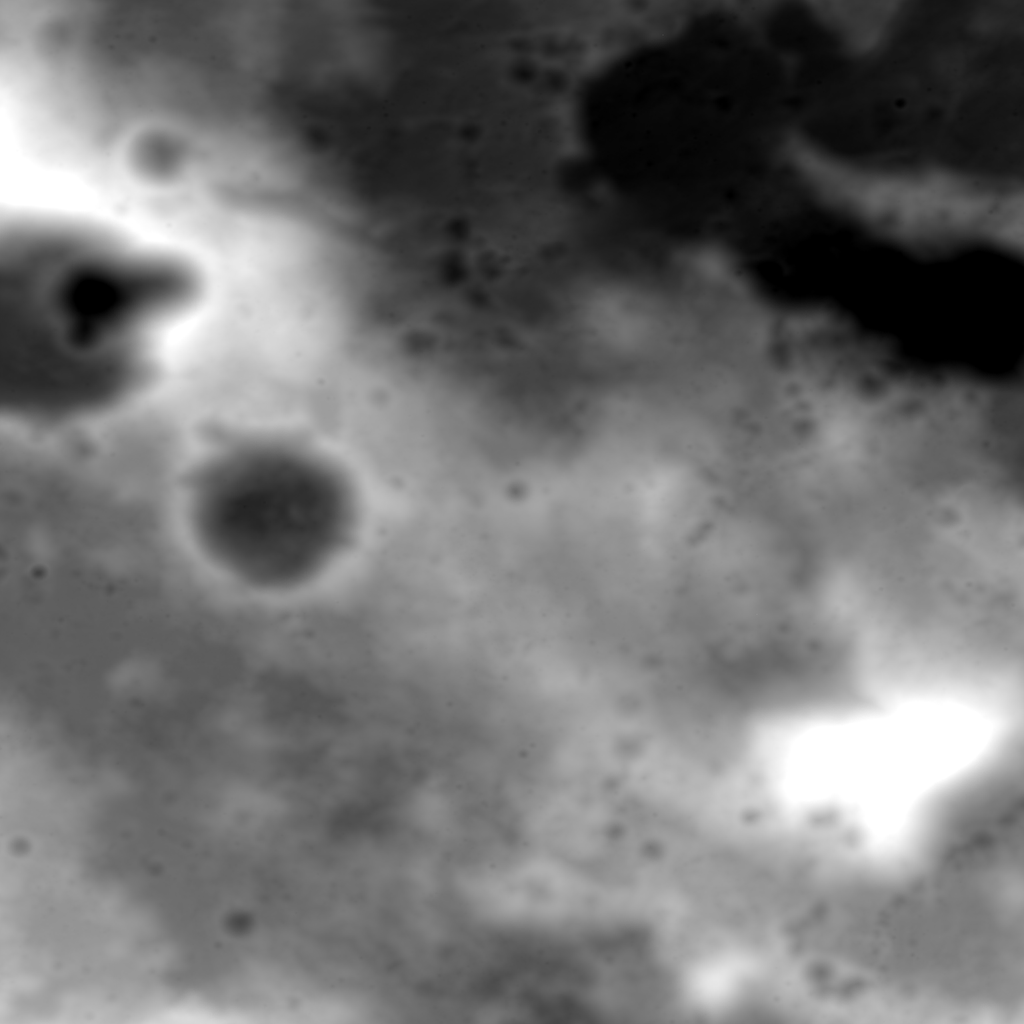

Supplement: Supplementary file 1 [file sensors-26-04344-s001.zip › data/images/test/tile_01157_lon51.0_lat30.0.png]

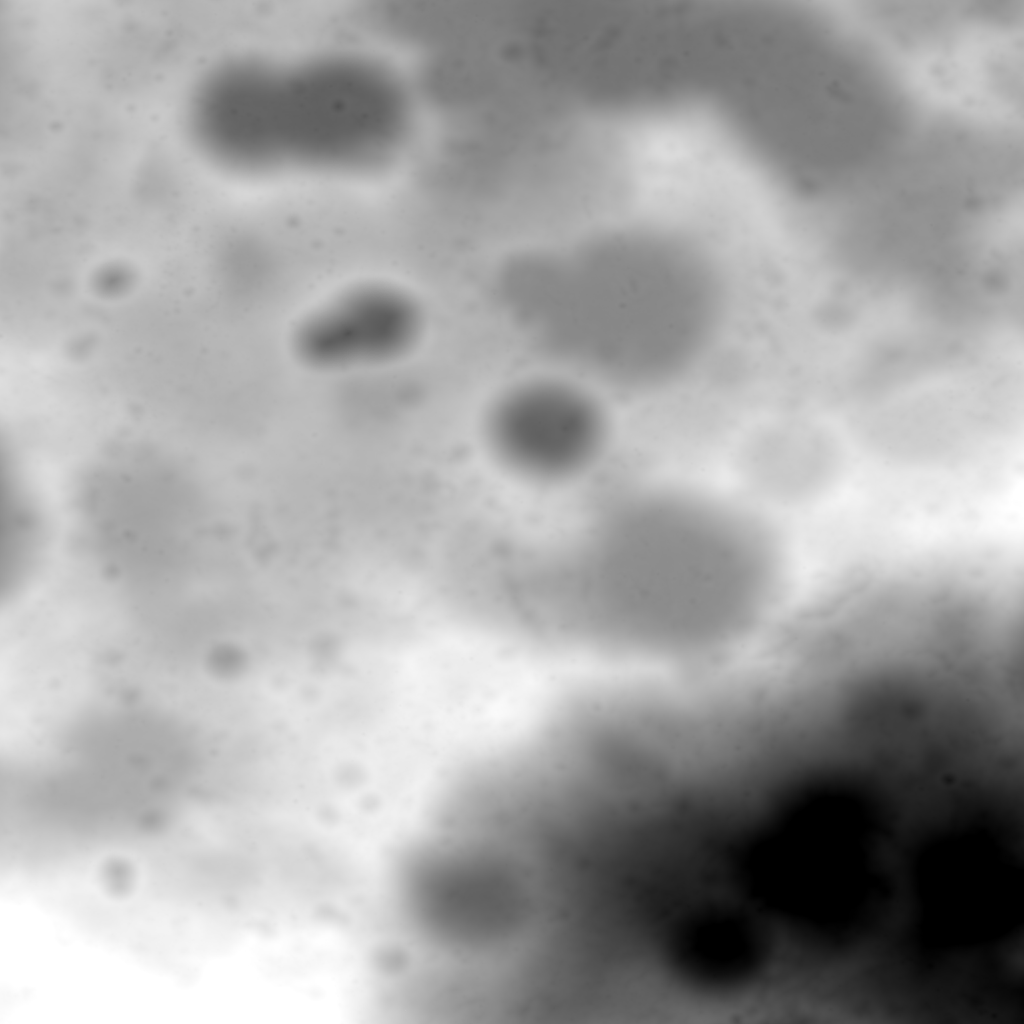

Supplement: Supplementary file 1 [file sensors-26-04344-s001.zip › data/images/test/tile_01179_lon117.0_lat30.0.png]

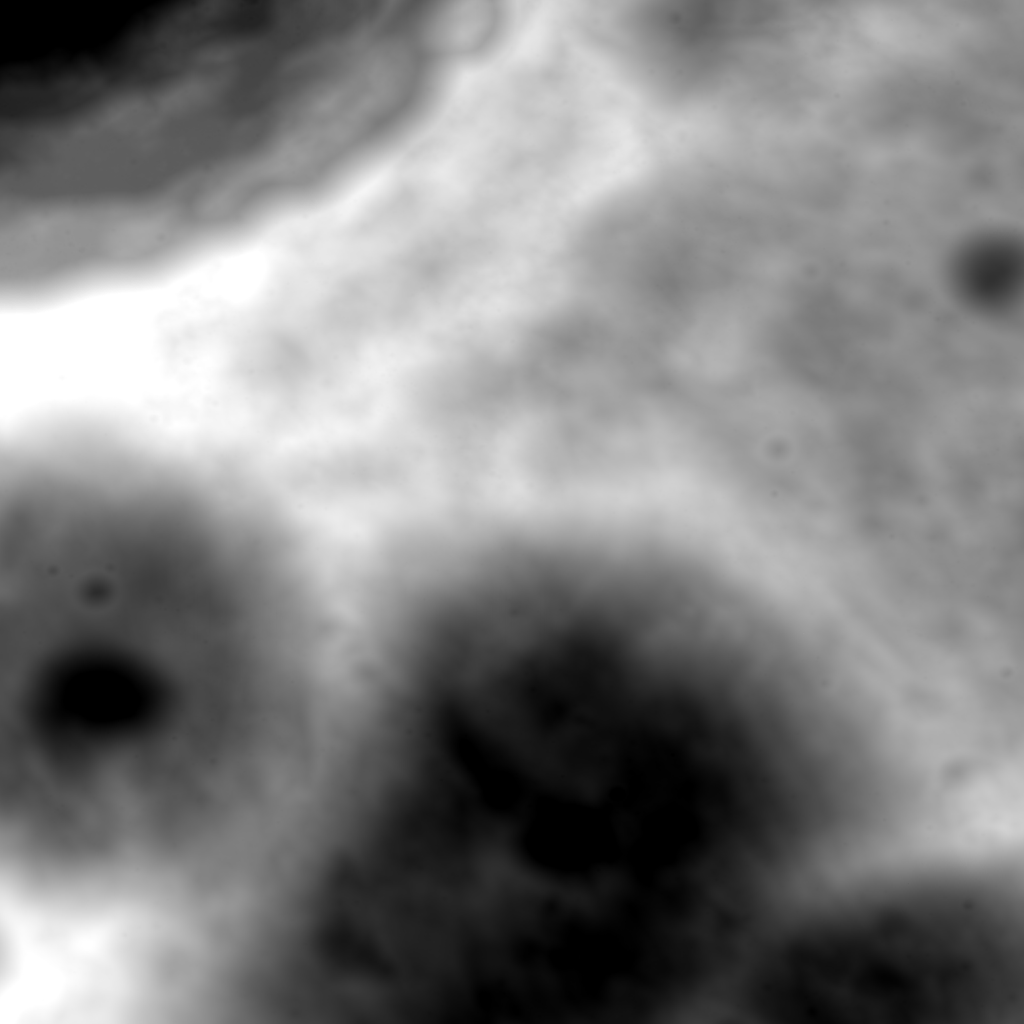

Supplement: Supplementary file 1 [file sensors-26-04344-s001.zip › data/images/test/tile_01217_lon-129.0_lat27.0.png]

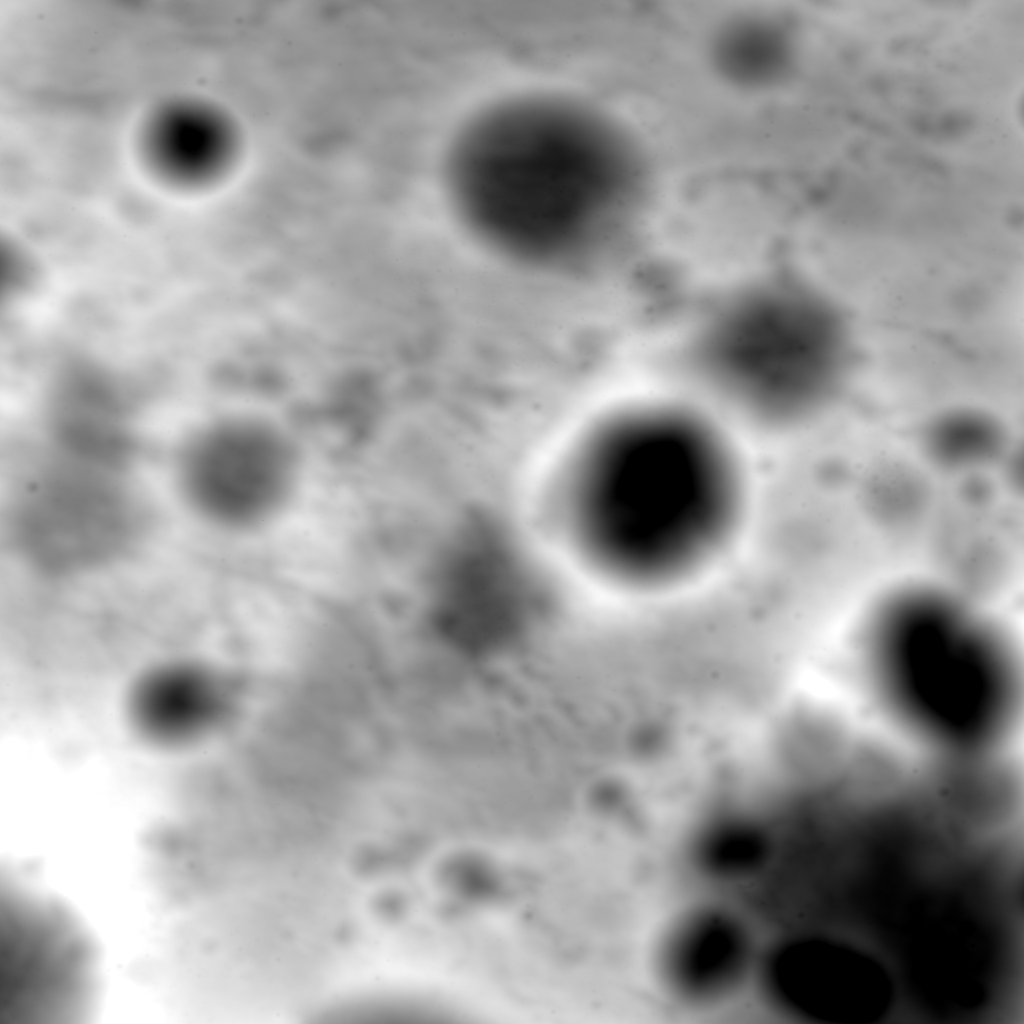

Supplement: Supplementary file 1 [file sensors-26-04344-s001.zip › data/images/test/tile_01218_lon-126.0_lat27.0.png]

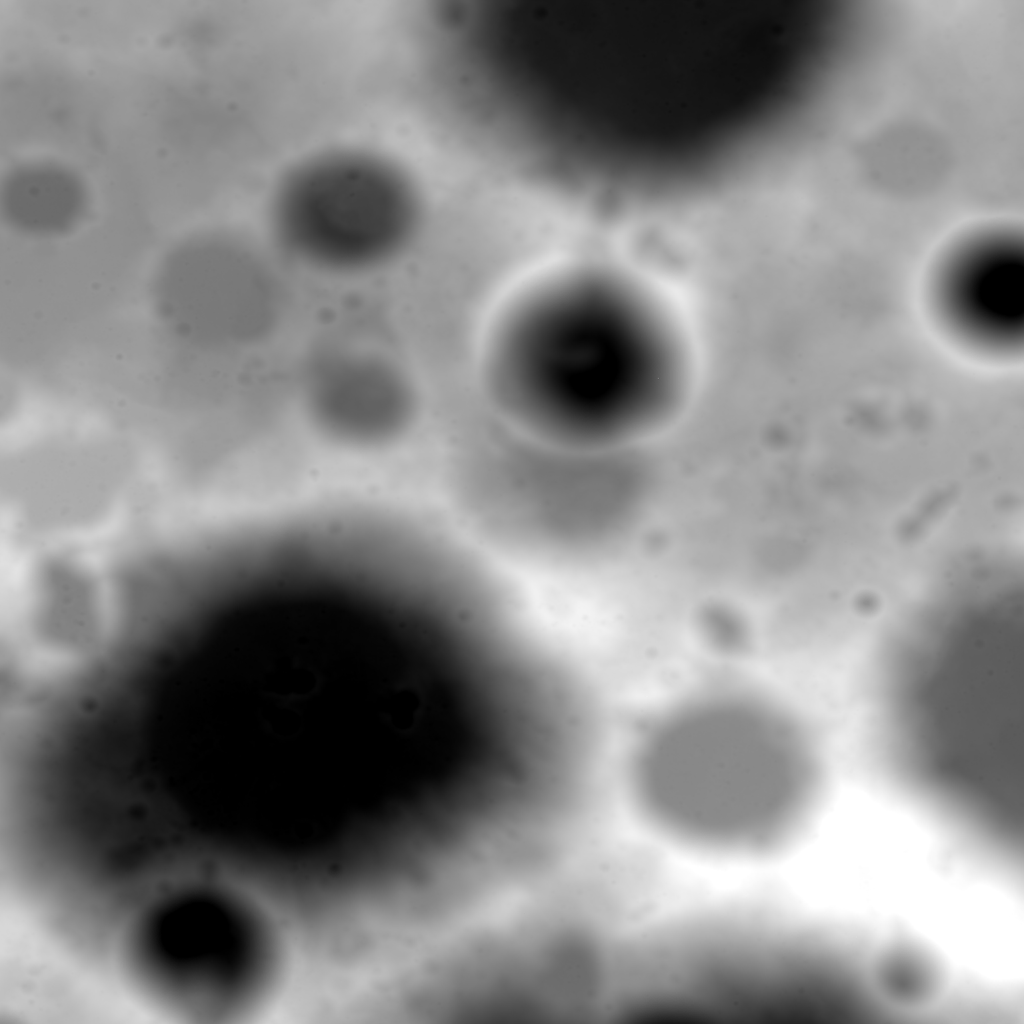

Supplement: Supplementary file 1 [file sensors-26-04344-s001.zip › data/images/test/tile_01223_lon-111.0_lat27.0.png]

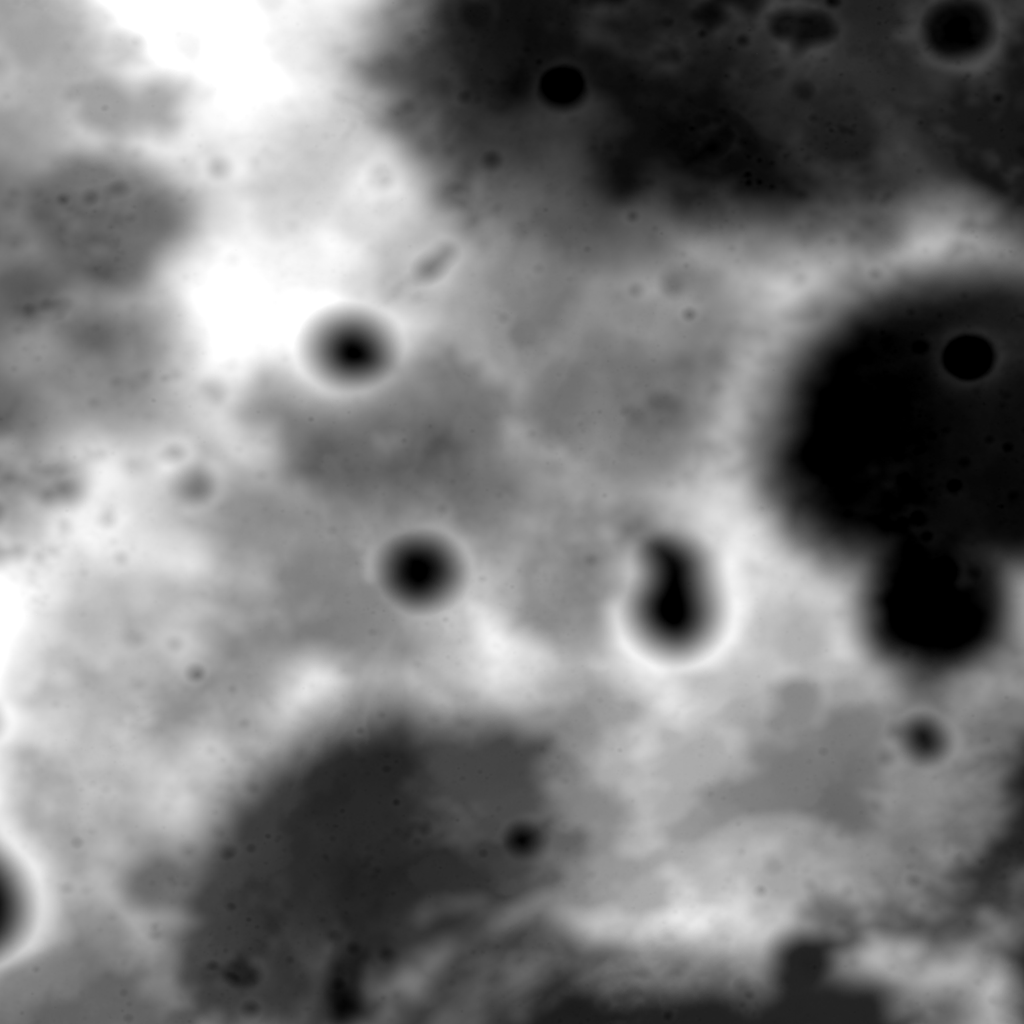

Supplement: Supplementary file 1 [file sensors-26-04344-s001.zip › data/images/test/tile_01231_lon-87.0_lat27.0.png]

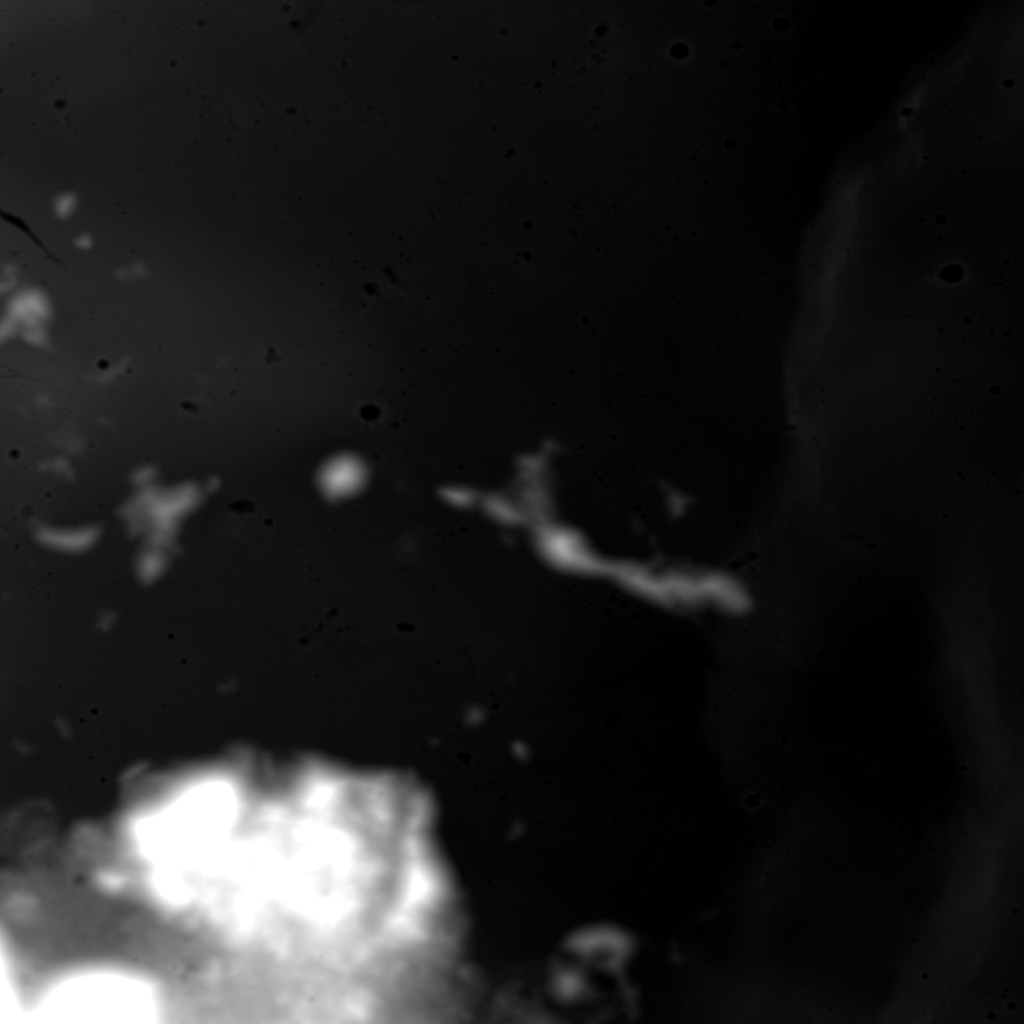

Supplement: Supplementary file 1 [file sensors-26-04344-s001.zip › data/images/test/tile_01262_lon6.0_lat27.0.png]

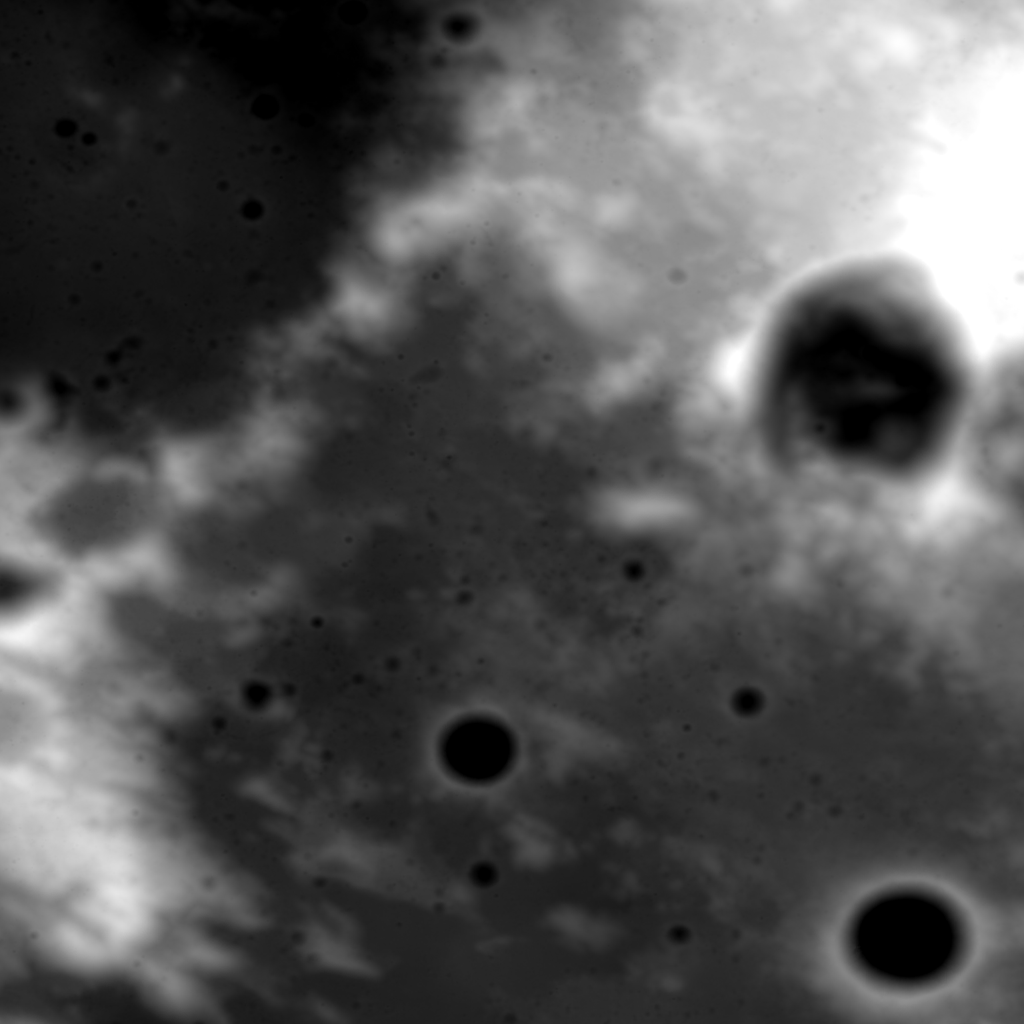

Supplement: Supplementary file 1 [file sensors-26-04344-s001.zip › data/images/test/tile_01276_lon48.0_lat27.0.png]

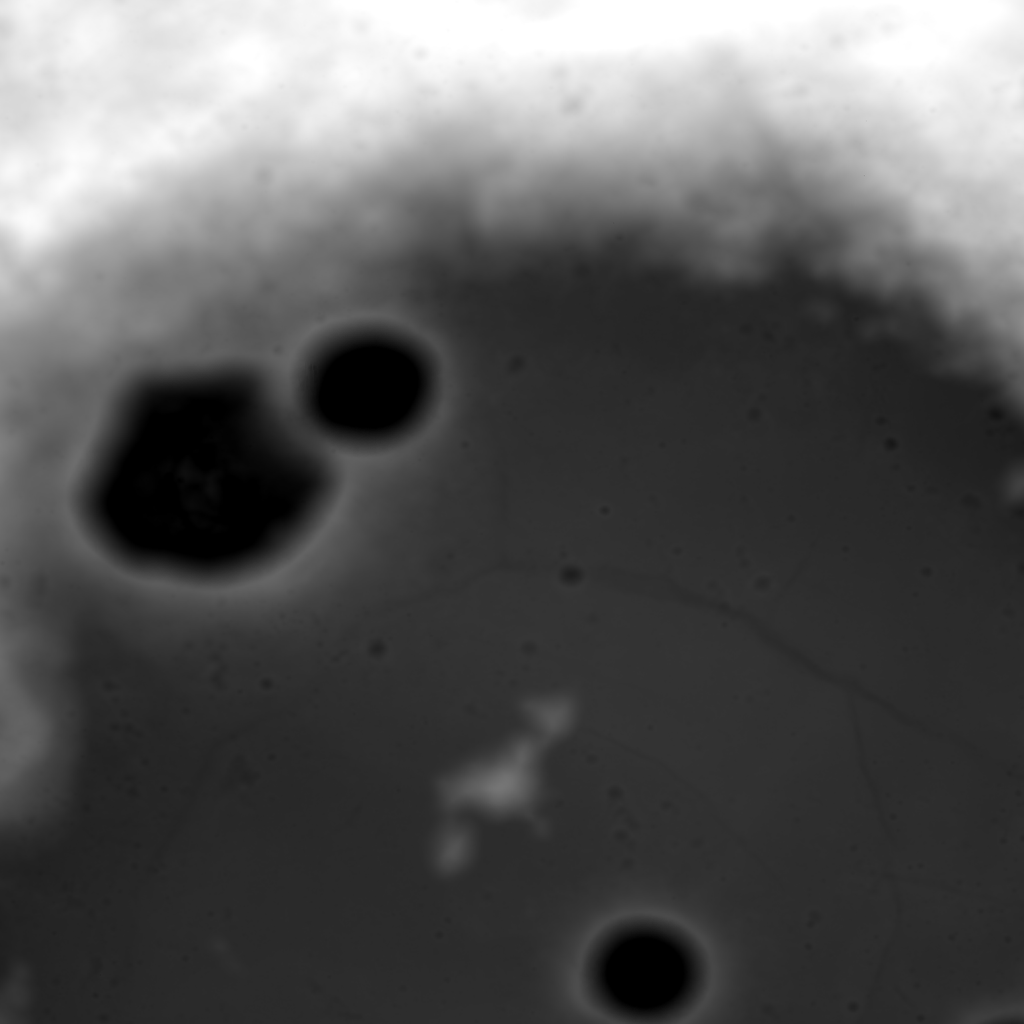

Supplement: Supplementary file 1 [file sensors-26-04344-s001.zip › data/images/test/tile_01278_lon54.0_lat27.0.png]

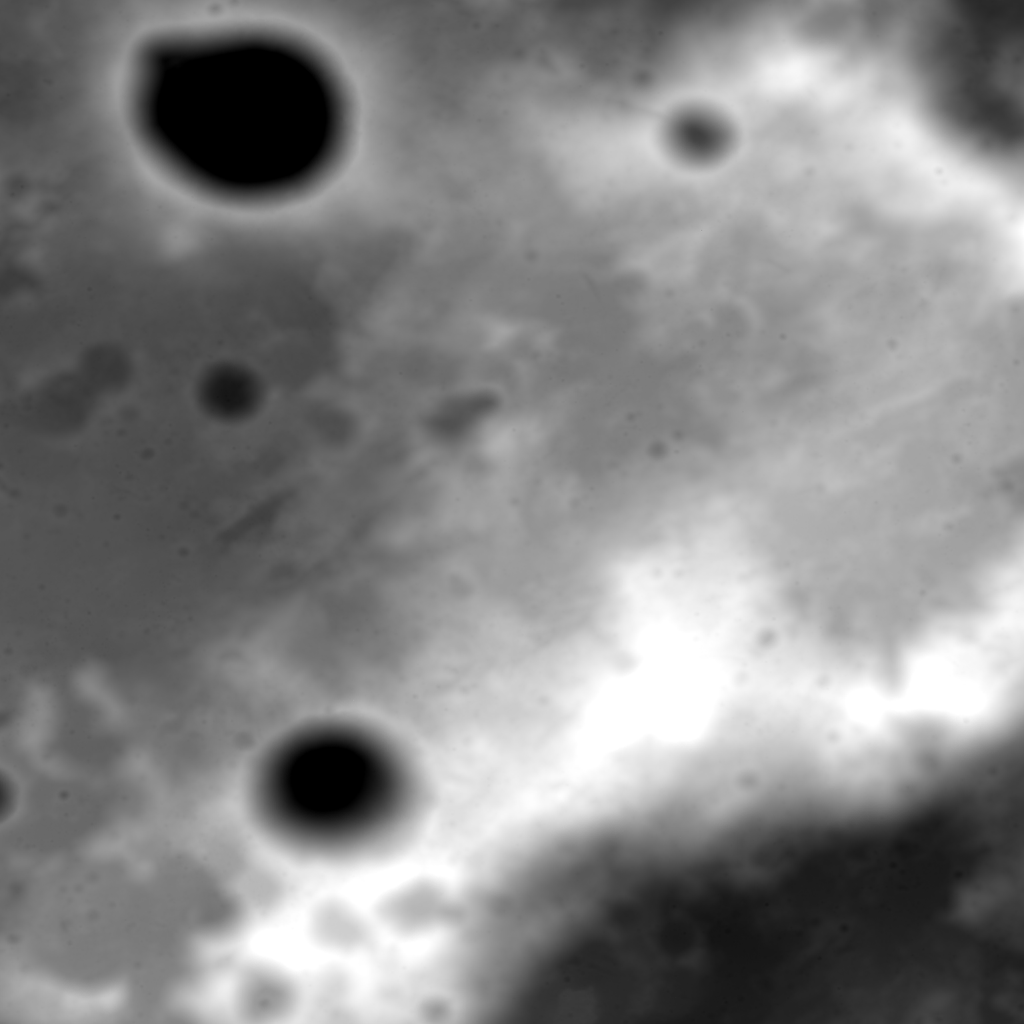

Supplement: Supplementary file 1 [file sensors-26-04344-s001.zip › data/images/test/tile_01283_lon69.0_lat27.0.png]

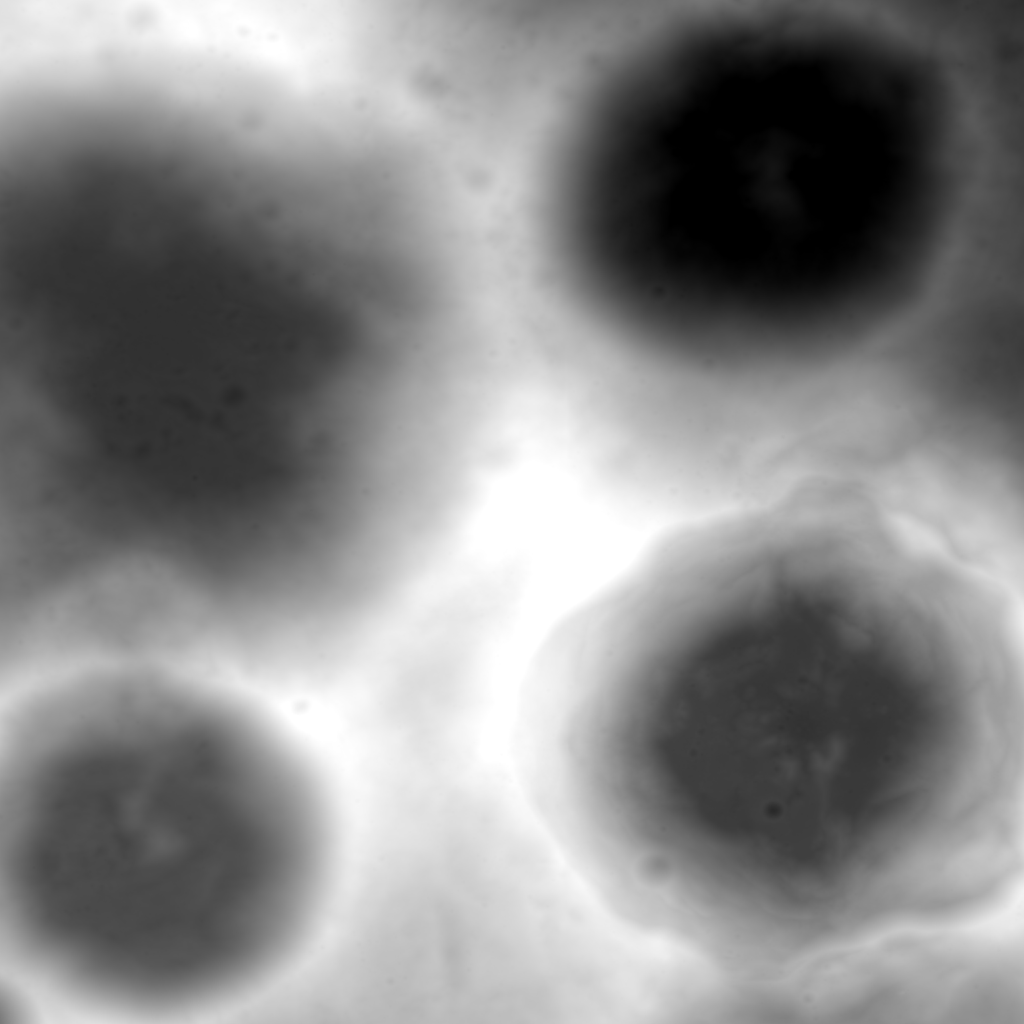

Supplement: Supplementary file 1 [file sensors-26-04344-s001.zip › data/images/test/tile_01299_lon117.0_lat27.0.png]

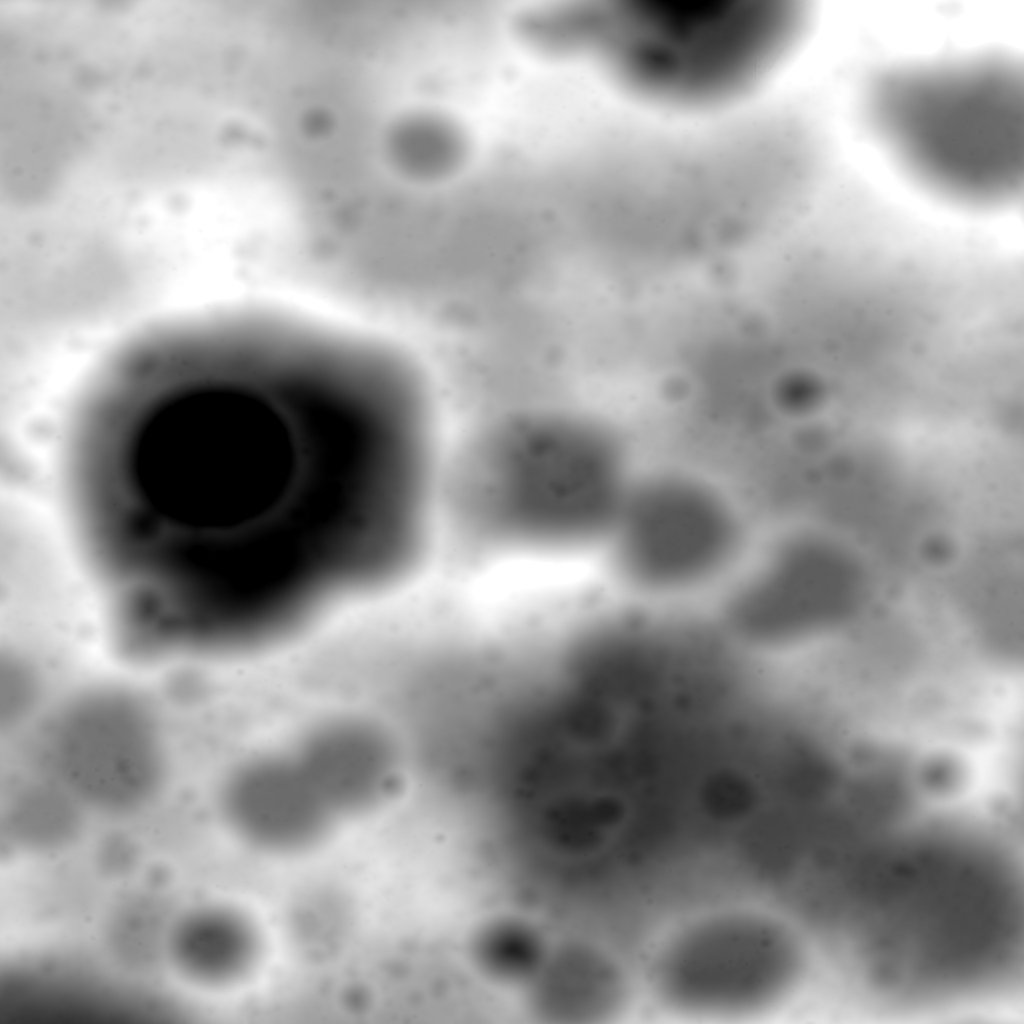

Supplement: Supplementary file 1 [file sensors-26-04344-s001.zip › data/images/test/tile_01302_lon126.0_lat27.0.png]

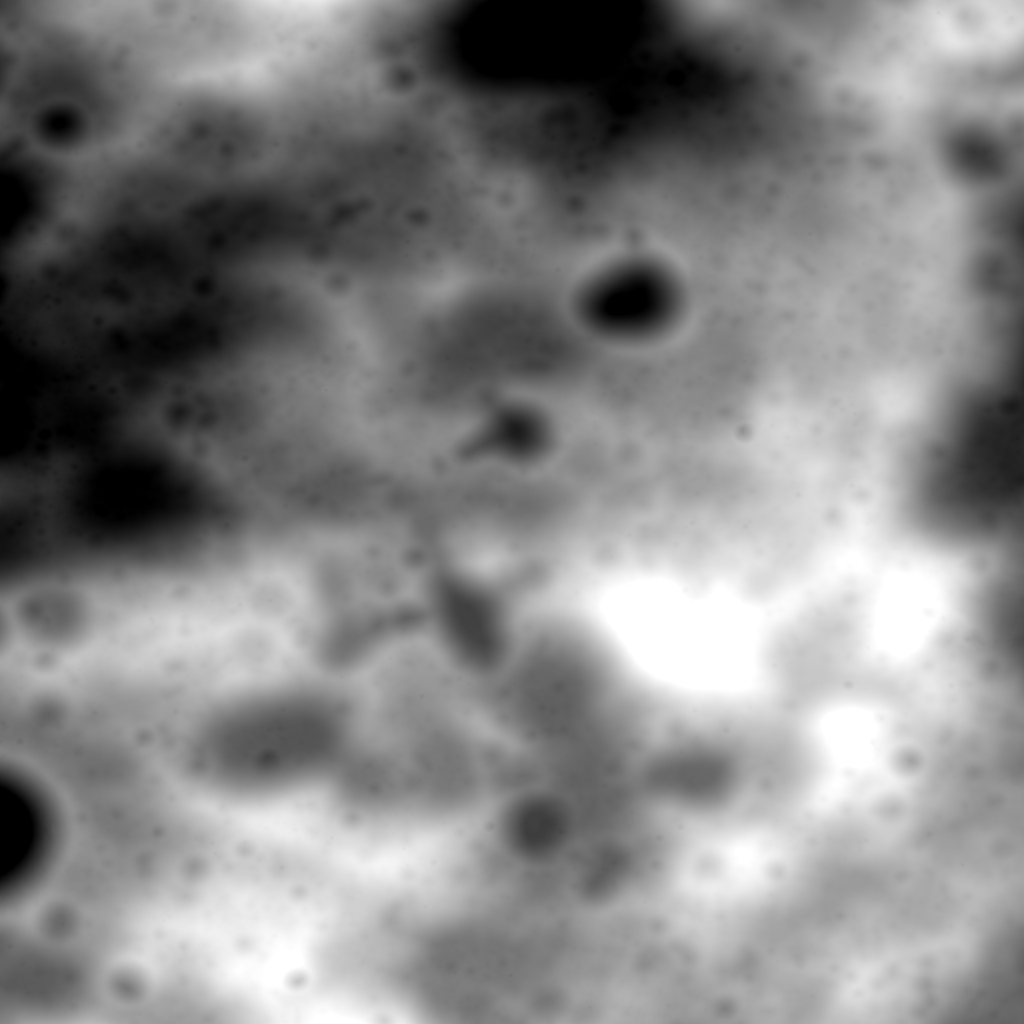

Supplement: Supplementary file 1 [file sensors-26-04344-s001.zip › data/images/test/tile_01305_lon135.0_lat27.0.png]

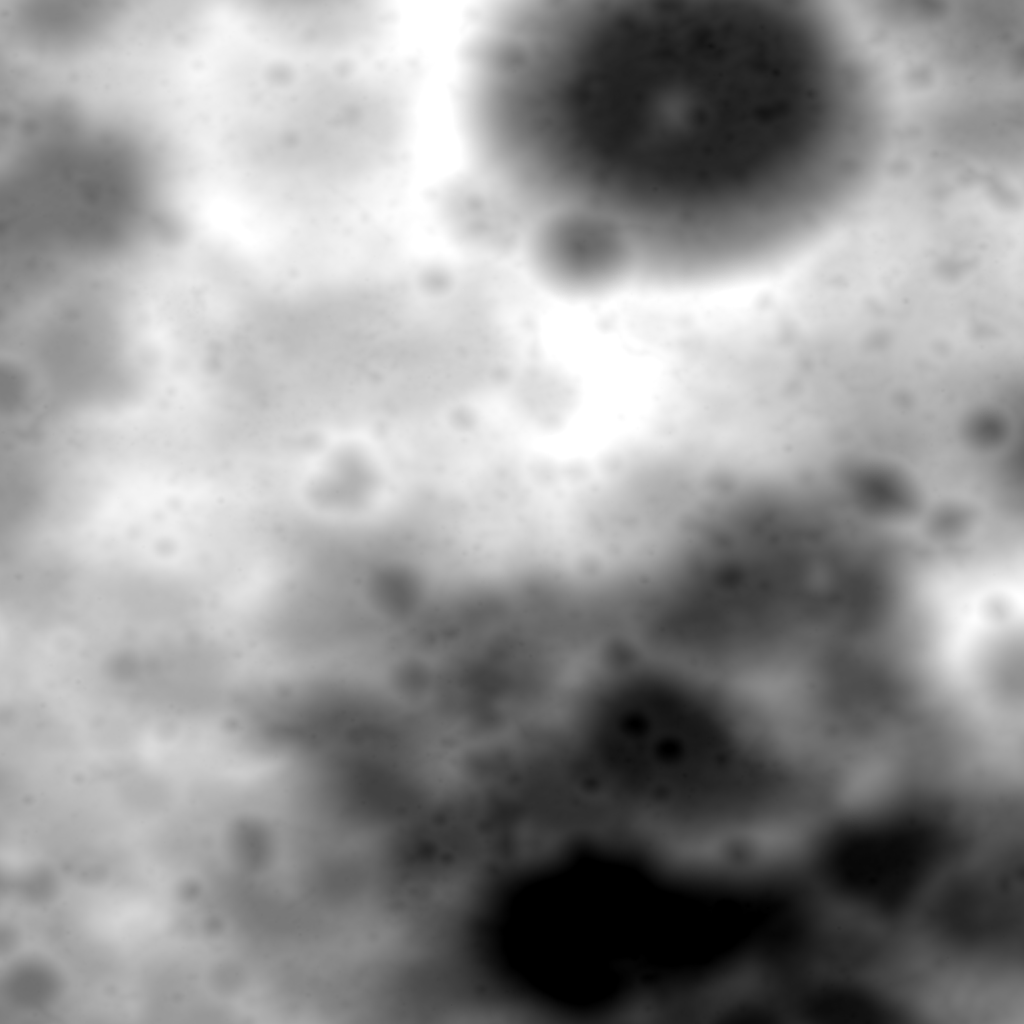

Supplement: Supplementary file 1 [file sensors-26-04344-s001.zip › data/images/test/tile_01314_lon162.0_lat27.0.png]

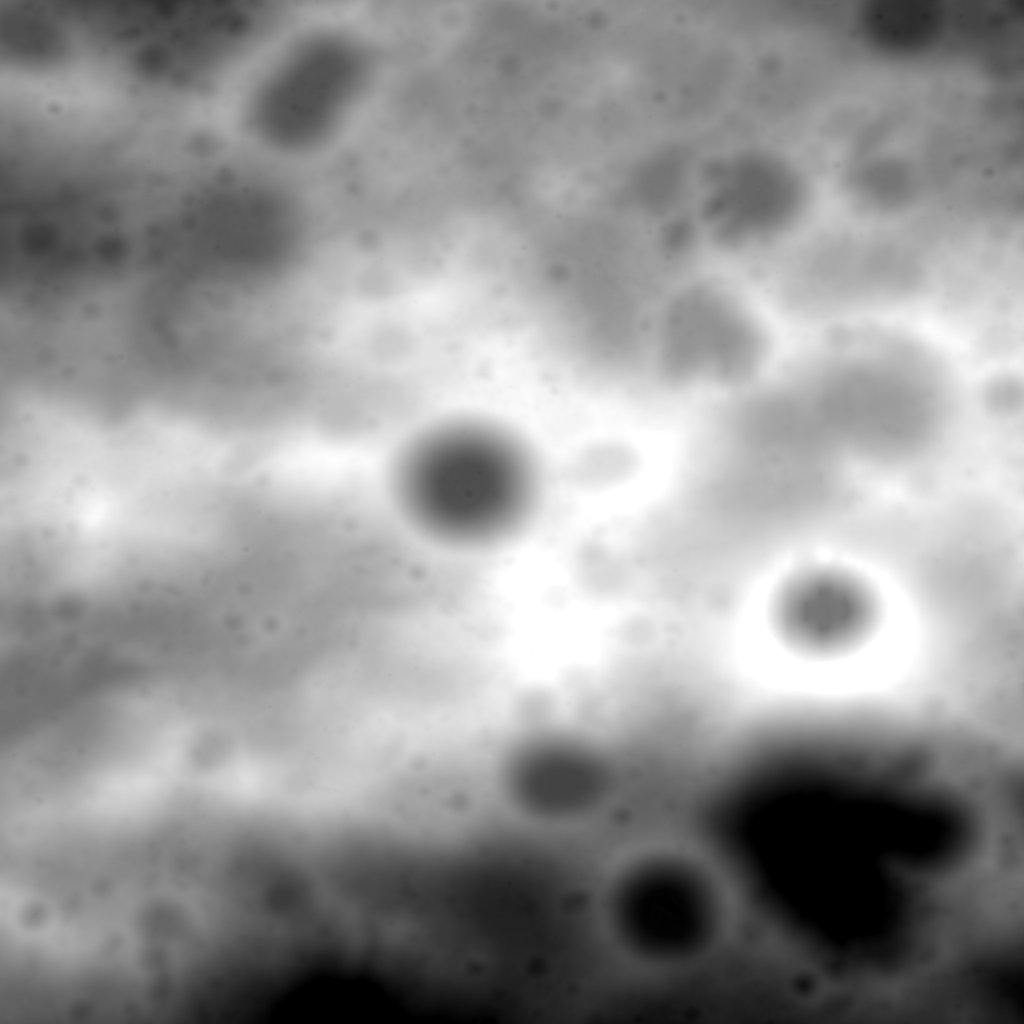

Supplement: Supplementary file 1 [file sensors-26-04344-s001.zip › data/images/test/tile_01317_lon171.0_lat27.0.png]

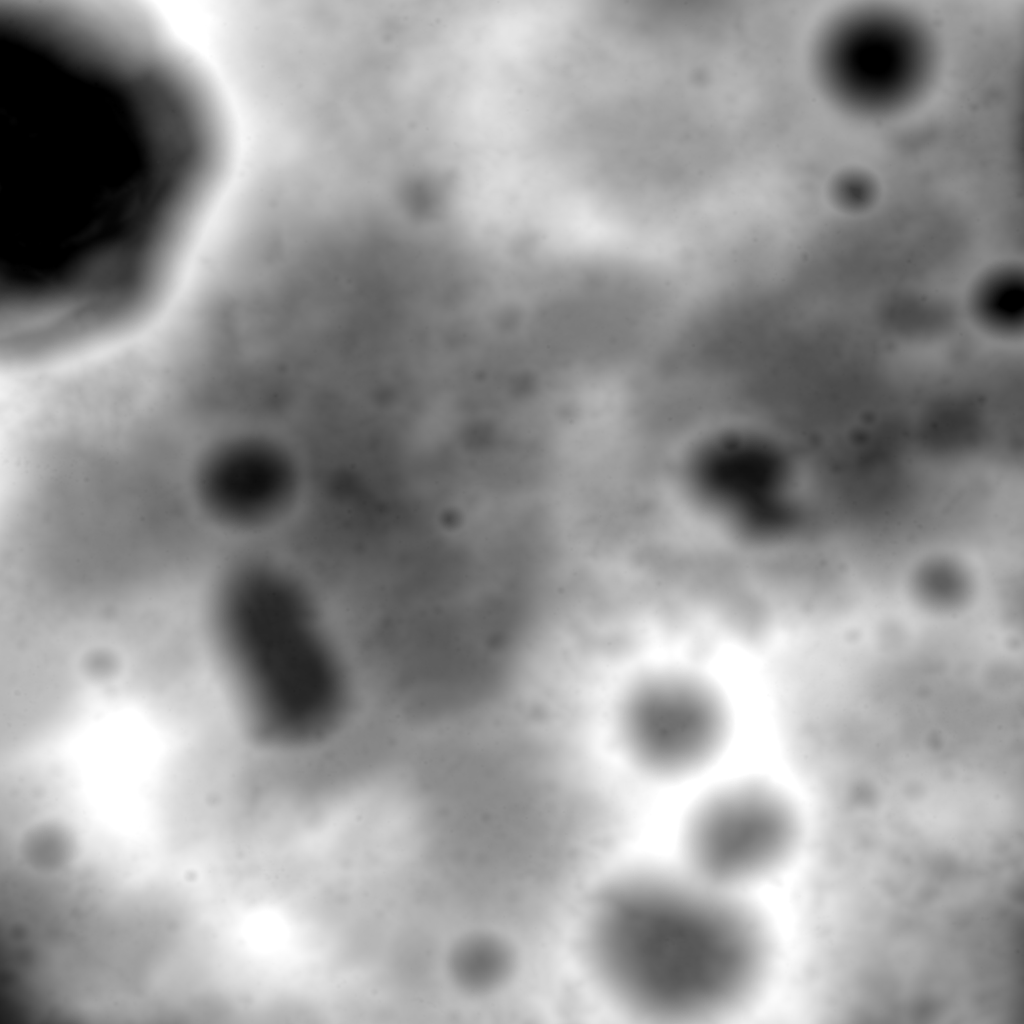

Supplement: Supplementary file 1 [file sensors-26-04344-s001.zip › data/images/test/tile_01321_lon-177.0_lat24.0.png]

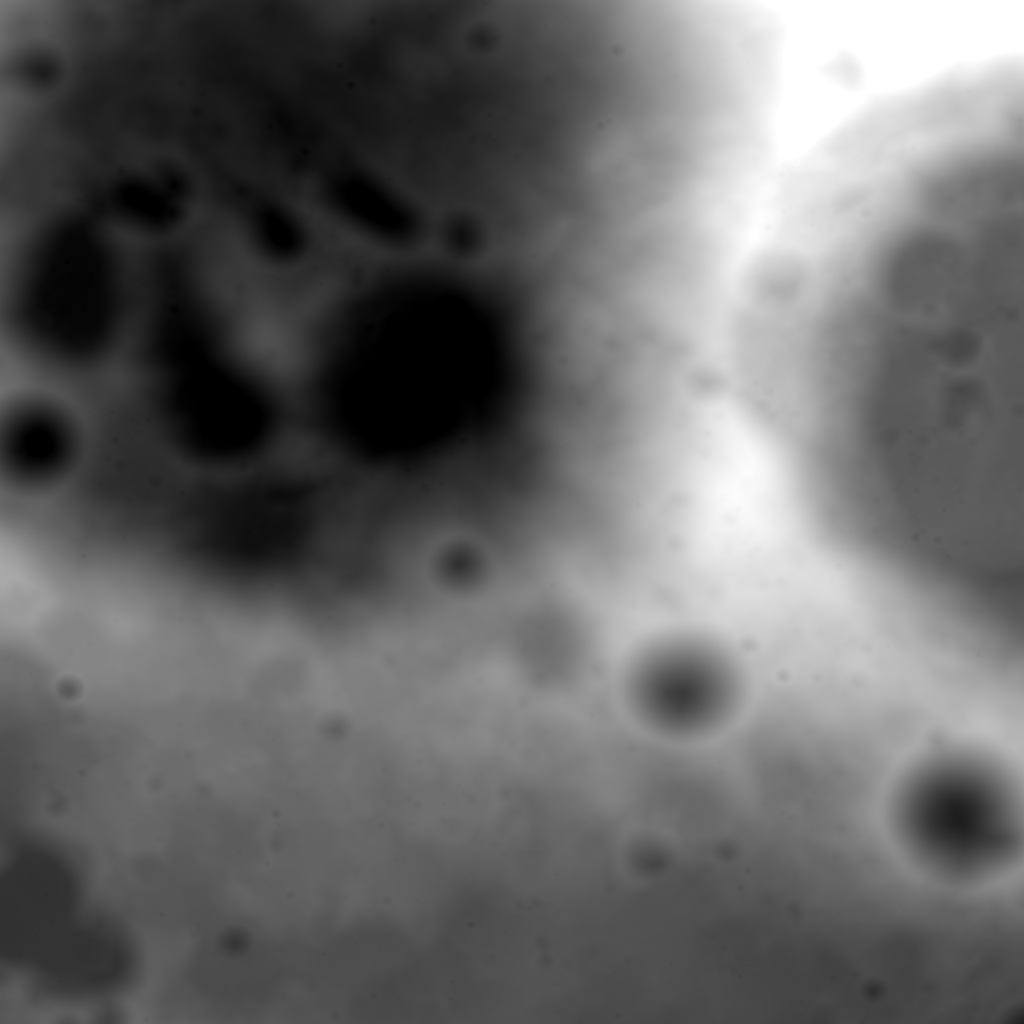

Supplement: Supplementary file 1 [file sensors-26-04344-s001.zip › data/images/test/tile_01334_lon-138.0_lat24.0.png]

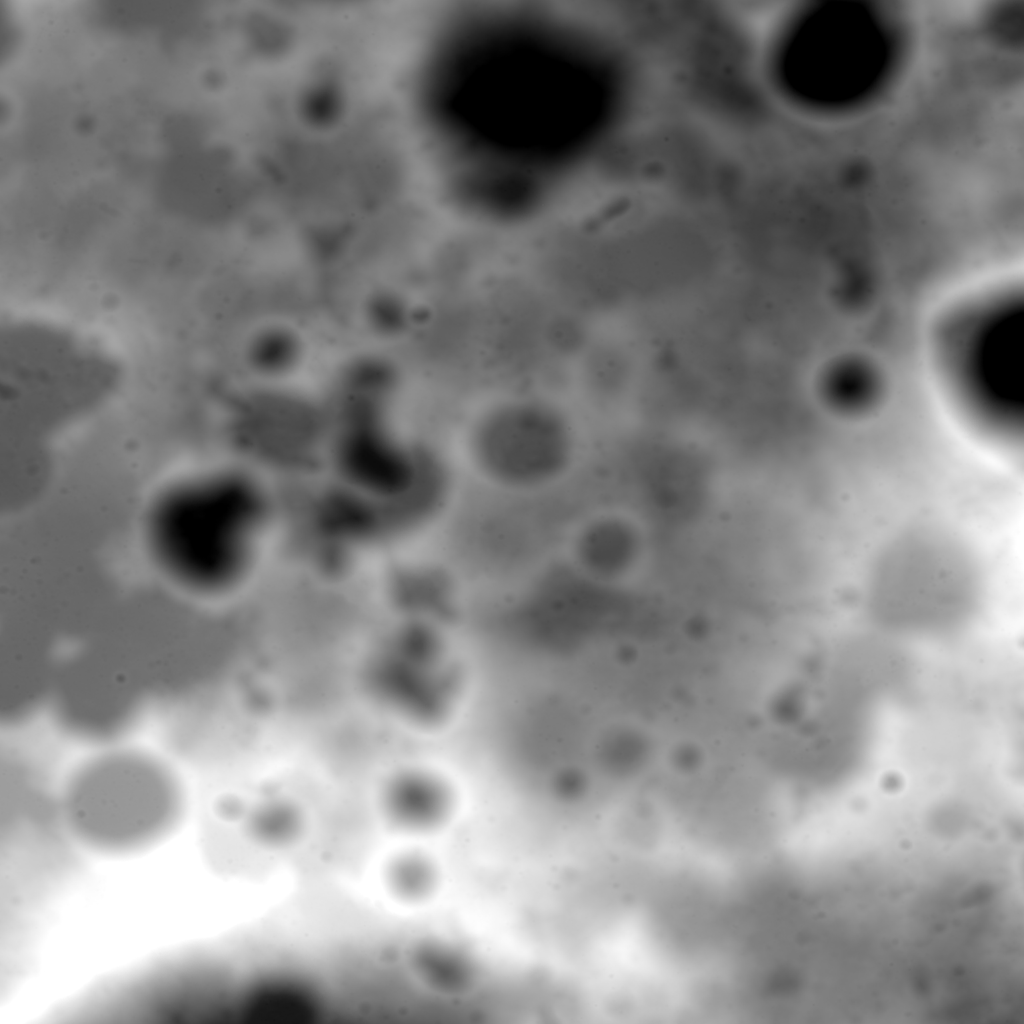

Supplement: Supplementary file 1 [file sensors-26-04344-s001.zip › data/images/test/tile_01342_lon-114.0_lat24.0.png]

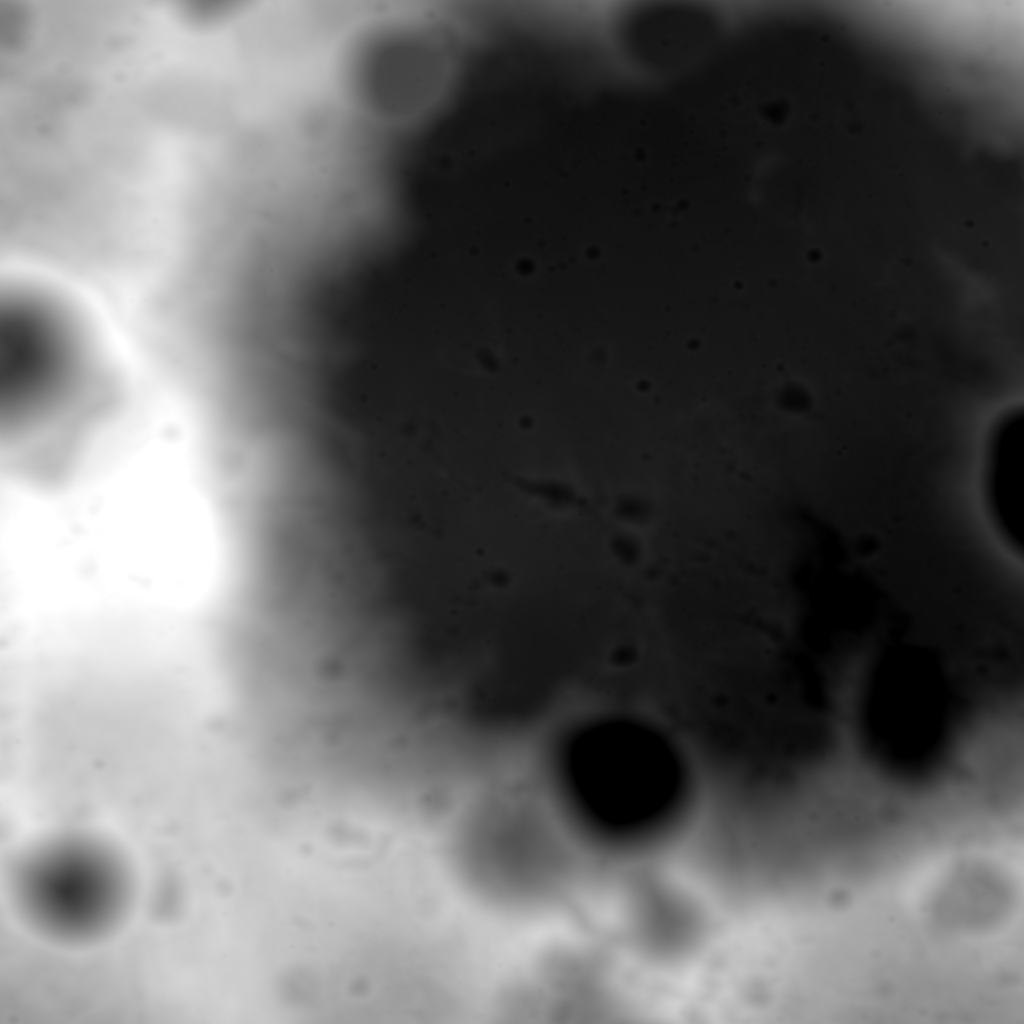

Supplement: Supplementary file 1 [file sensors-26-04344-s001.zip › data/images/test/tile_01343_lon-111.0_lat24.0.png]

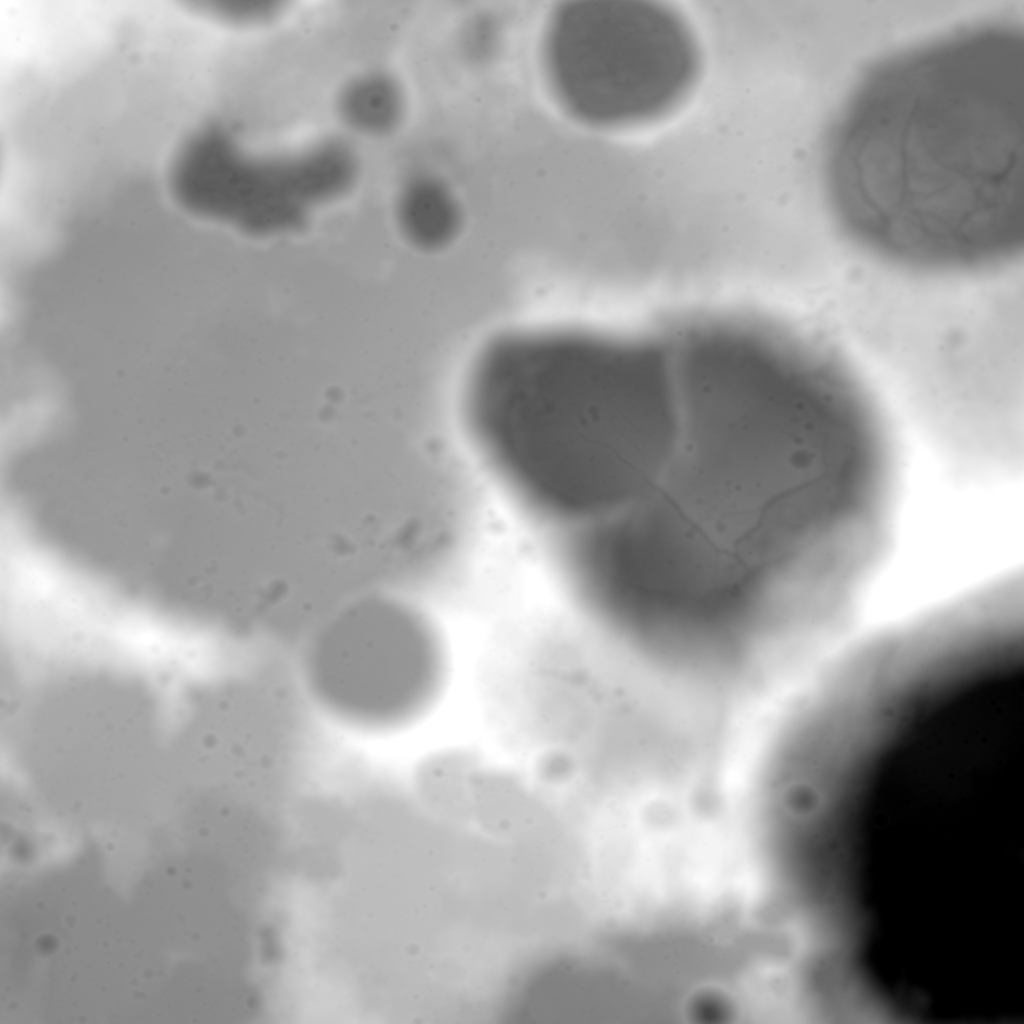

Supplement: Supplementary file 1 [file sensors-26-04344-s001.zip › data/images/test/tile_01349_lon-93.0_lat24.0.png]

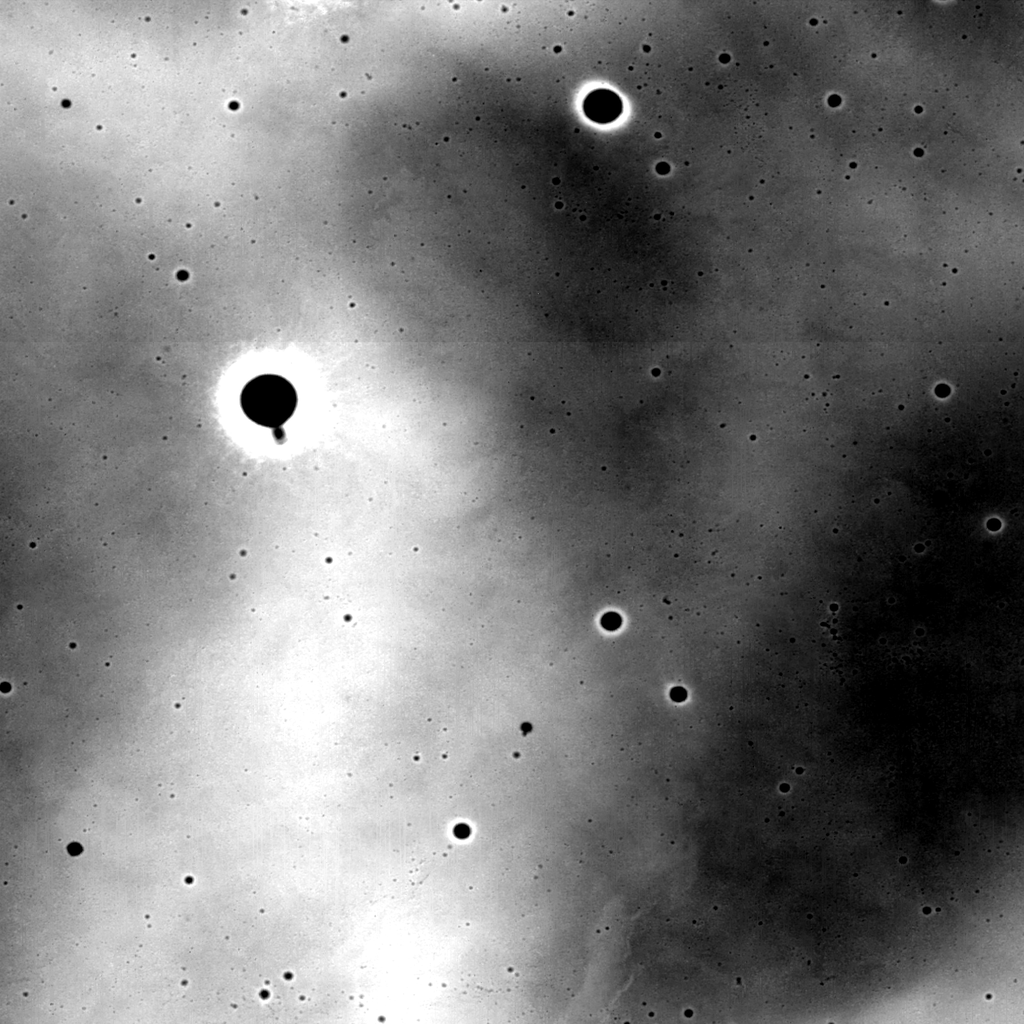

Supplement: Supplementary file 1 [file sensors-26-04344-s001.zip › data/images/test/tile_01359_lon-63.0_lat24.0.png]

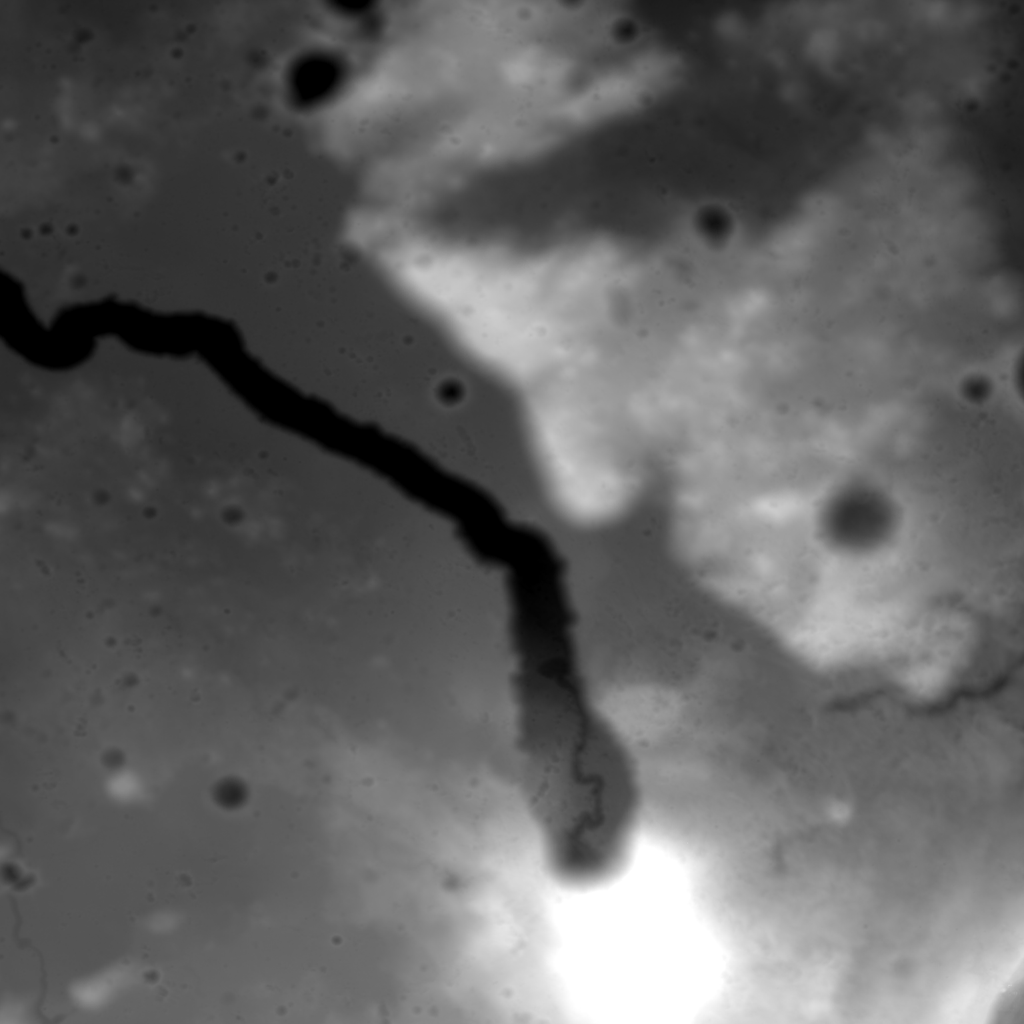

Supplement: Supplementary file 1 [file sensors-26-04344-s001.zip › data/images/test/tile_01363_lon-51.0_lat24.0.png]

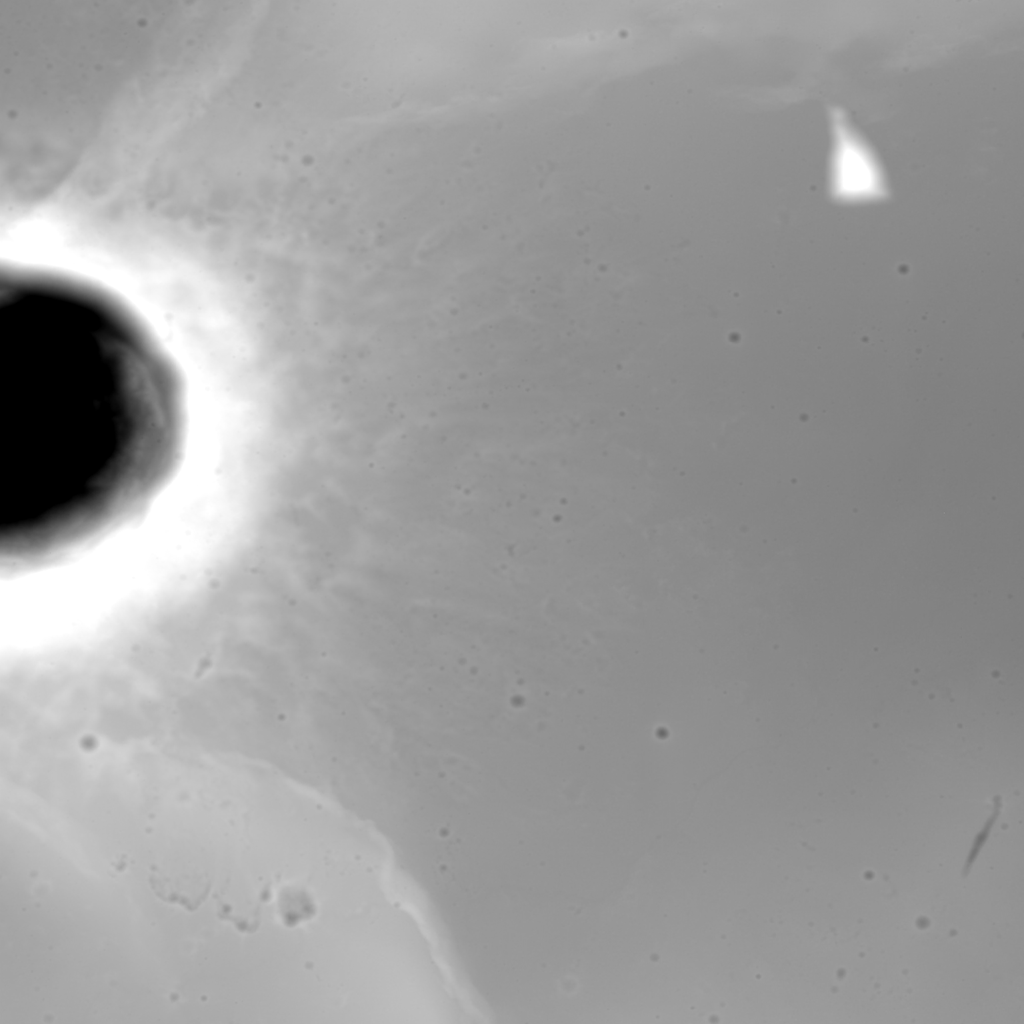

Supplement: Supplementary file 1 [file sensors-26-04344-s001.zip › data/images/test/tile_01373_lon-21.0_lat24.0.png]

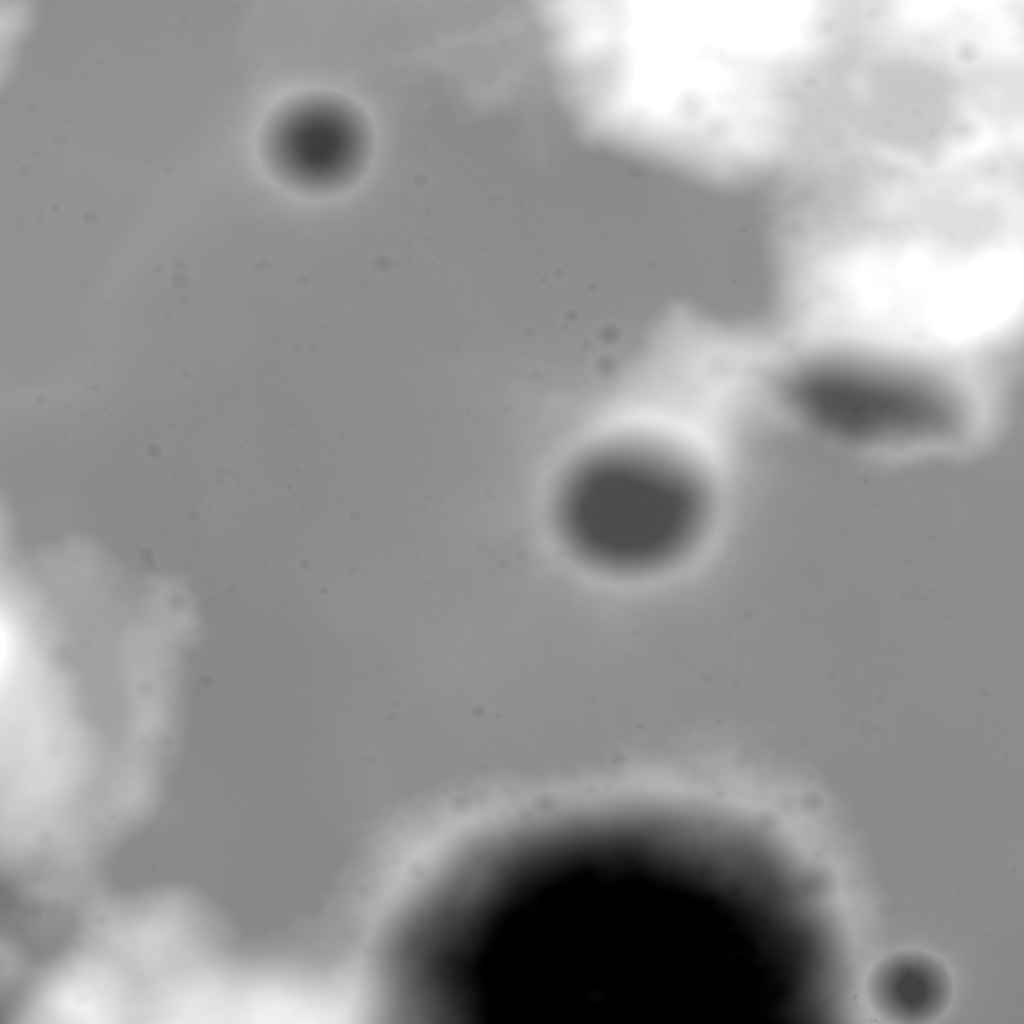

Supplement: Supplementary file 1 [file sensors-26-04344-s001.zip › data/images/test/tile_01401_lon63.0_lat24.0.png]

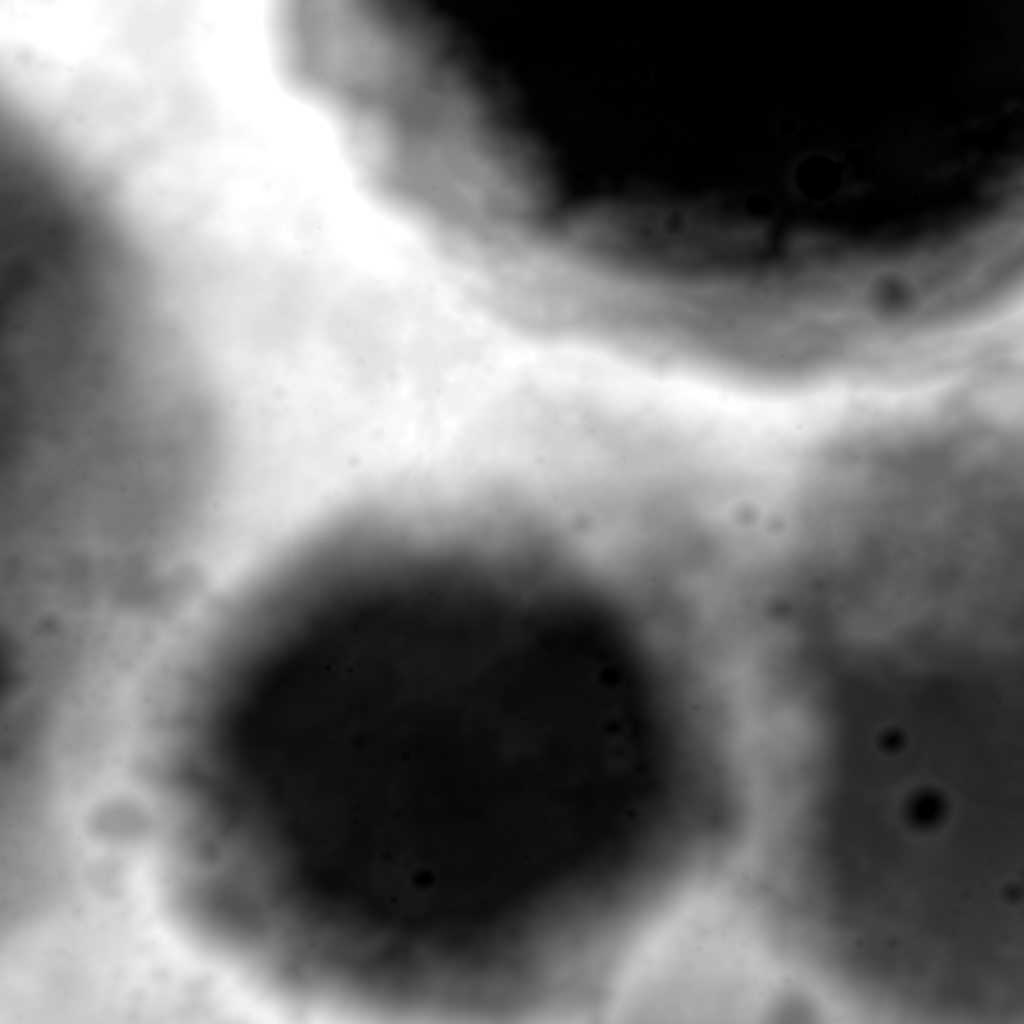

Supplement: Supplementary file 1 [file sensors-26-04344-s001.zip › data/images/test/tile_01412_lon96.0_lat24.0.png]

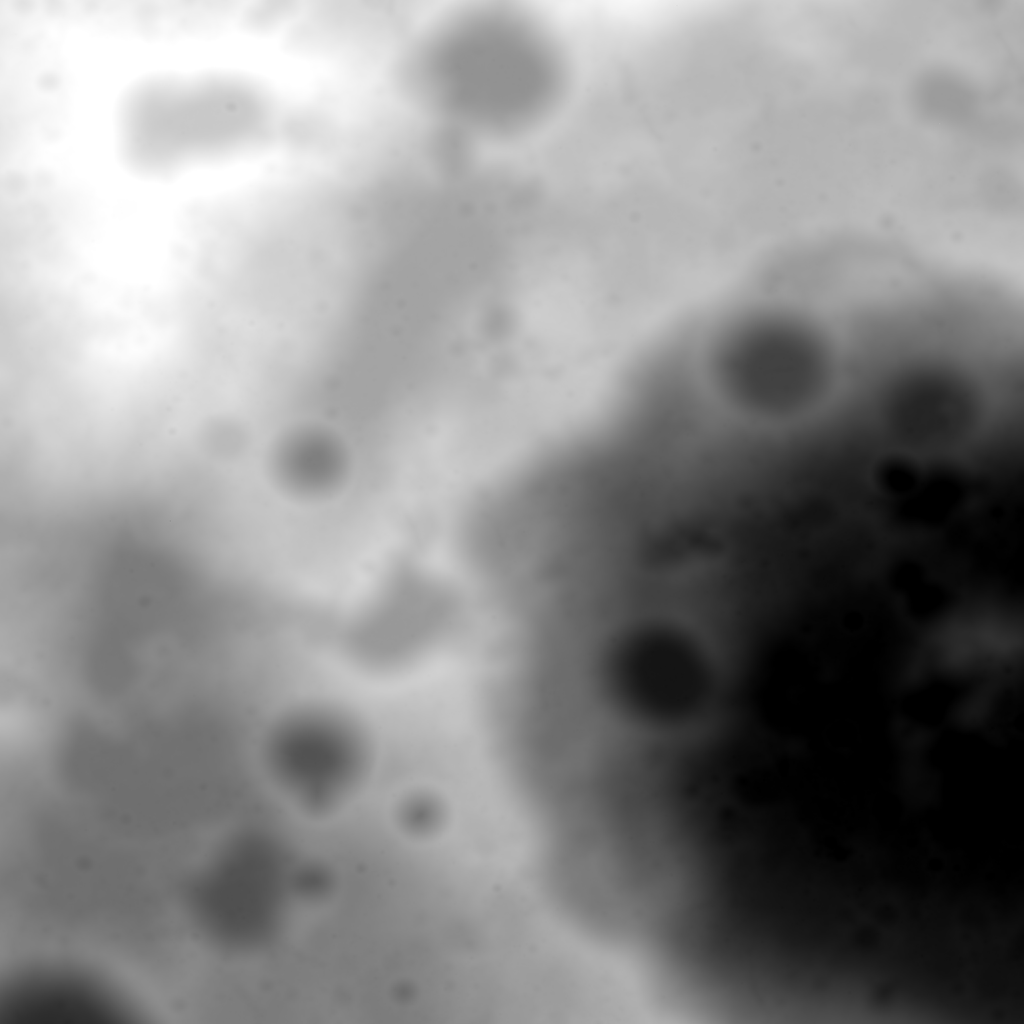

Supplement: Supplementary file 1 [file sensors-26-04344-s001.zip › data/images/test/tile_01436_lon168.0_lat24.0.png]

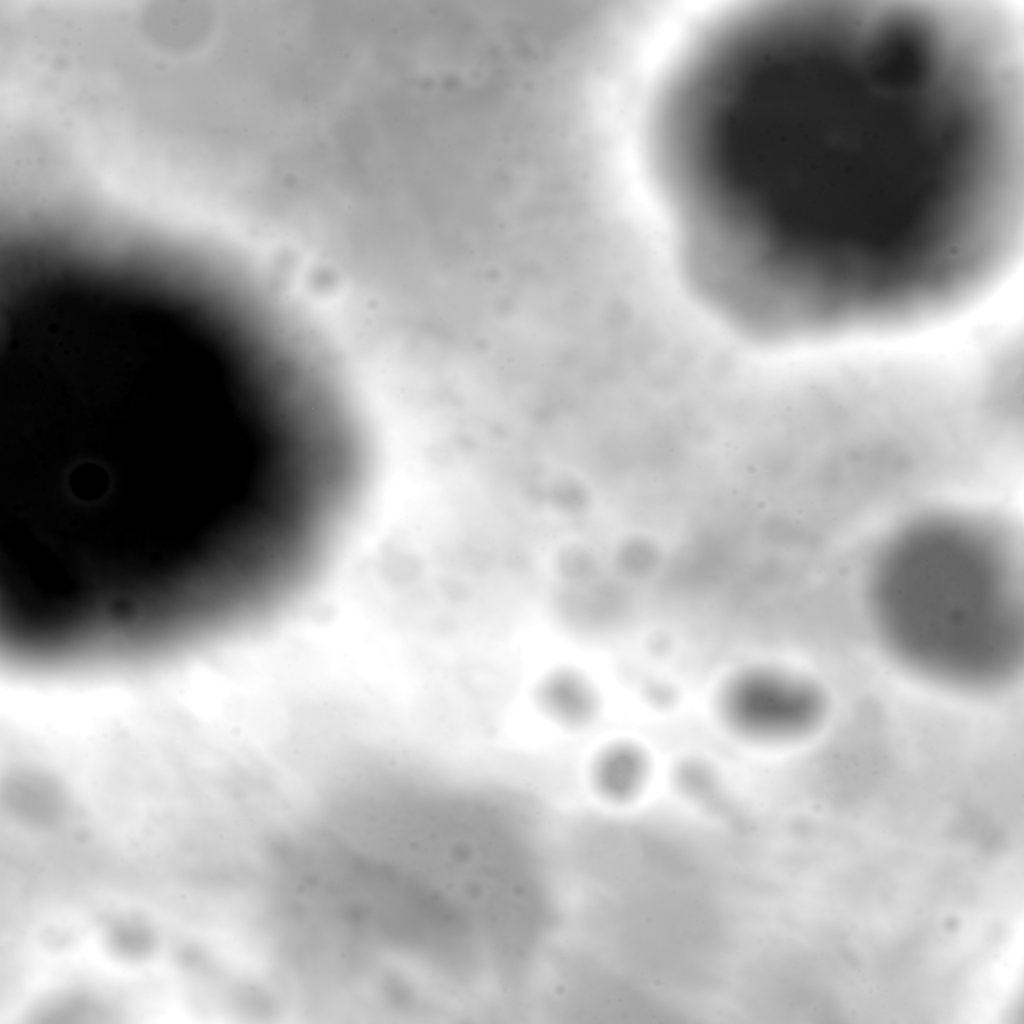

Supplement: Supplementary file 1 [file sensors-26-04344-s001.zip › data/images/test/tile_01452_lon-144.0_lat21.0.png]

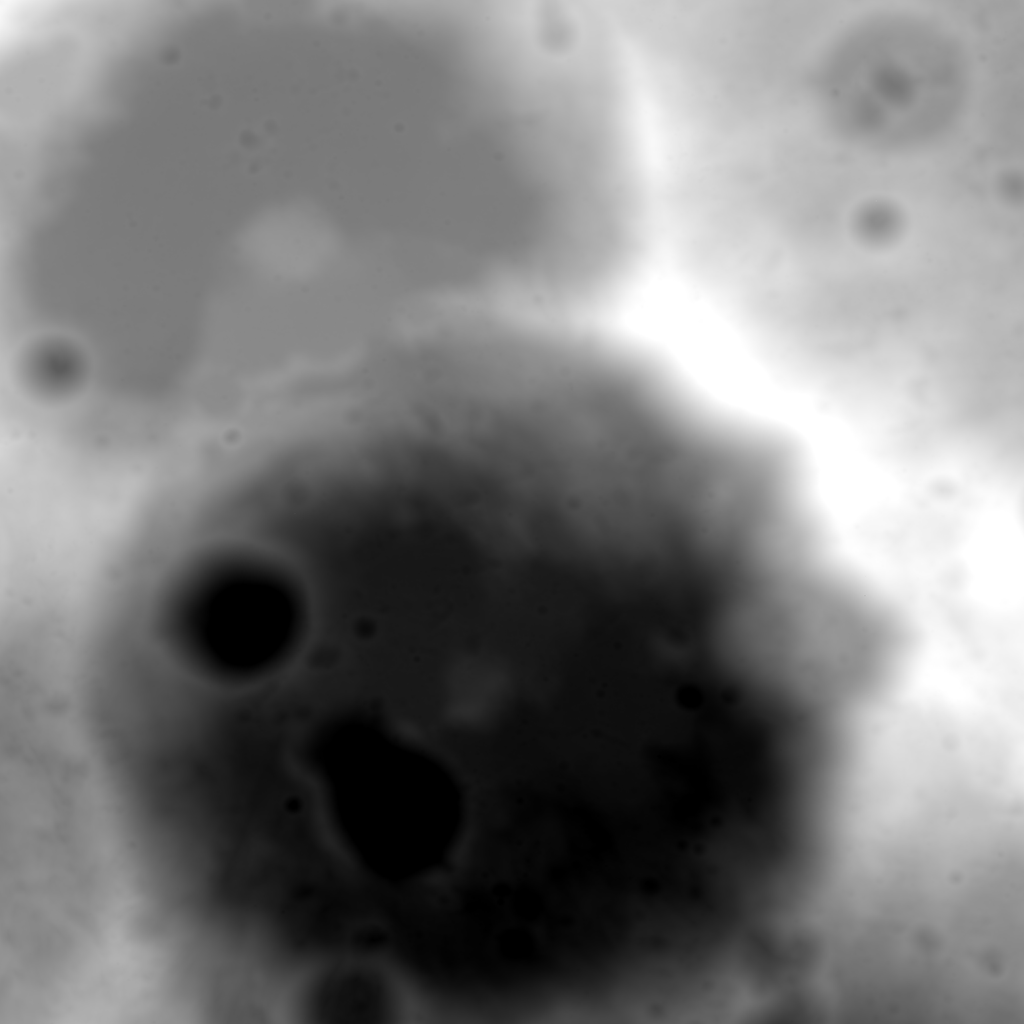

Supplement: Supplementary file 1 [file sensors-26-04344-s001.zip › data/images/test/tile_01462_lon-114.0_lat21.0.png]

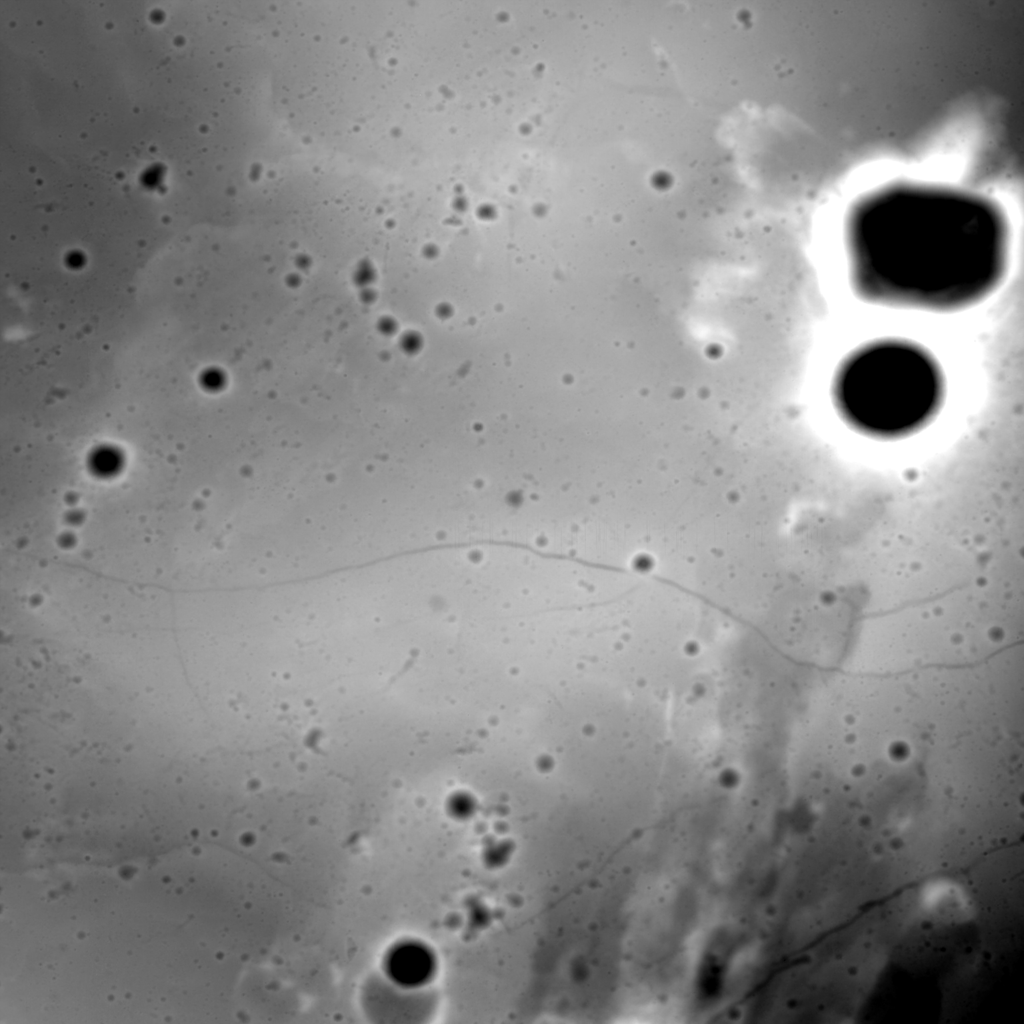

Supplement: Supplementary file 1 [file sensors-26-04344-s001.zip › data/images/test/tile_01474_lon-78.0_lat21.0.png]

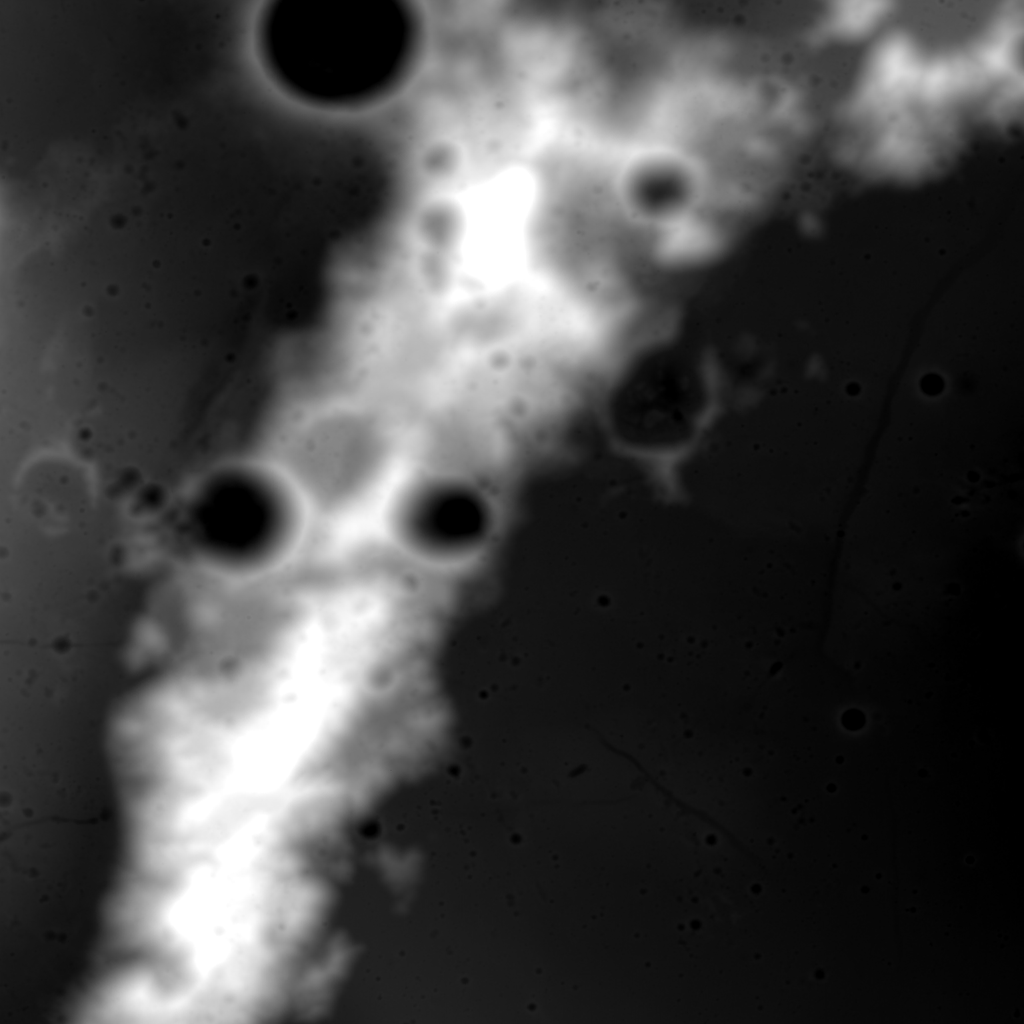

Supplement: Supplementary file 1 [file sensors-26-04344-s001.zip › data/images/test/tile_01475_lon-75.0_lat21.0.png]

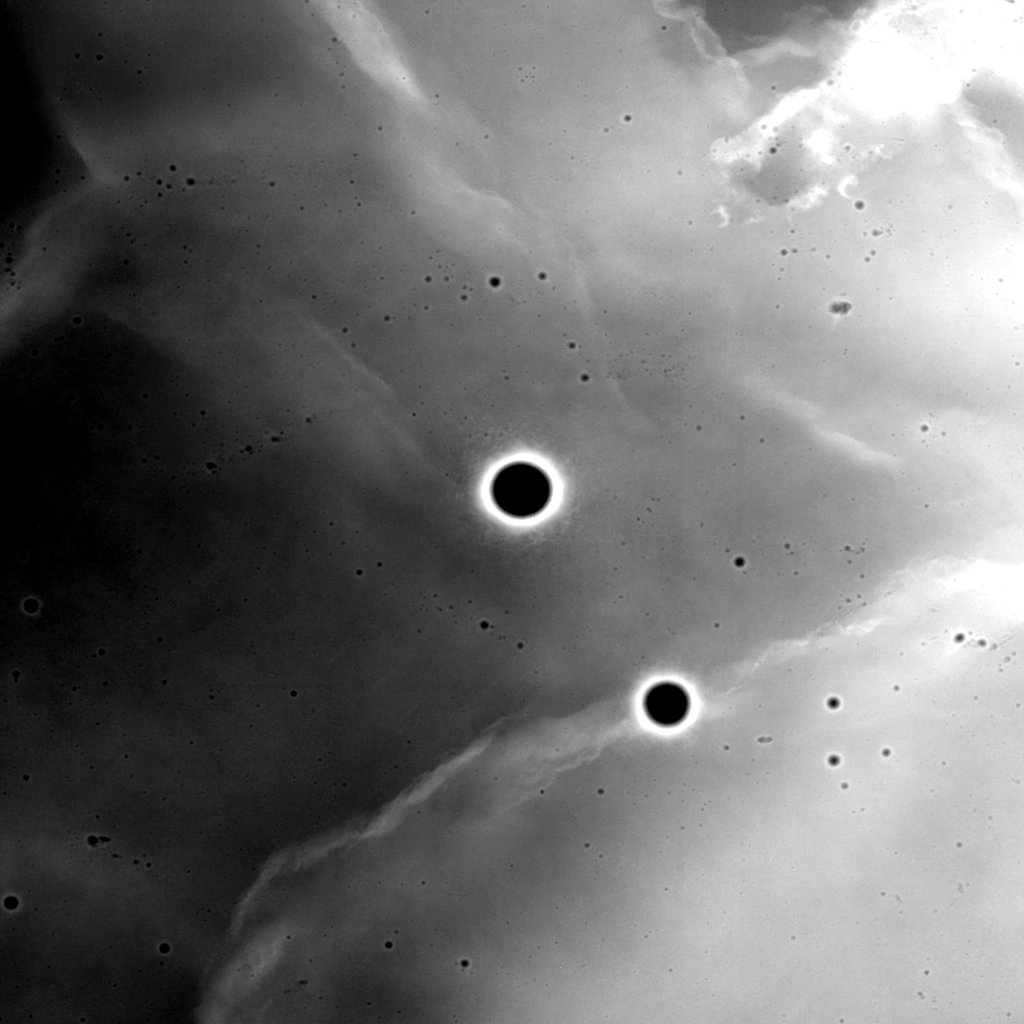

Supplement: Supplementary file 1 [file sensors-26-04344-s001.zip › data/images/test/tile_01481_lon-57.0_lat21.0.png]

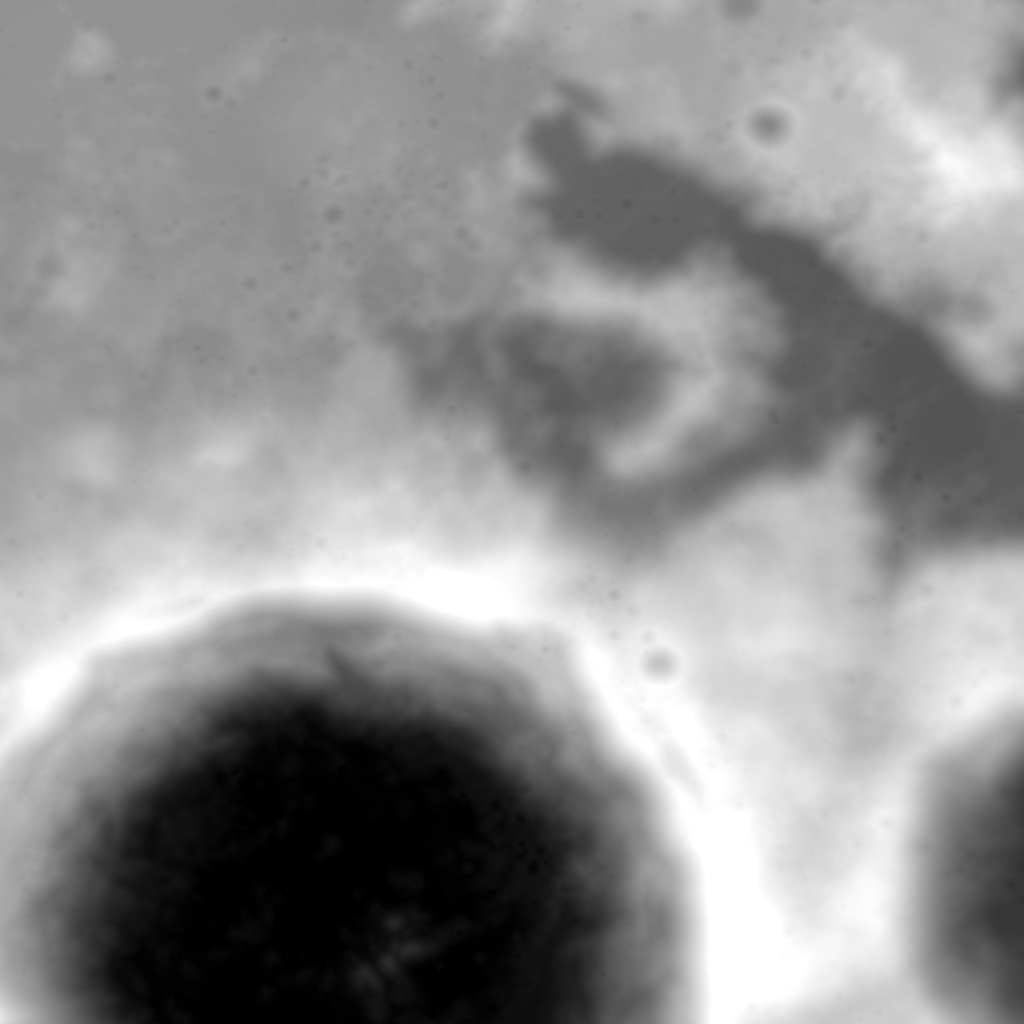

Supplement: Supplementary file 1 [file sensors-26-04344-s001.zip › data/images/test/tile_01515_lon45.0_lat21.0.png]

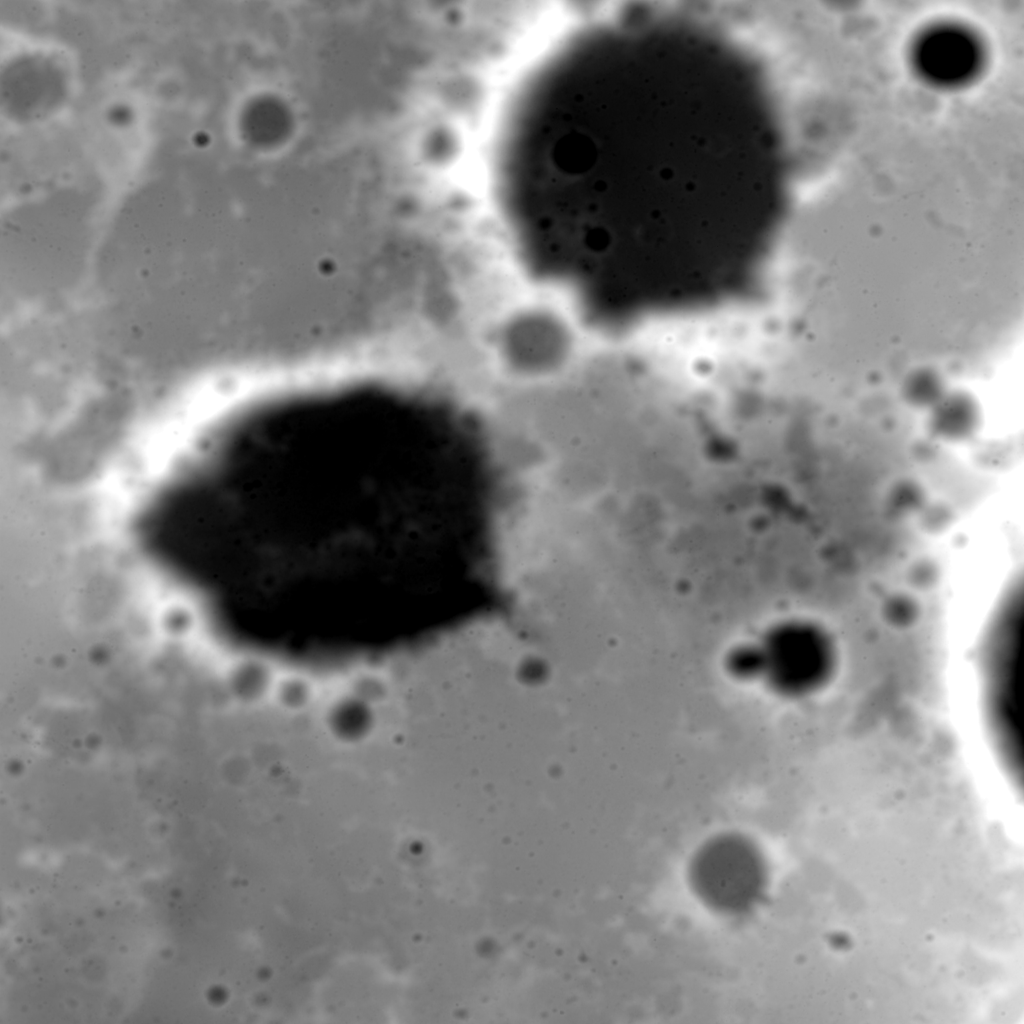

Supplement: Supplementary file 1 [file sensors-26-04344-s001.zip › data/images/test/tile_01534_lon102.0_lat21.0.png]

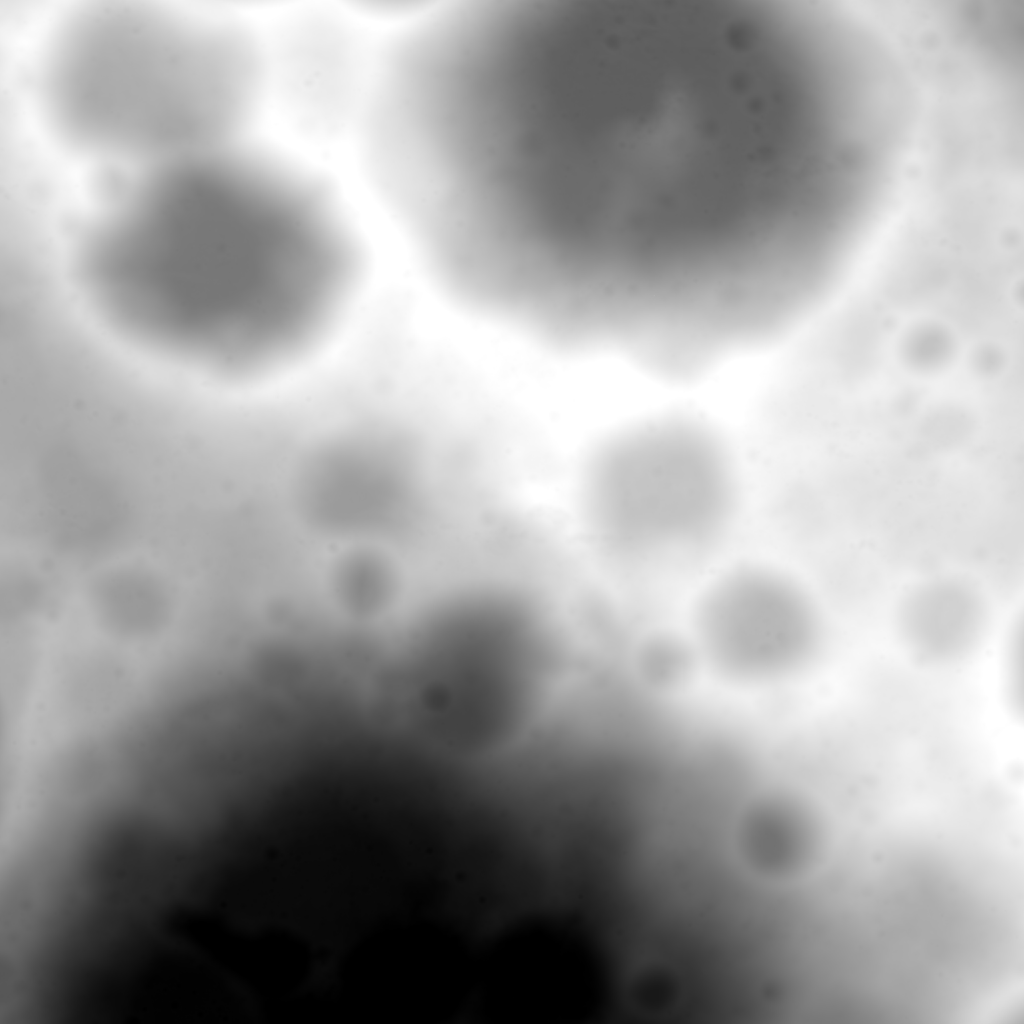

Supplement: Supplementary file 1 [file sensors-26-04344-s001.zip › data/images/test/tile_01568_lon-156.0_lat18.0.png]

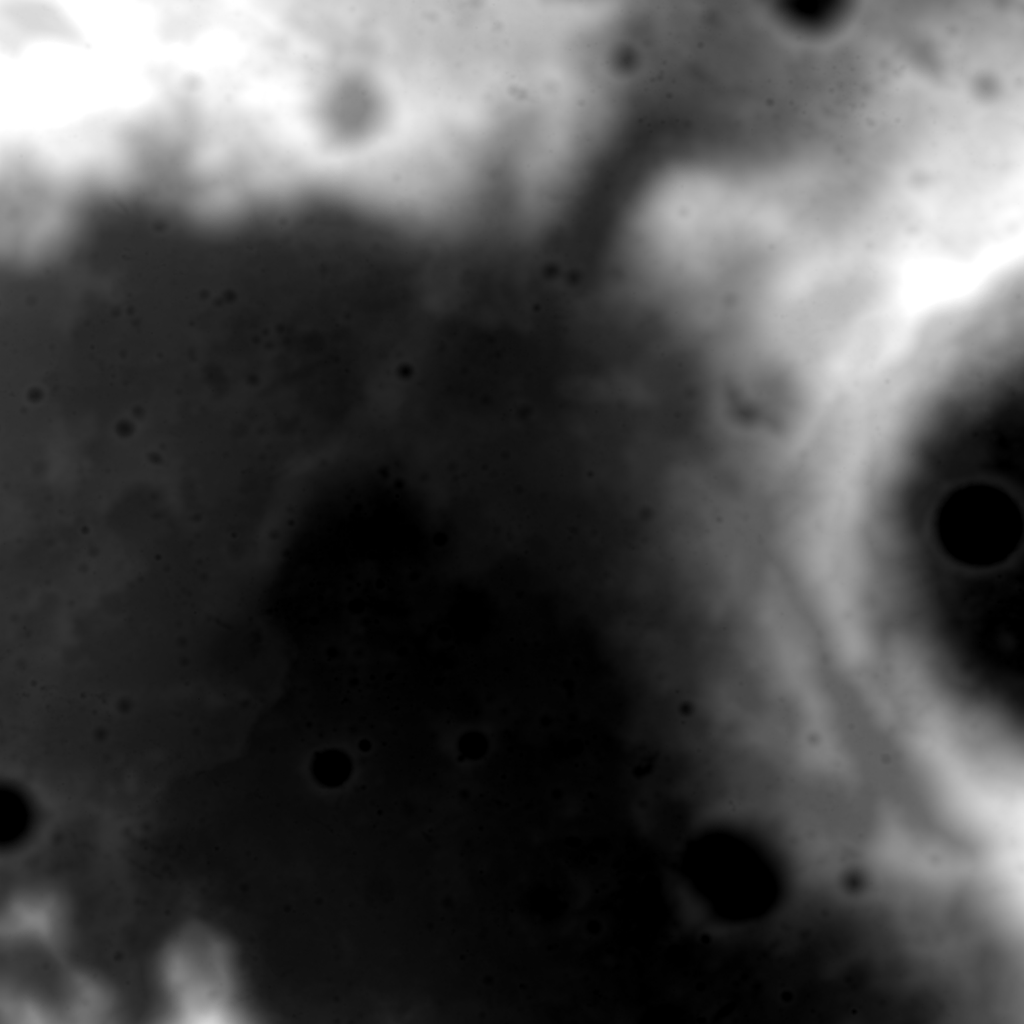

Supplement: Supplementary file 1 [file sensors-26-04344-s001.zip › data/images/test/tile_01570_lon-150.0_lat18.0.png]

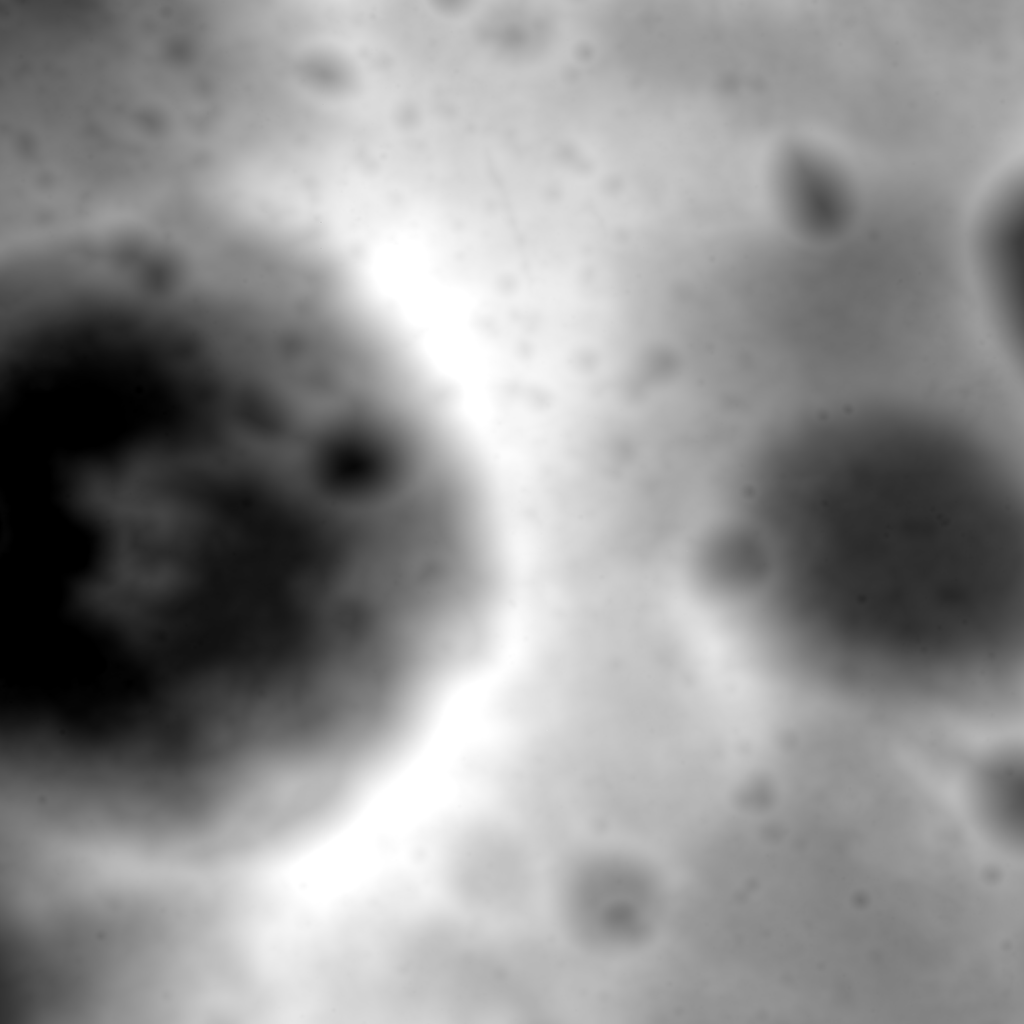

Supplement: Supplementary file 1 [file sensors-26-04344-s001.zip › data/images/test/tile_01571_lon-147.0_lat18.0.png]

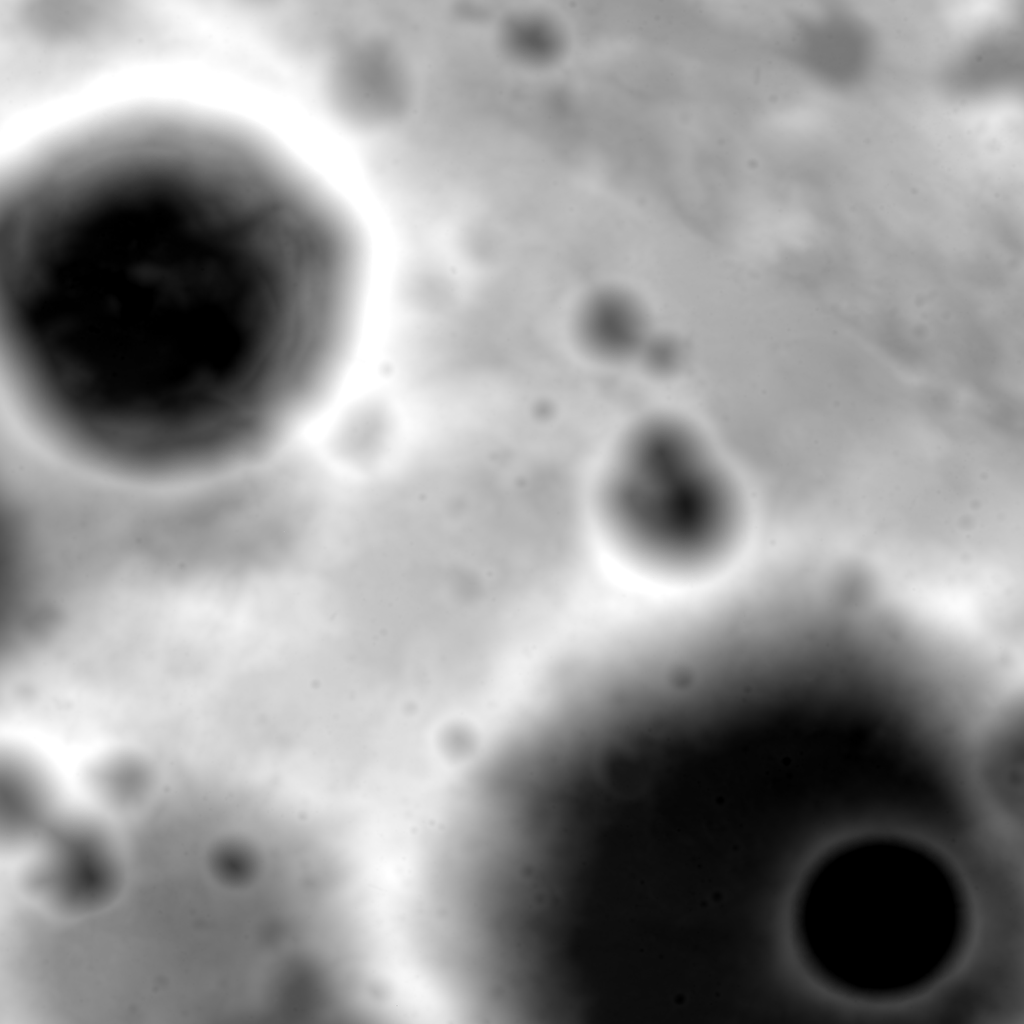

Supplement: Supplementary file 1 [file sensors-26-04344-s001.zip › data/images/test/tile_01572_lon-144.0_lat18.0.png]

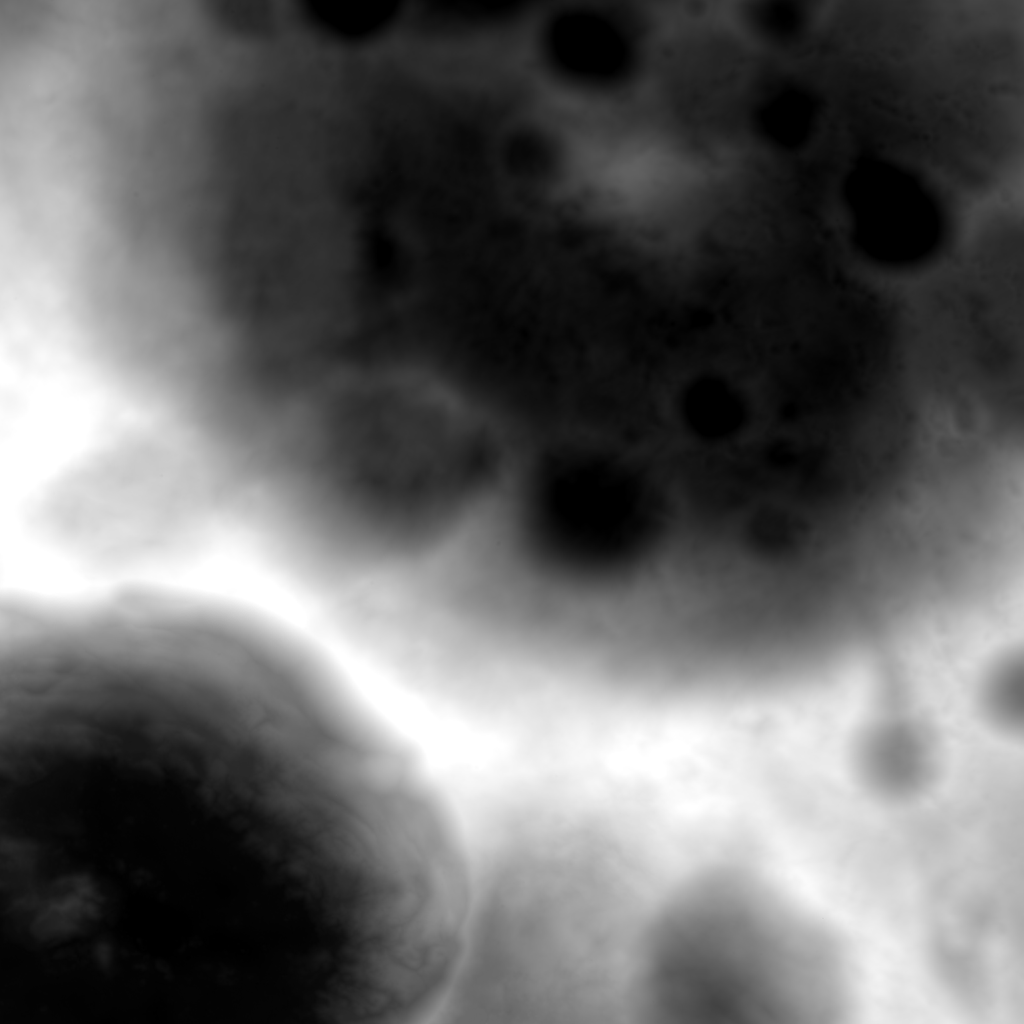

Supplement: Supplementary file 1 [file sensors-26-04344-s001.zip › data/images/test/tile_01582_lon-114.0_lat18.0.png]

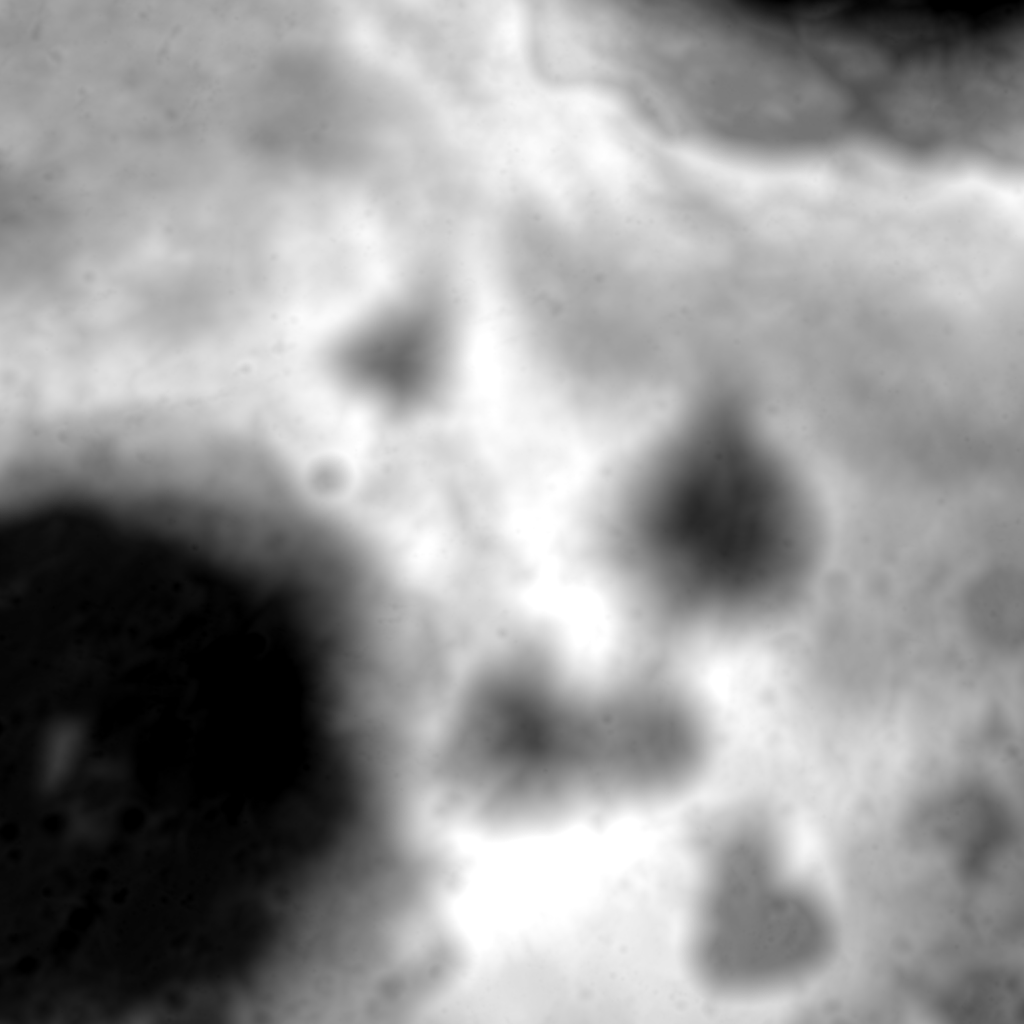

Supplement: Supplementary file 1 [file sensors-26-04344-s001.zip › data/images/test/tile_01584_lon-108.0_lat18.0.png]

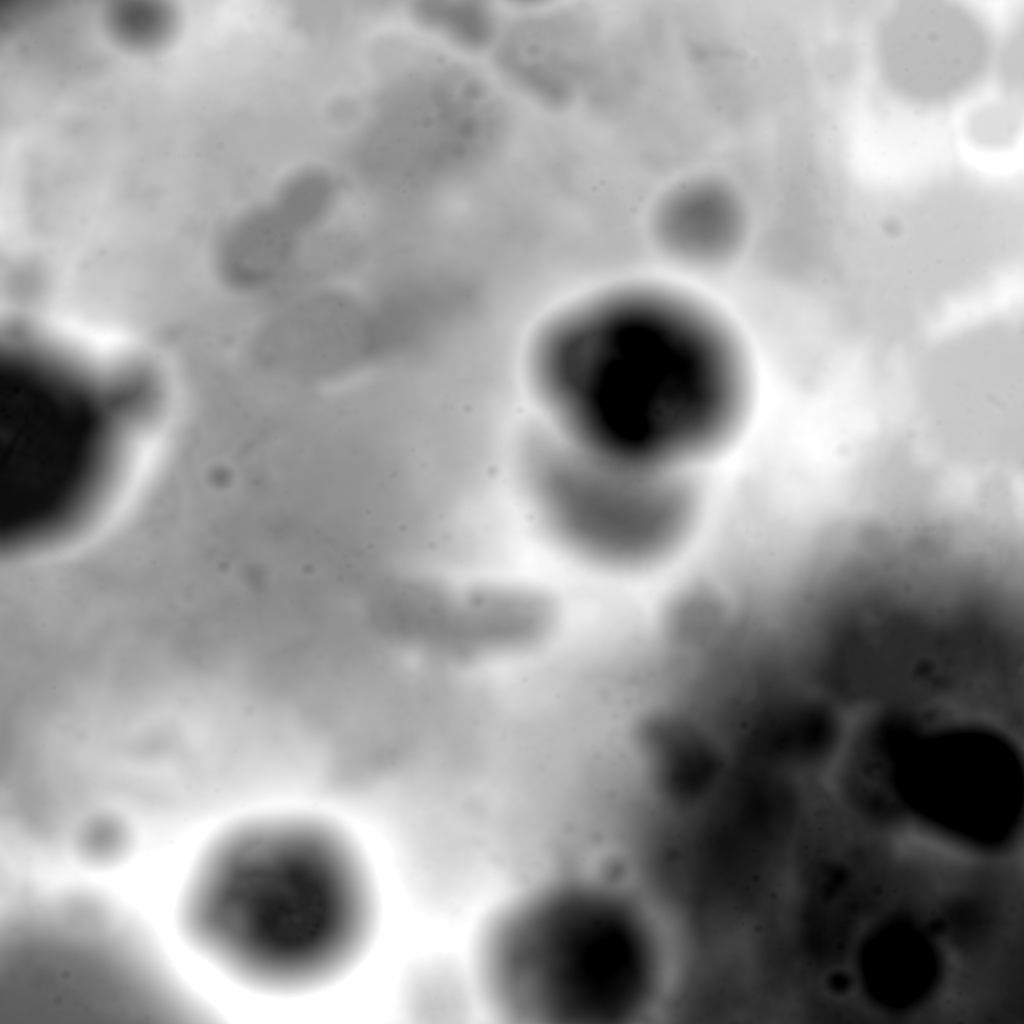

Supplement: Supplementary file 1 [file sensors-26-04344-s001.zip › data/images/test/tile_01588_lon-96.0_lat18.0.png]

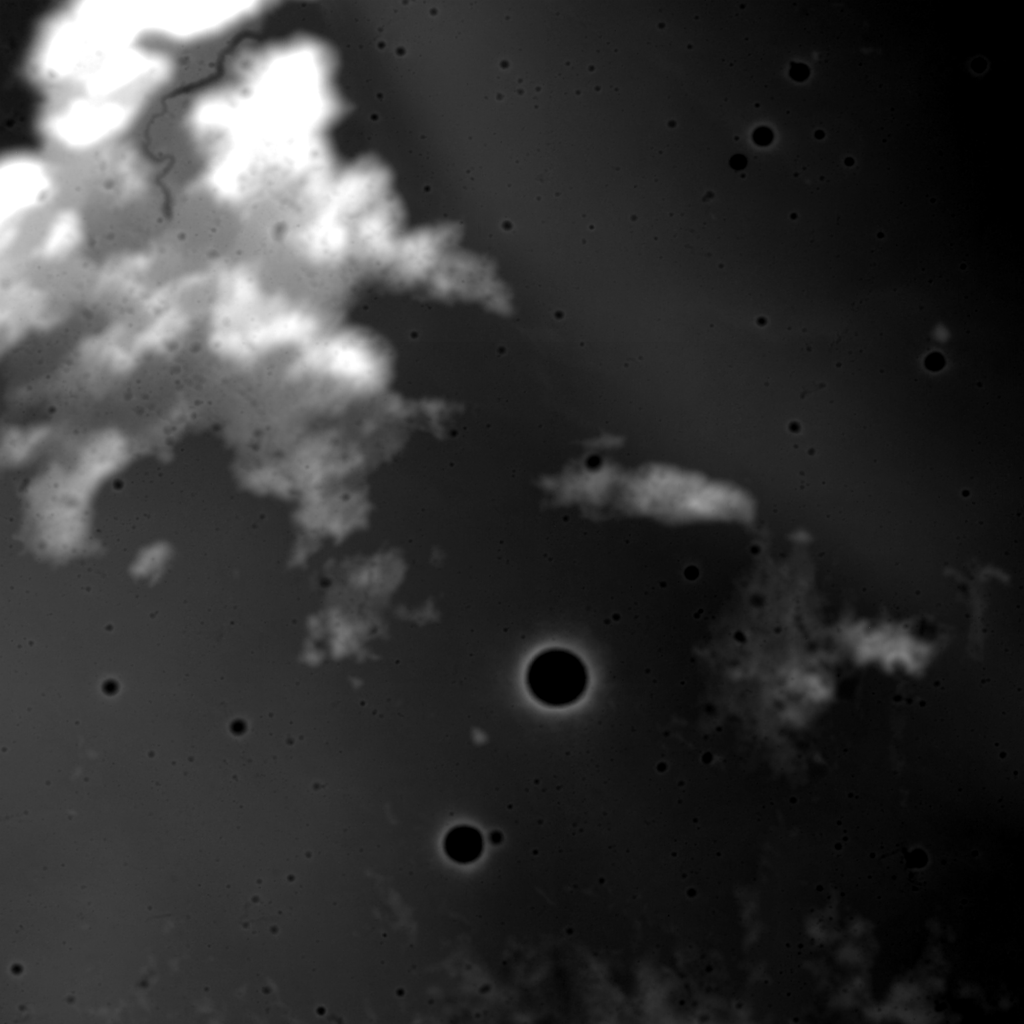

Supplement: Supplementary file 1 [file sensors-26-04344-s001.zip › data/images/test/tile_01595_lon-75.0_lat18.0.png]

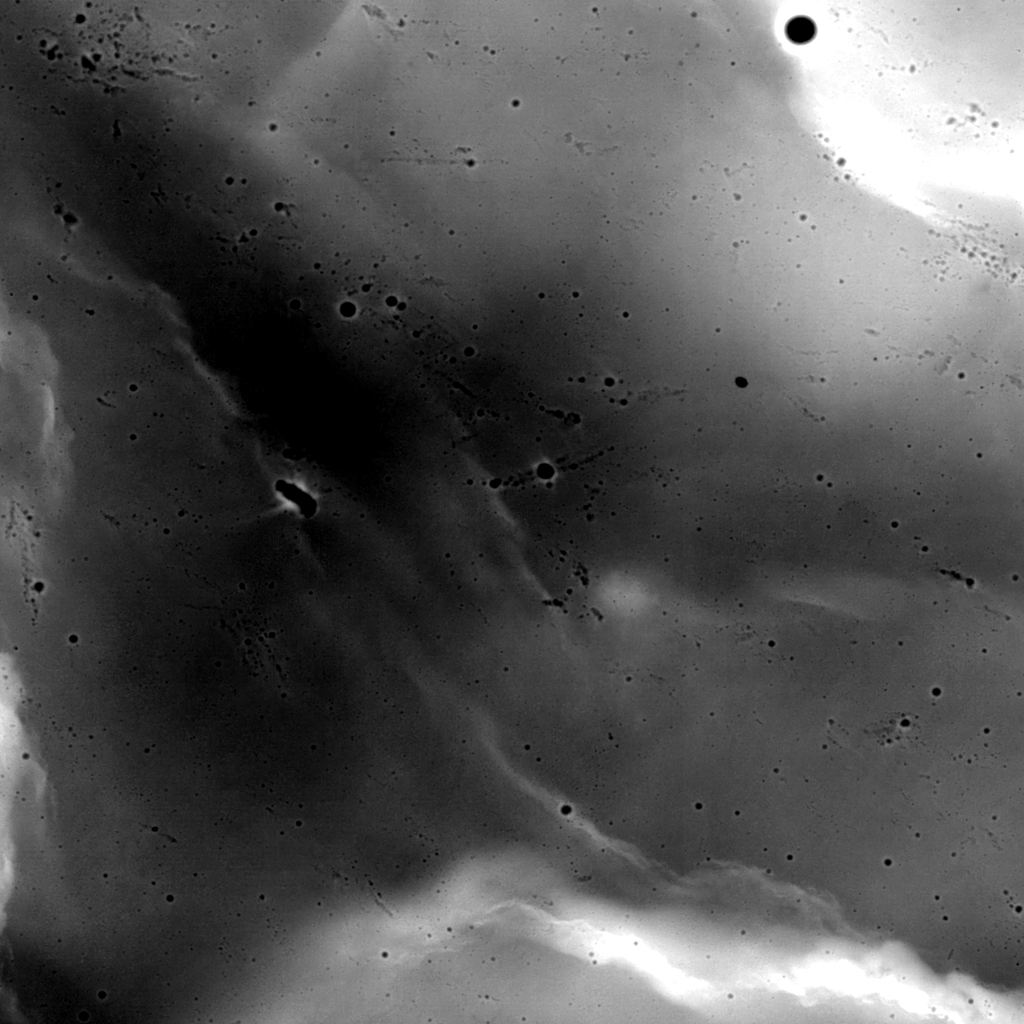

Supplement: Supplementary file 1 [file sensors-26-04344-s001.zip › data/images/test/tile_01605_lon-45.0_lat18.0.png]

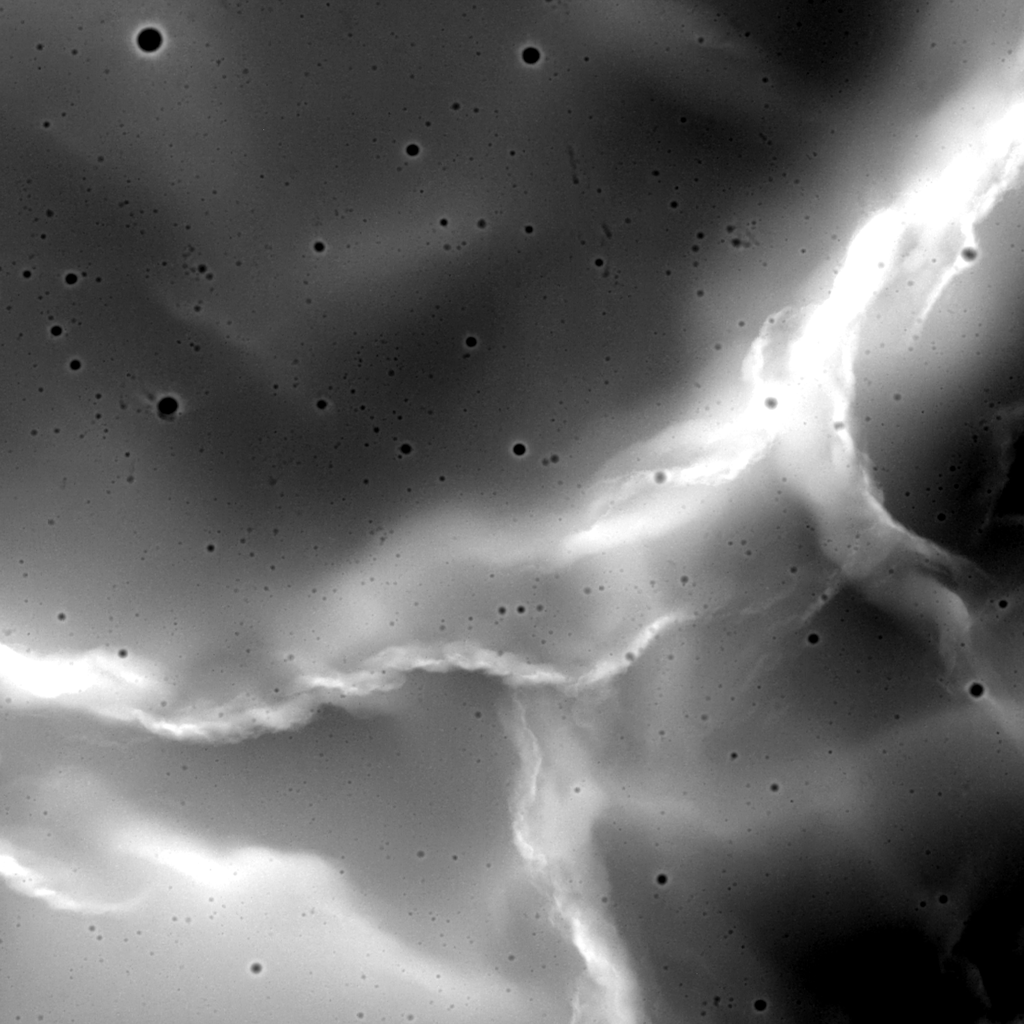

Supplement: Supplementary file 1 [file sensors-26-04344-s001.zip › data/images/test/tile_01627_lon21.0_lat18.0.png]

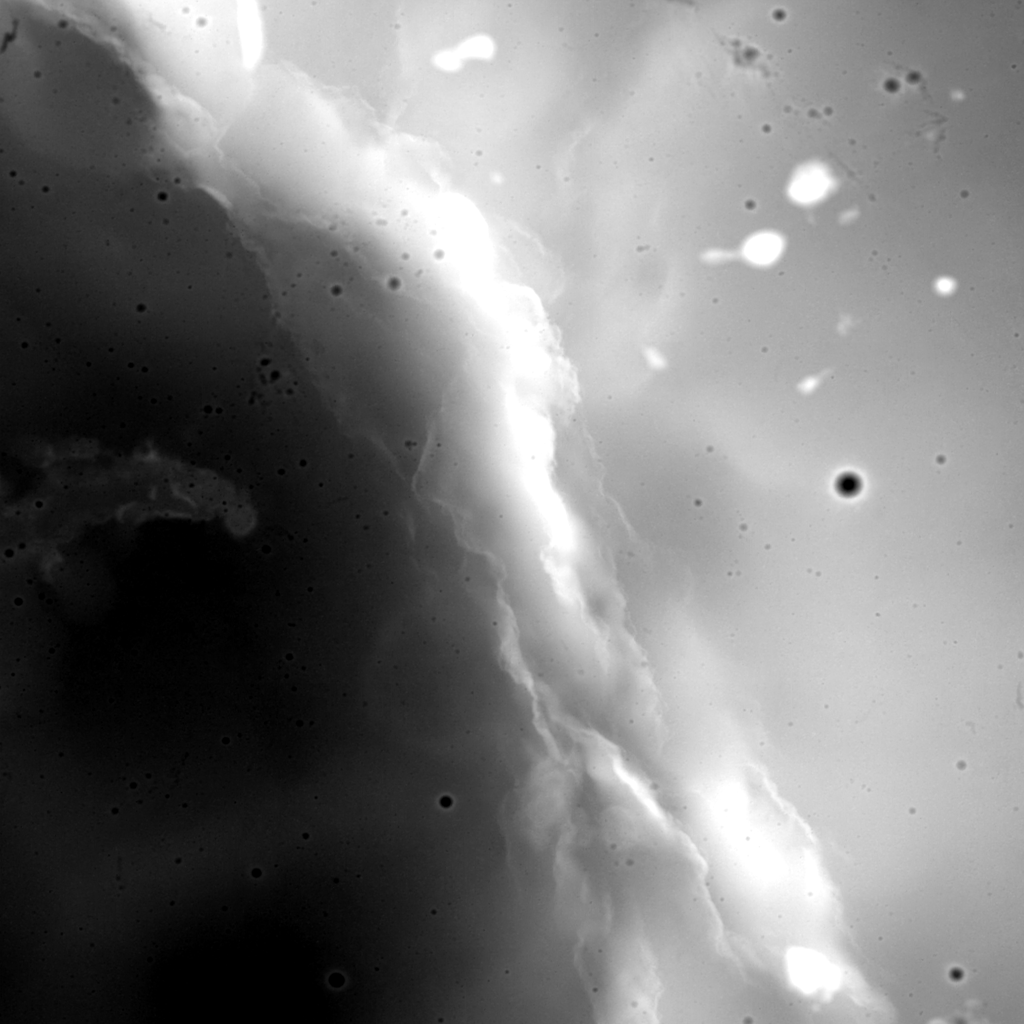

Supplement: Supplementary file 1 [file sensors-26-04344-s001.zip › data/images/test/tile_01641_lon63.0_lat18.0.png]

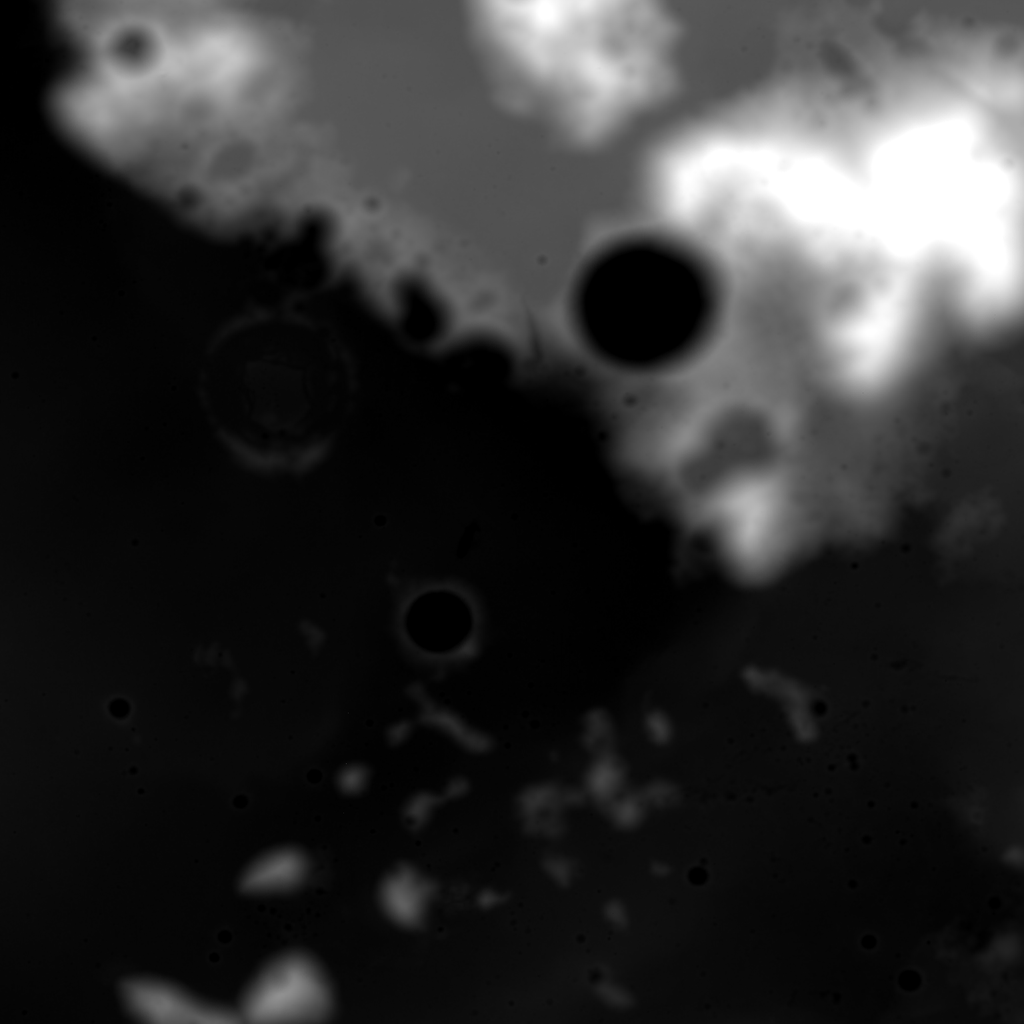

Supplement: Supplementary file 1 [file sensors-26-04344-s001.zip › data/images/test/tile_01642_lon66.0_lat18.0.png]

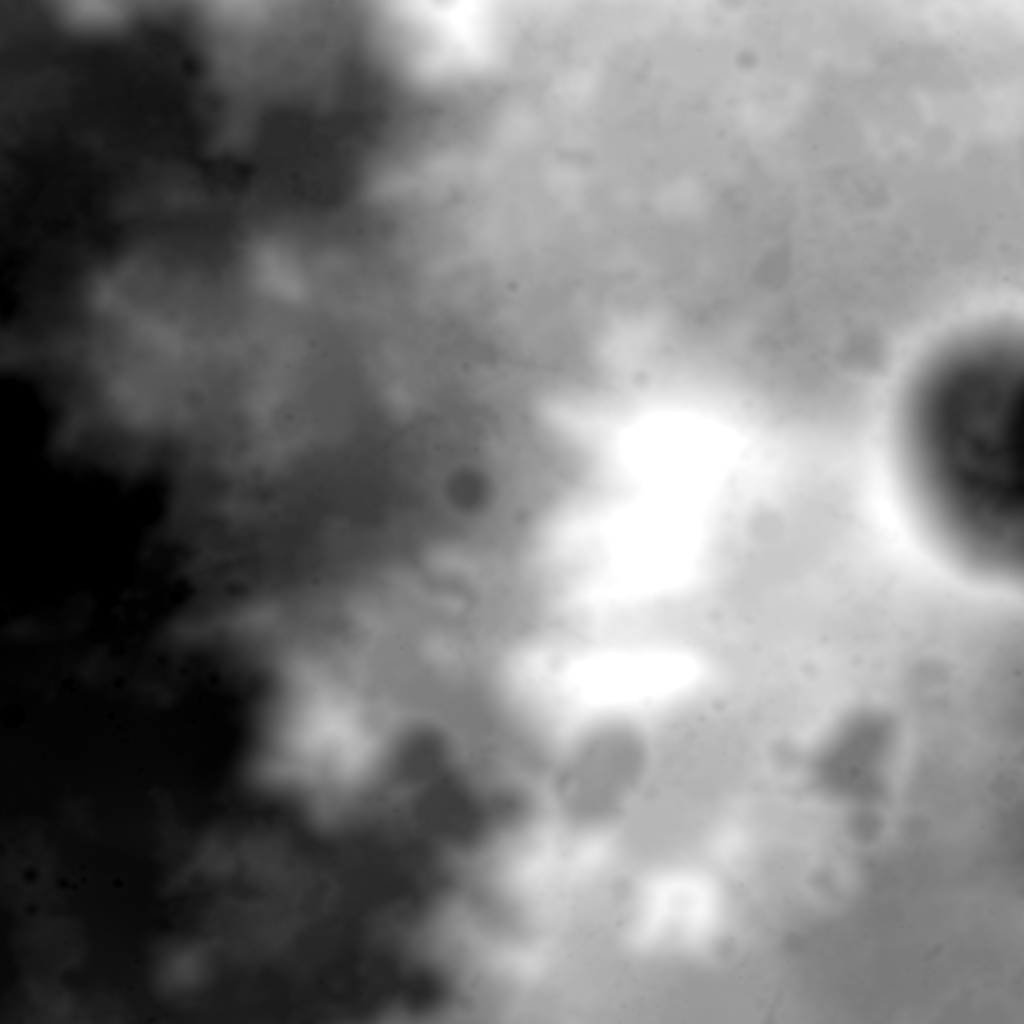

Supplement: Supplementary file 1 [file sensors-26-04344-s001.zip › data/images/test/tile_01644_lon72.0_lat18.0.png]

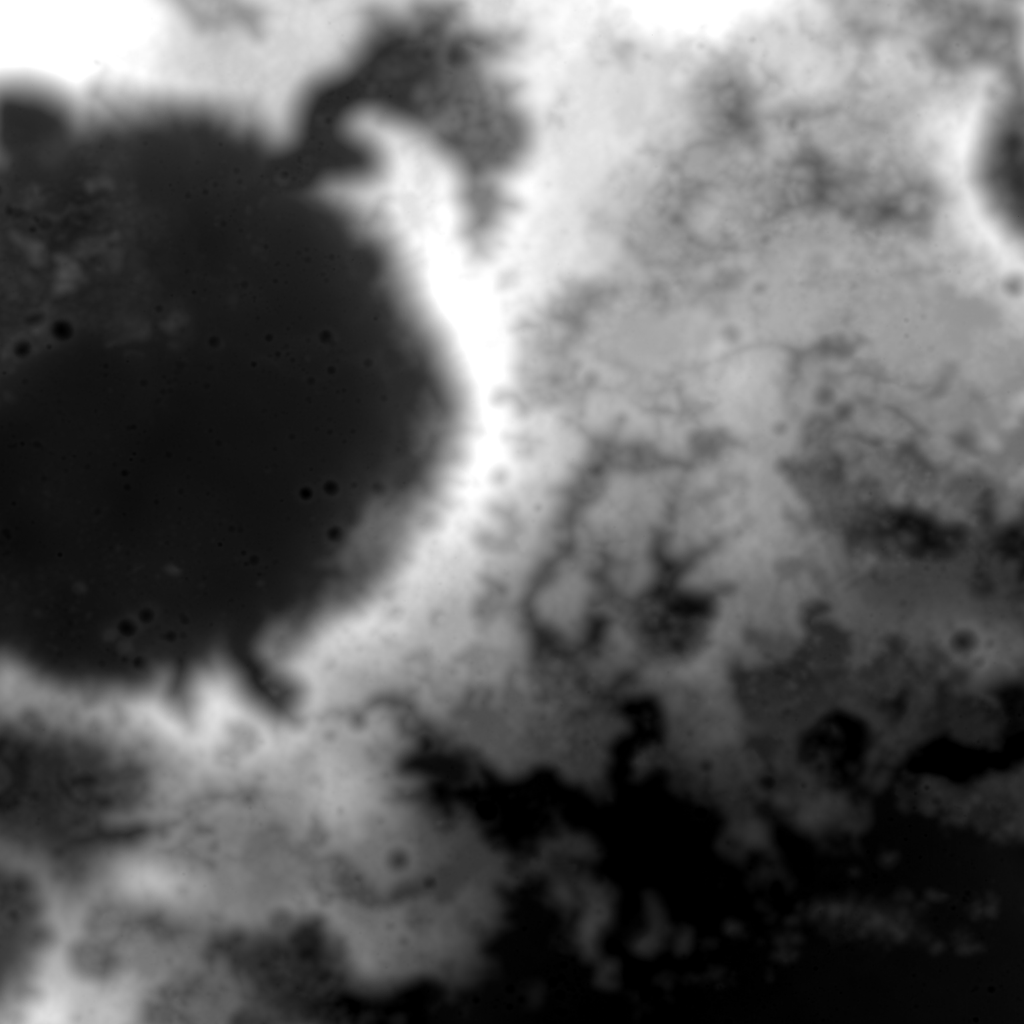

Supplement: Supplementary file 1 [file sensors-26-04344-s001.zip › data/images/test/tile_01647_lon81.0_lat18.0.png]

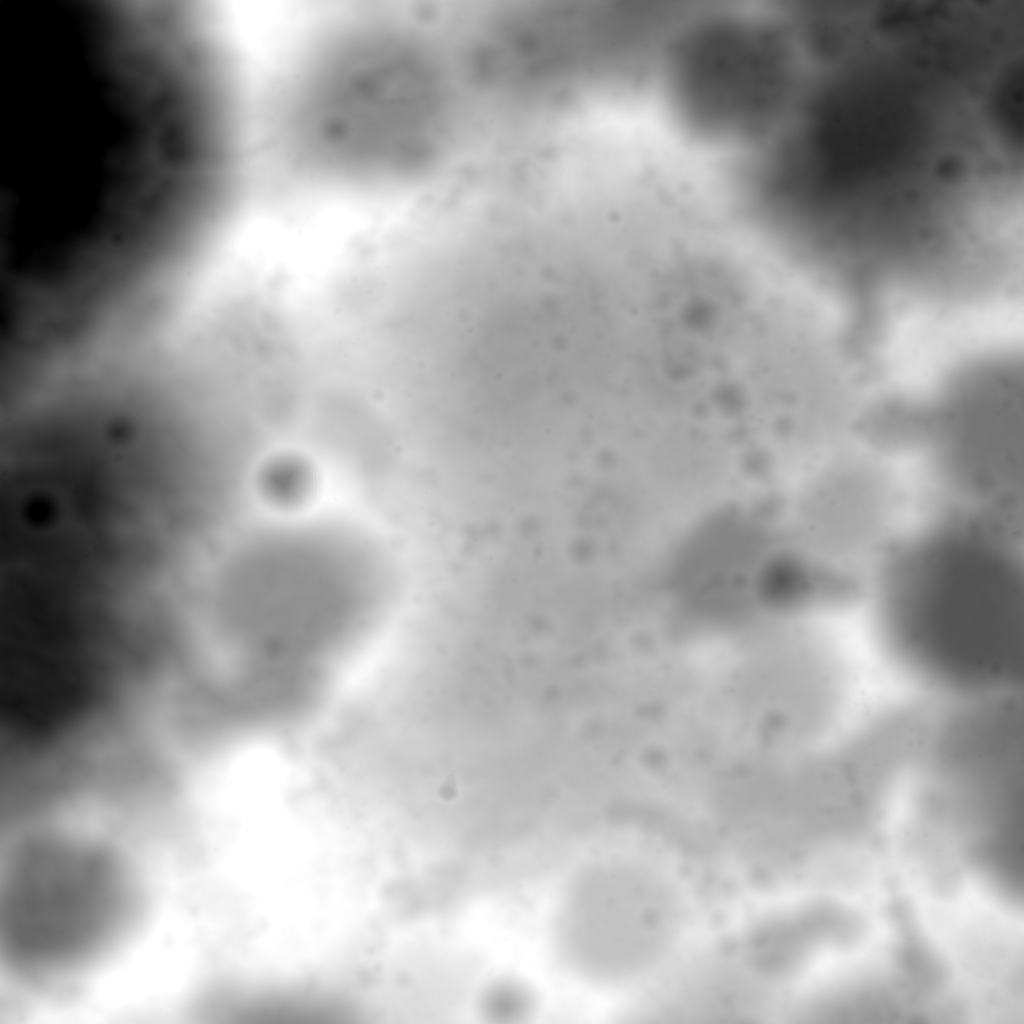

Supplement: Supplementary file 1 [file sensors-26-04344-s001.zip › data/images/test/tile_01660_lon120.0_lat18.0.png]

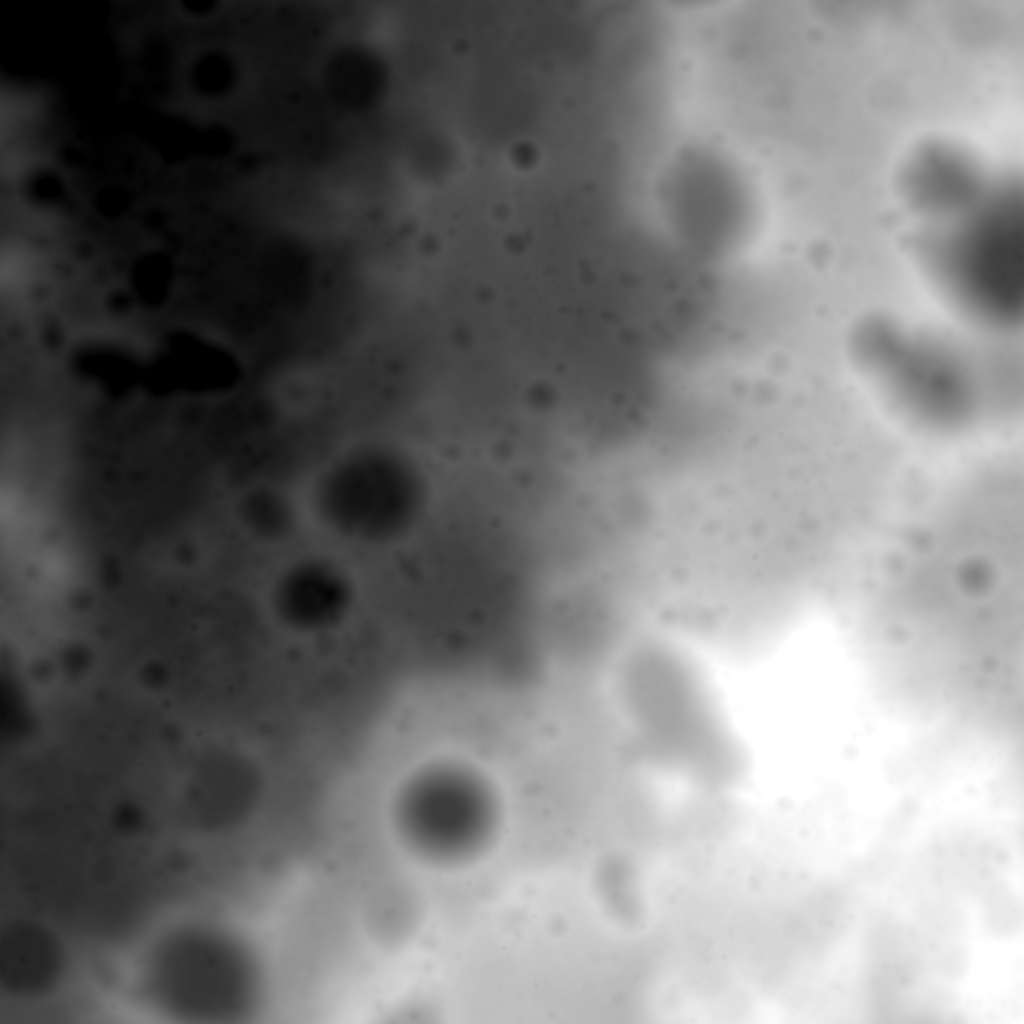

Supplement: Supplementary file 1 [file sensors-26-04344-s001.zip › data/images/test/tile_01681_lon-177.0_lat15.0.png]

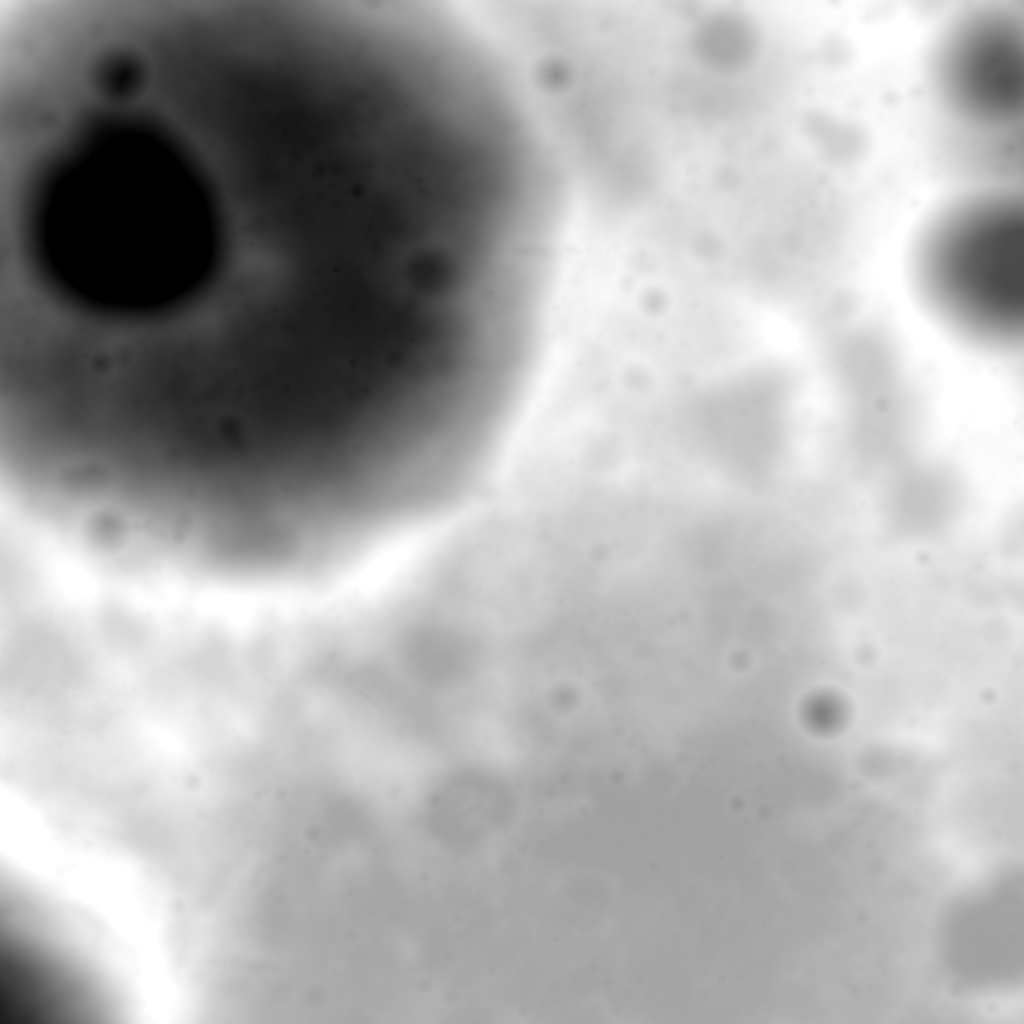

Supplement: Supplementary file 1 [file sensors-26-04344-s001.zip › data/images/test/tile_01687_lon-159.0_lat15.0.png]

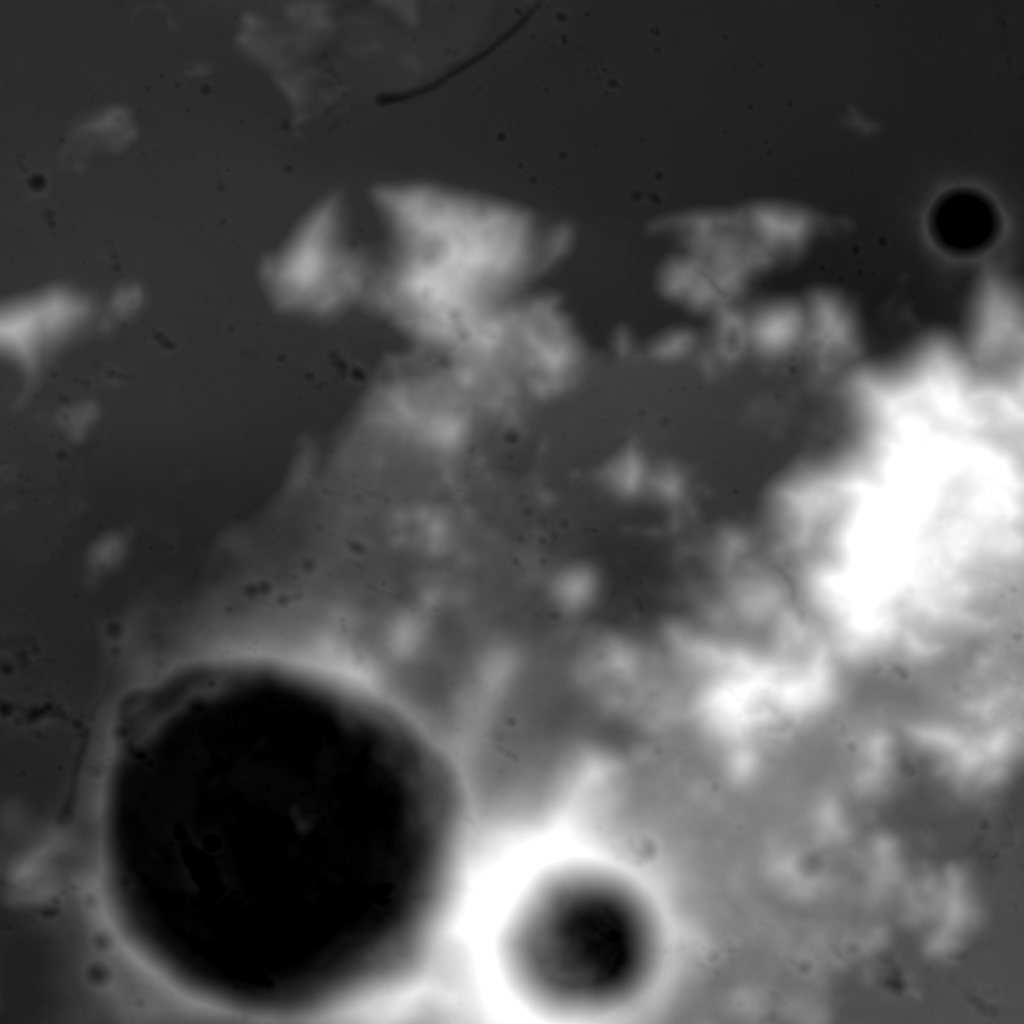

Supplement: Supplementary file 1 [file sensors-26-04344-s001.zip › data/images/test/tile_01730_lon-30.0_lat15.0.png]

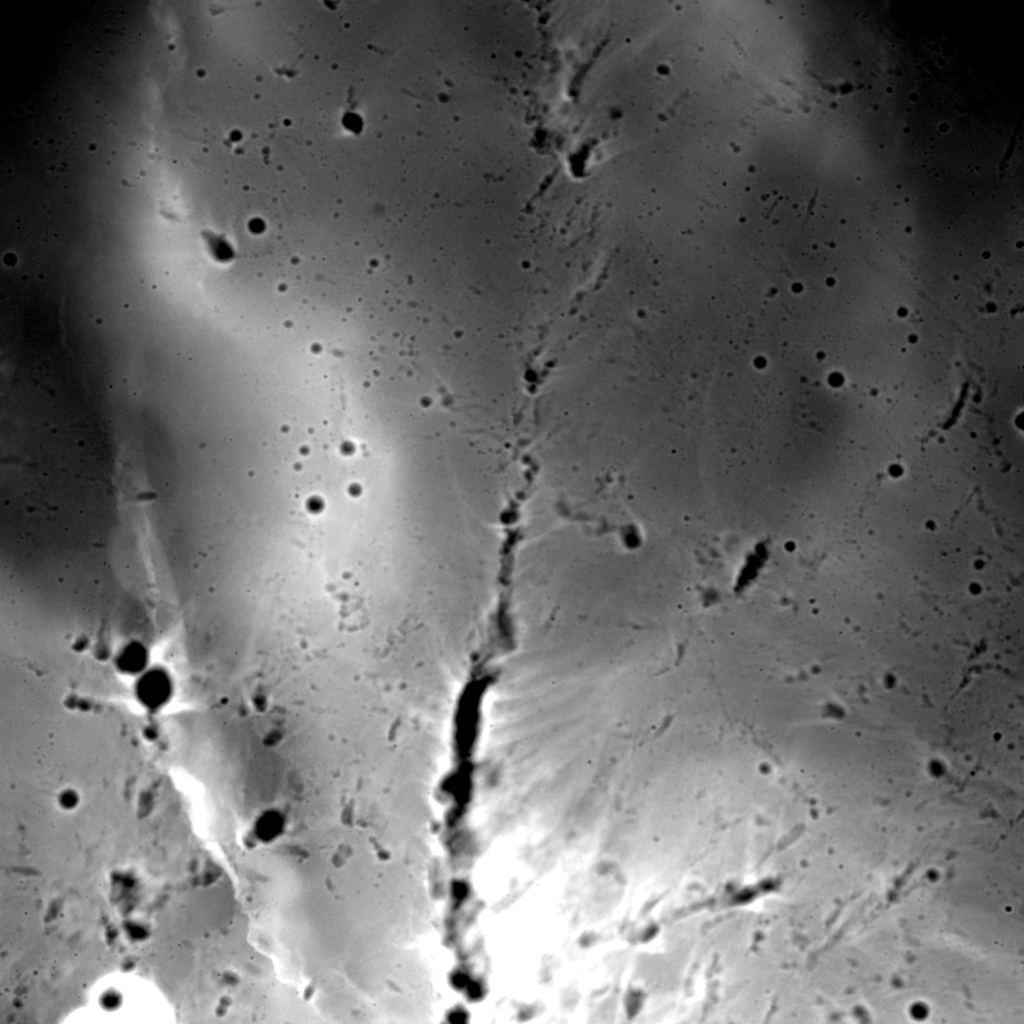

Supplement: Supplementary file 1 [file sensors-26-04344-s001.zip › data/images/test/tile_01734_lon-18.0_lat15.0.png]

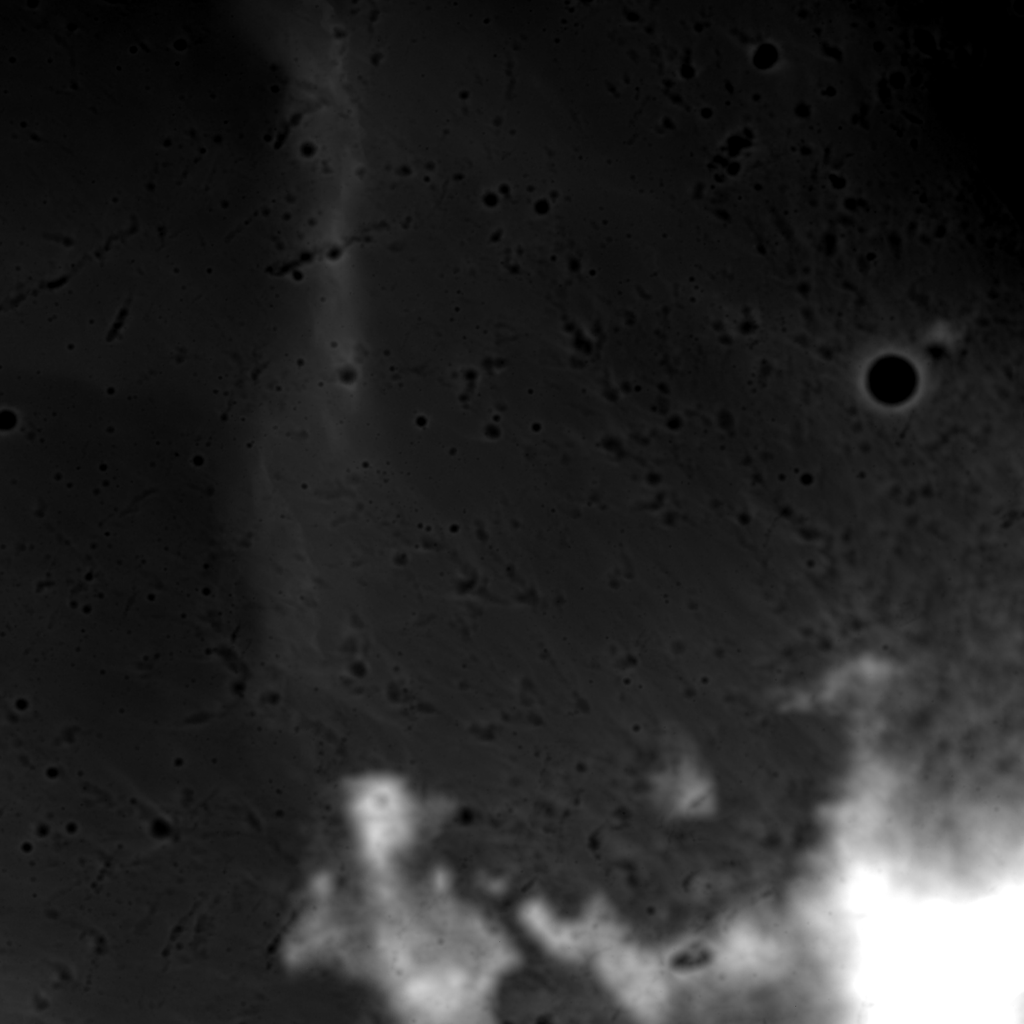

Supplement: Supplementary file 1 [file sensors-26-04344-s001.zip › data/images/test/tile_01735_lon-15.0_lat15.0.png]

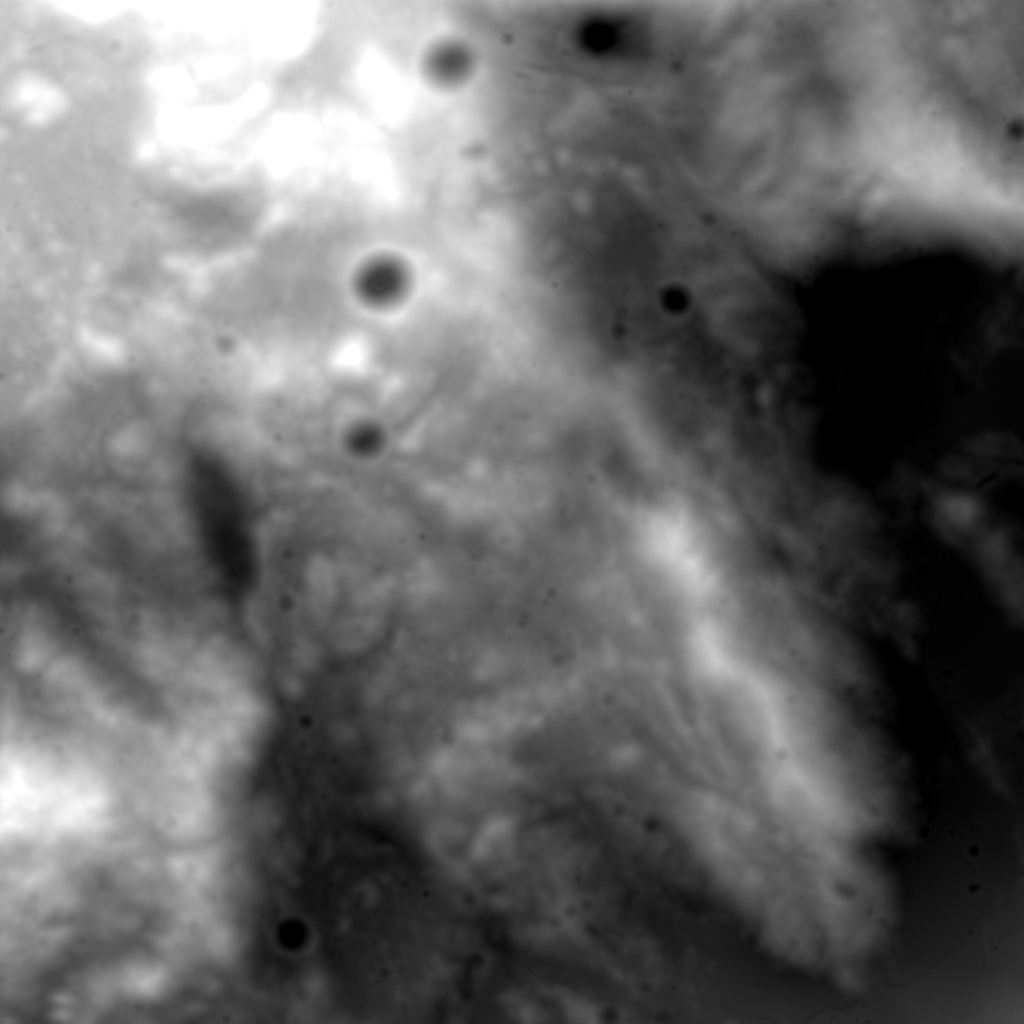

Supplement: Supplementary file 1 [file sensors-26-04344-s001.zip › data/images/test/tile_01739_lon-3.0_lat15.0.png]

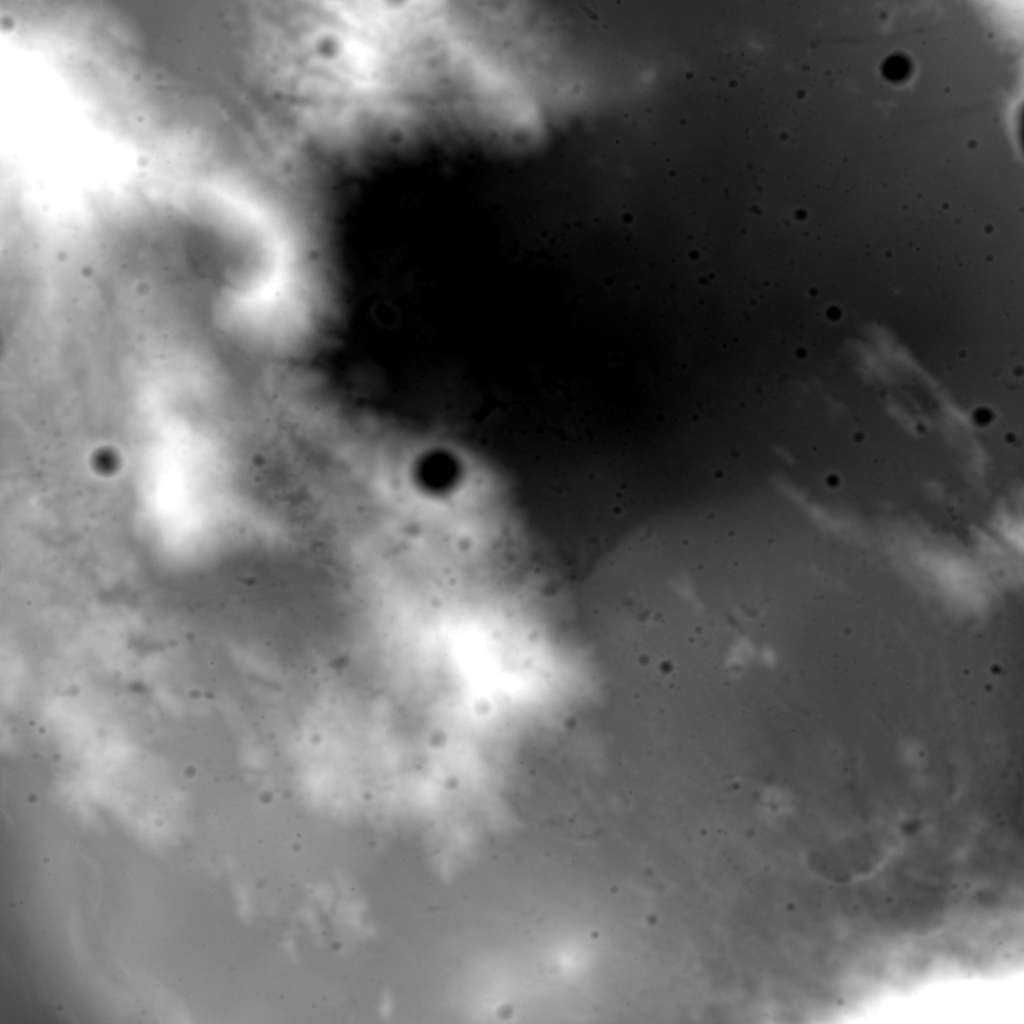

Supplement: Supplementary file 1 [file sensors-26-04344-s001.zip › data/images/test/tile_01742_lon6.0_lat15.0.png]

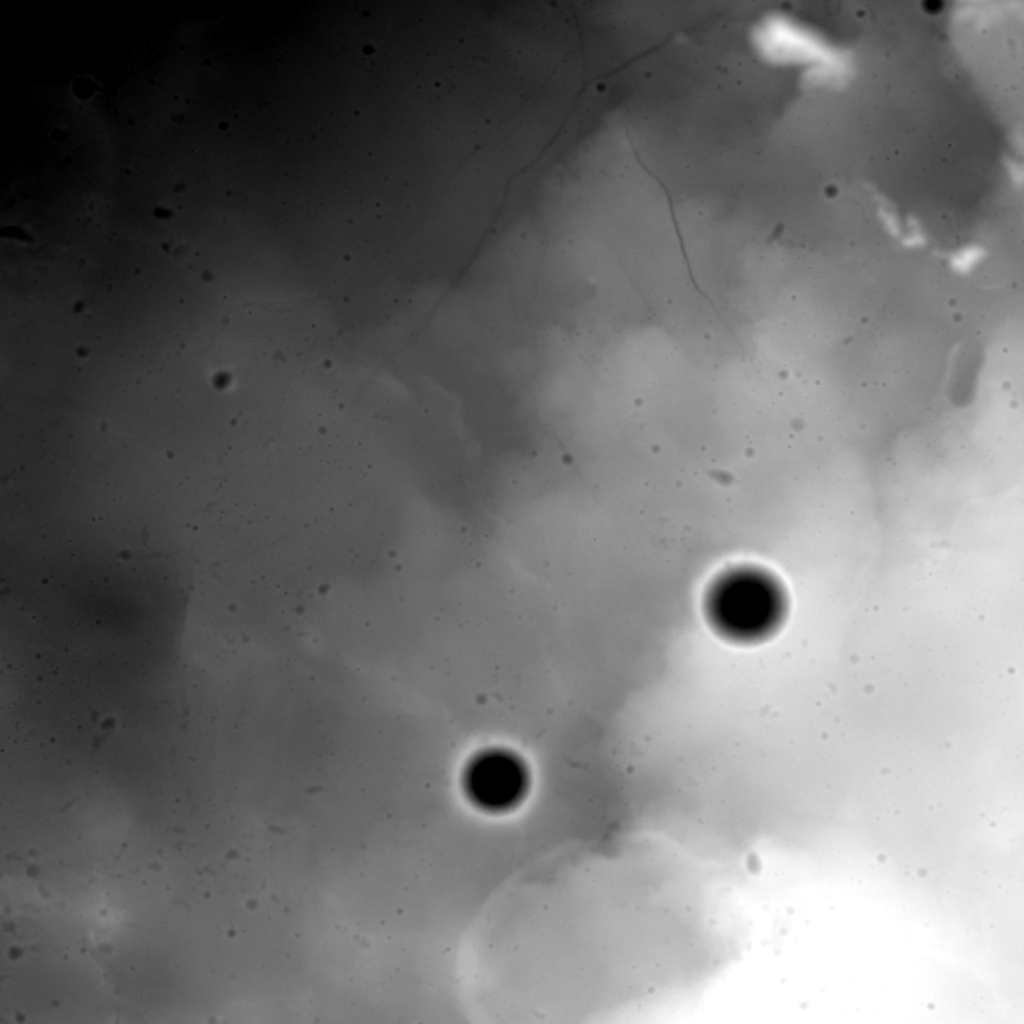

Supplement: Supplementary file 1 [file sensors-26-04344-s001.zip › data/images/test/tile_01749_lon27.0_lat15.0.png]

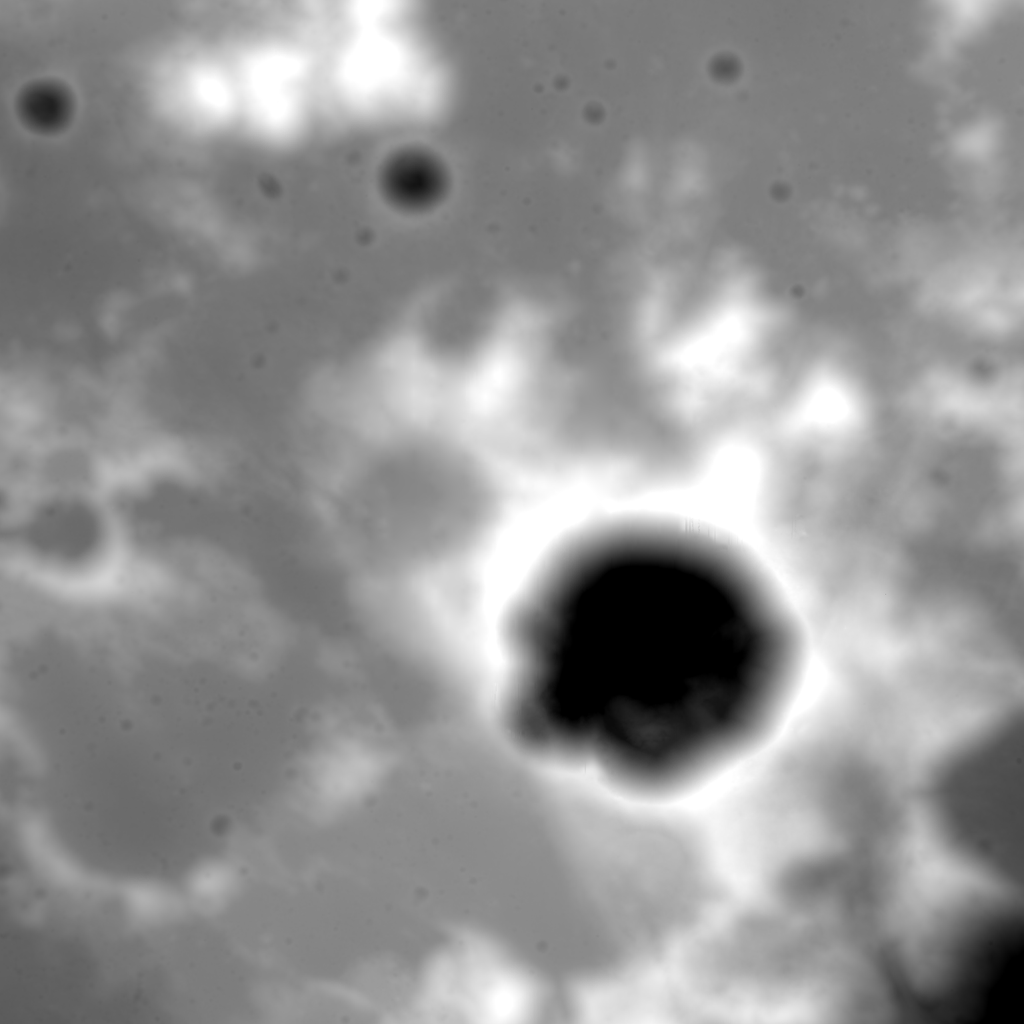

Supplement: Supplementary file 1 [file sensors-26-04344-s001.zip › data/images/test/tile_01755_lon45.0_lat15.0.png]

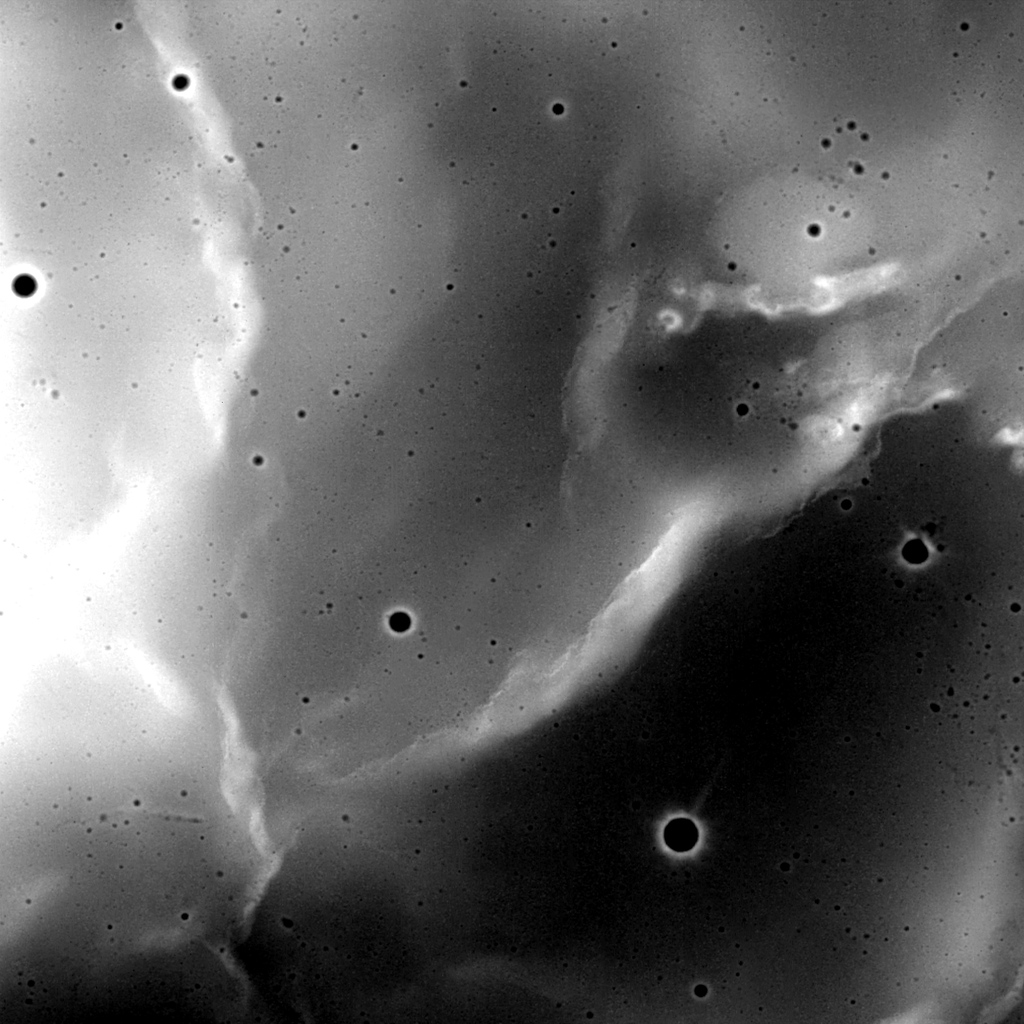

Supplement: Supplementary file 1 [file sensors-26-04344-s001.zip › data/images/test/tile_01760_lon60.0_lat15.0.png]

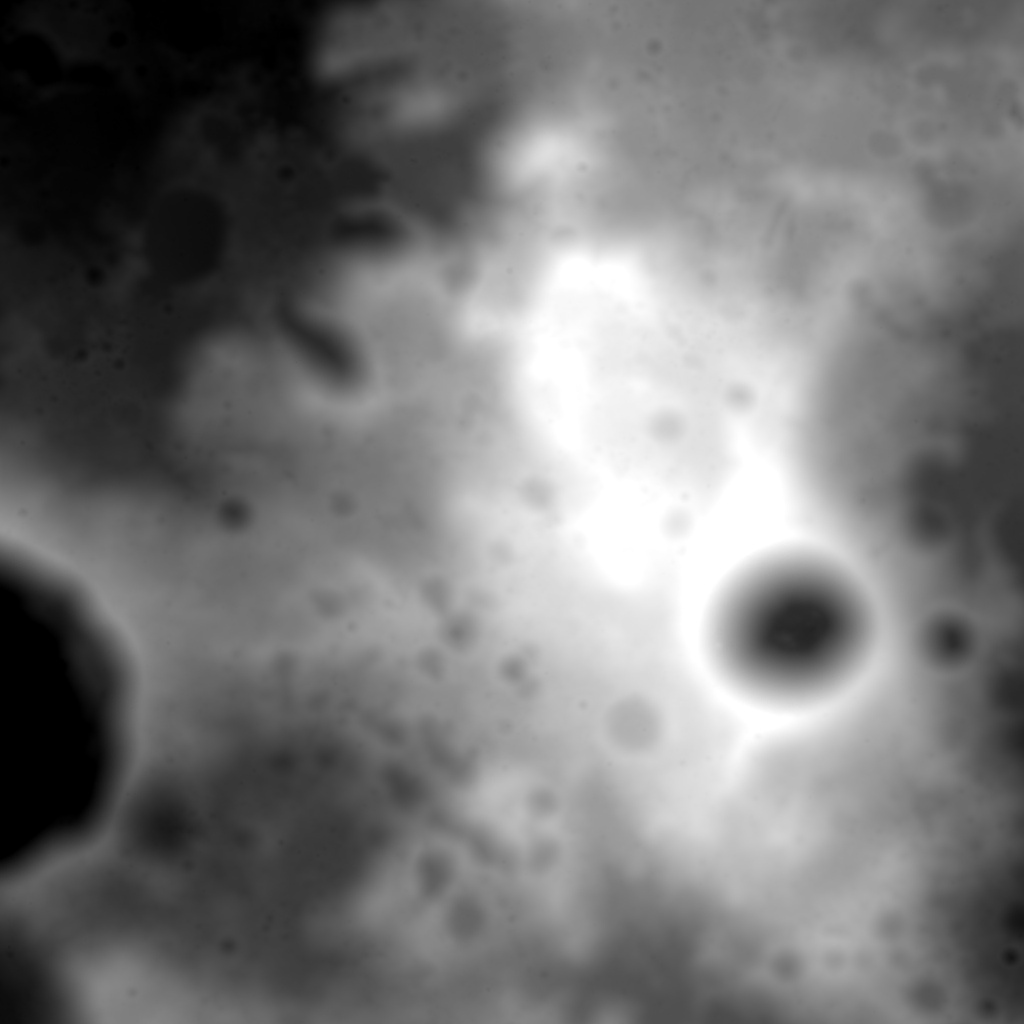

Supplement: Supplementary file 1 [file sensors-26-04344-s001.zip › data/images/test/tile_01764_lon72.0_lat15.0.png]

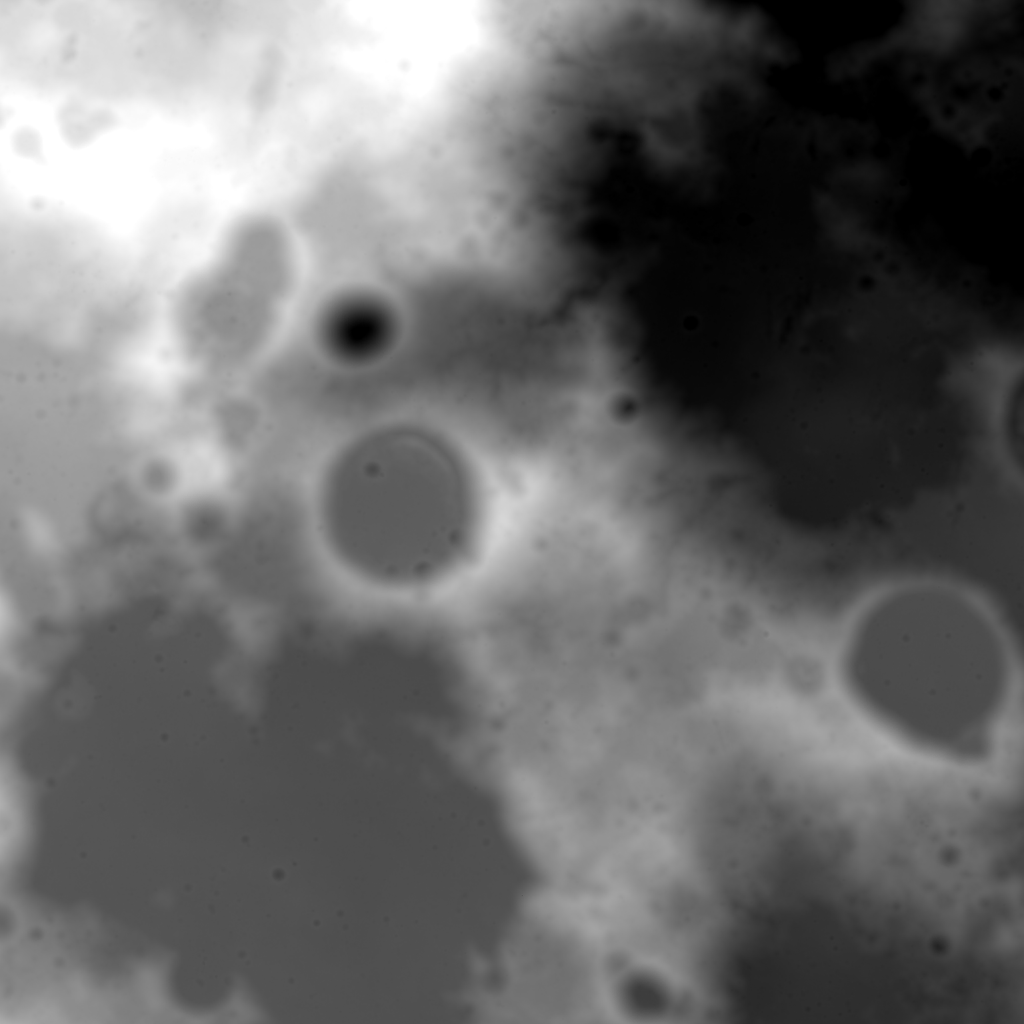

Supplement: Supplementary file 1 [file sensors-26-04344-s001.zip › data/images/test/tile_01765_lon75.0_lat15.0.png]

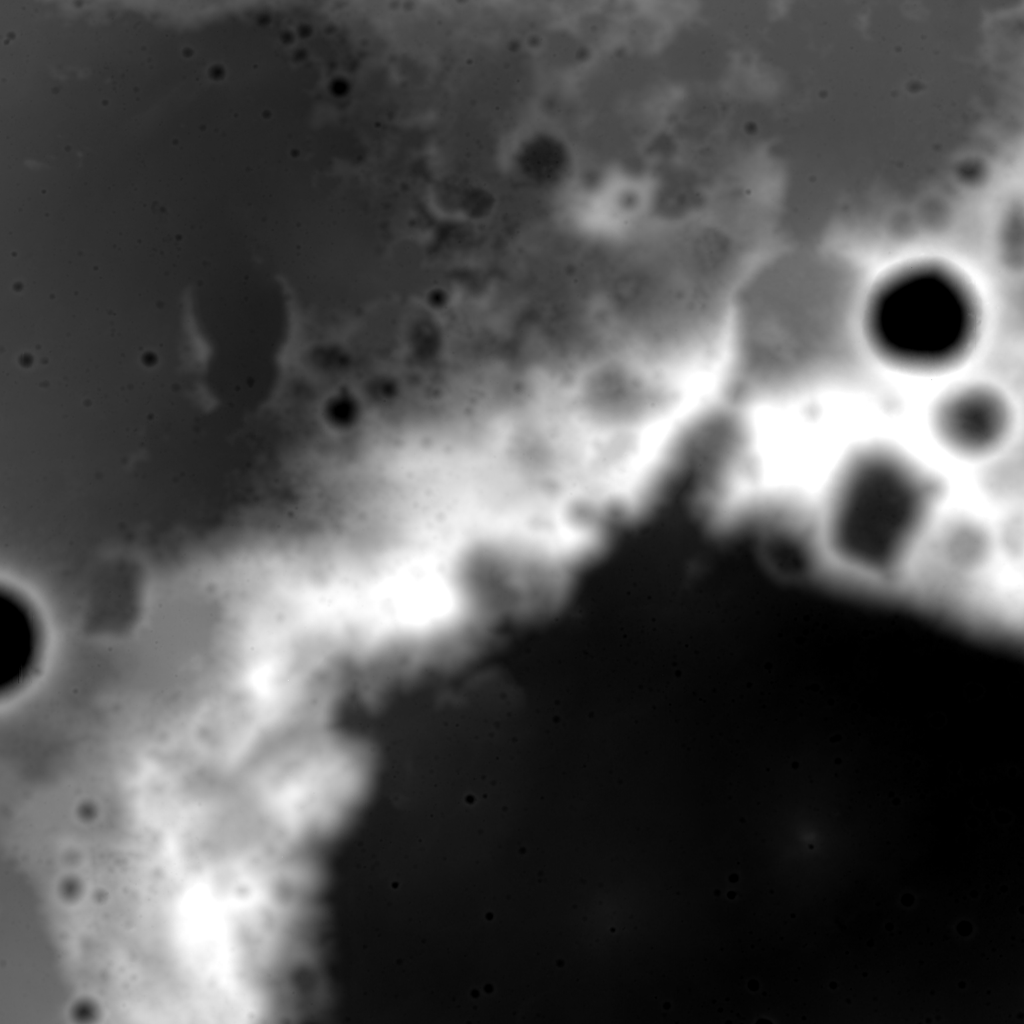

Supplement: Supplementary file 1 [file sensors-26-04344-s001.zip › data/images/test/tile_01769_lon87.0_lat15.0.png]

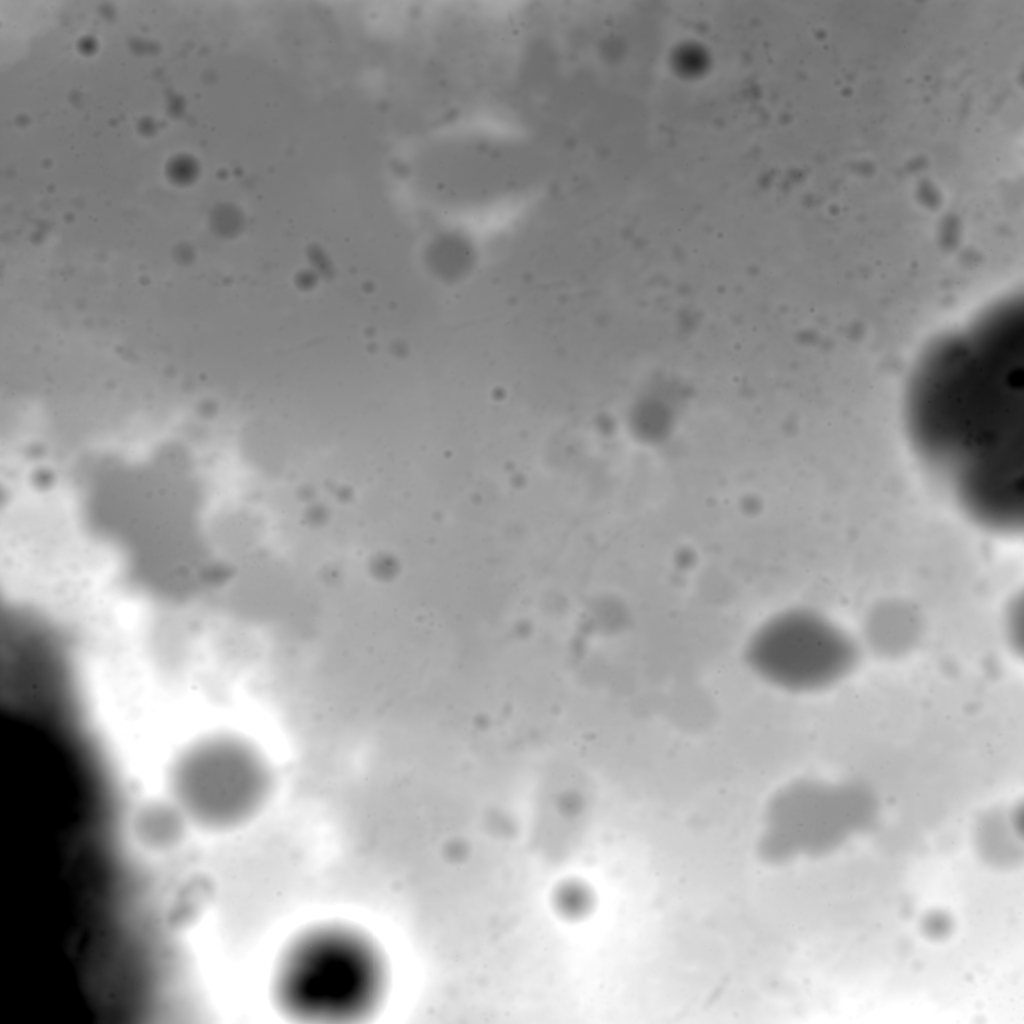

Supplement: Supplementary file 1 [file sensors-26-04344-s001.zip › data/images/test/tile_01777_lon111.0_lat15.0.png]

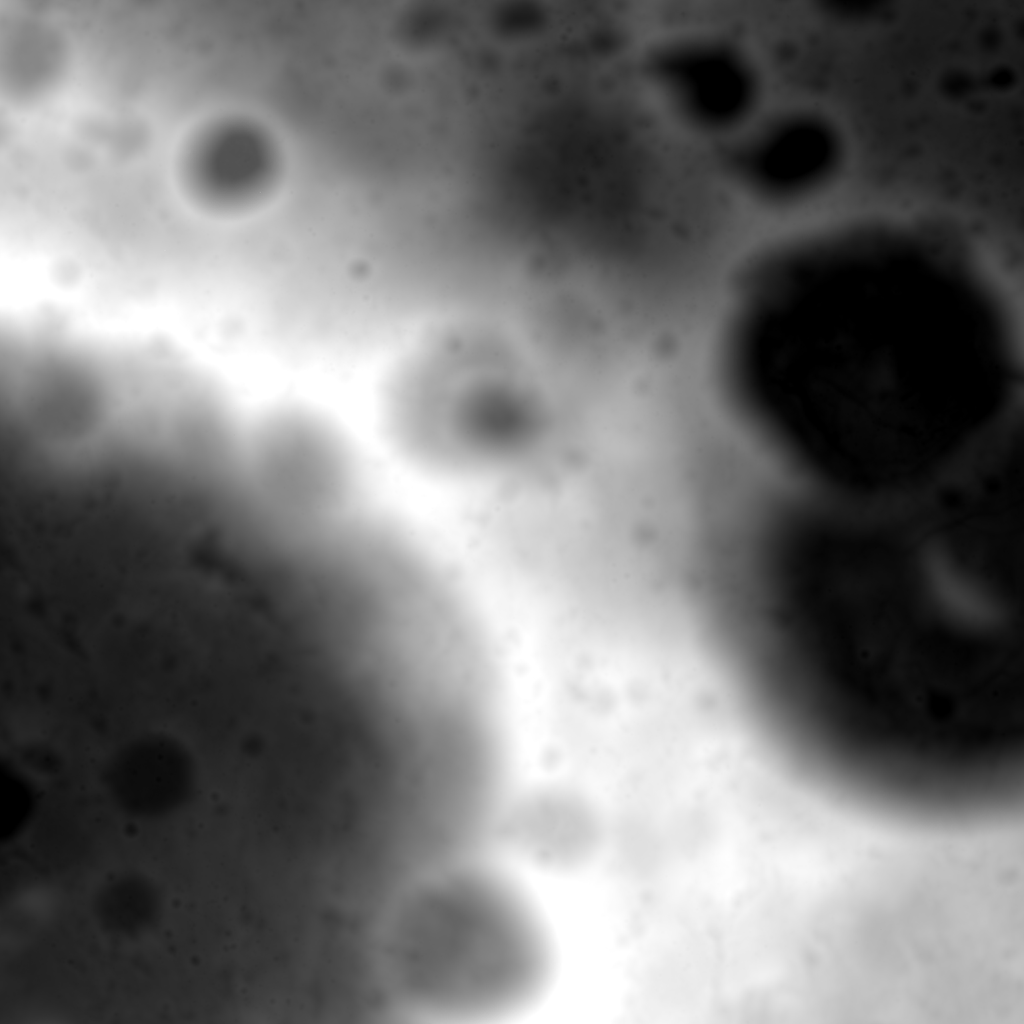

Supplement: Supplementary file 1 [file sensors-26-04344-s001.zip › data/images/test/tile_01797_lon171.0_lat15.0.png]

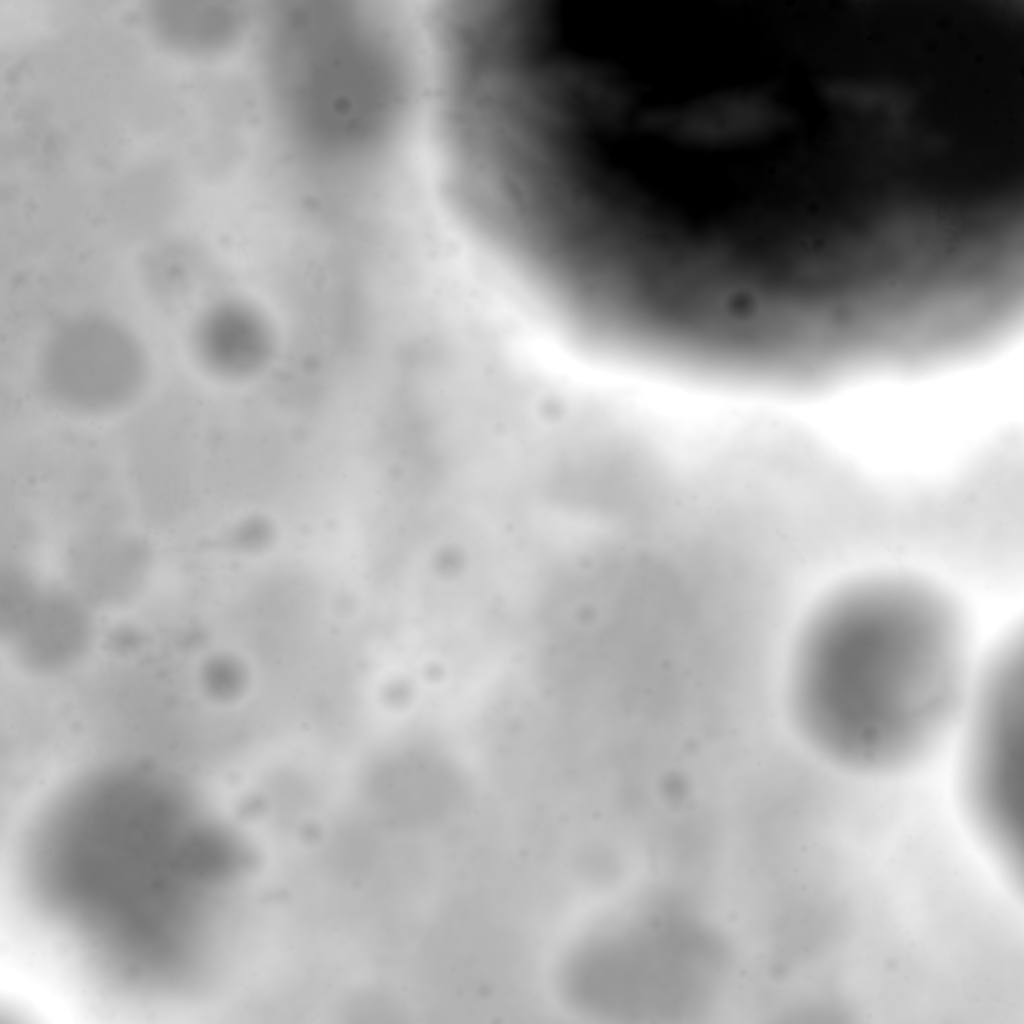

Supplement: Supplementary file 1 [file sensors-26-04344-s001.zip › data/images/test/tile_01806_lon-162.0_lat12.0.png]

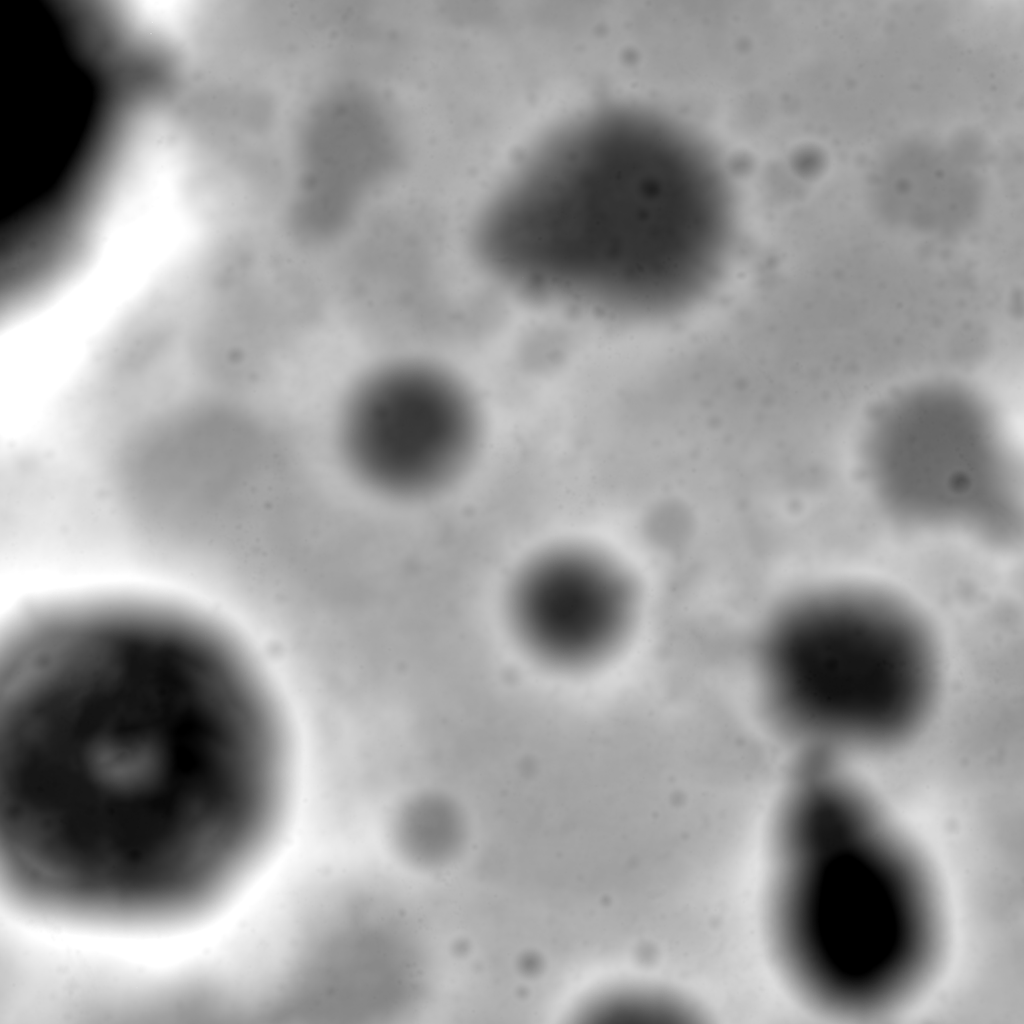

Supplement: Supplementary file 1 [file sensors-26-04344-s001.zip › data/images/test/tile_01807_lon-159.0_lat12.0.png]

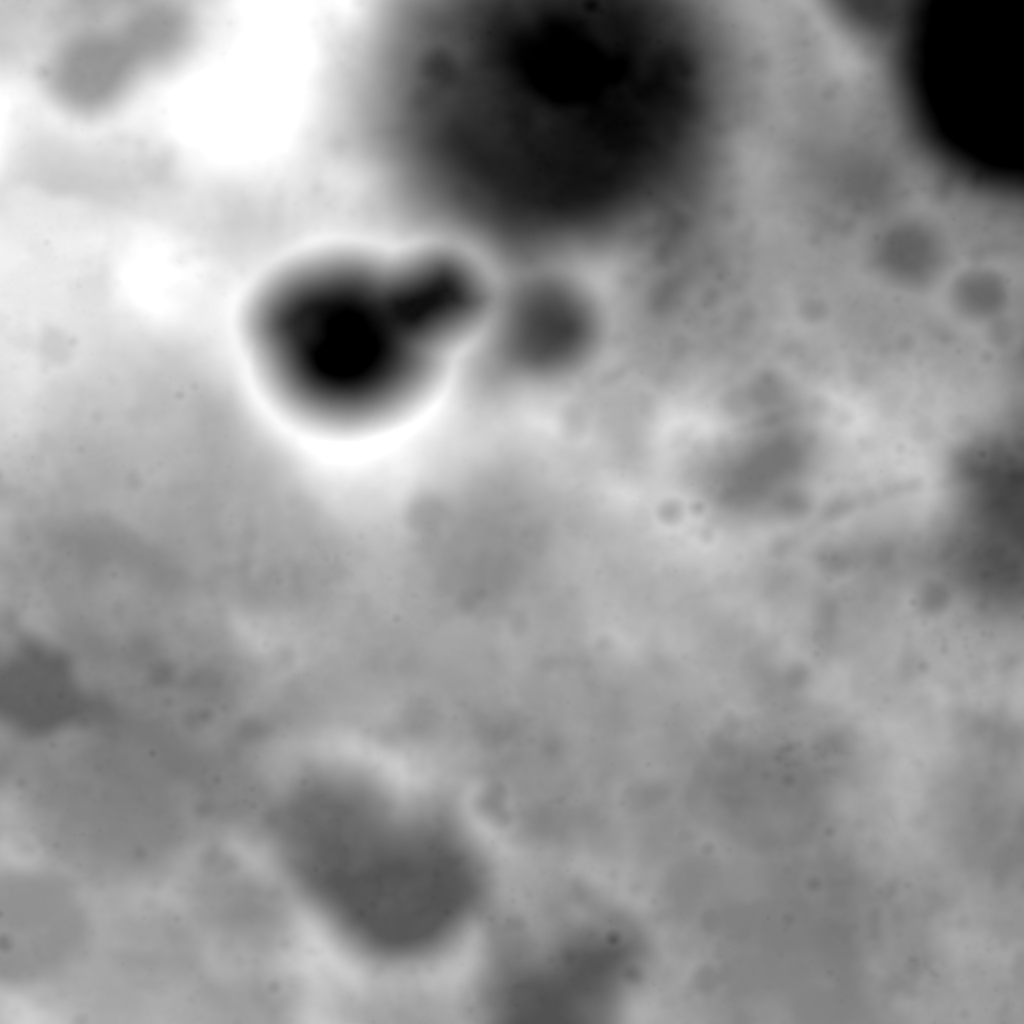

Supplement: Supplementary file 1 [file sensors-26-04344-s001.zip › data/images/test/tile_01811_lon-147.0_lat12.0.png]

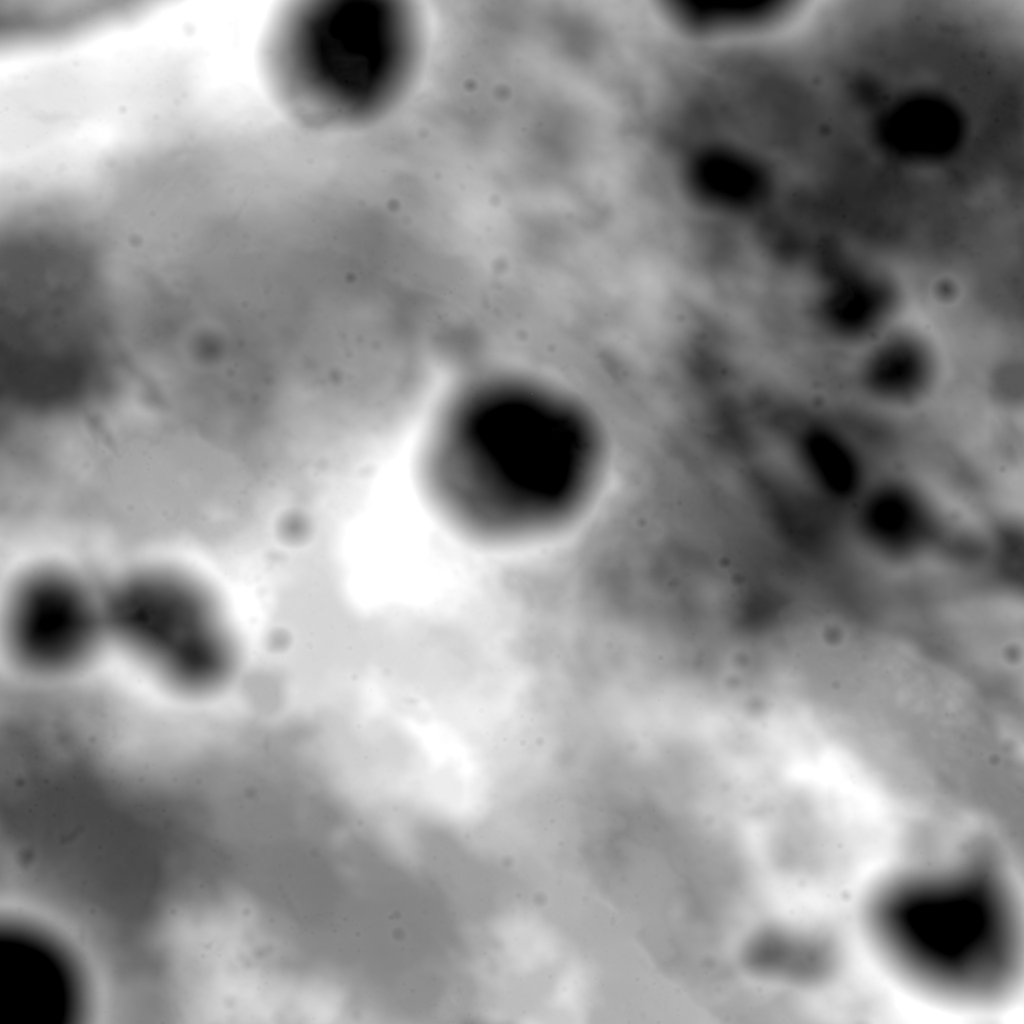

Supplement: Supplementary file 1 [file sensors-26-04344-s001.zip › data/images/test/tile_01814_lon-138.0_lat12.0.png]

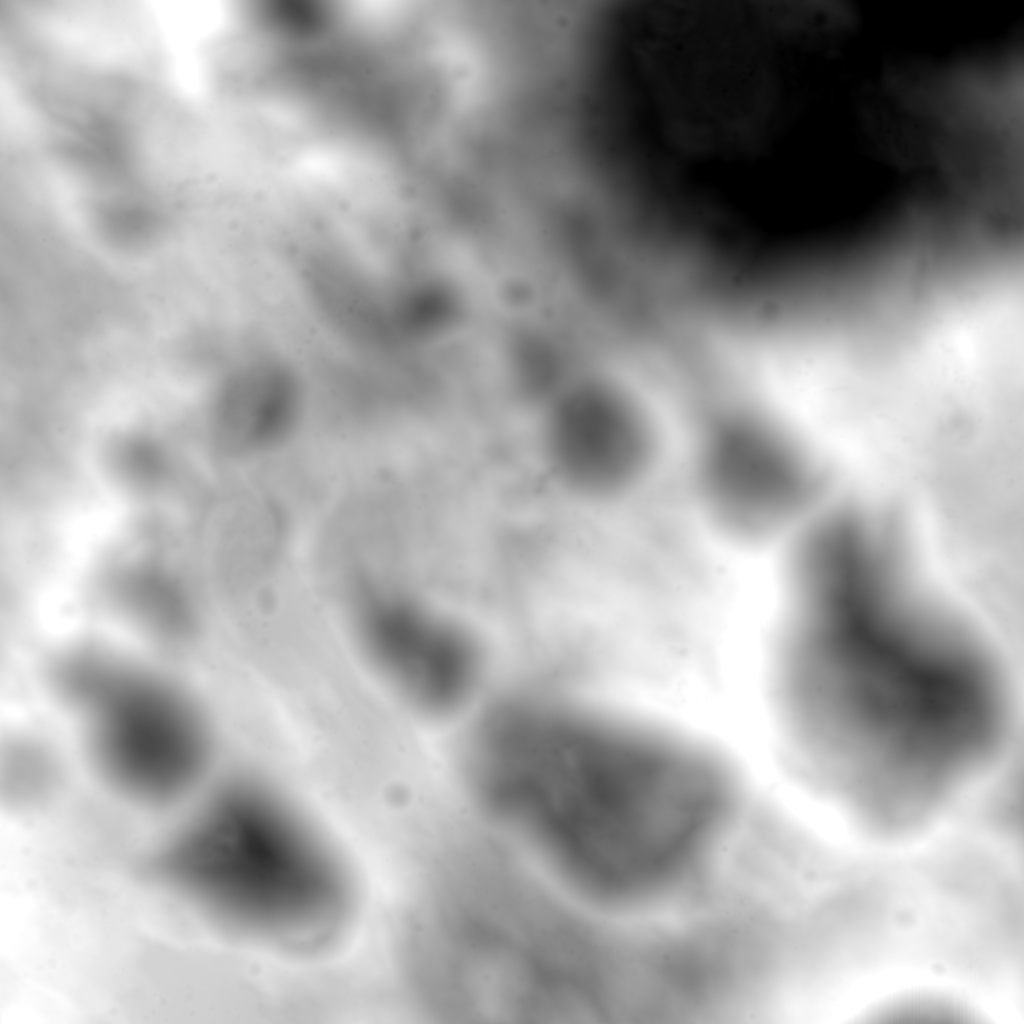

Supplement: Supplementary file 1 [file sensors-26-04344-s001.zip › data/images/test/tile_01819_lon-123.0_lat12.0.png]

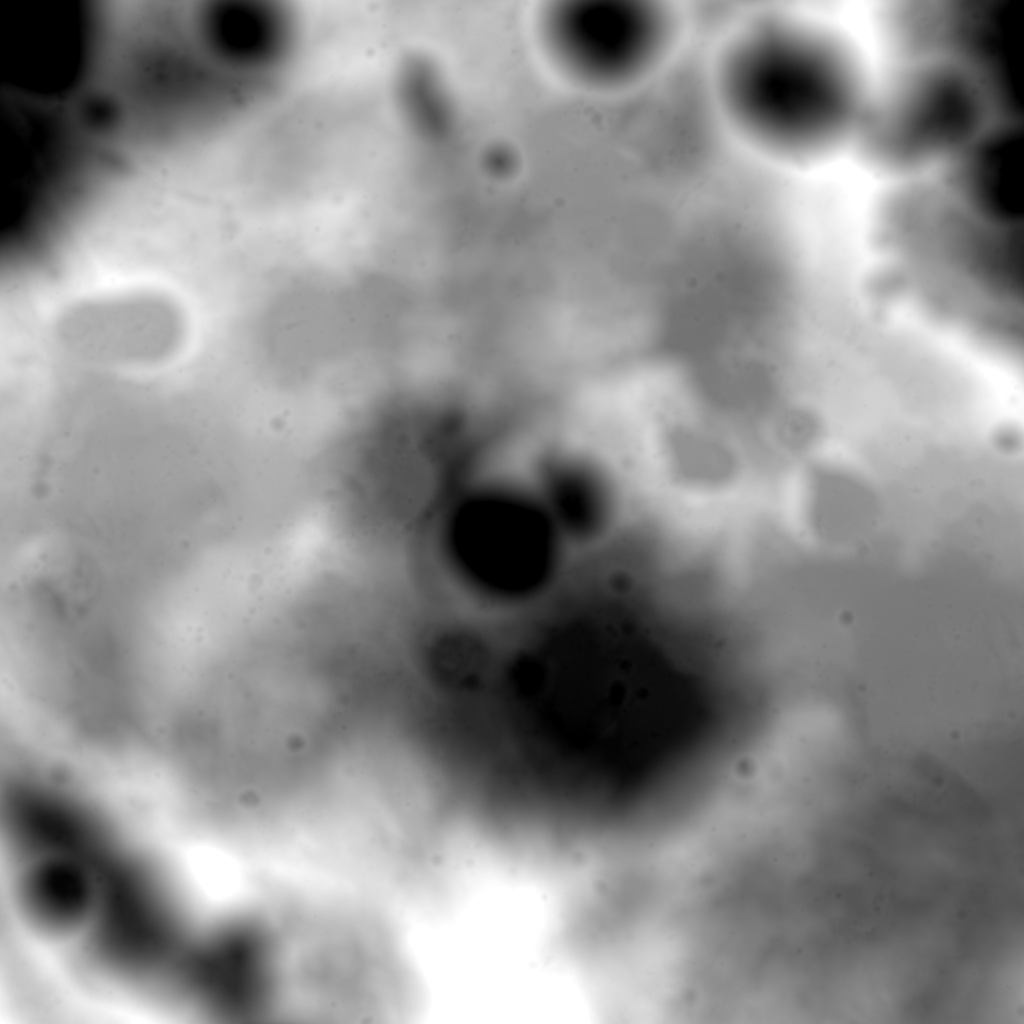

Supplement: Supplementary file 1 [file sensors-26-04344-s001.zip › data/images/test/tile_01820_lon-120.0_lat12.0.png]

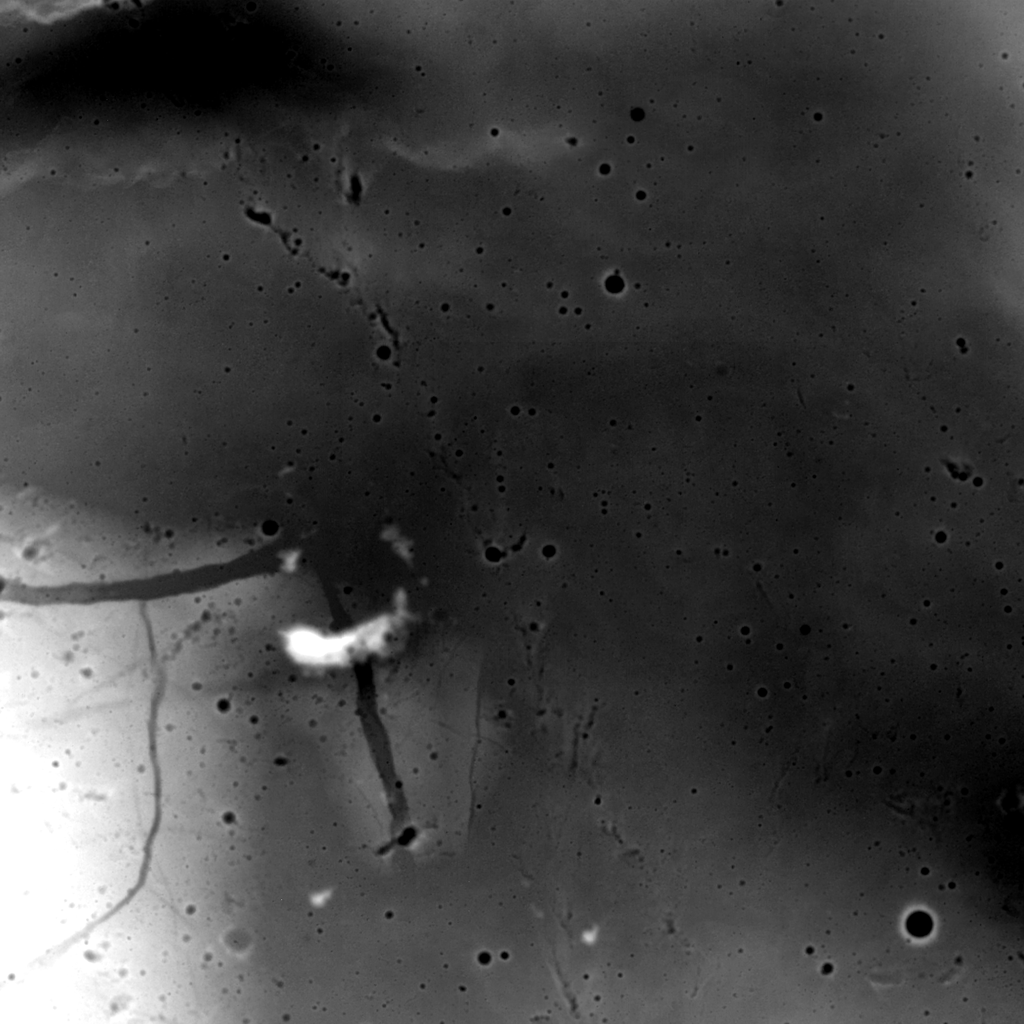

Supplement: Supplementary file 1 [file sensors-26-04344-s001.zip › data/images/test/tile_01837_lon-69.0_lat12.0.png]

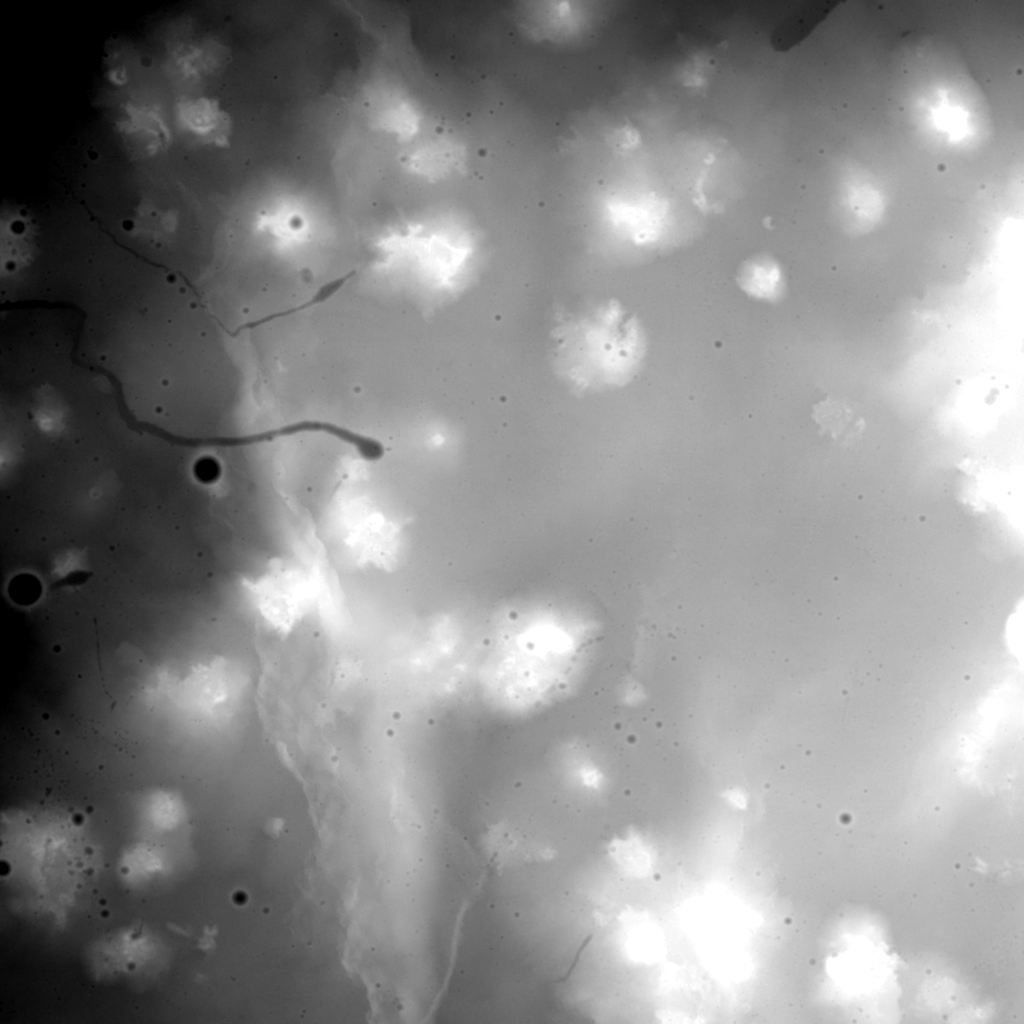

Supplement: Supplementary file 1 [file sensors-26-04344-s001.zip › data/images/test/tile_01841_lon-57.0_lat12.0.png]

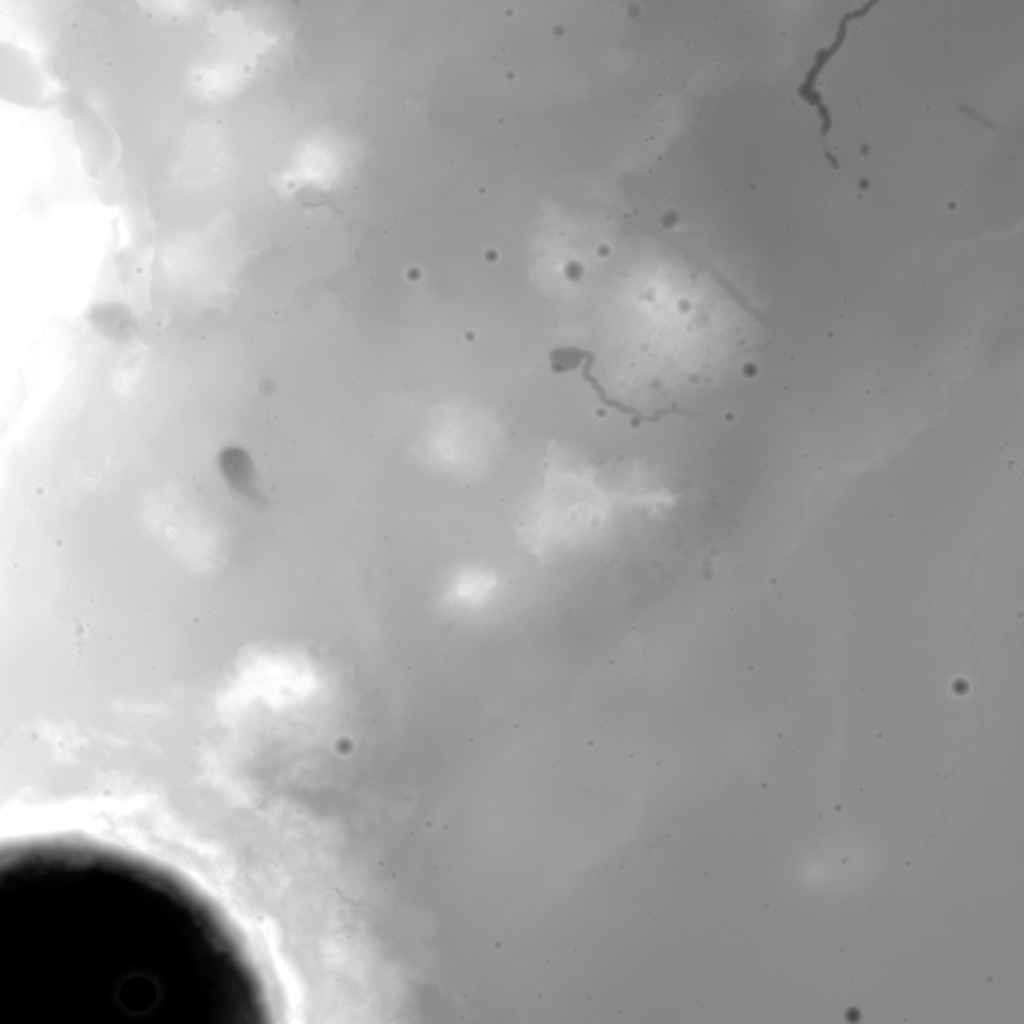

Supplement: Supplementary file 1 [file sensors-26-04344-s001.zip › data/images/test/tile_01843_lon-51.0_lat12.0.png]

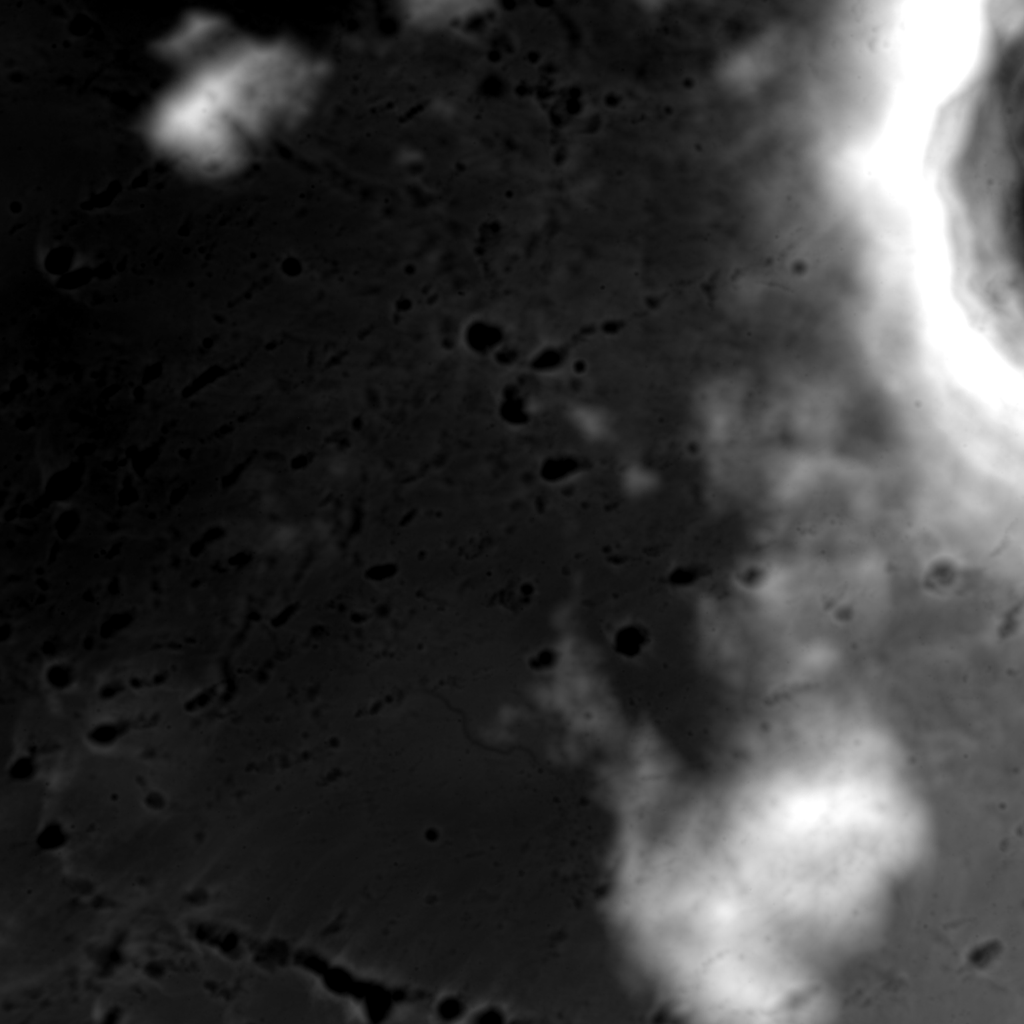

Supplement: Supplementary file 1 [file sensors-26-04344-s001.zip › data/images/test/tile_01855_lon-15.0_lat12.0.png]

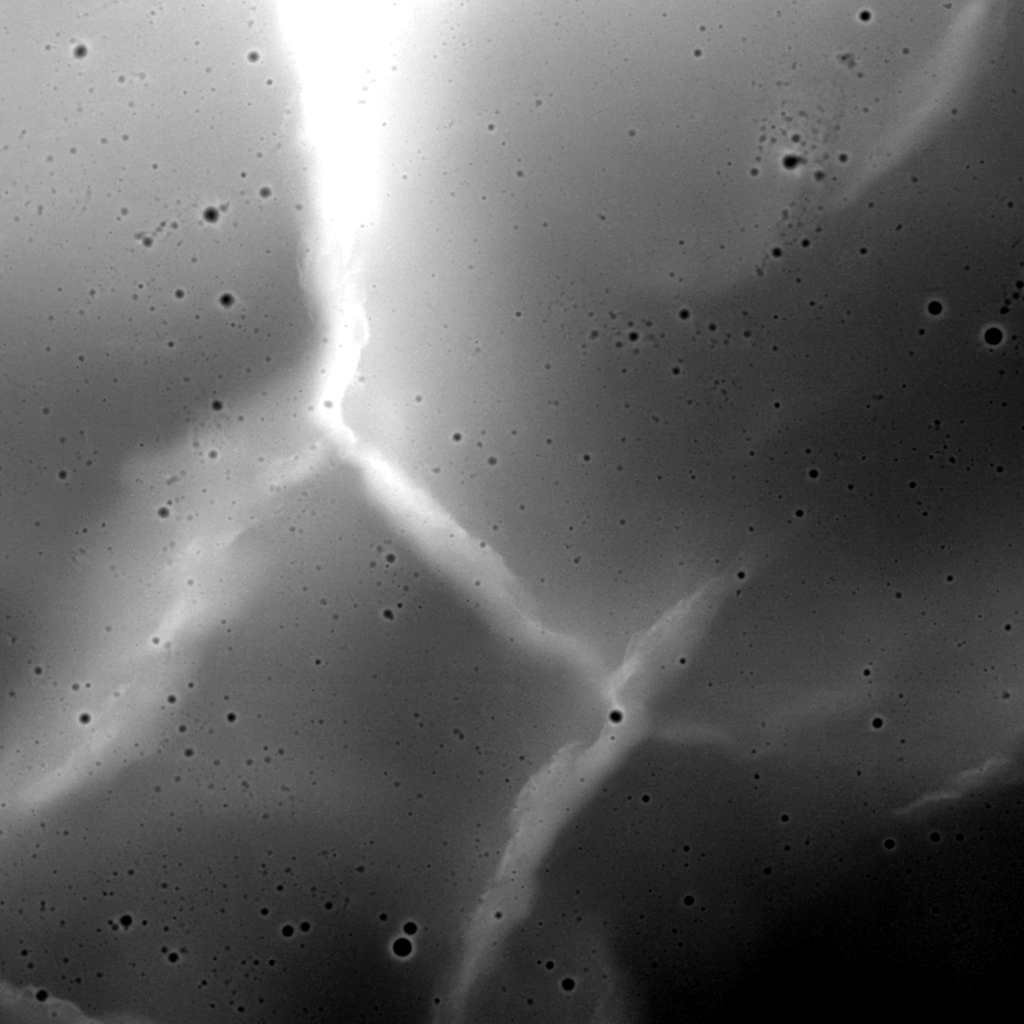

Supplement: Supplementary file 1 [file sensors-26-04344-s001.zip › data/images/test/tile_01879_lon57.0_lat12.0.png]

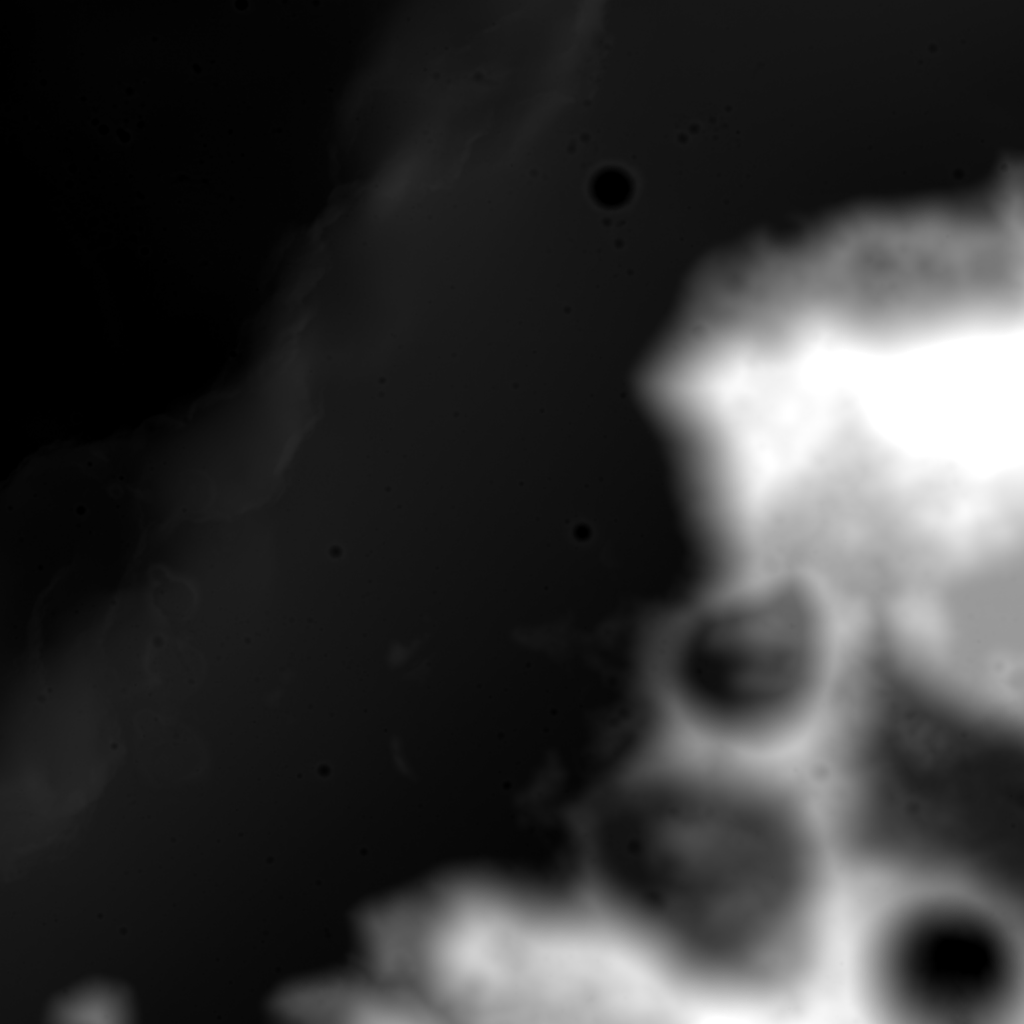

Supplement: Supplementary file 1 [file sensors-26-04344-s001.zip › data/images/test/tile_01881_lon63.0_lat12.0.png]

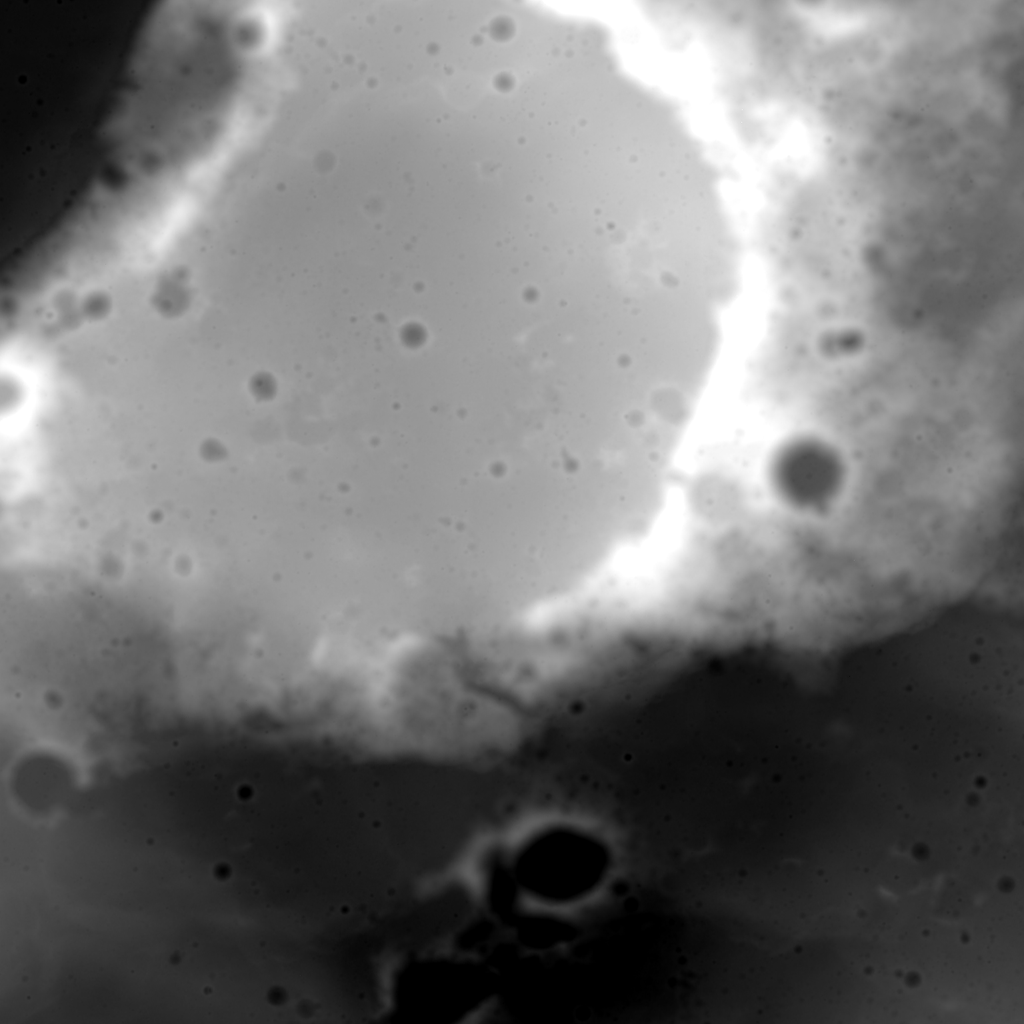

Supplement: Supplementary file 1 [file sensors-26-04344-s001.zip › data/images/test/tile_01890_lon90.0_lat12.0.png]

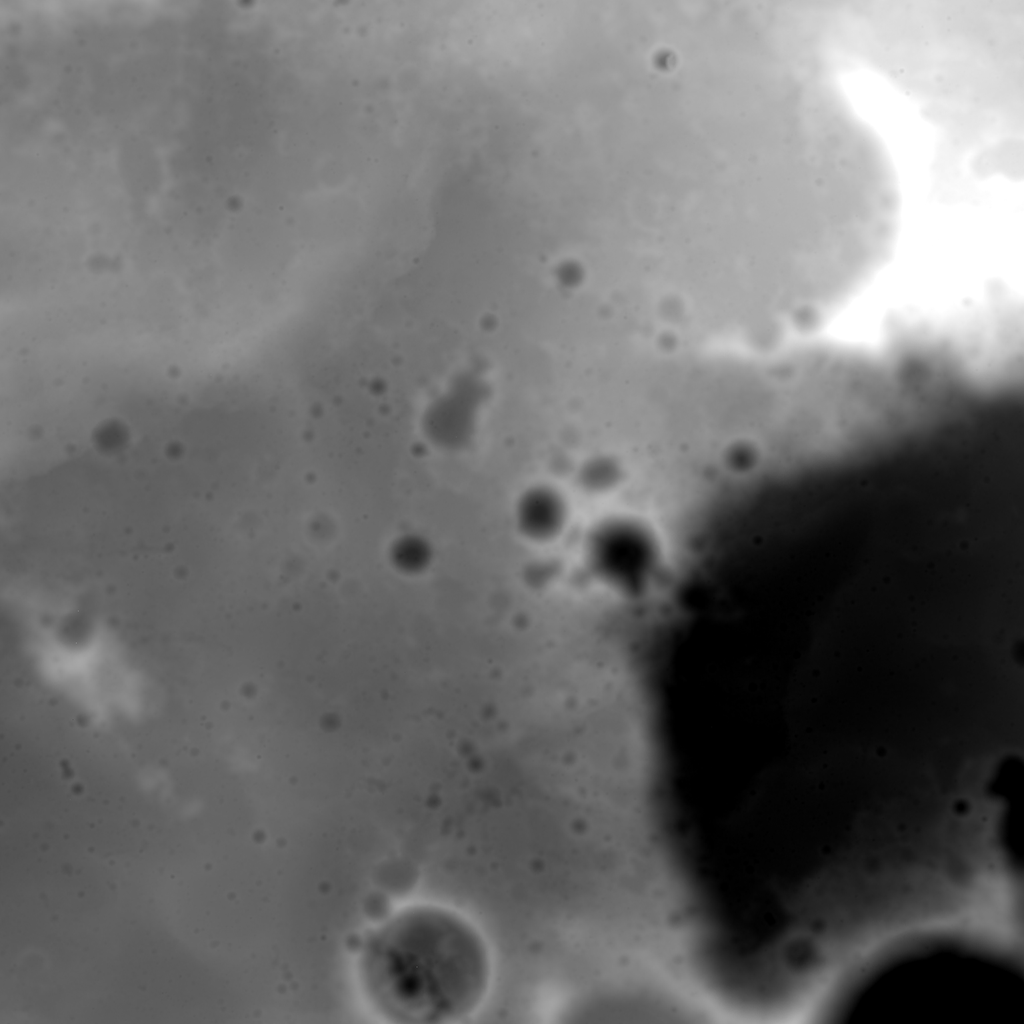

Supplement: Supplementary file 1 [file sensors-26-04344-s001.zip › data/images/test/tile_01891_lon93.0_lat12.0.png]
