# Supplementary figures and images for: FPW-YOLO11n: A Lightweight Frequency-Perception Framework for Lunar Impact Crater Detection (part 1 of 2)
Source: Sensors (Basel). 2026 Jul 9;26(14):4344. doi: 10.3390/s26144344 (PMC13417639; doi:10.3390/s26144344)

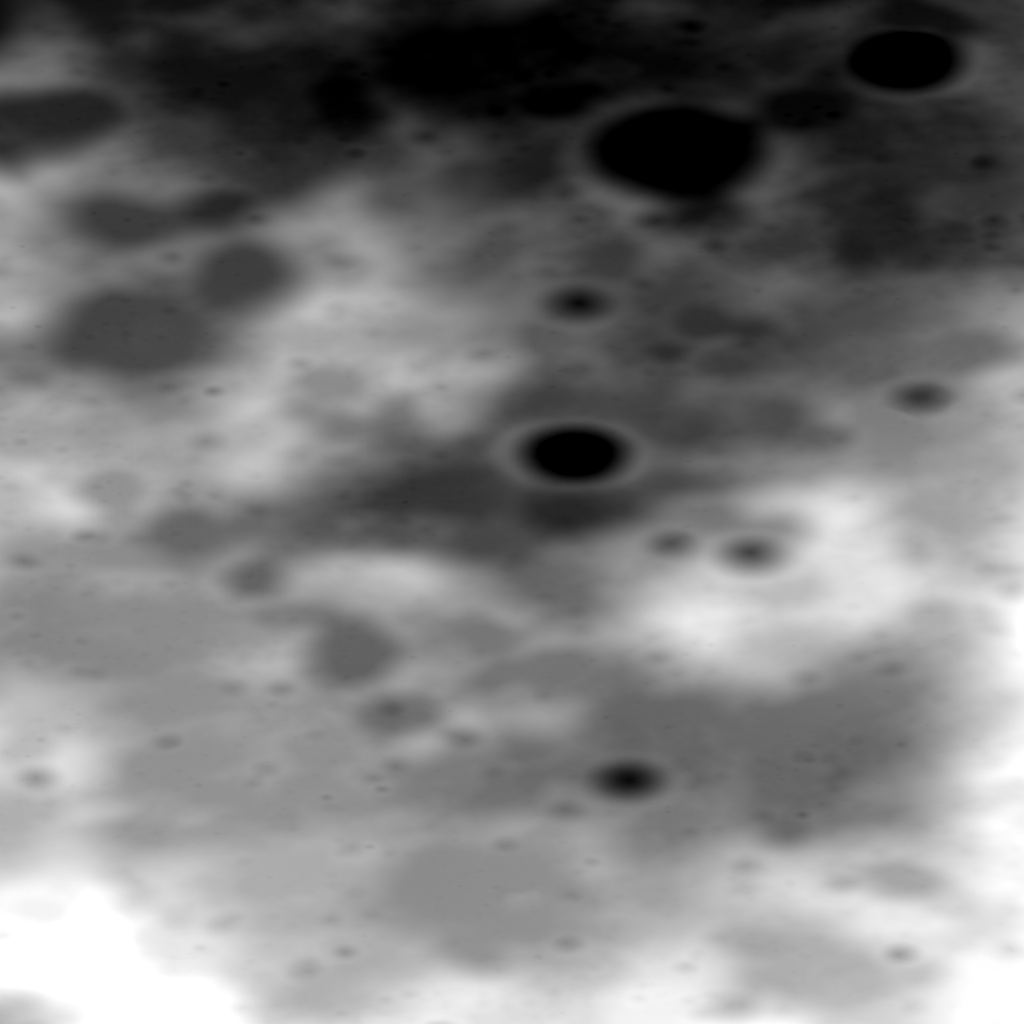

Supplement: Supplementary file 1 [file sensors-26-04344-s001.zip › data/images/test/tile_00003_lon-171.0_lat57.0.png]

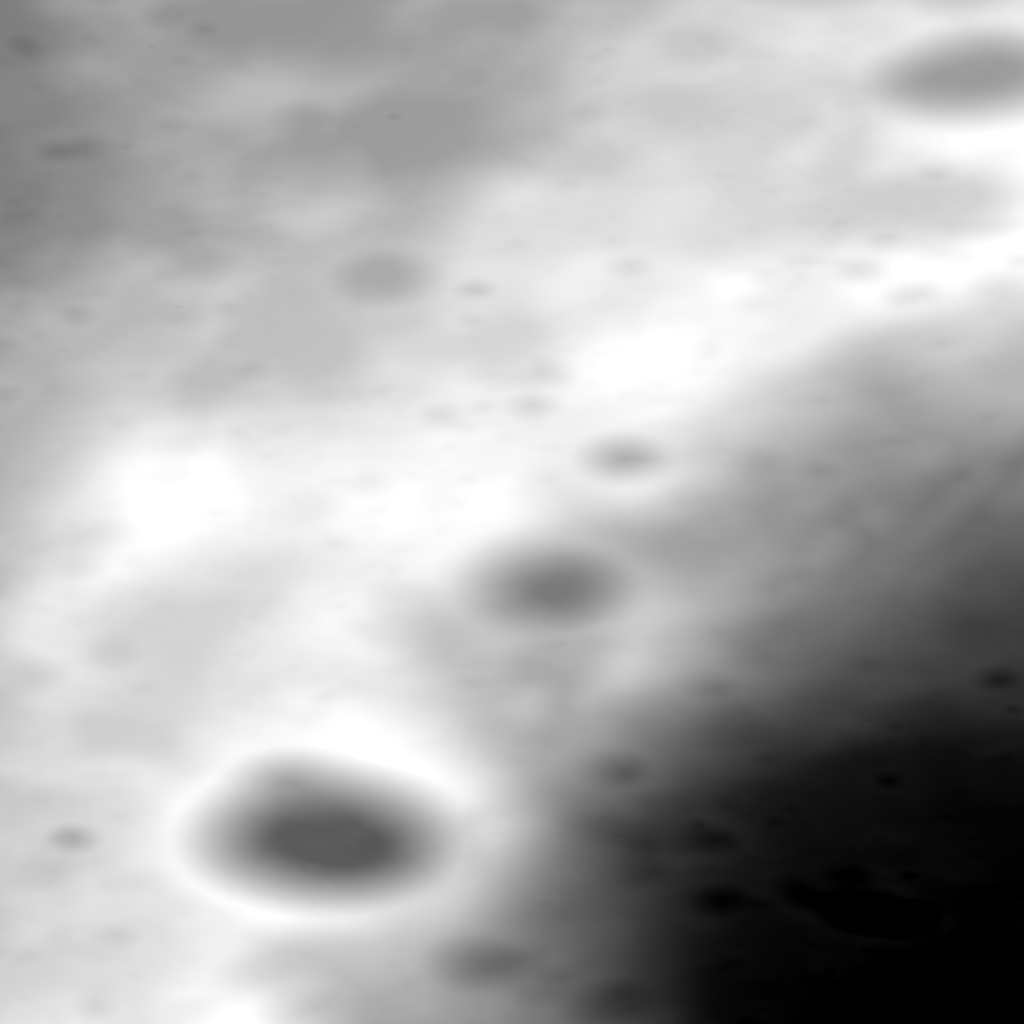

Supplement: Supplementary file 1 [file sensors-26-04344-s001.zip › data/images/test/tile_00004_lon-168.0_lat57.0.png]

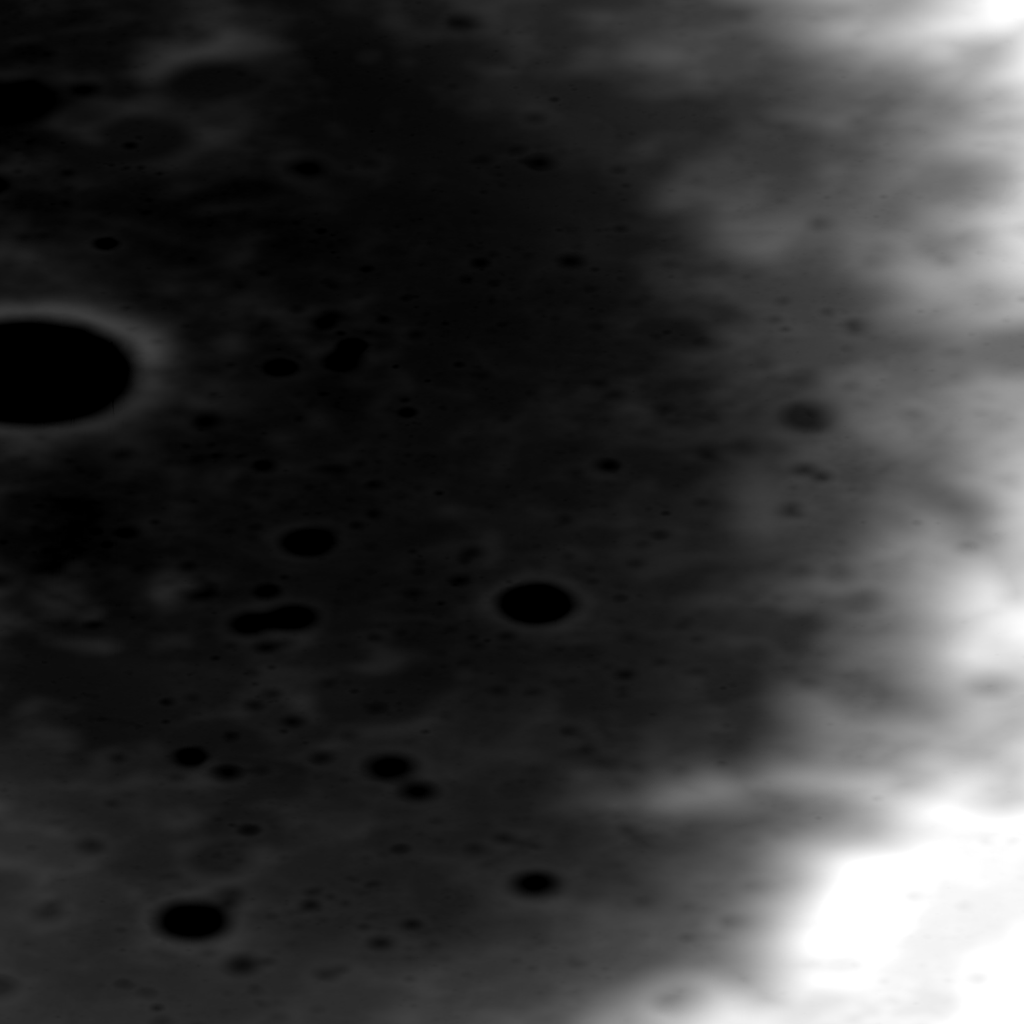

Supplement: Supplementary file 1 [file sensors-26-04344-s001.zip › data/images/test/tile_00013_lon-141.0_lat57.0.png]

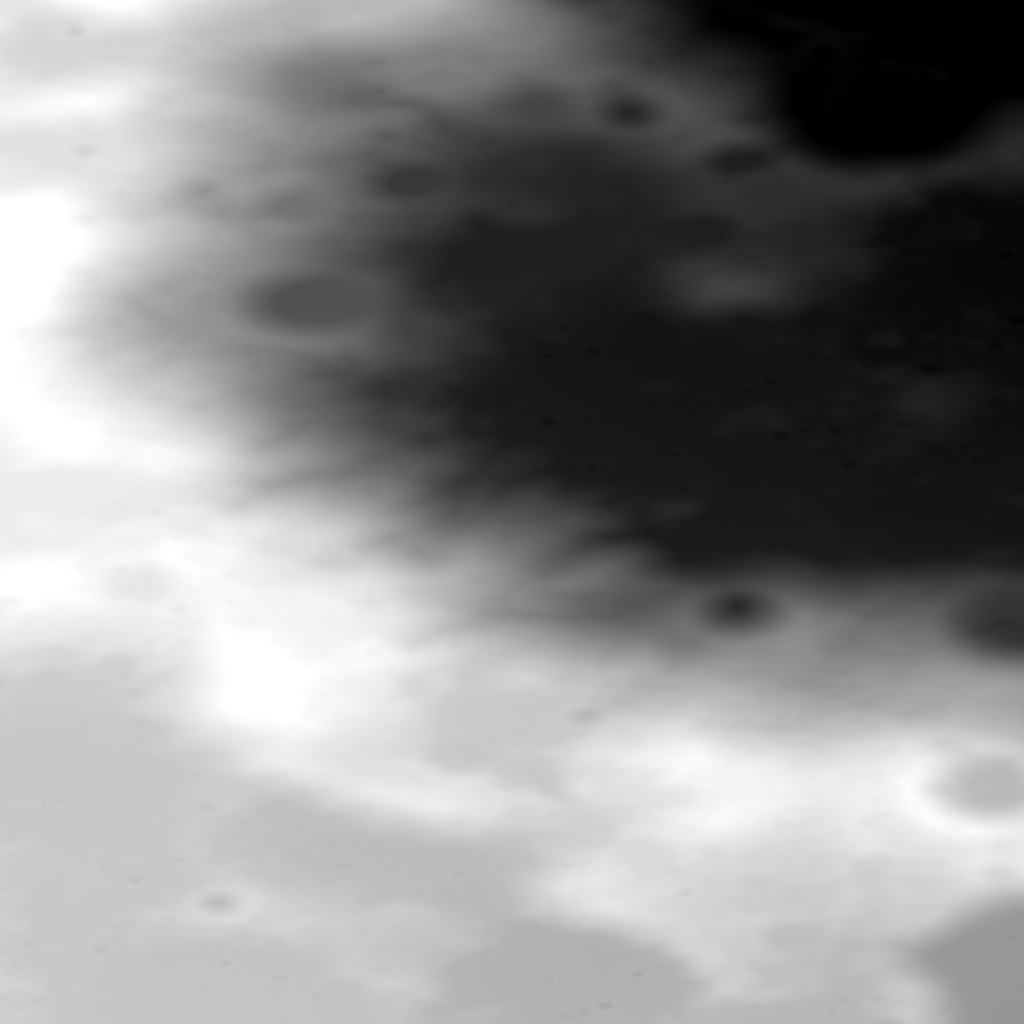

Supplement: Supplementary file 1 [file sensors-26-04344-s001.zip › data/images/test/tile_00017_lon-129.0_lat57.0.png]

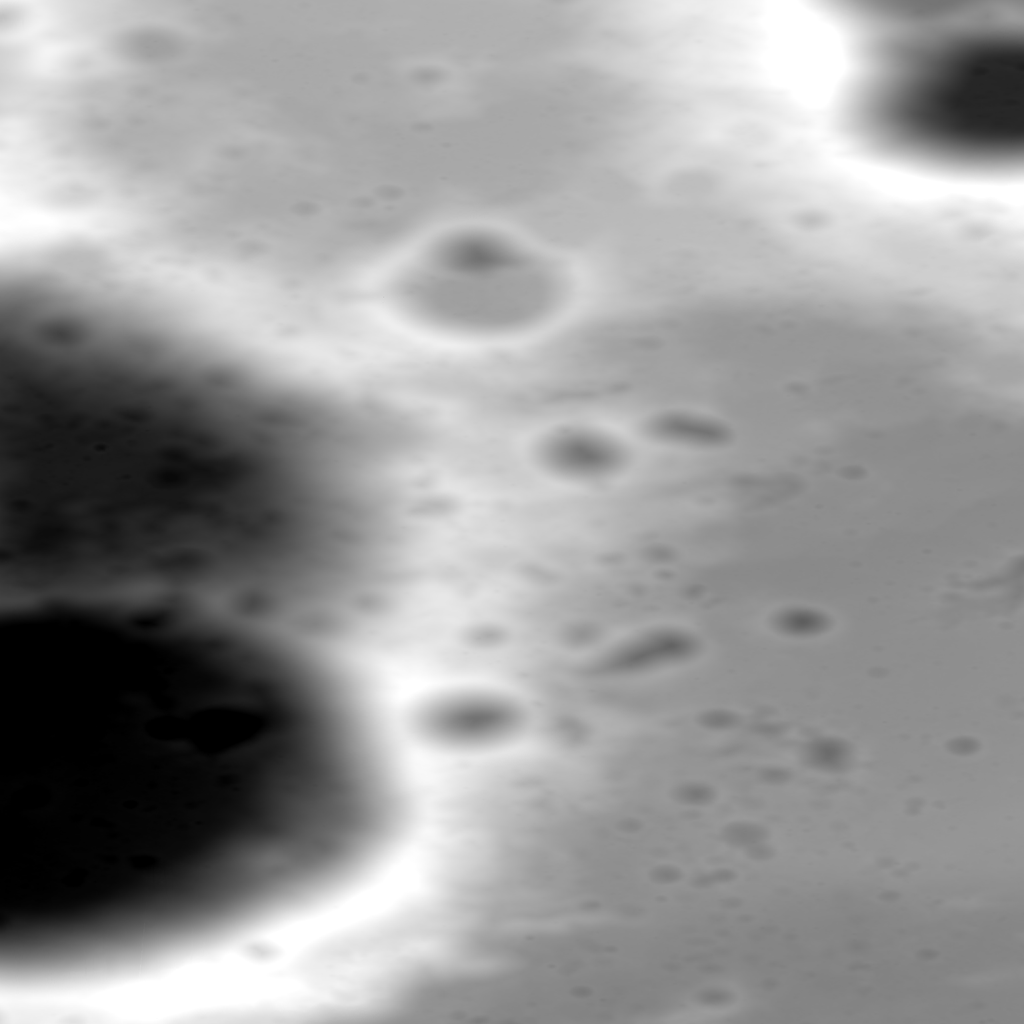

Supplement: Supplementary file 1 [file sensors-26-04344-s001.zip › data/images/test/tile_00026_lon-102.0_lat57.0.png]

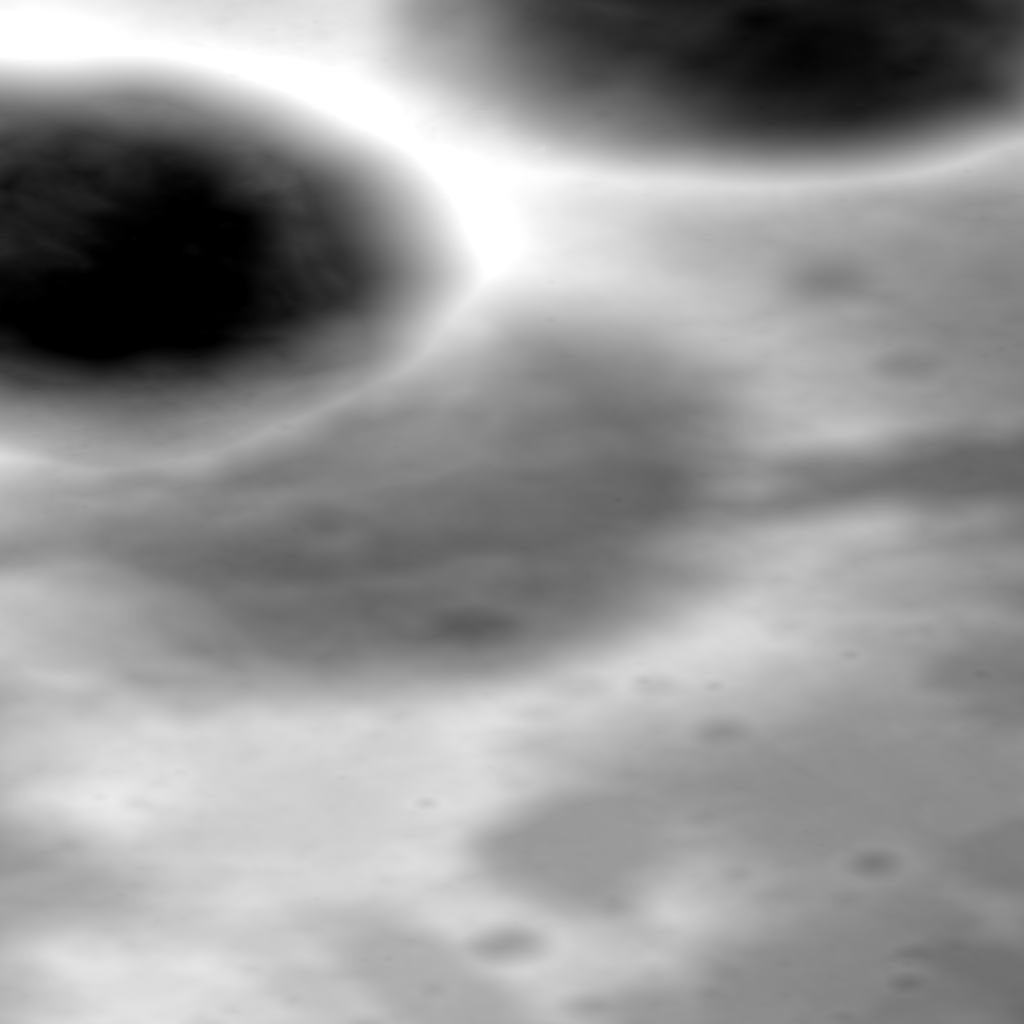

Supplement: Supplementary file 1 [file sensors-26-04344-s001.zip › data/images/test/tile_00029_lon-93.0_lat57.0.png]

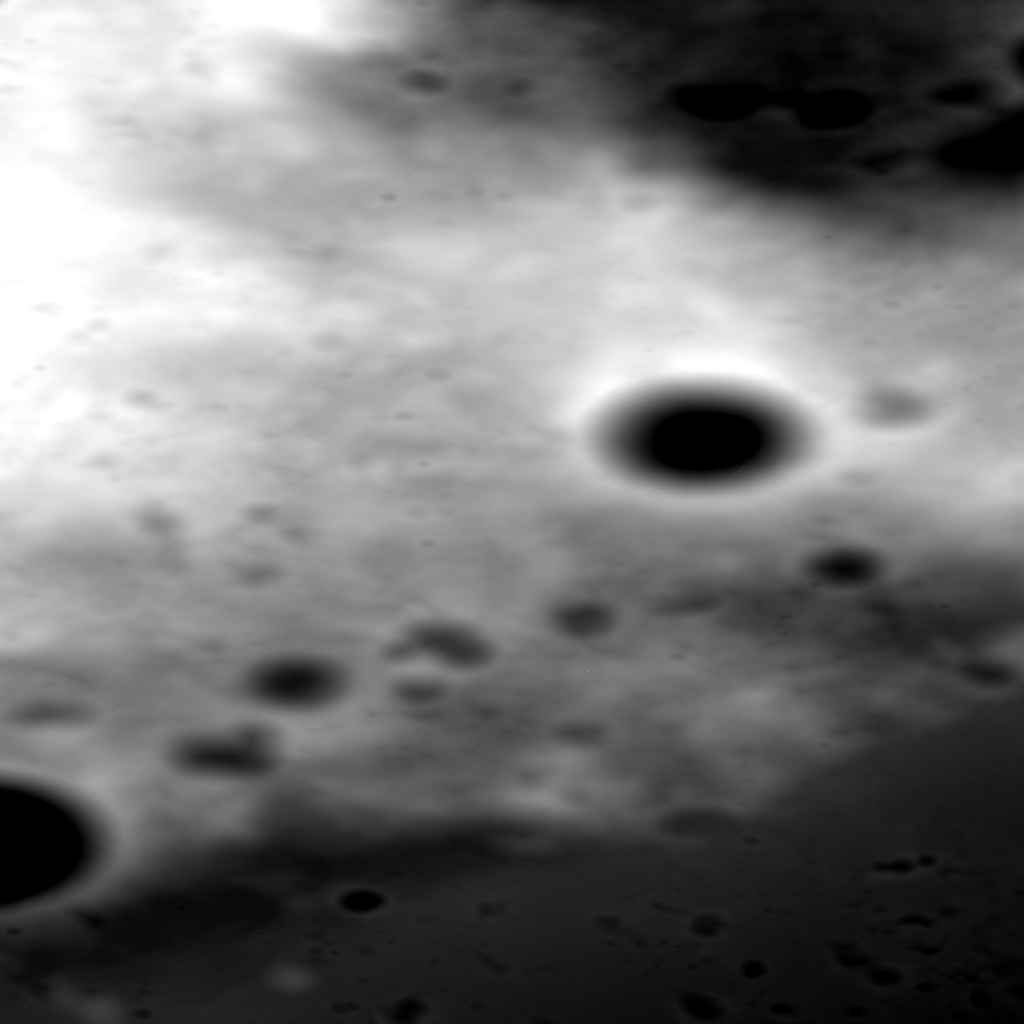

Supplement: Supplementary file 1 [file sensors-26-04344-s001.zip › data/images/test/tile_00045_lon-45.0_lat57.0.png]

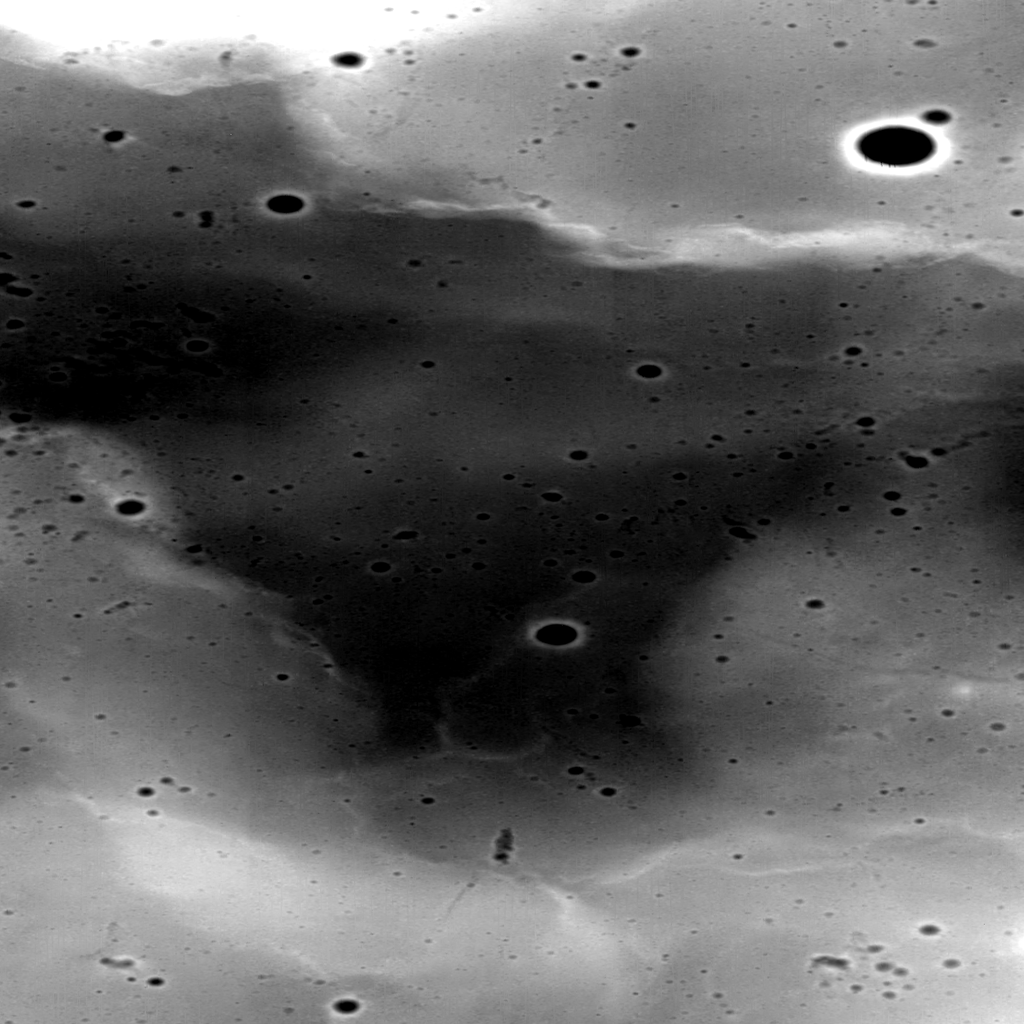

Supplement: Supplementary file 1 [file sensors-26-04344-s001.zip › data/images/test/tile_00053_lon-21.0_lat57.0.png]

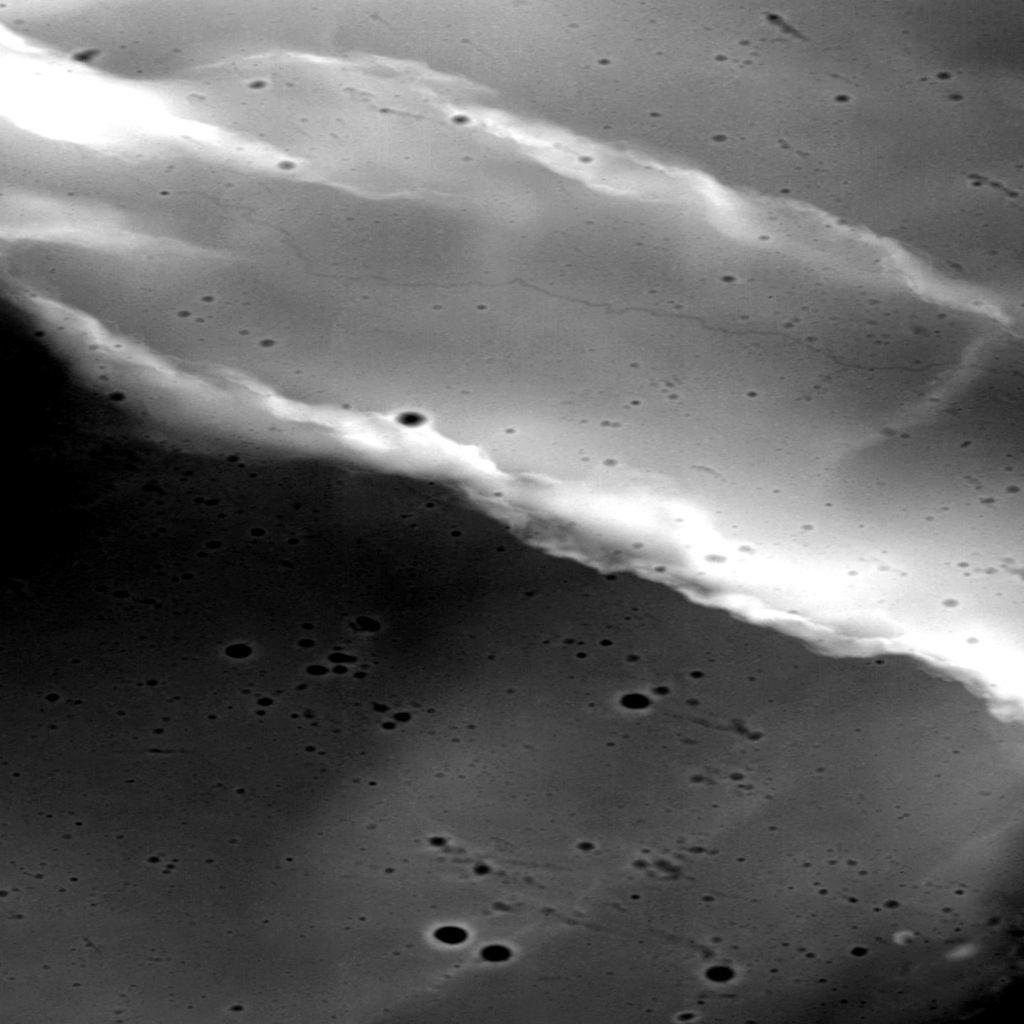

Supplement: Supplementary file 1 [file sensors-26-04344-s001.zip › data/images/test/tile_00058_lon-6.0_lat57.0.png]

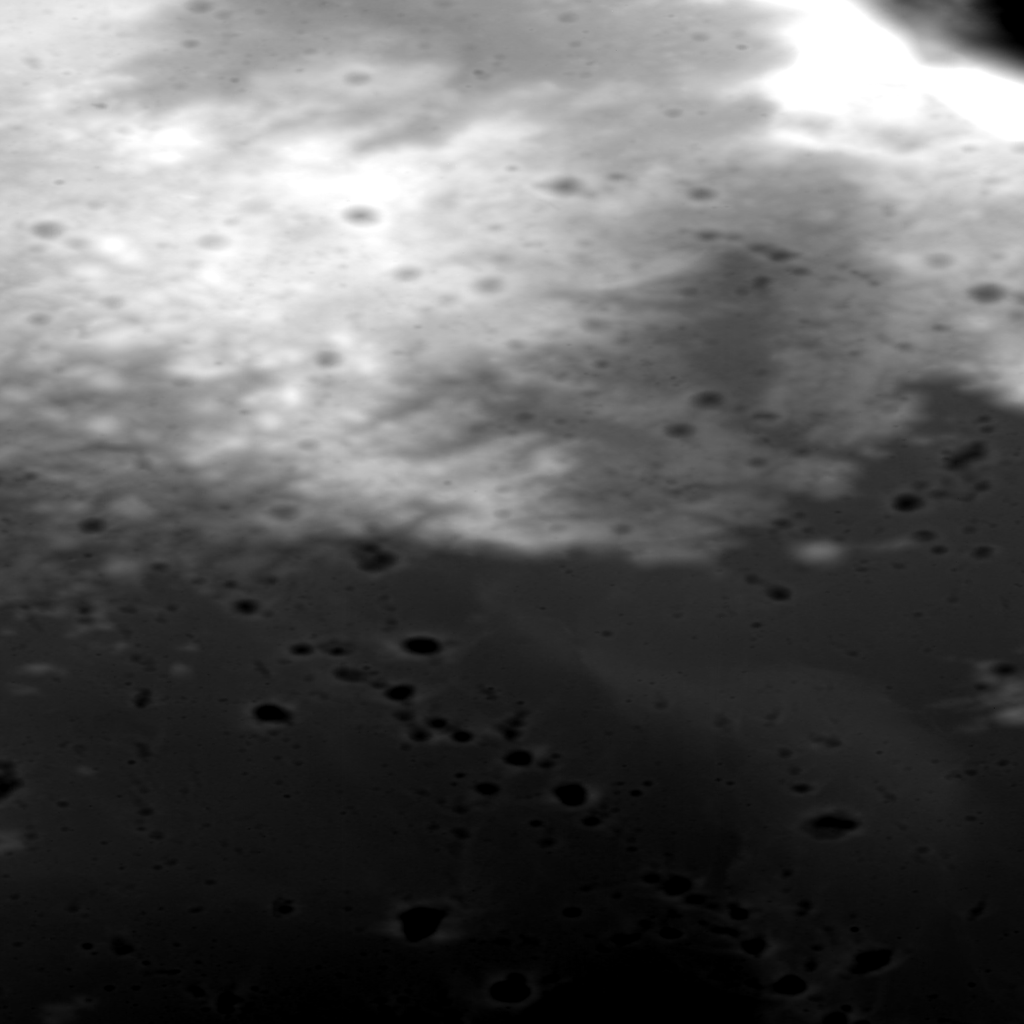

Supplement: Supplementary file 1 [file sensors-26-04344-s001.zip › data/images/test/tile_00064_lon12.0_lat57.0.png]

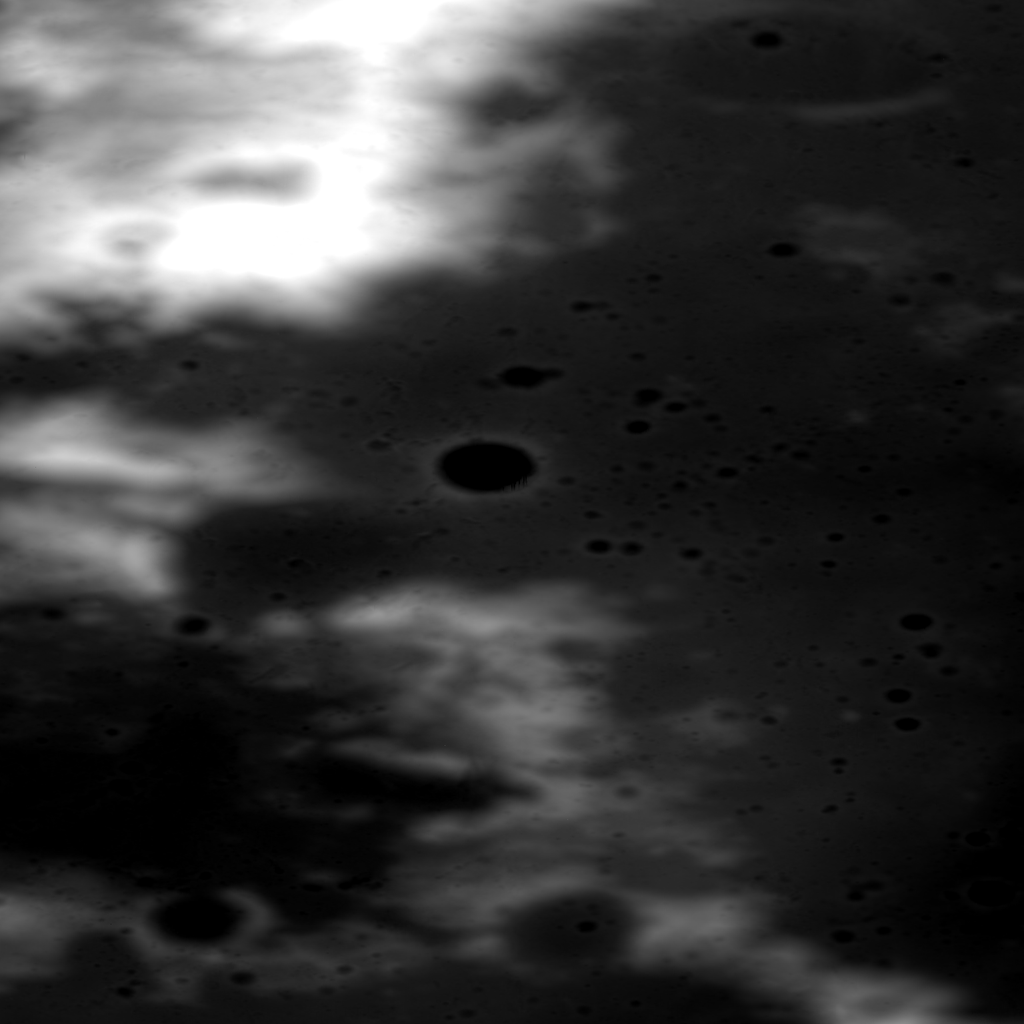

Supplement: Supplementary file 1 [file sensors-26-04344-s001.zip › data/images/test/tile_00076_lon48.0_lat57.0.png]

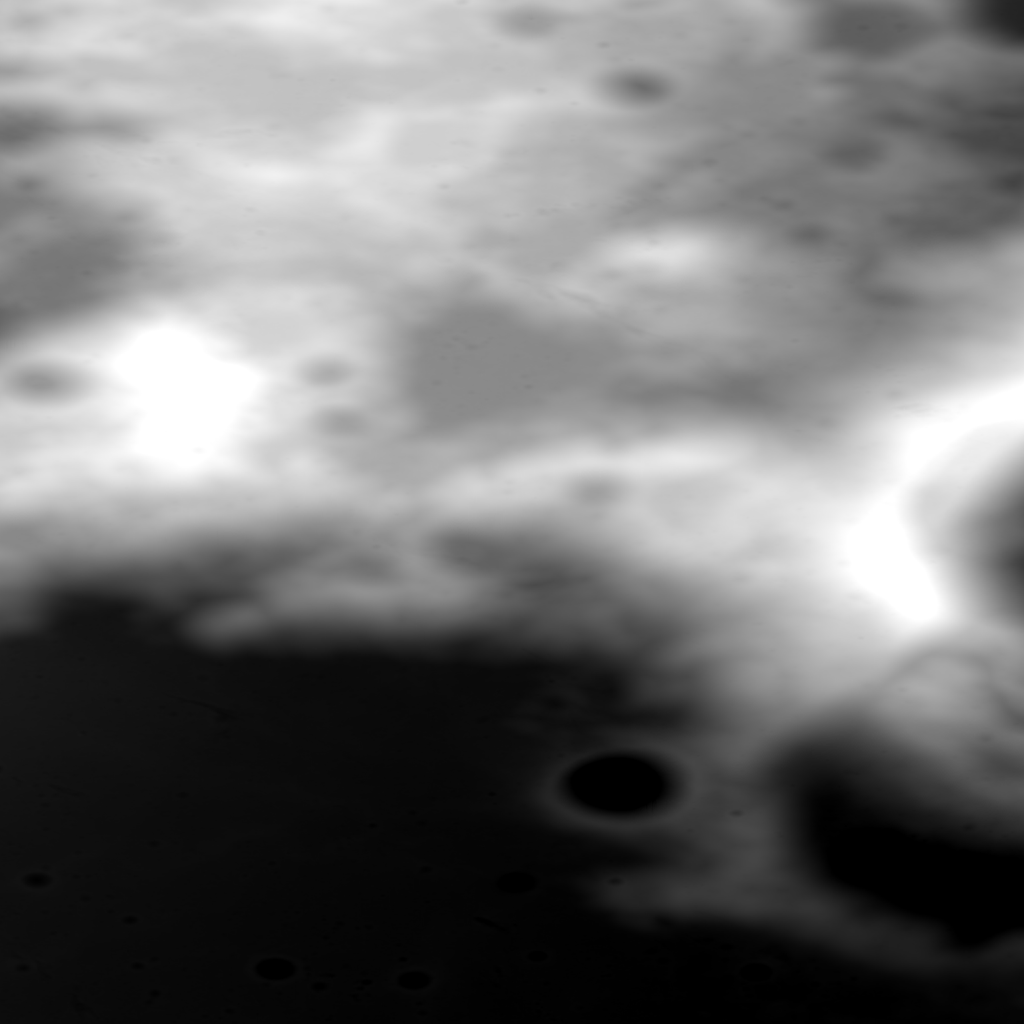

Supplement: Supplementary file 1 [file sensors-26-04344-s001.zip › data/images/test/tile_00079_lon57.0_lat57.0.png]

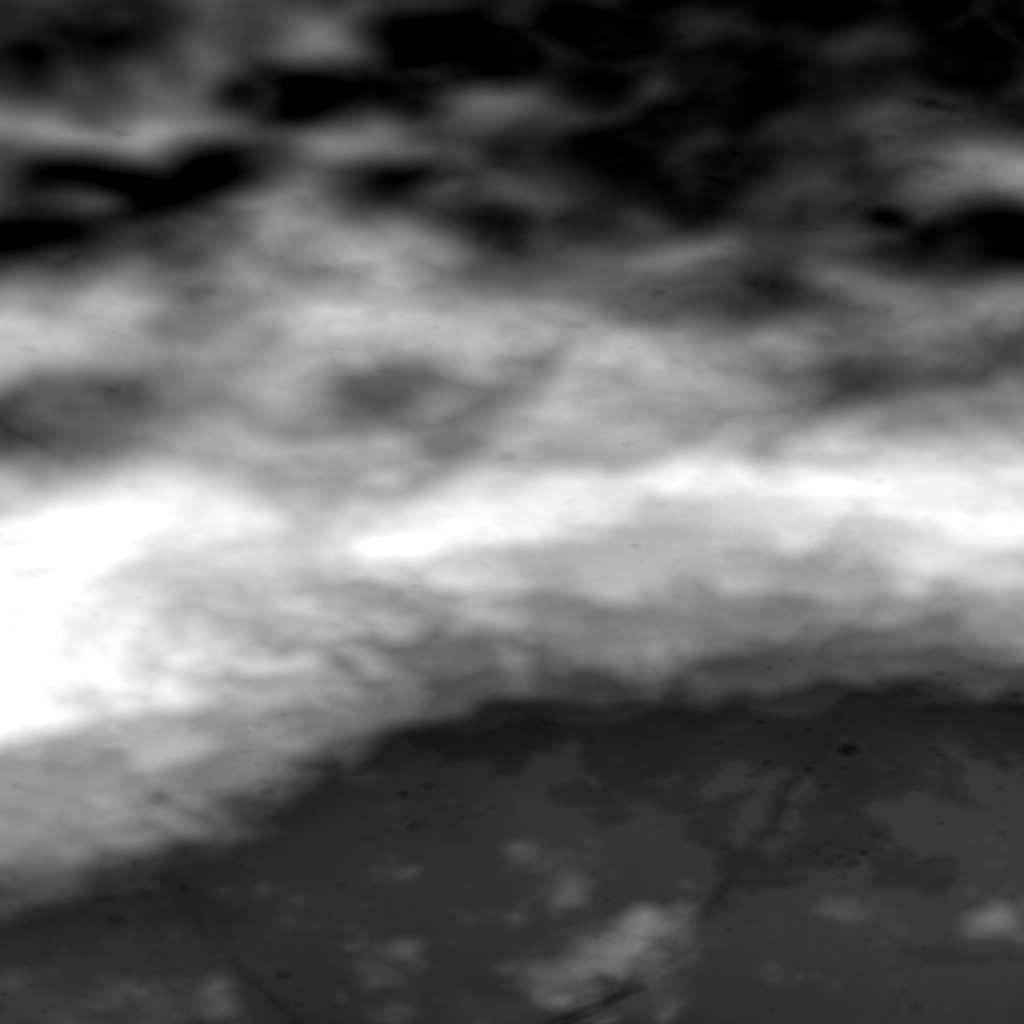

Supplement: Supplementary file 1 [file sensors-26-04344-s001.zip › data/images/test/tile_00094_lon102.0_lat57.0.png]

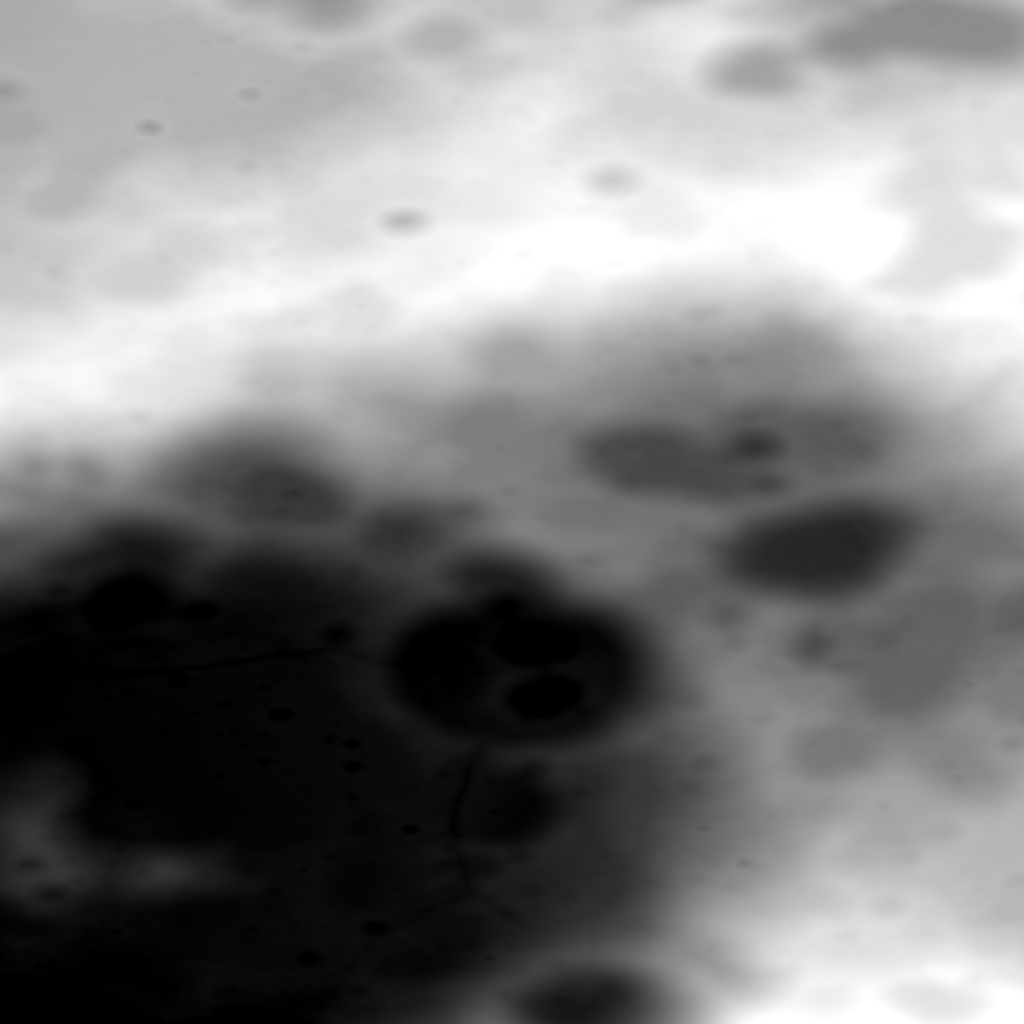

Supplement: Supplementary file 1 [file sensors-26-04344-s001.zip › data/images/test/tile_00121_lon-177.0_lat54.0.png]

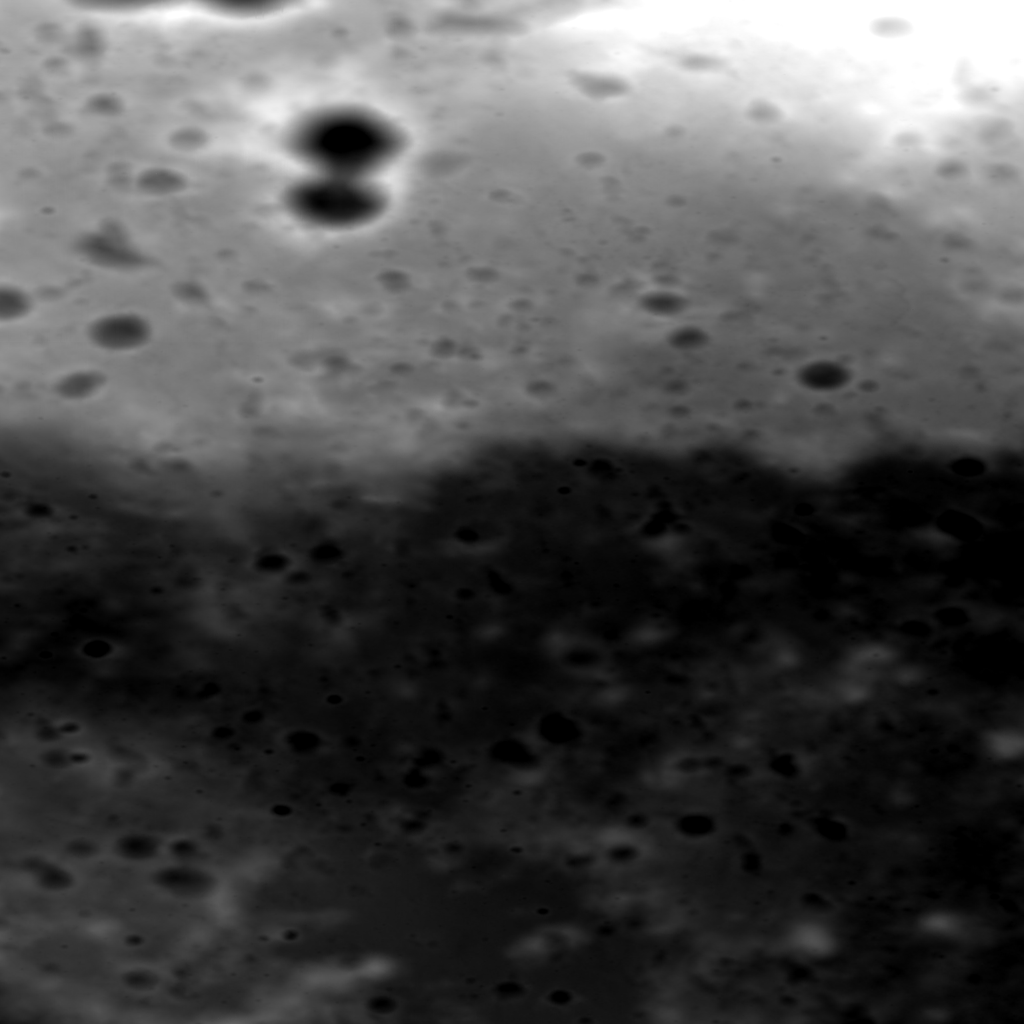

Supplement: Supplementary file 1 [file sensors-26-04344-s001.zip › data/images/test/tile_00160_lon-60.0_lat54.0.png]

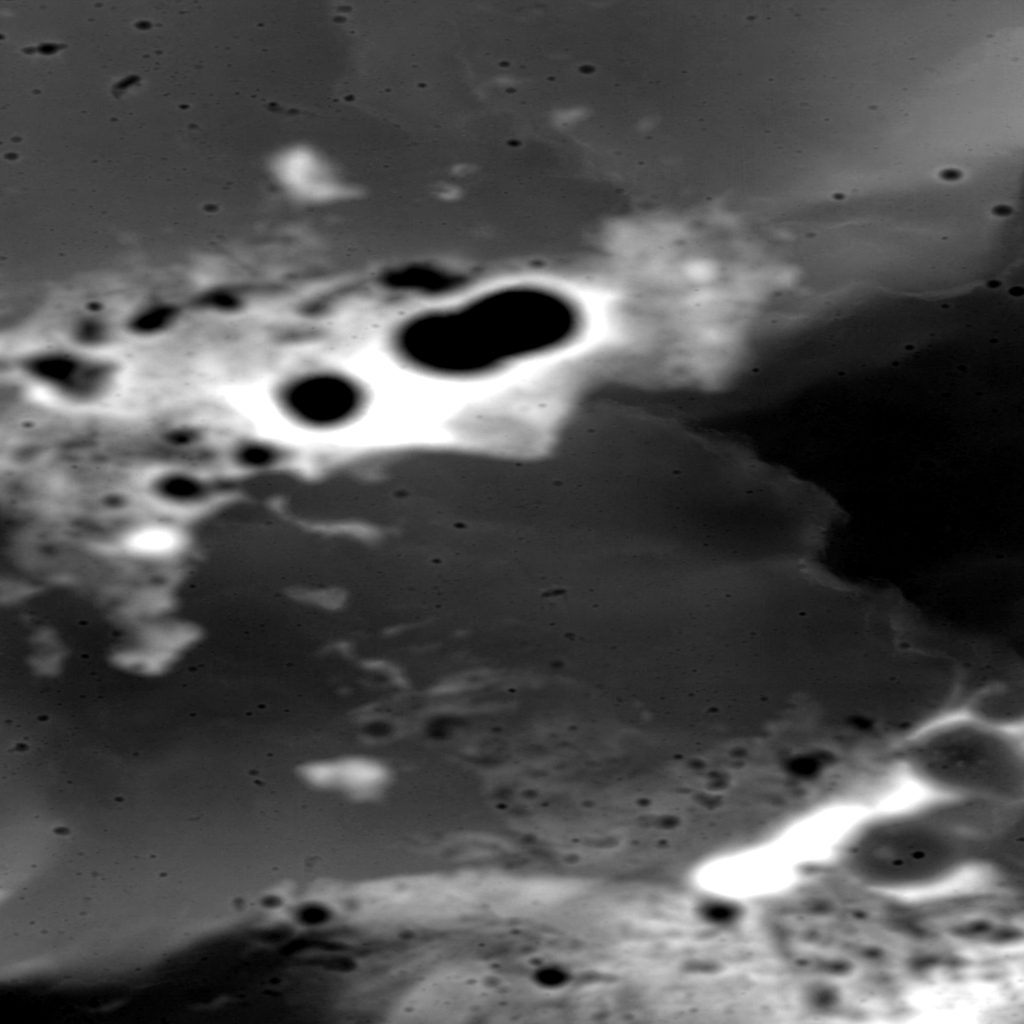

Supplement: Supplementary file 1 [file sensors-26-04344-s001.zip › data/images/test/tile_00173_lon-21.0_lat54.0.png]

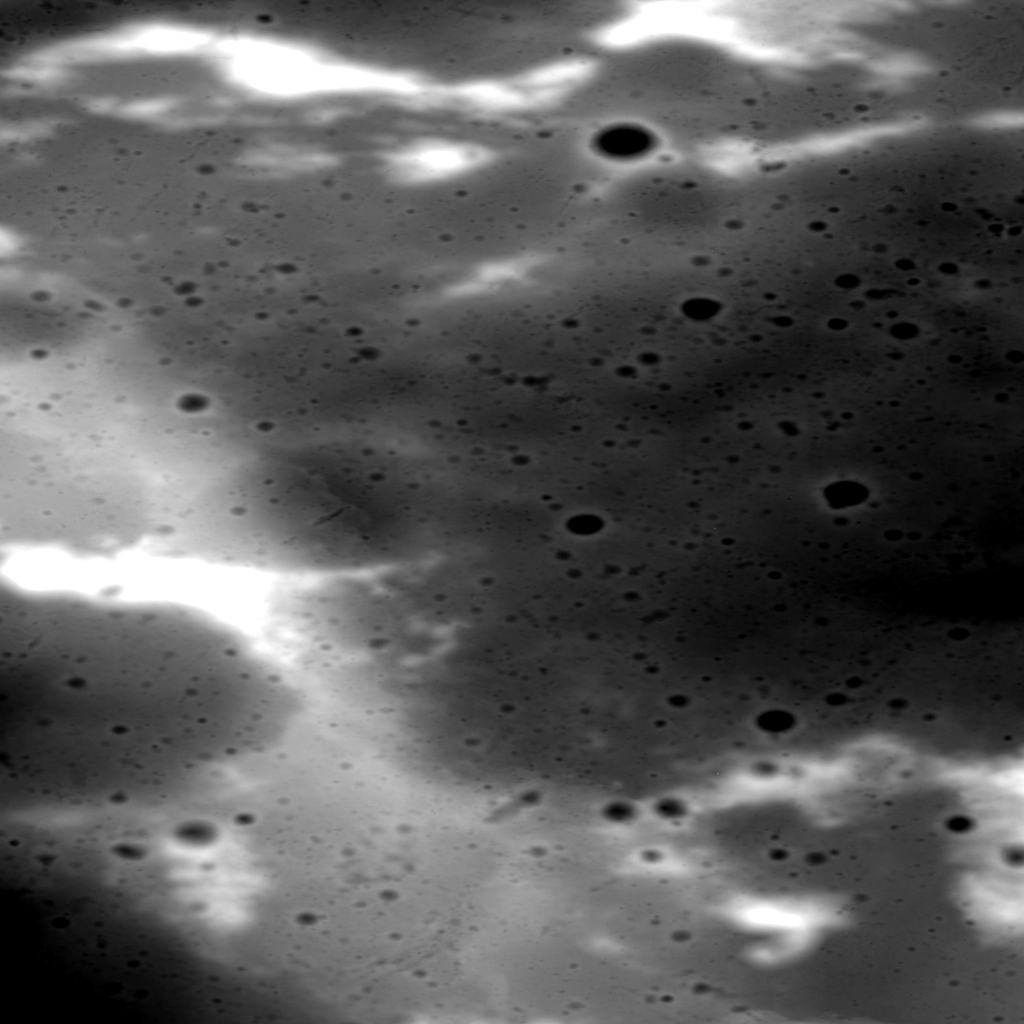

Supplement: Supplementary file 1 [file sensors-26-04344-s001.zip › data/images/test/tile_00194_lon42.0_lat54.0.png]

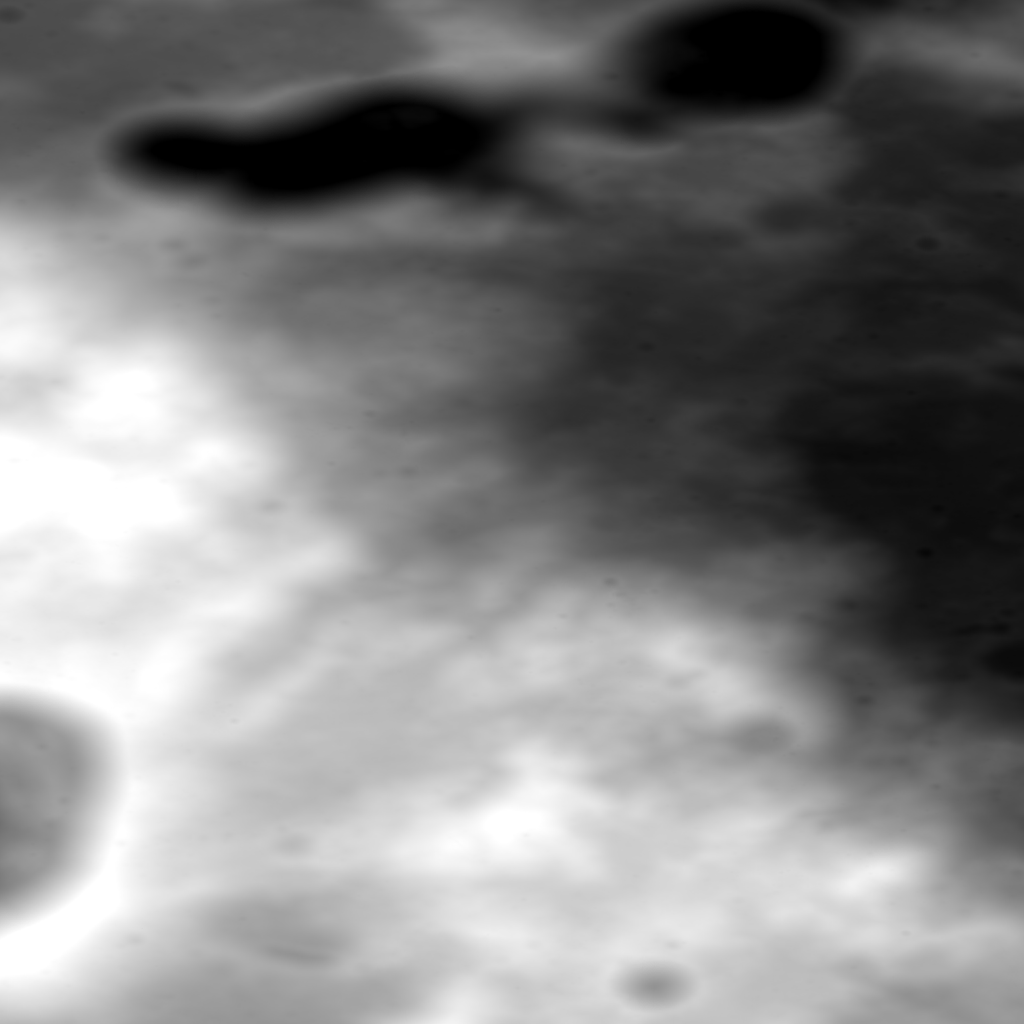

Supplement: Supplementary file 1 [file sensors-26-04344-s001.zip › data/images/test/tile_00201_lon63.0_lat54.0.png]

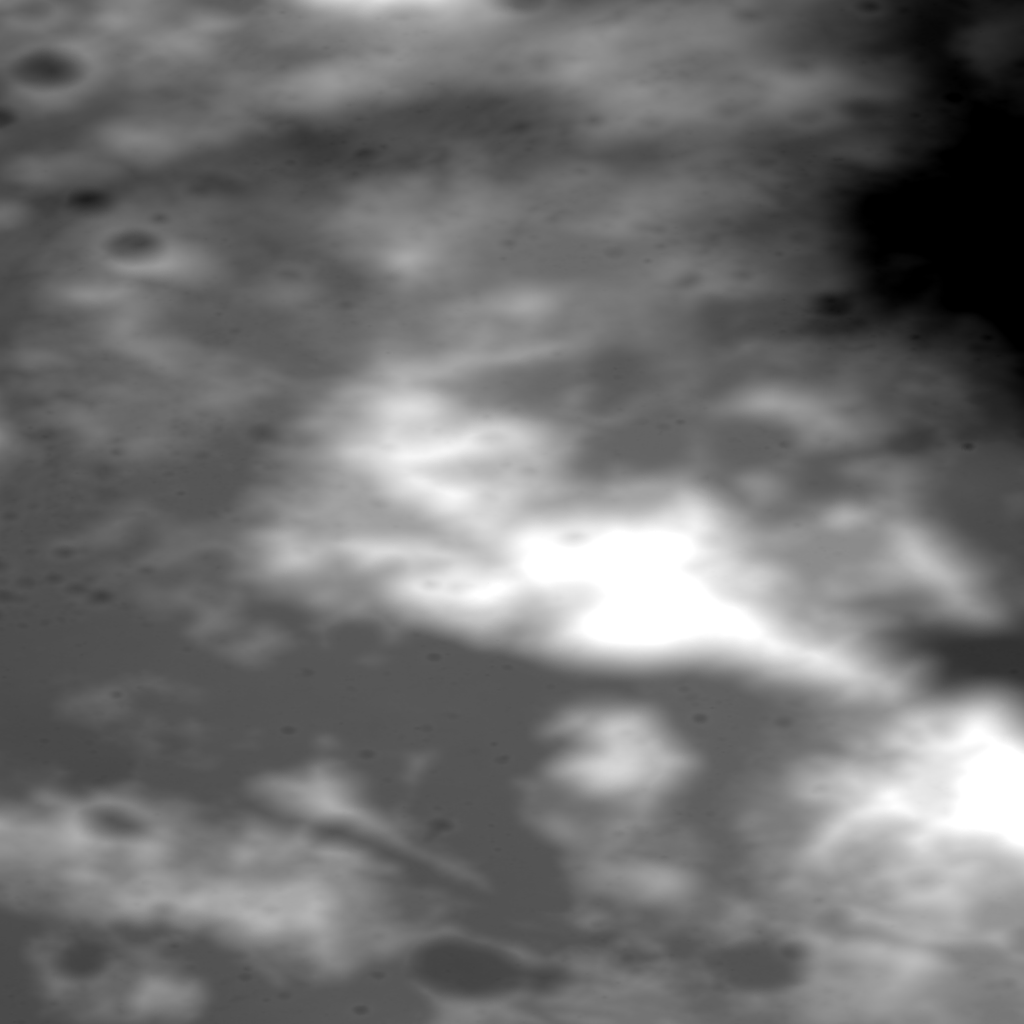

Supplement: Supplementary file 1 [file sensors-26-04344-s001.zip › data/images/test/tile_00204_lon72.0_lat54.0.png]

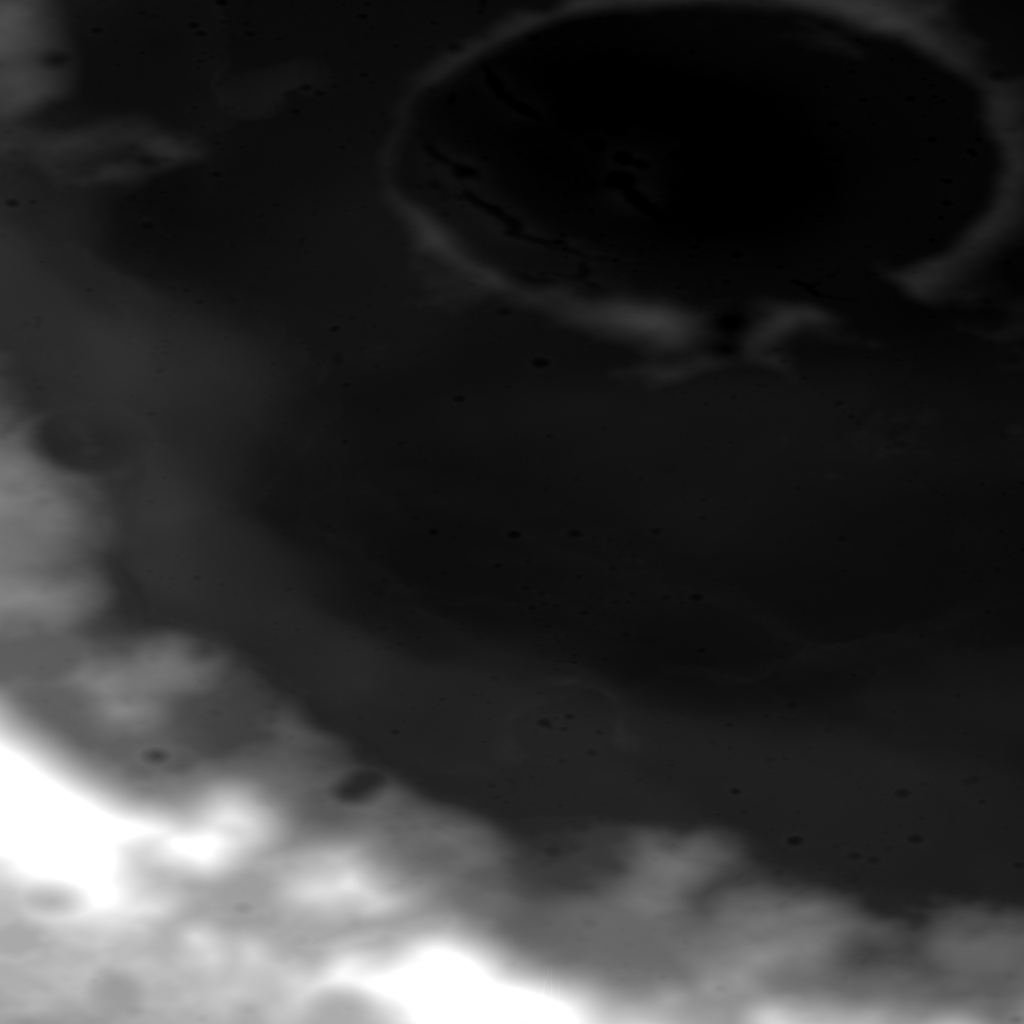

Supplement: Supplementary file 1 [file sensors-26-04344-s001.zip › data/images/test/tile_00205_lon75.0_lat54.0.png]

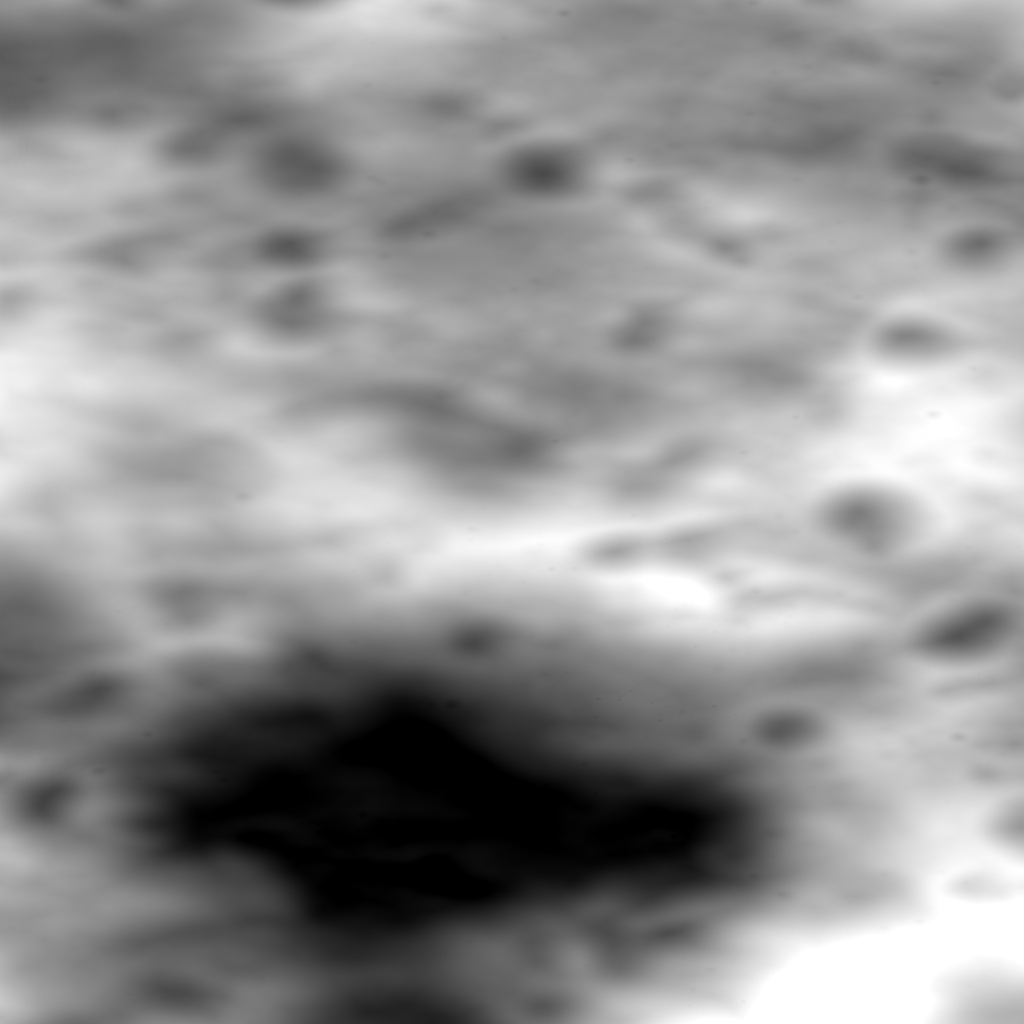

Supplement: Supplementary file 1 [file sensors-26-04344-s001.zip › data/images/test/tile_00218_lon114.0_lat54.0.png]

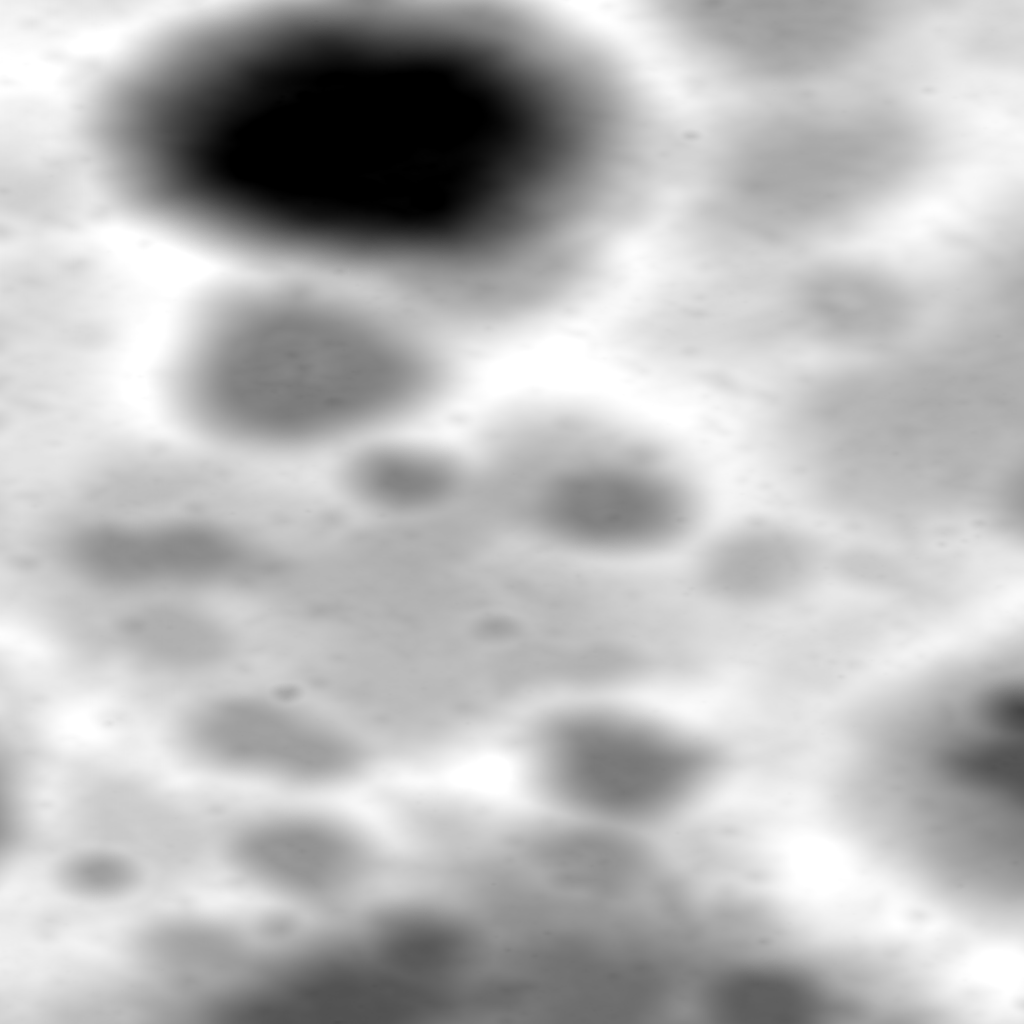

Supplement: Supplementary file 1 [file sensors-26-04344-s001.zip › data/images/test/tile_00227_lon141.0_lat54.0.png]

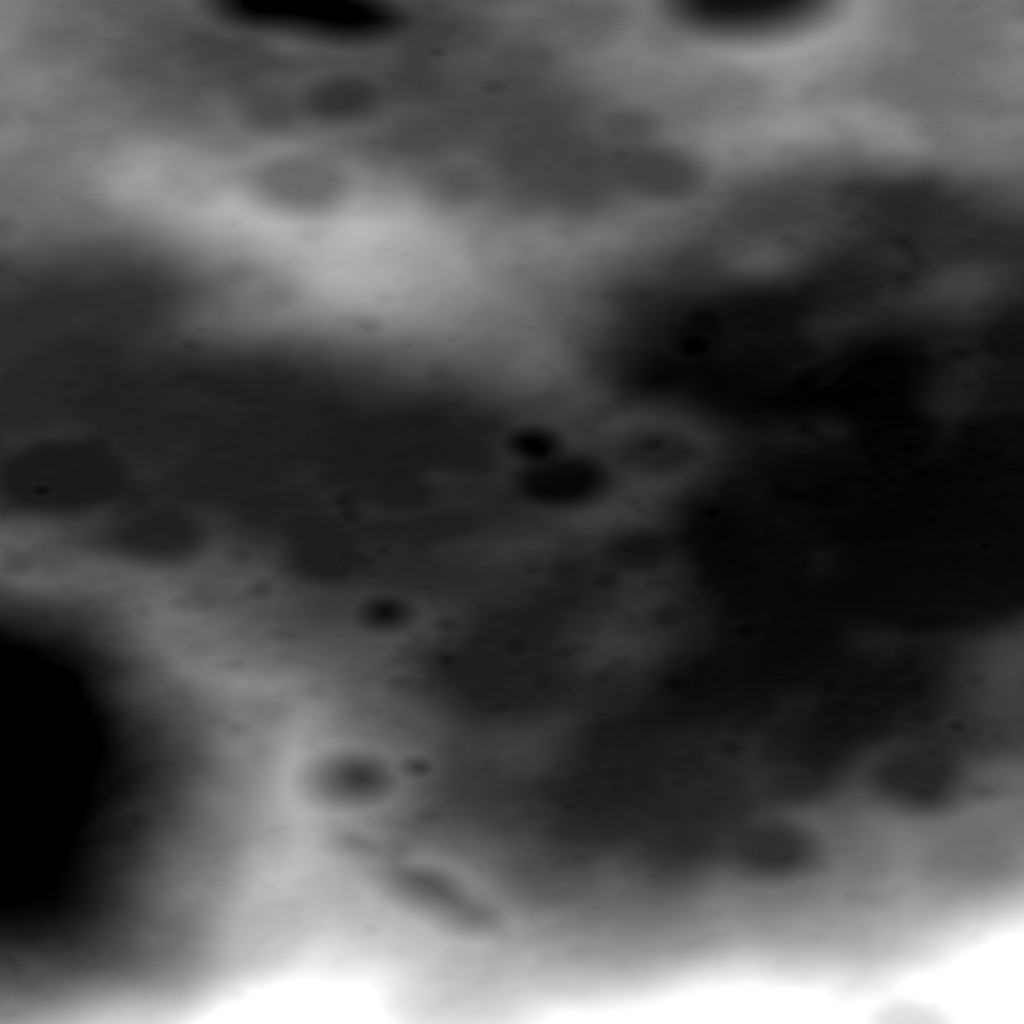

Supplement: Supplementary file 1 [file sensors-26-04344-s001.zip › data/images/test/tile_00246_lon-162.0_lat51.0.png]

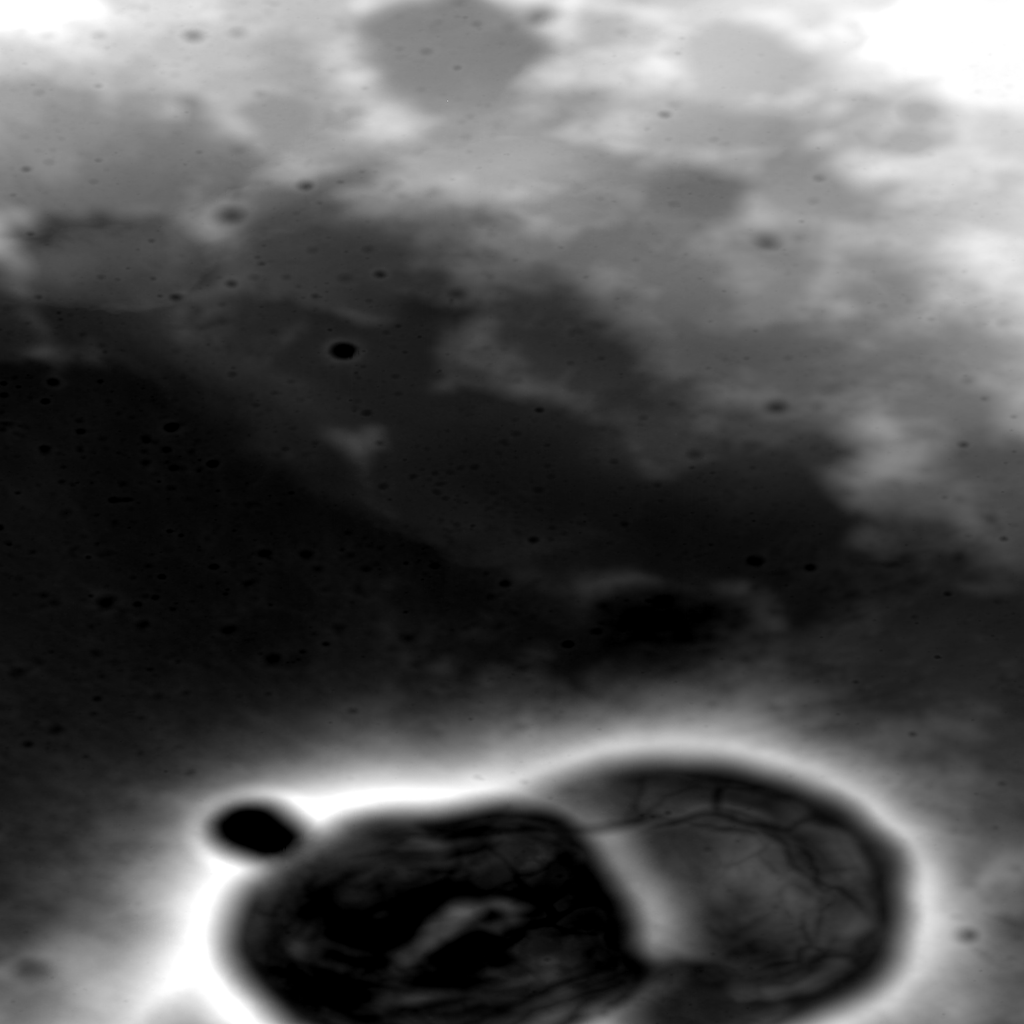

Supplement: Supplementary file 1 [file sensors-26-04344-s001.zip › data/images/test/tile_00259_lon-123.0_lat51.0.png]

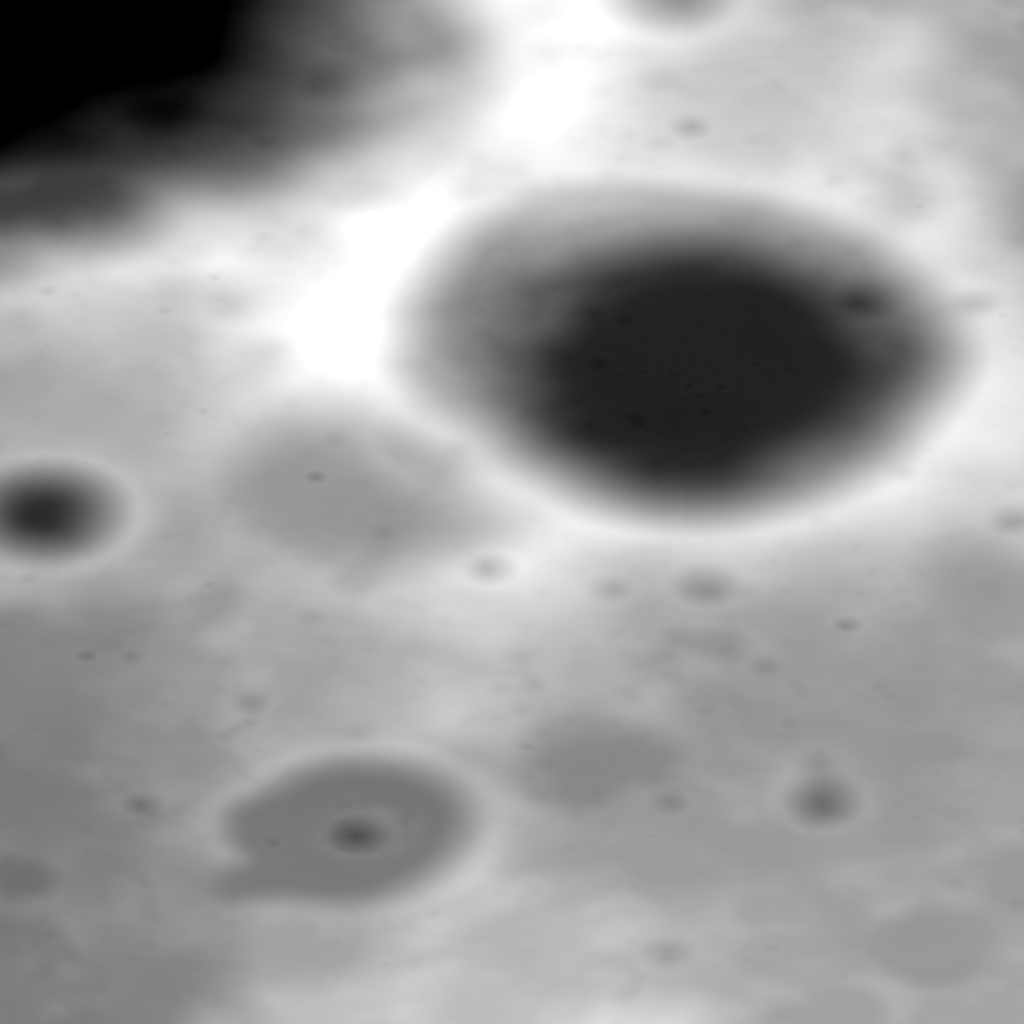

Supplement: Supplementary file 1 [file sensors-26-04344-s001.zip › data/images/test/tile_00262_lon-114.0_lat51.0.png]

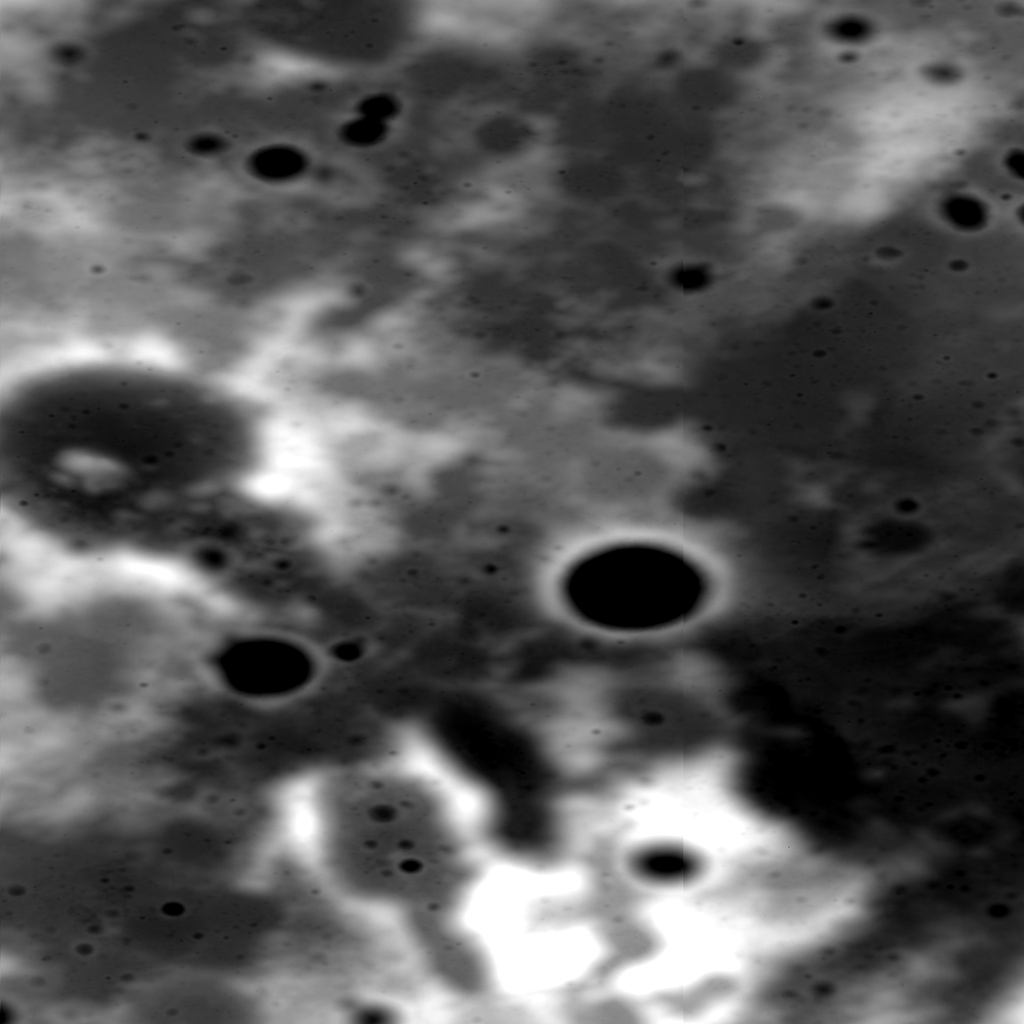

Supplement: Supplementary file 1 [file sensors-26-04344-s001.zip › data/images/test/tile_00264_lon-108.0_lat51.0.png]

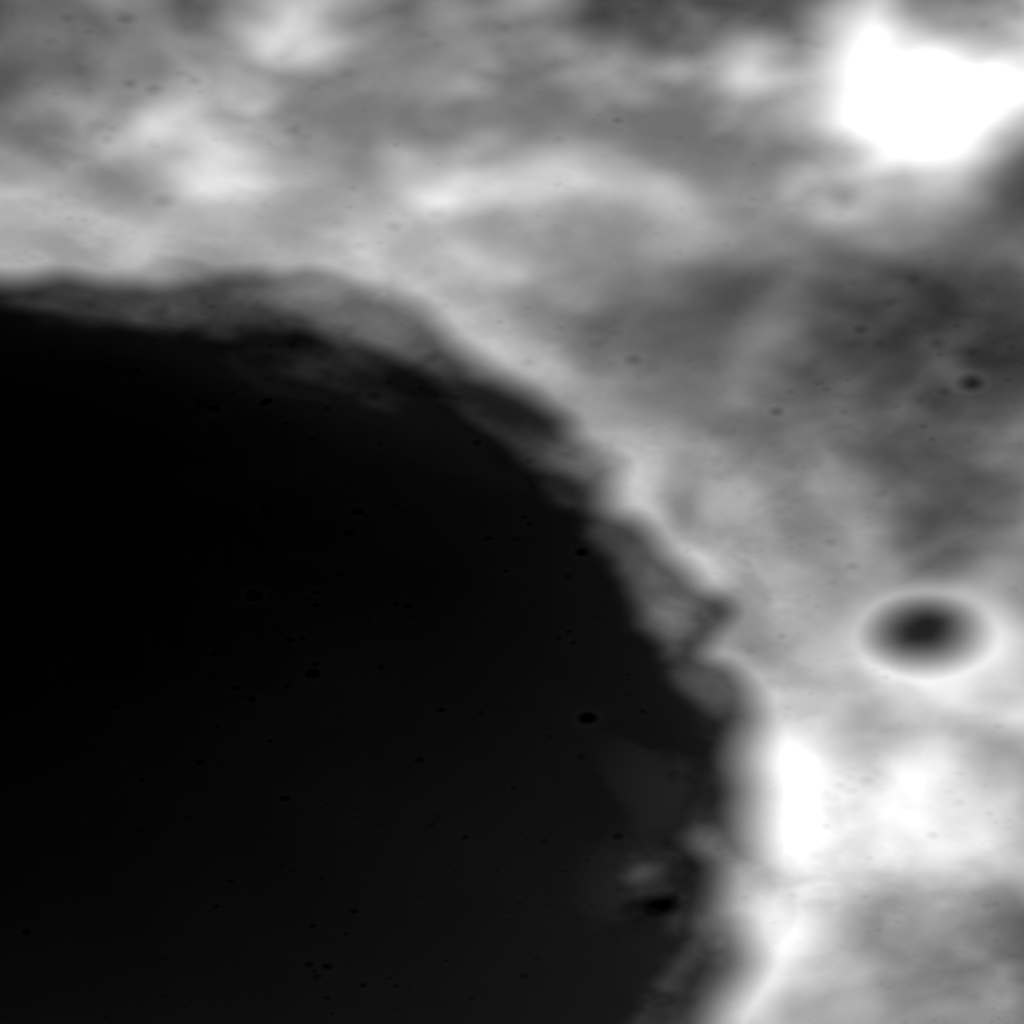

Supplement: Supplementary file 1 [file sensors-26-04344-s001.zip › data/images/test/tile_00297_lon-9.0_lat51.0.png]

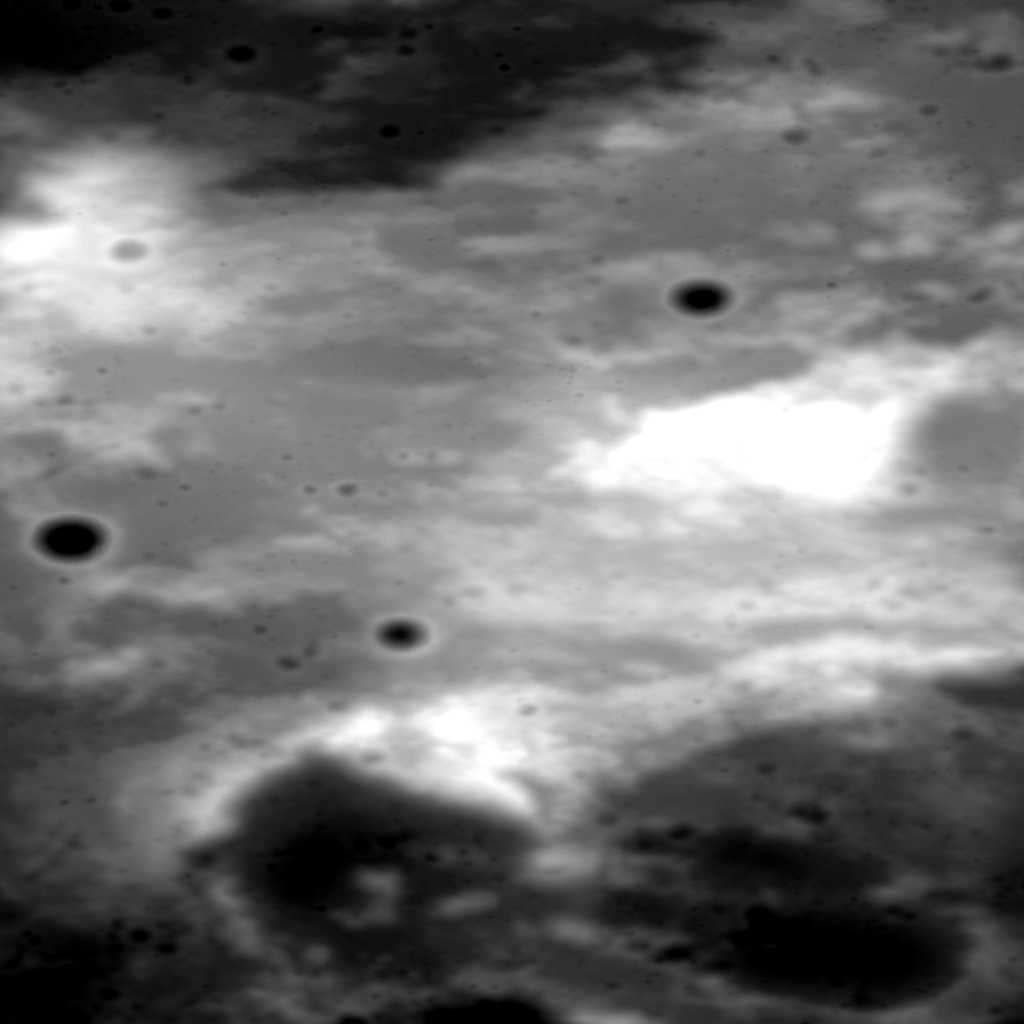

Supplement: Supplementary file 1 [file sensors-26-04344-s001.zip › data/images/test/tile_00315_lon45.0_lat51.0.png]

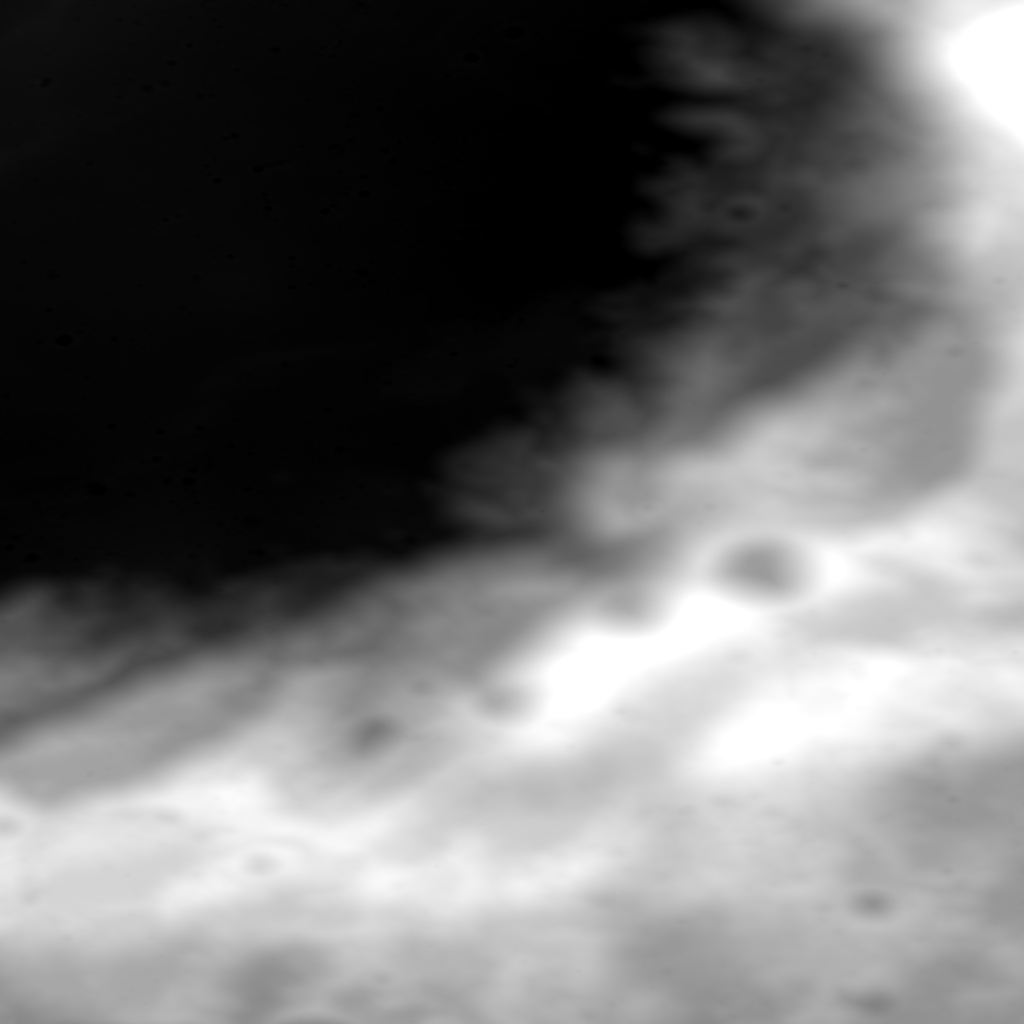

Supplement: Supplementary file 1 [file sensors-26-04344-s001.zip › data/images/test/tile_00319_lon57.0_lat51.0.png]

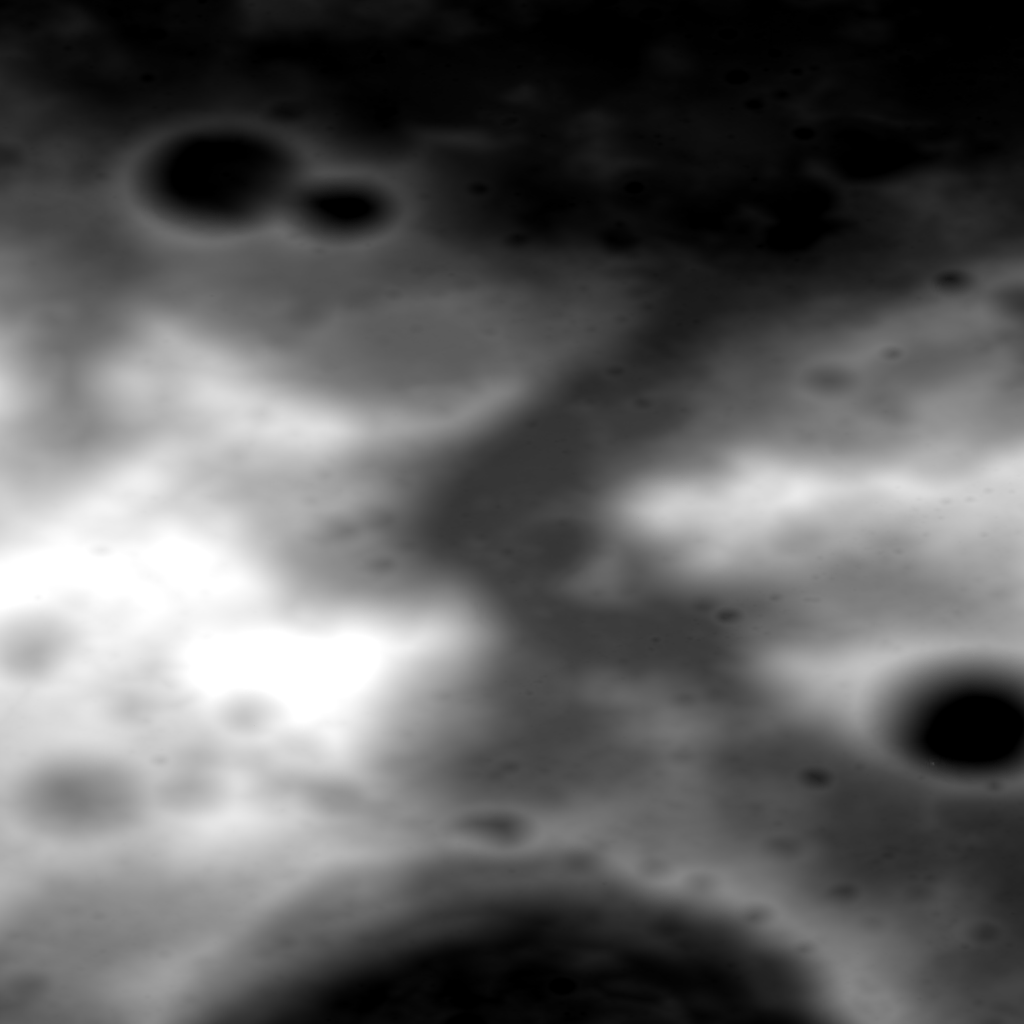

Supplement: Supplementary file 1 [file sensors-26-04344-s001.zip › data/images/test/tile_00327_lon81.0_lat51.0.png]

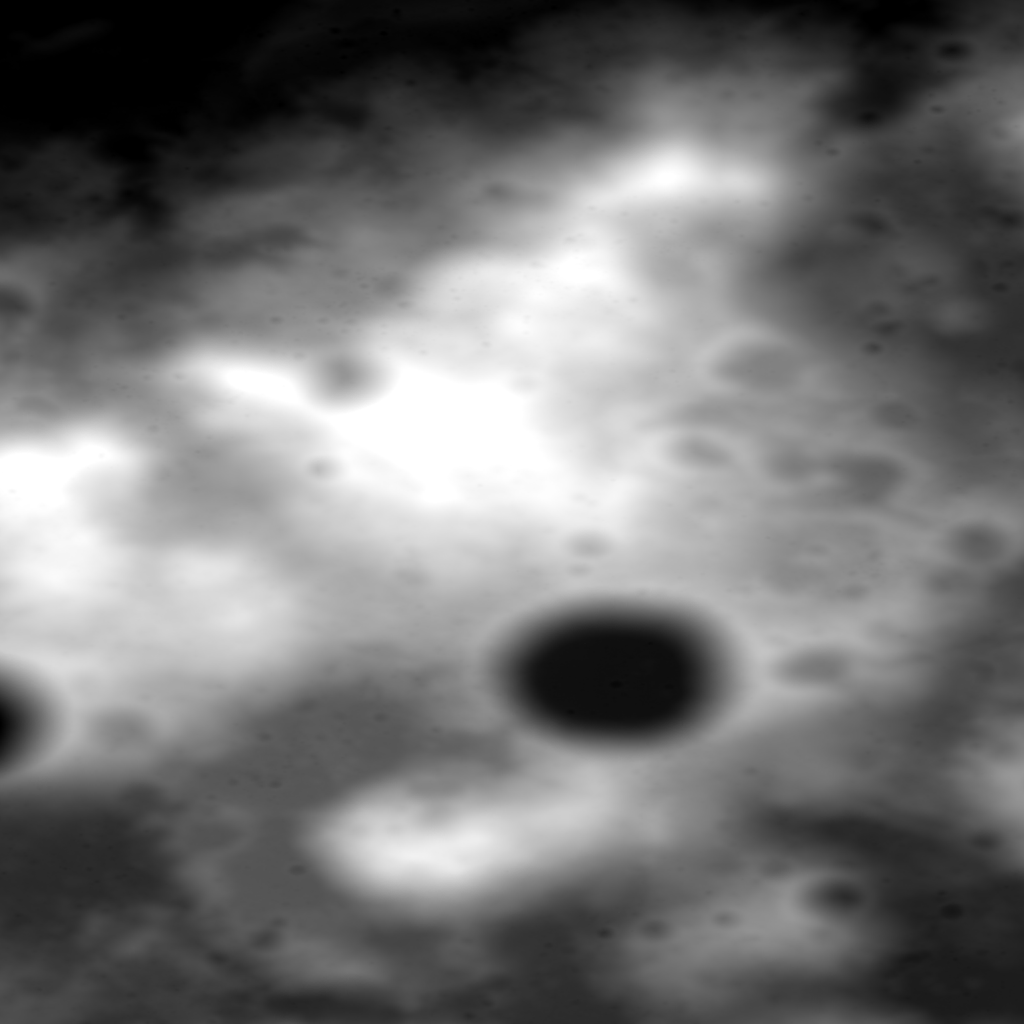

Supplement: Supplementary file 1 [file sensors-26-04344-s001.zip › data/images/test/tile_00328_lon84.0_lat51.0.png]

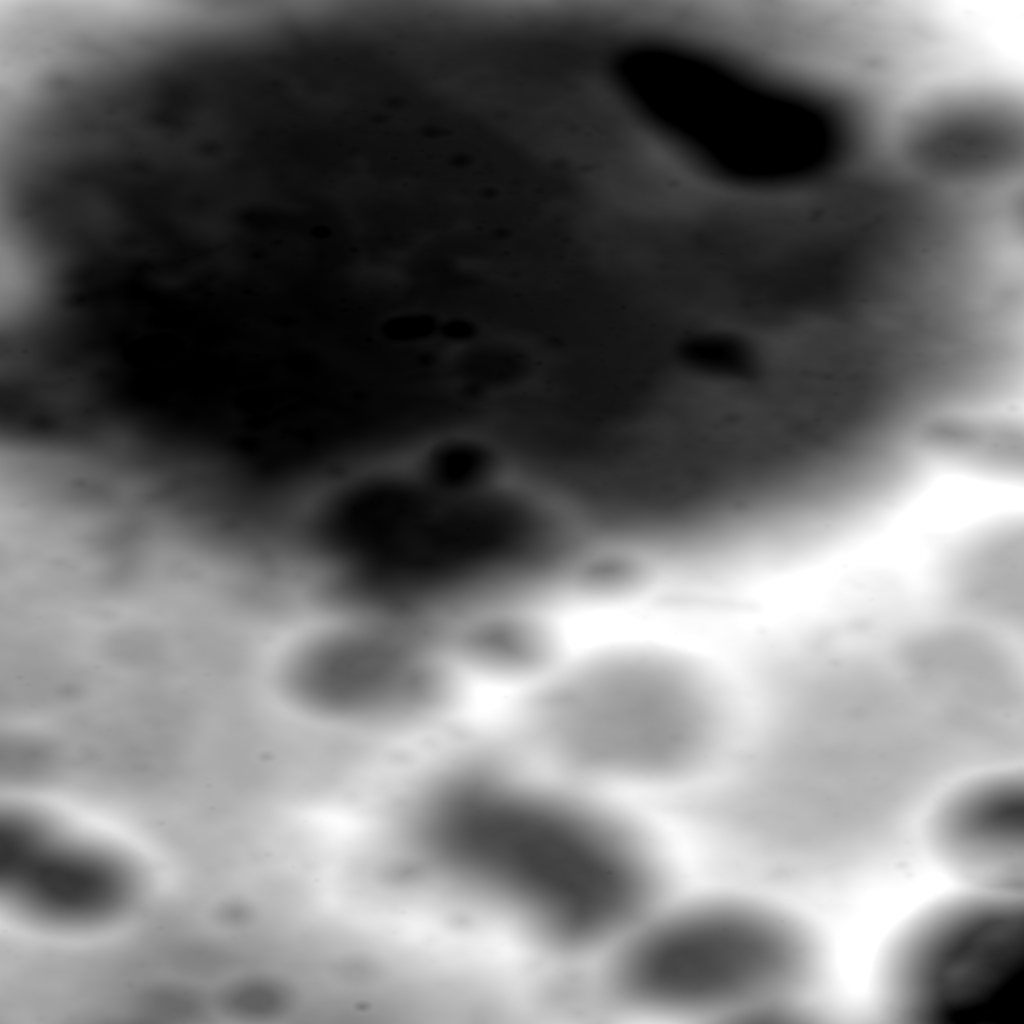

Supplement: Supplementary file 1 [file sensors-26-04344-s001.zip › data/images/test/tile_00344_lon132.0_lat51.0.png]

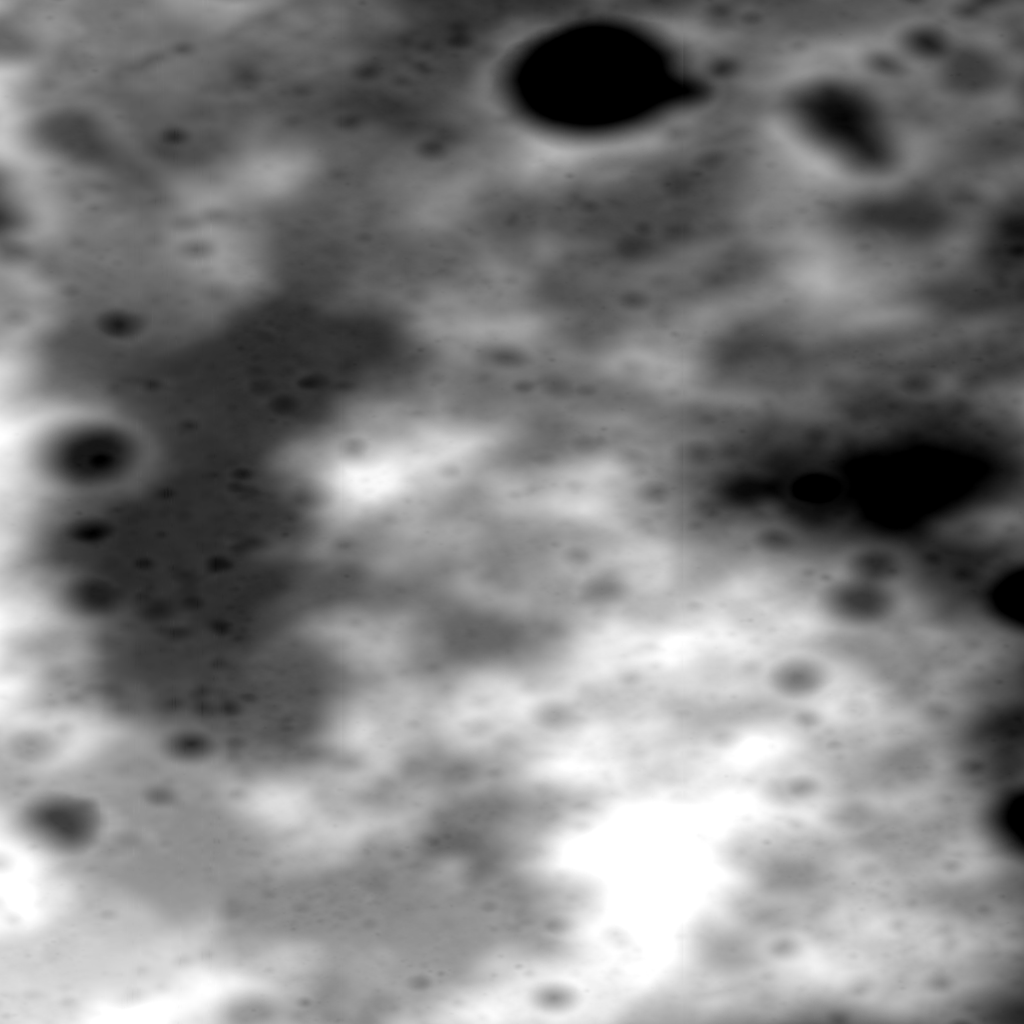

Supplement: Supplementary file 1 [file sensors-26-04344-s001.zip › data/images/test/tile_00357_lon171.0_lat51.0.png]

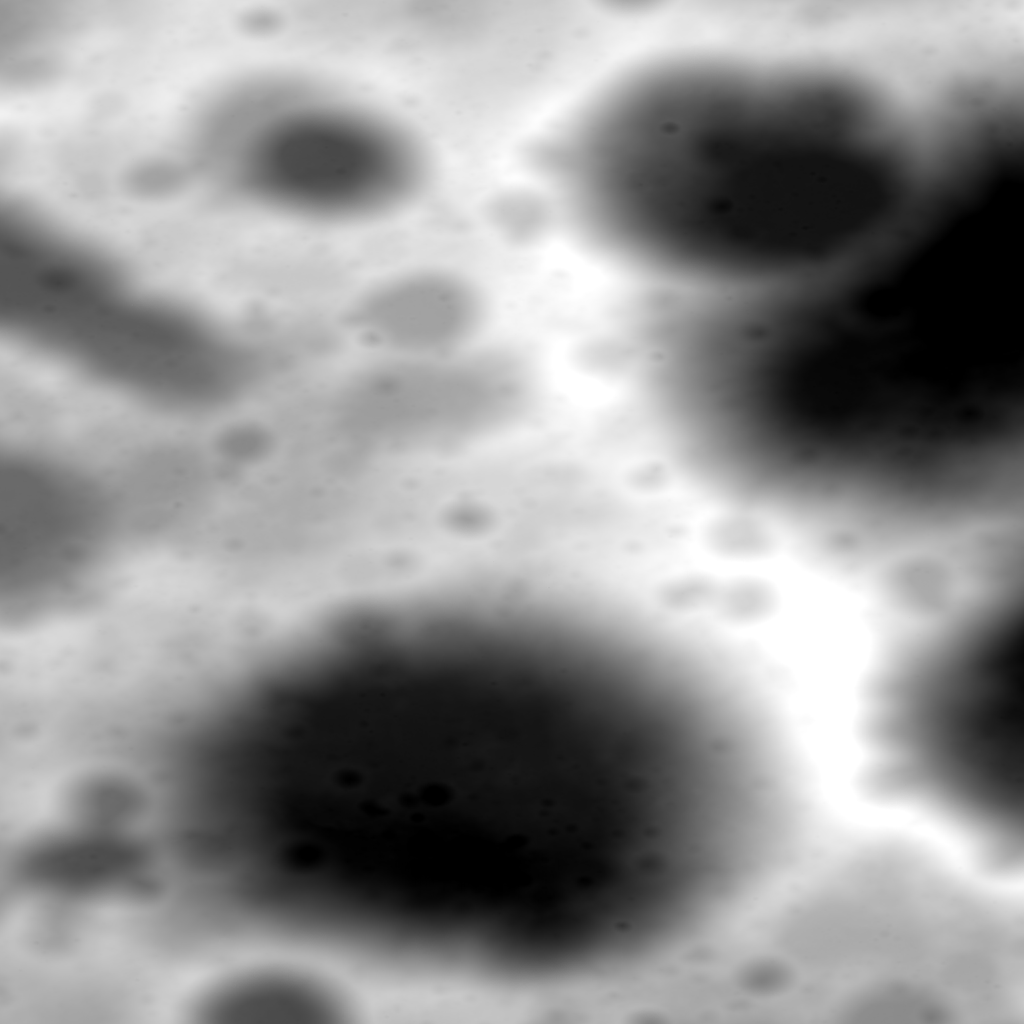

Supplement: Supplementary file 1 [file sensors-26-04344-s001.zip › data/images/test/tile_00364_lon-168.0_lat48.0.png]

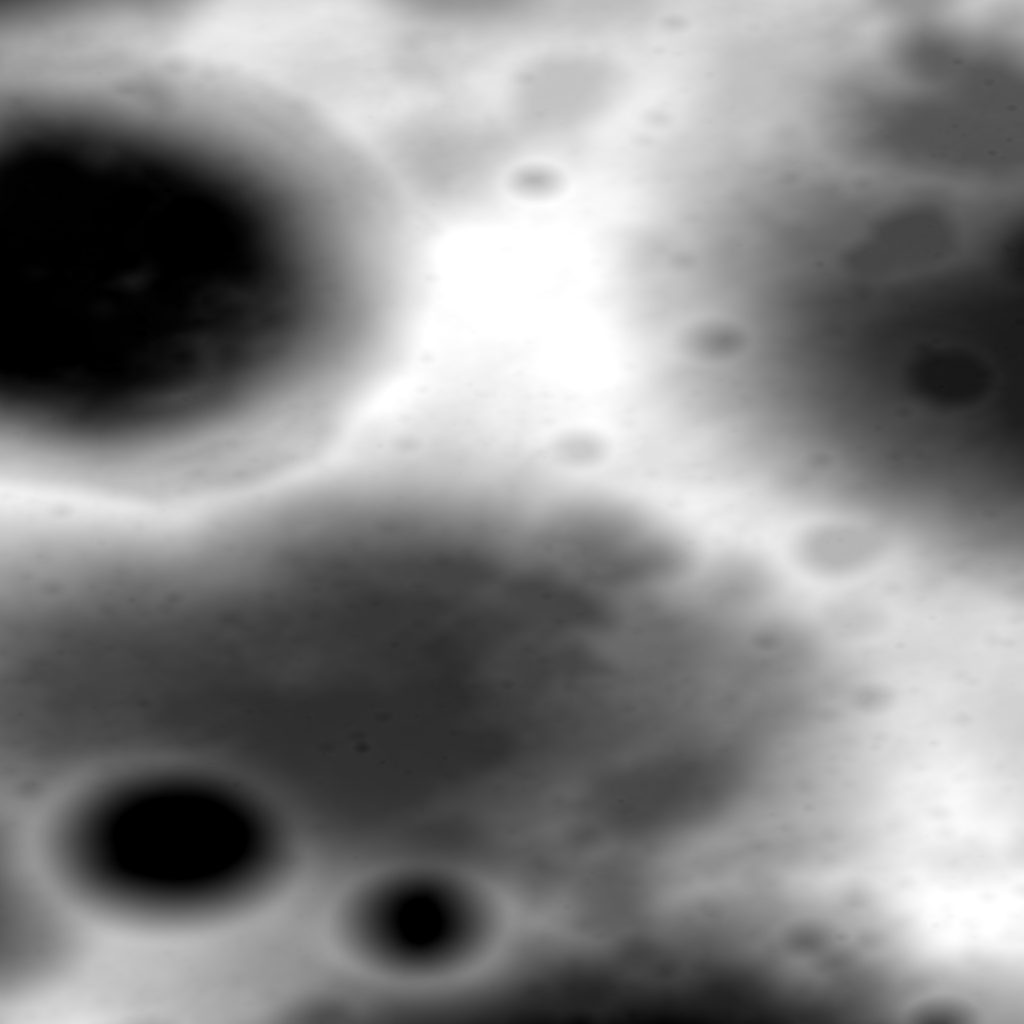

Supplement: Supplementary file 1 [file sensors-26-04344-s001.zip › data/images/test/tile_00366_lon-162.0_lat48.0.png]

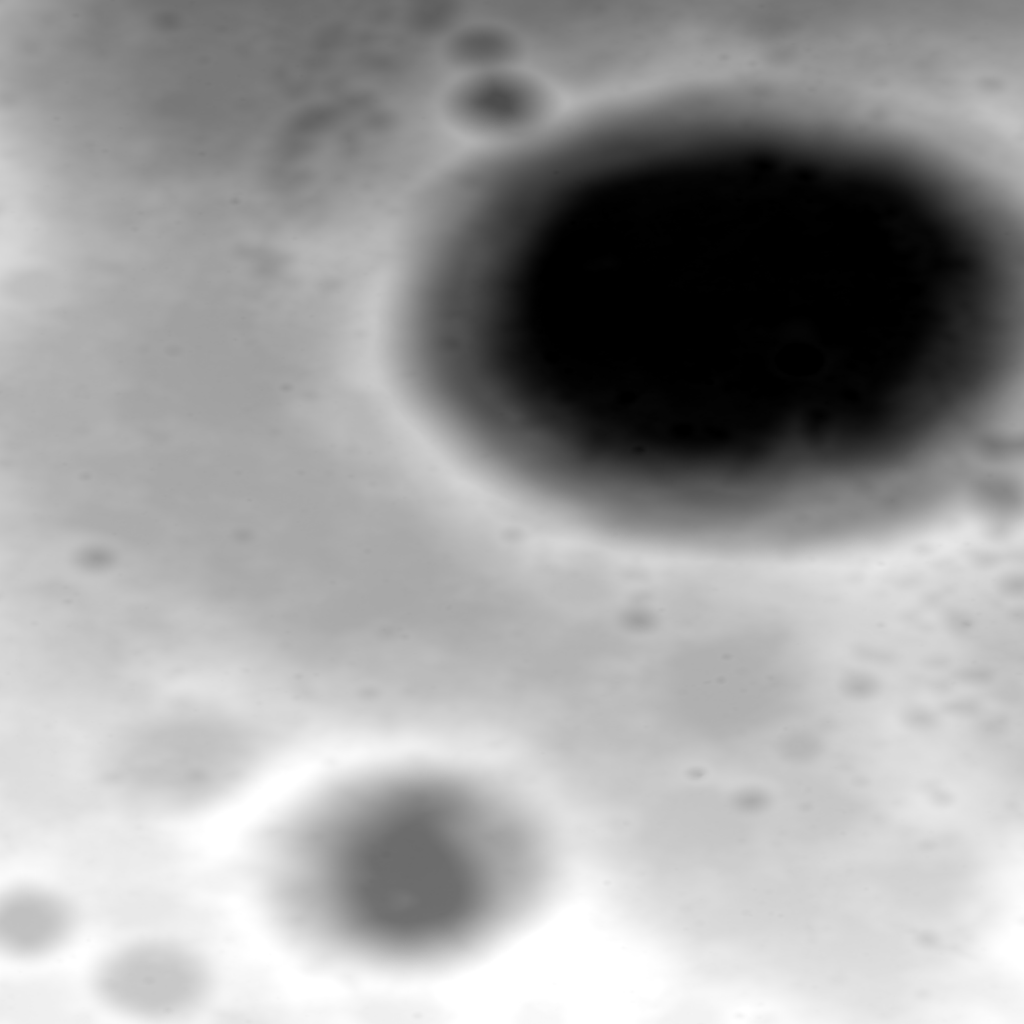

Supplement: Supplementary file 1 [file sensors-26-04344-s001.zip › data/images/test/tile_00378_lon-126.0_lat48.0.png]

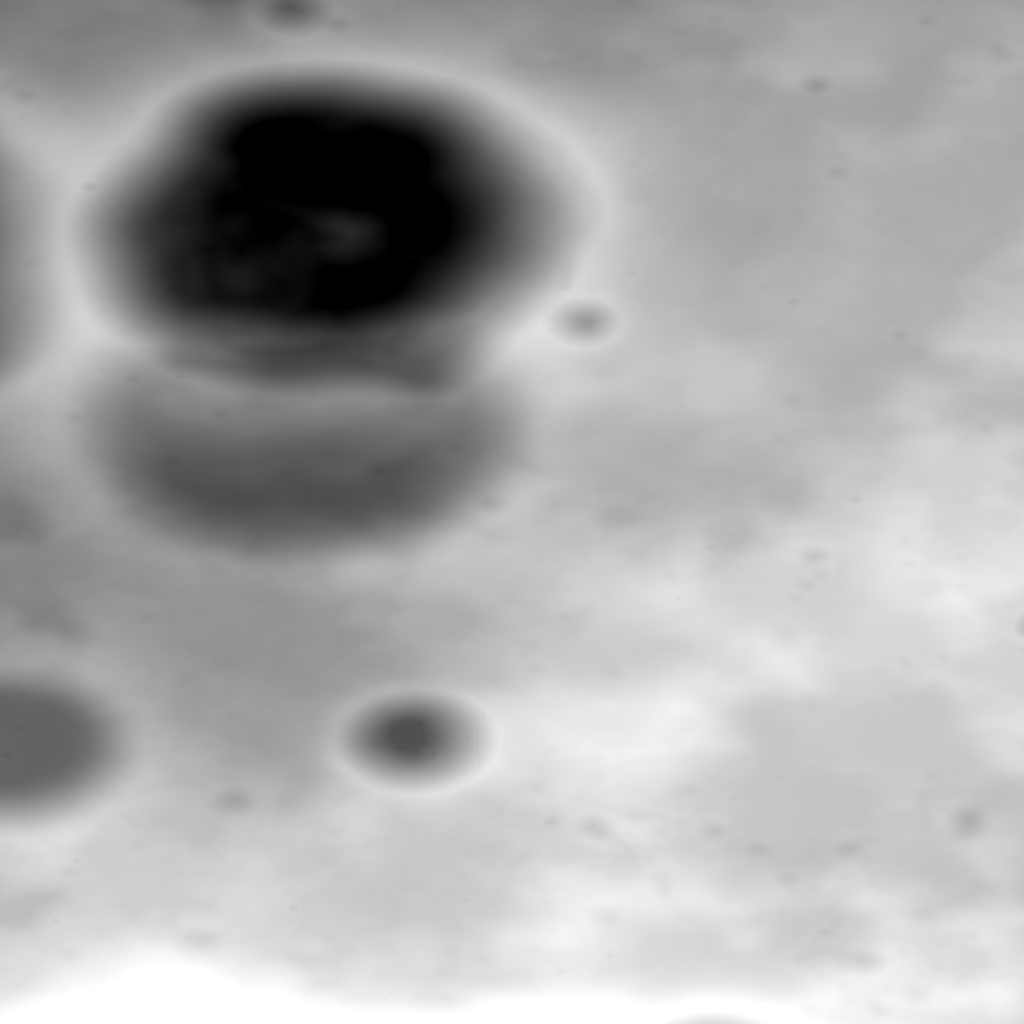

Supplement: Supplementary file 1 [file sensors-26-04344-s001.zip › data/images/test/tile_00381_lon-117.0_lat48.0.png]

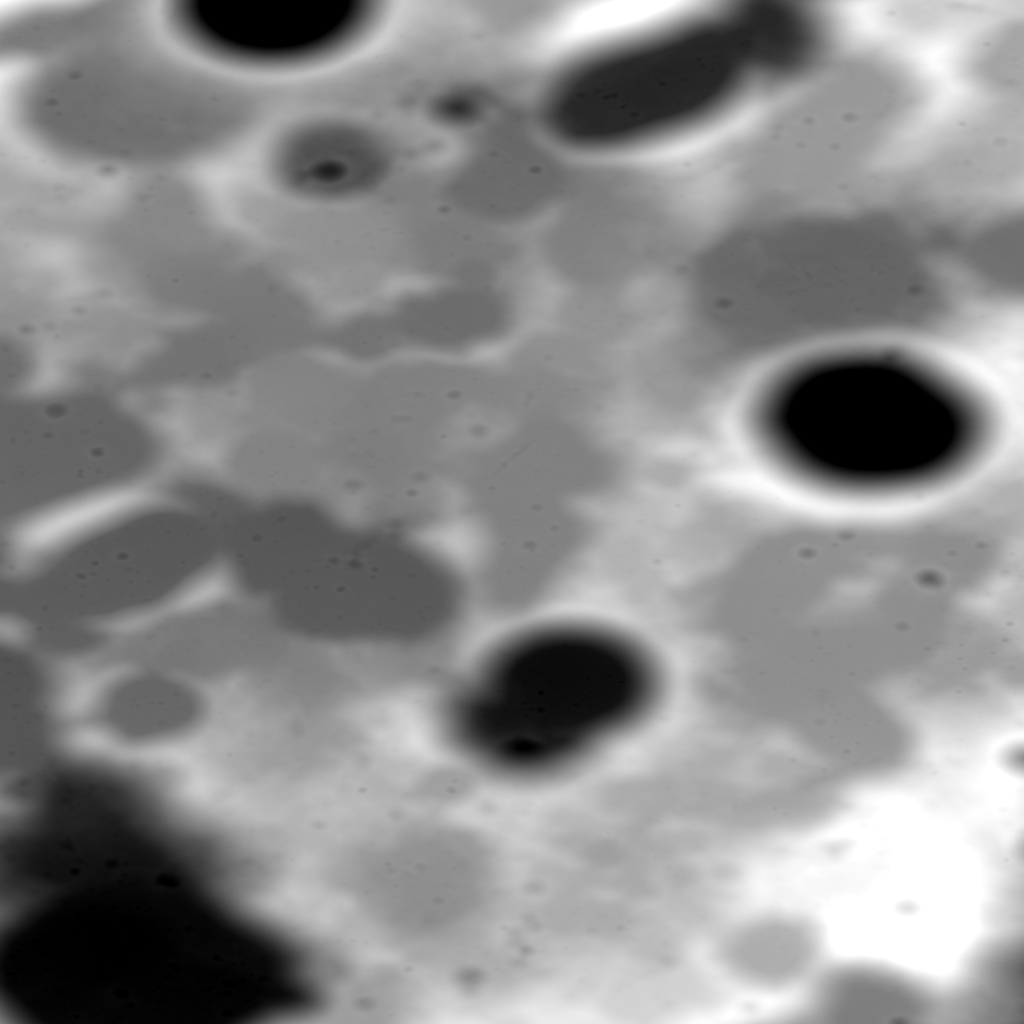

Supplement: Supplementary file 1 [file sensors-26-04344-s001.zip › data/images/test/tile_00388_lon-96.0_lat48.0.png]

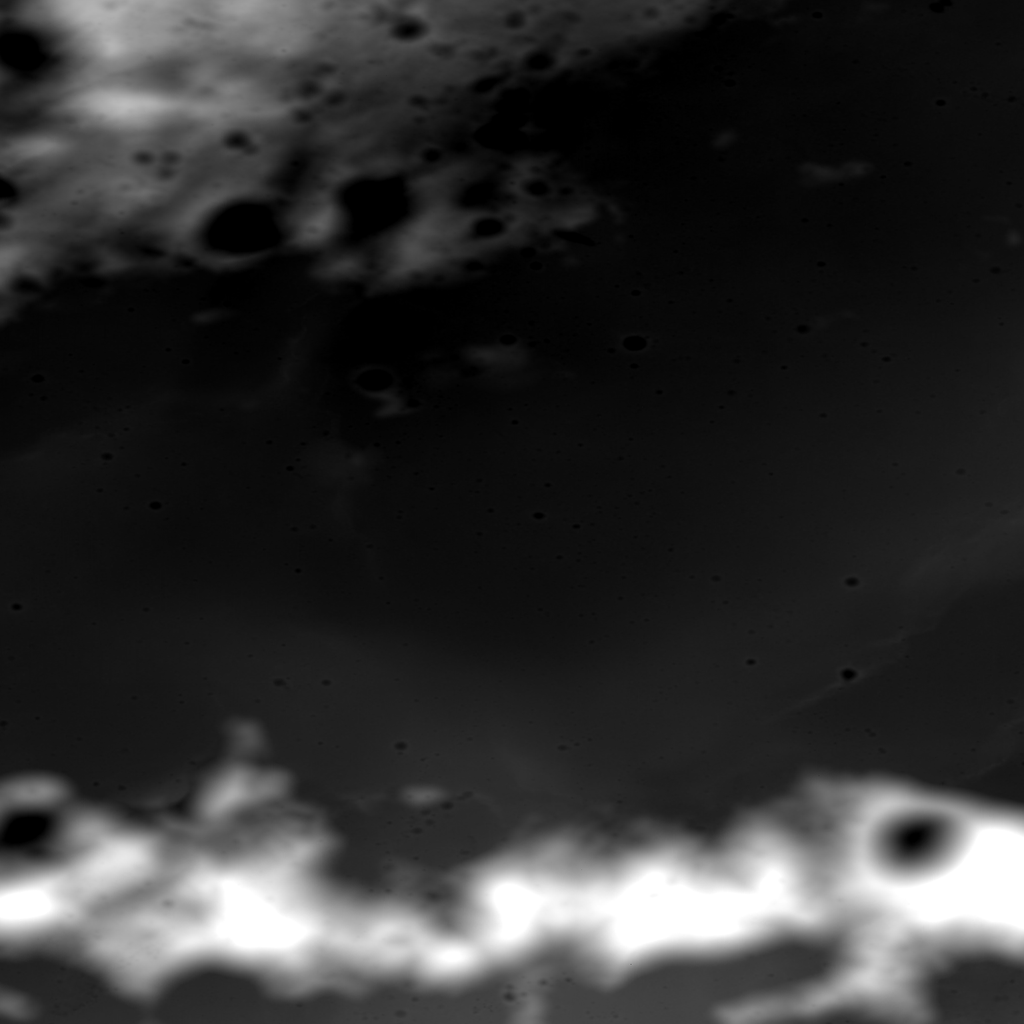

Supplement: Supplementary file 1 [file sensors-26-04344-s001.zip › data/images/test/tile_00413_lon-21.0_lat48.0.png]

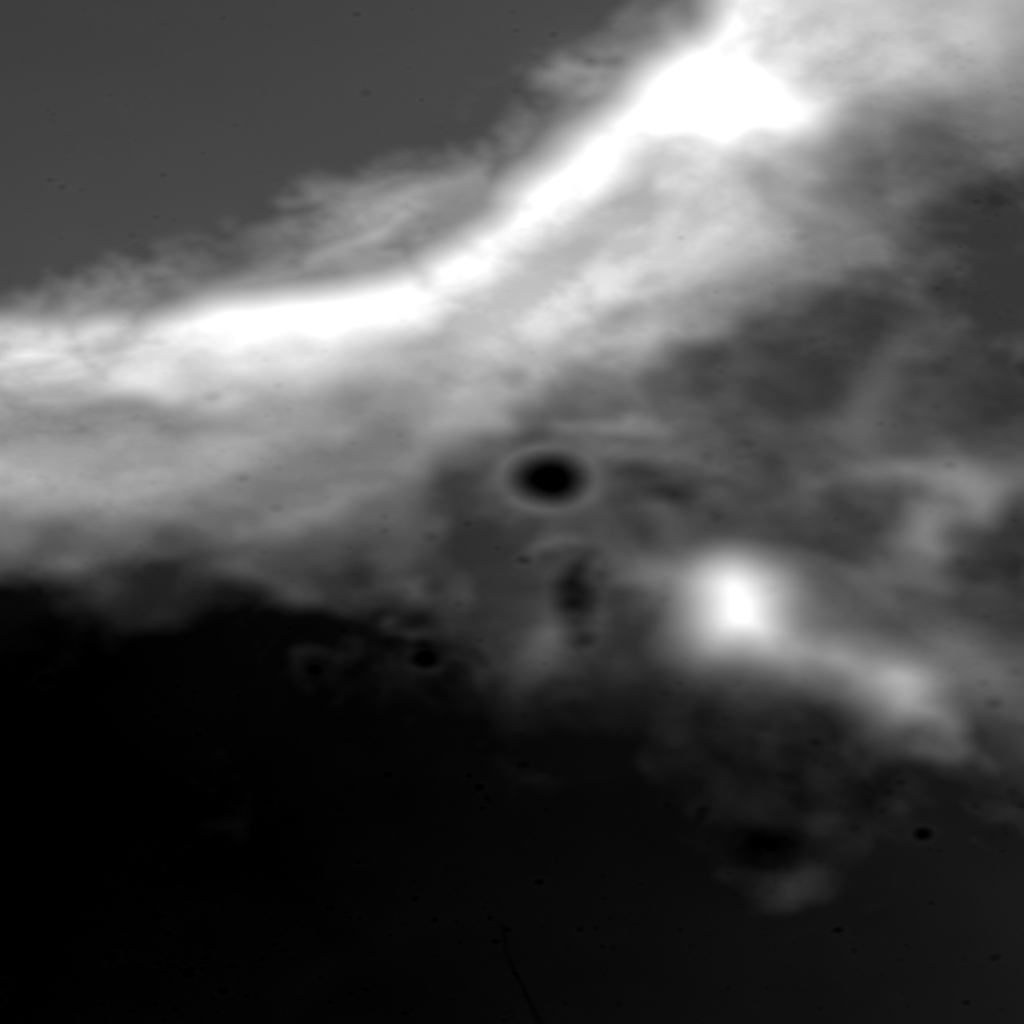

Supplement: Supplementary file 1 [file sensors-26-04344-s001.zip › data/images/test/tile_00417_lon-9.0_lat48.0.png]

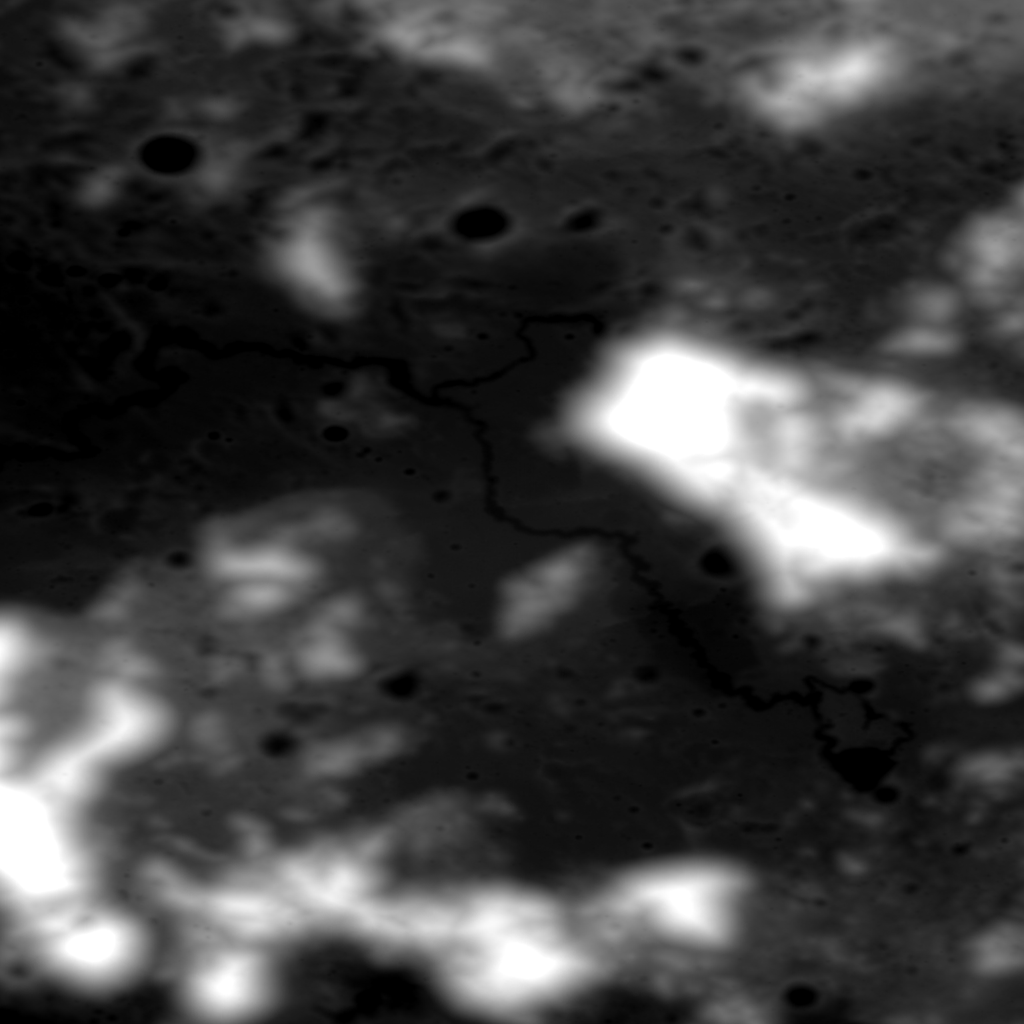

Supplement: Supplementary file 1 [file sensors-26-04344-s001.zip › data/images/test/tile_00419_lon-3.0_lat48.0.png]

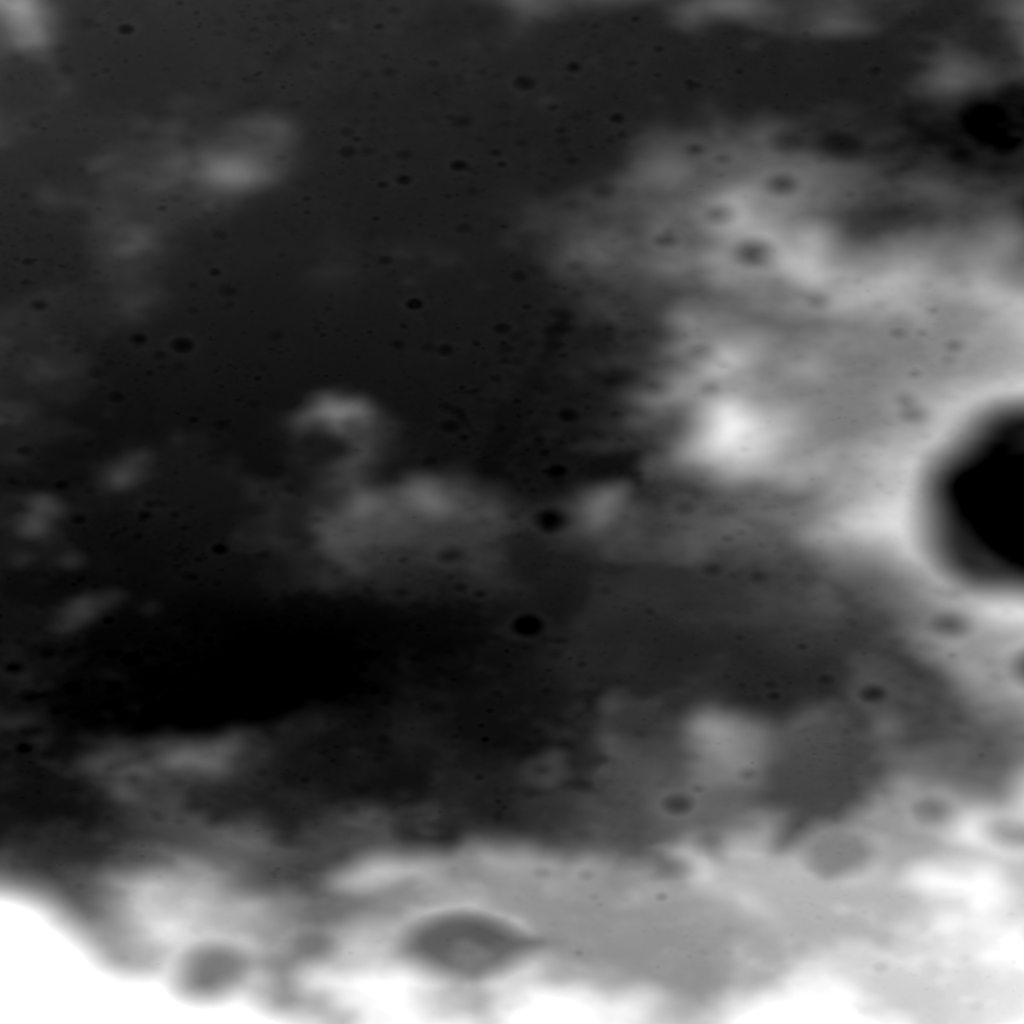

Supplement: Supplementary file 1 [file sensors-26-04344-s001.zip › data/images/test/tile_00446_lon78.0_lat48.0.png]

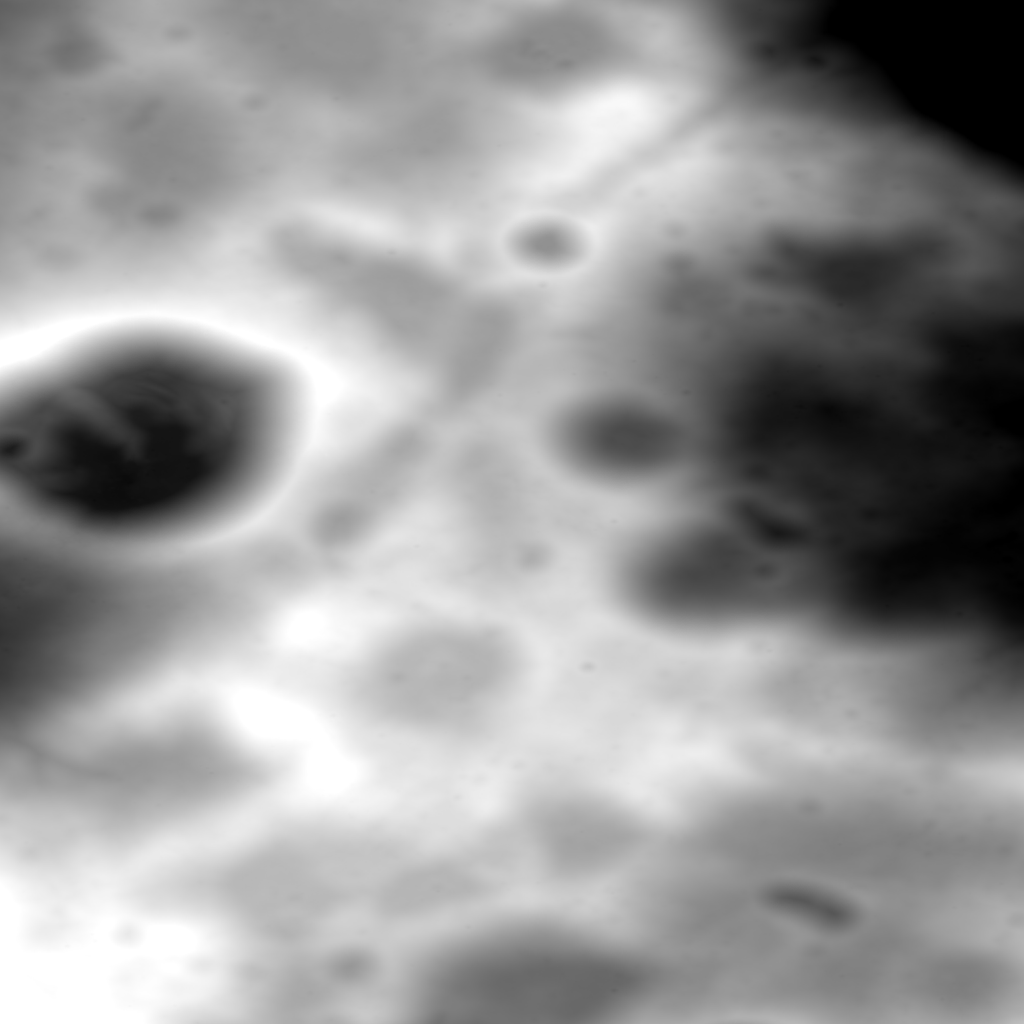

Supplement: Supplementary file 1 [file sensors-26-04344-s001.zip › data/images/test/tile_00461_lon123.0_lat48.0.png]

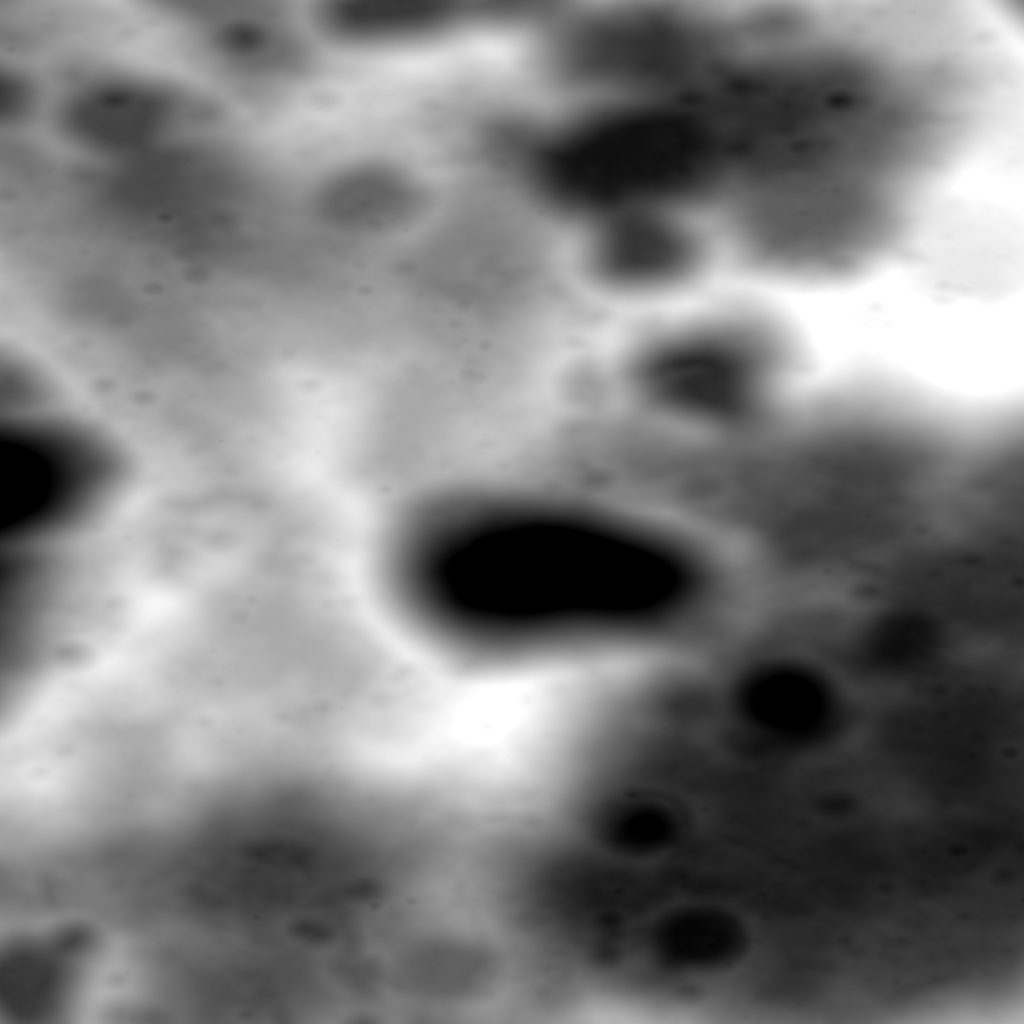

Supplement: Supplementary file 1 [file sensors-26-04344-s001.zip › data/images/test/tile_00468_lon144.0_lat48.0.png]

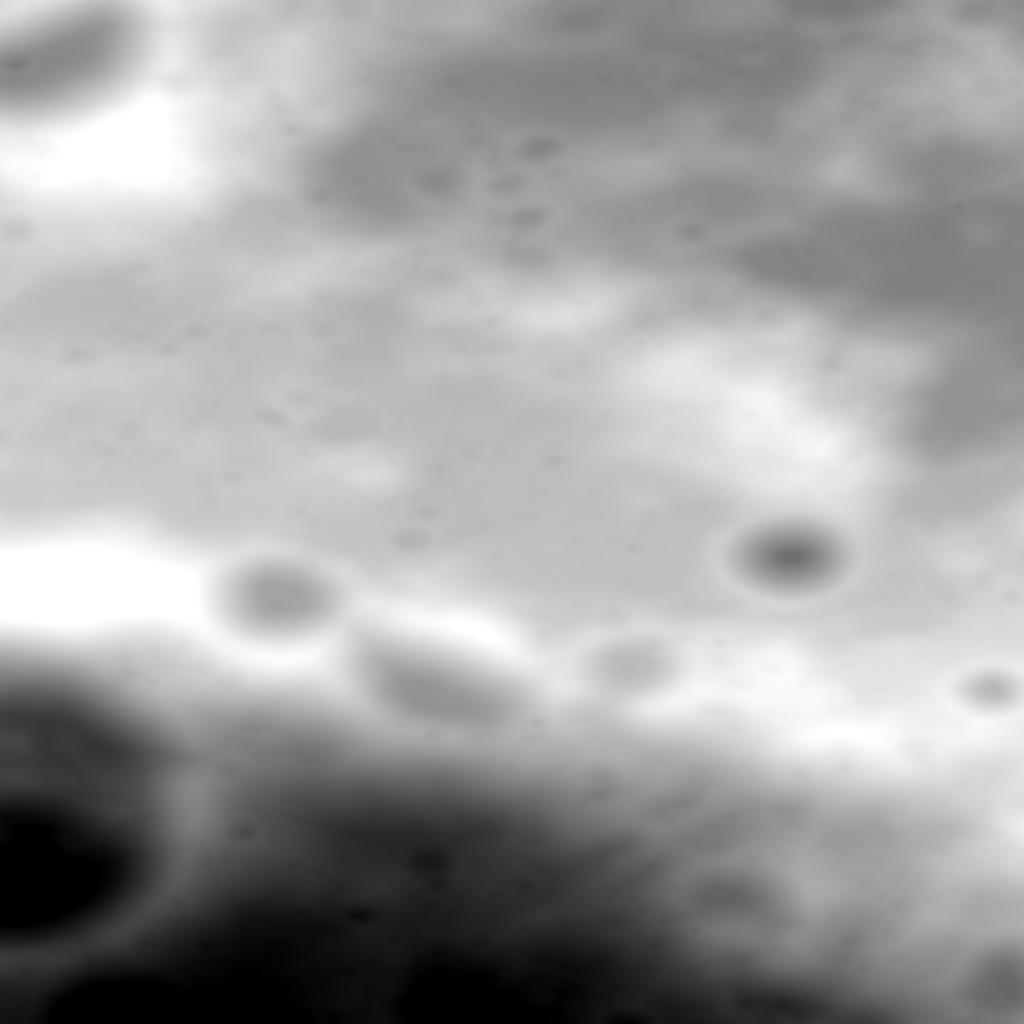

Supplement: Supplementary file 1 [file sensors-26-04344-s001.zip › data/images/test/tile_00471_lon153.0_lat48.0.png]

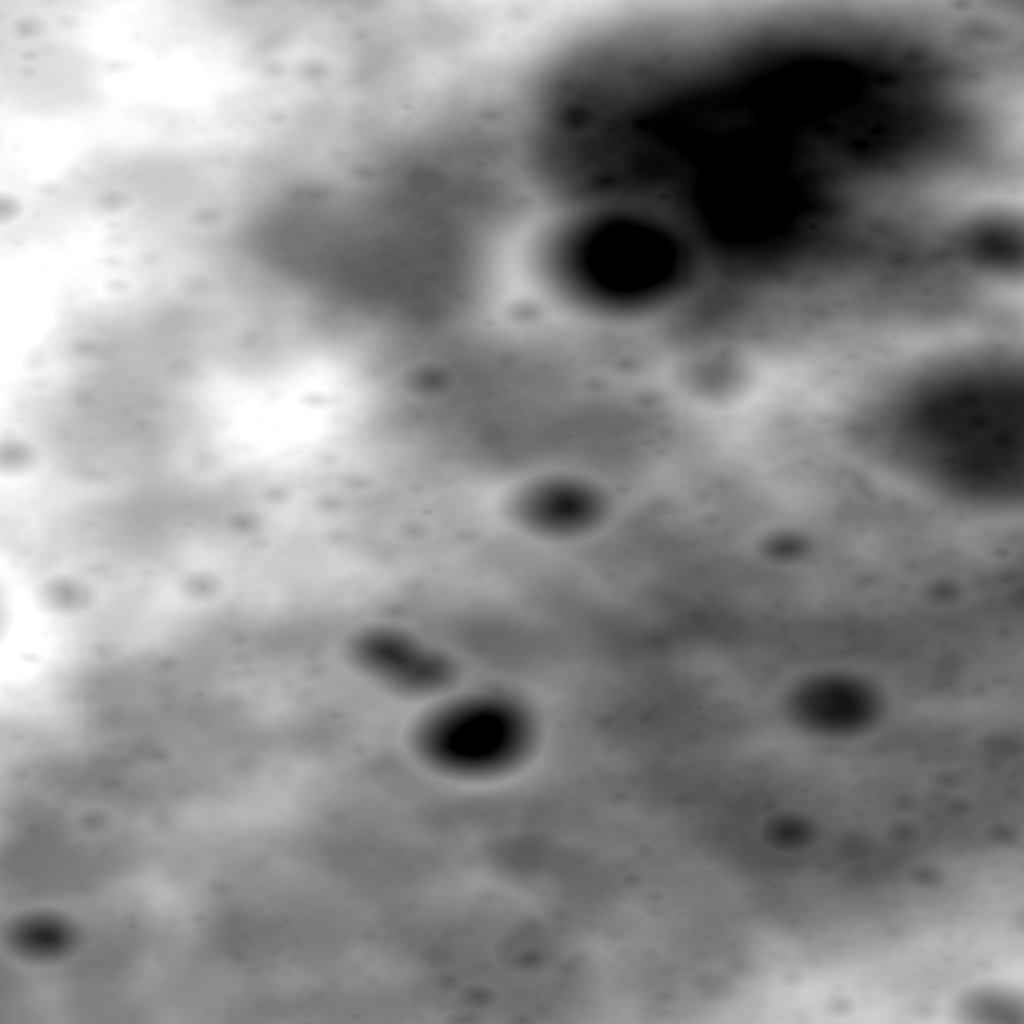

Supplement: Supplementary file 1 [file sensors-26-04344-s001.zip › data/images/test/tile_00477_lon171.0_lat48.0.png]

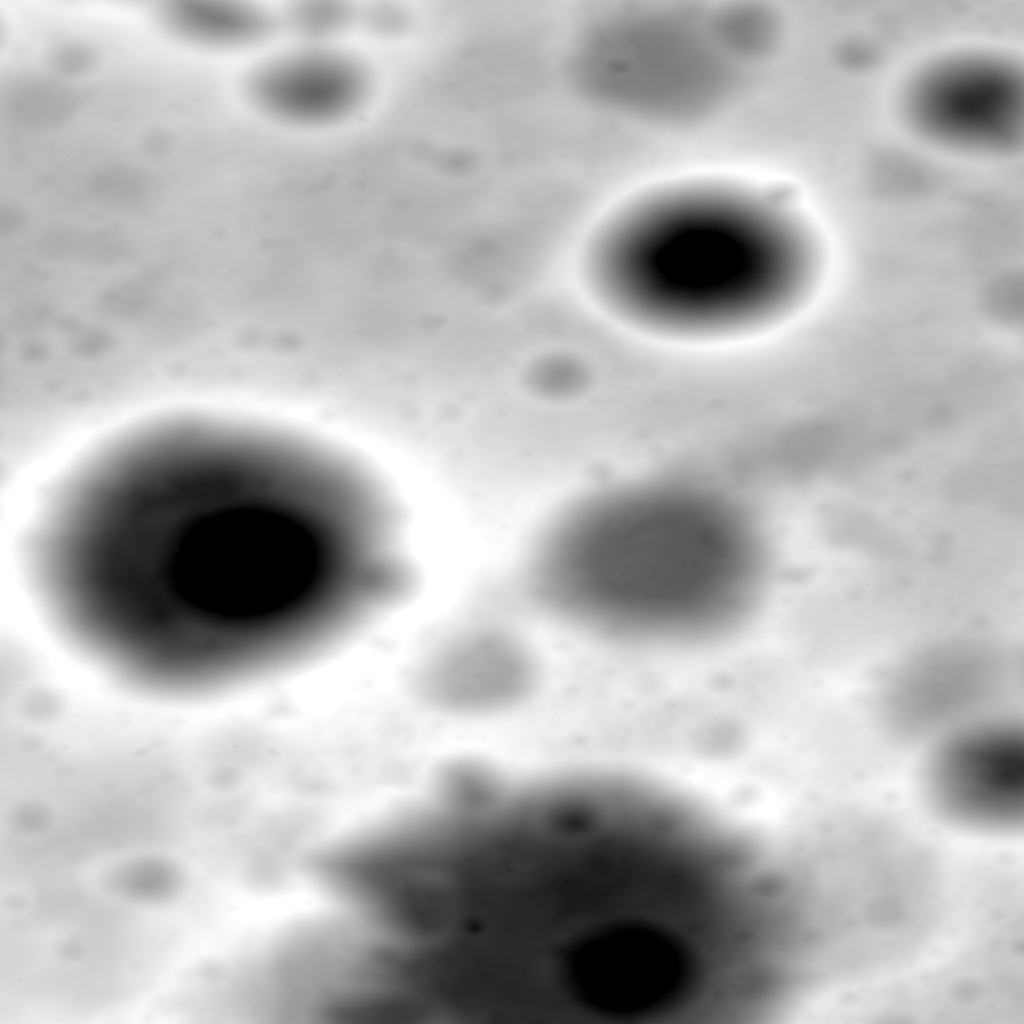

Supplement: Supplementary file 1 [file sensors-26-04344-s001.zip › data/images/test/tile_00483_lon-171.0_lat45.0.png]

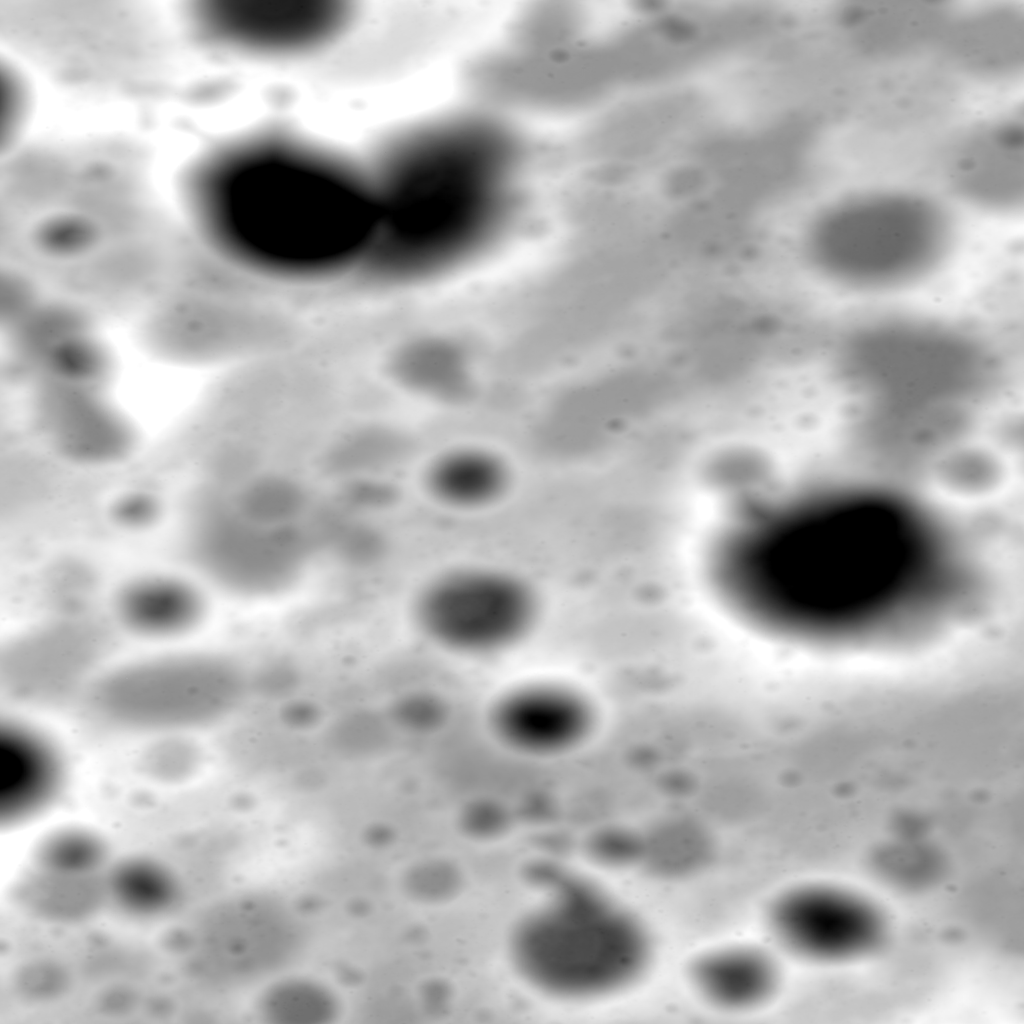

Supplement: Supplementary file 1 [file sensors-26-04344-s001.zip › data/images/test/tile_00484_lon-168.0_lat45.0.png]

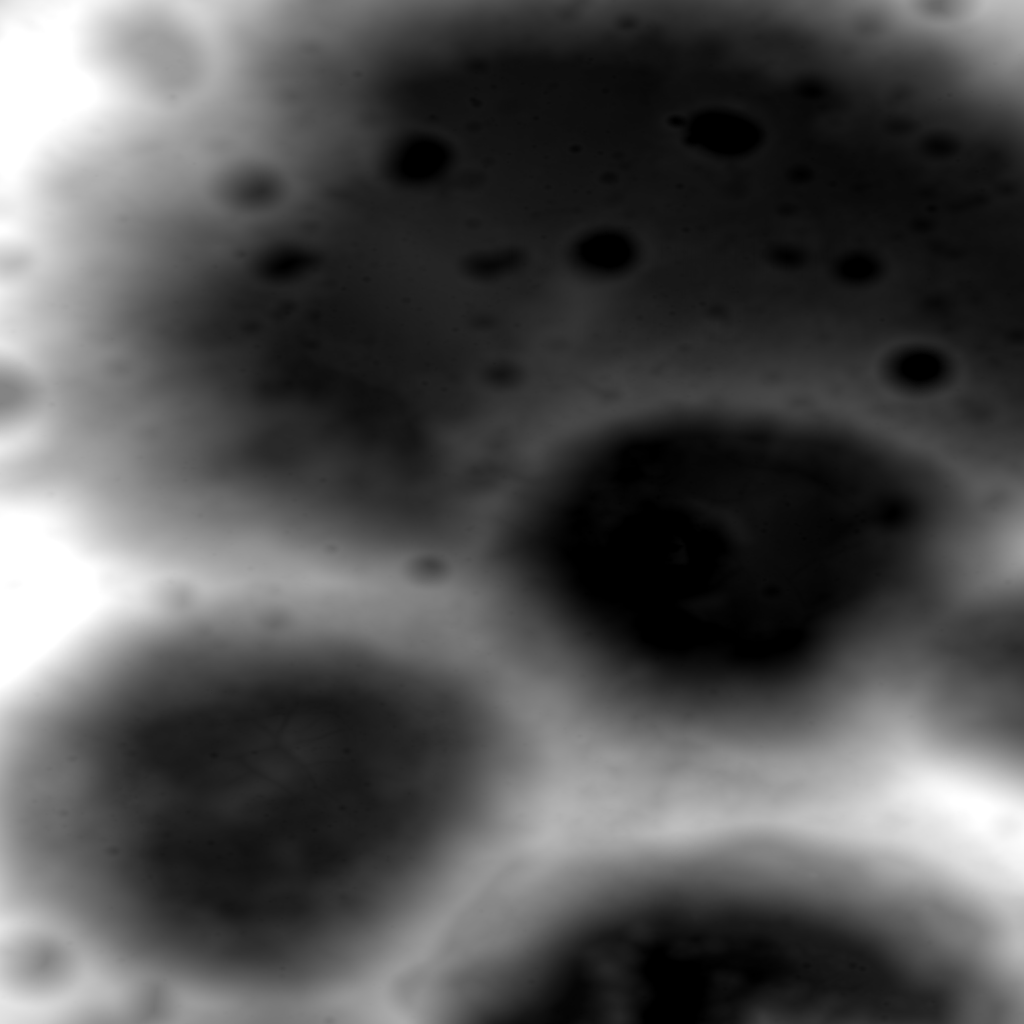

Supplement: Supplementary file 1 [file sensors-26-04344-s001.zip › data/images/test/tile_00486_lon-162.0_lat45.0.png]

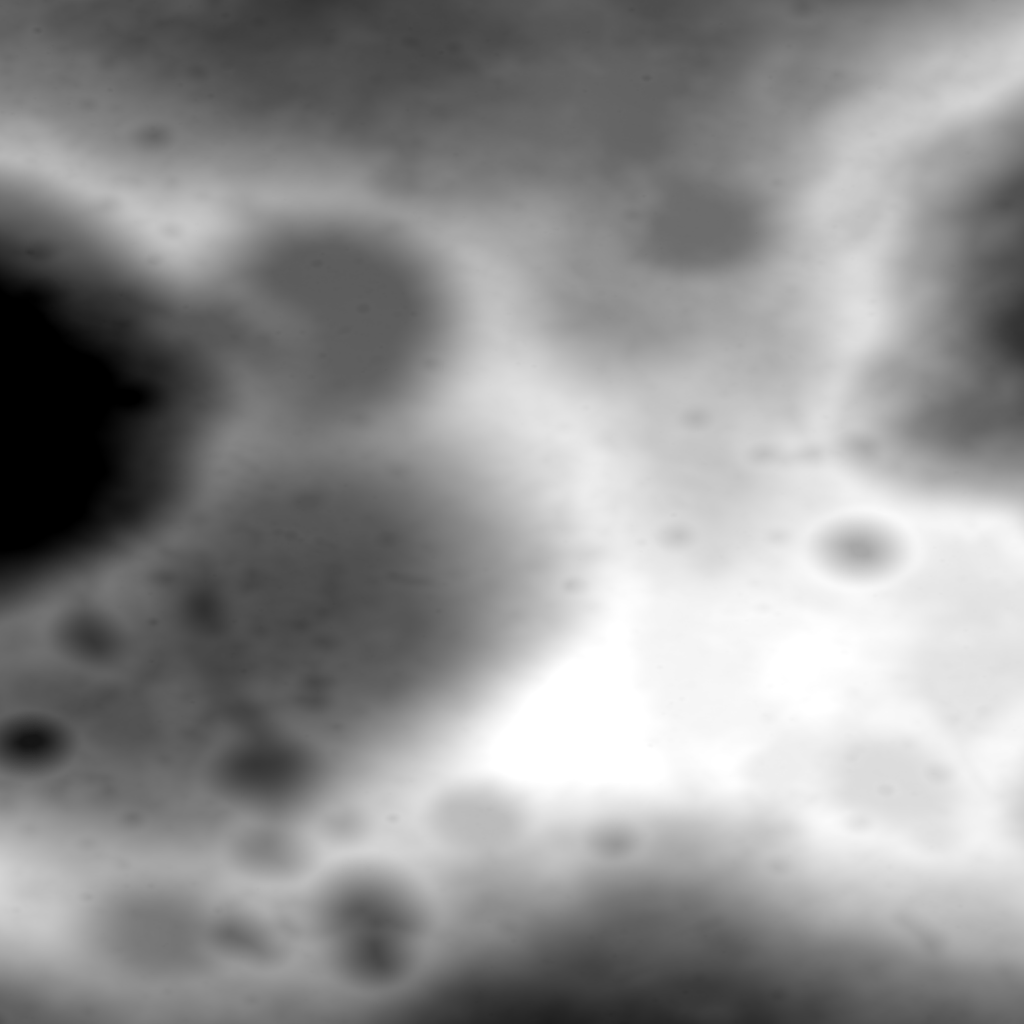

Supplement: Supplementary file 1 [file sensors-26-04344-s001.zip › data/images/test/tile_00500_lon-120.0_lat45.0.png]

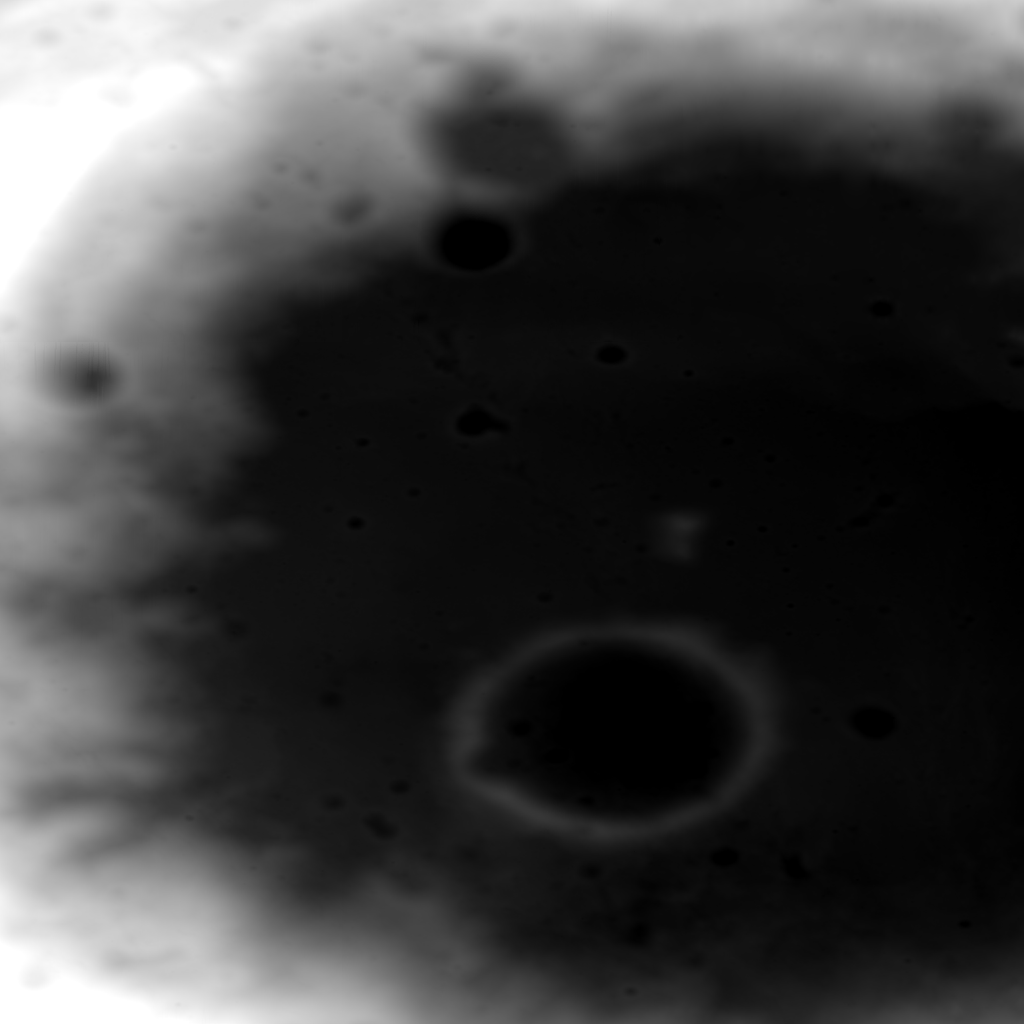

Supplement: Supplementary file 1 [file sensors-26-04344-s001.zip › data/images/test/tile_00503_lon-111.0_lat45.0.png]

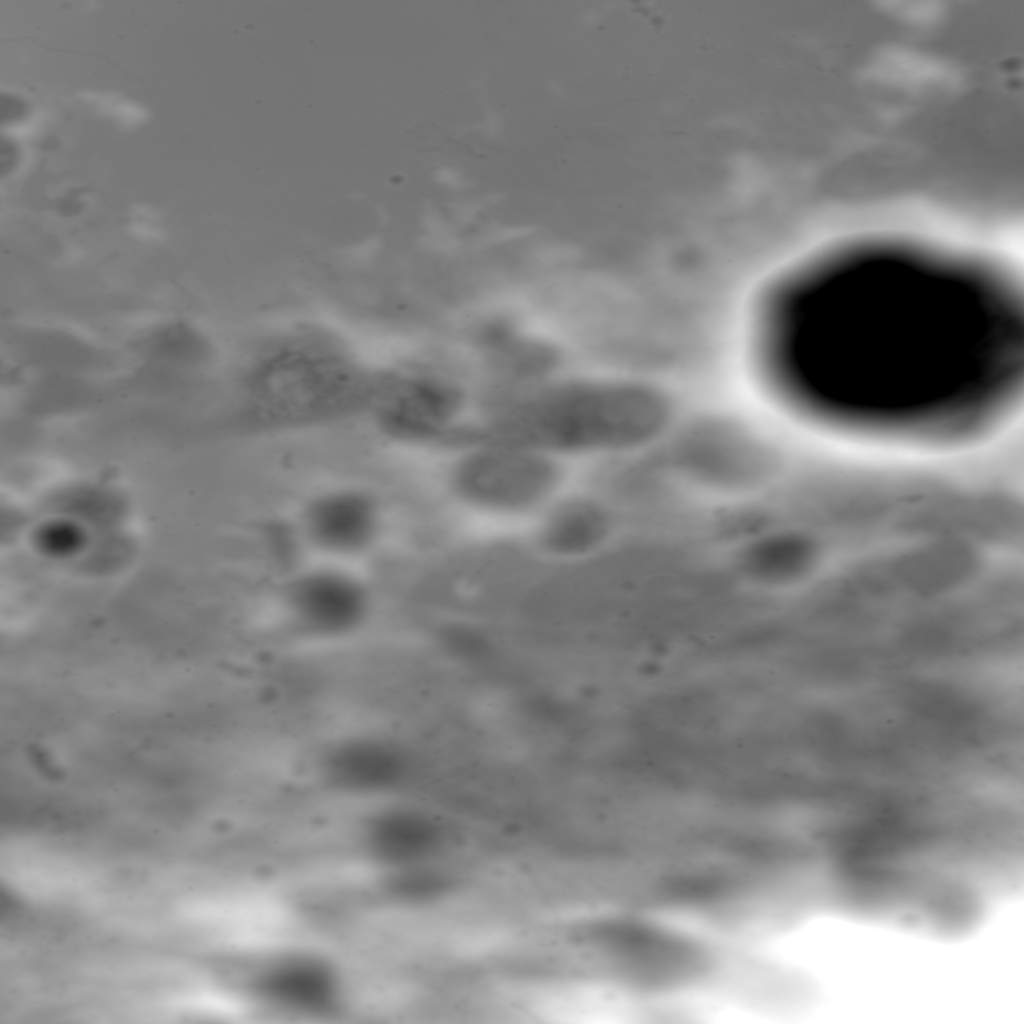

Supplement: Supplementary file 1 [file sensors-26-04344-s001.zip › data/images/test/tile_00524_lon-48.0_lat45.0.png]

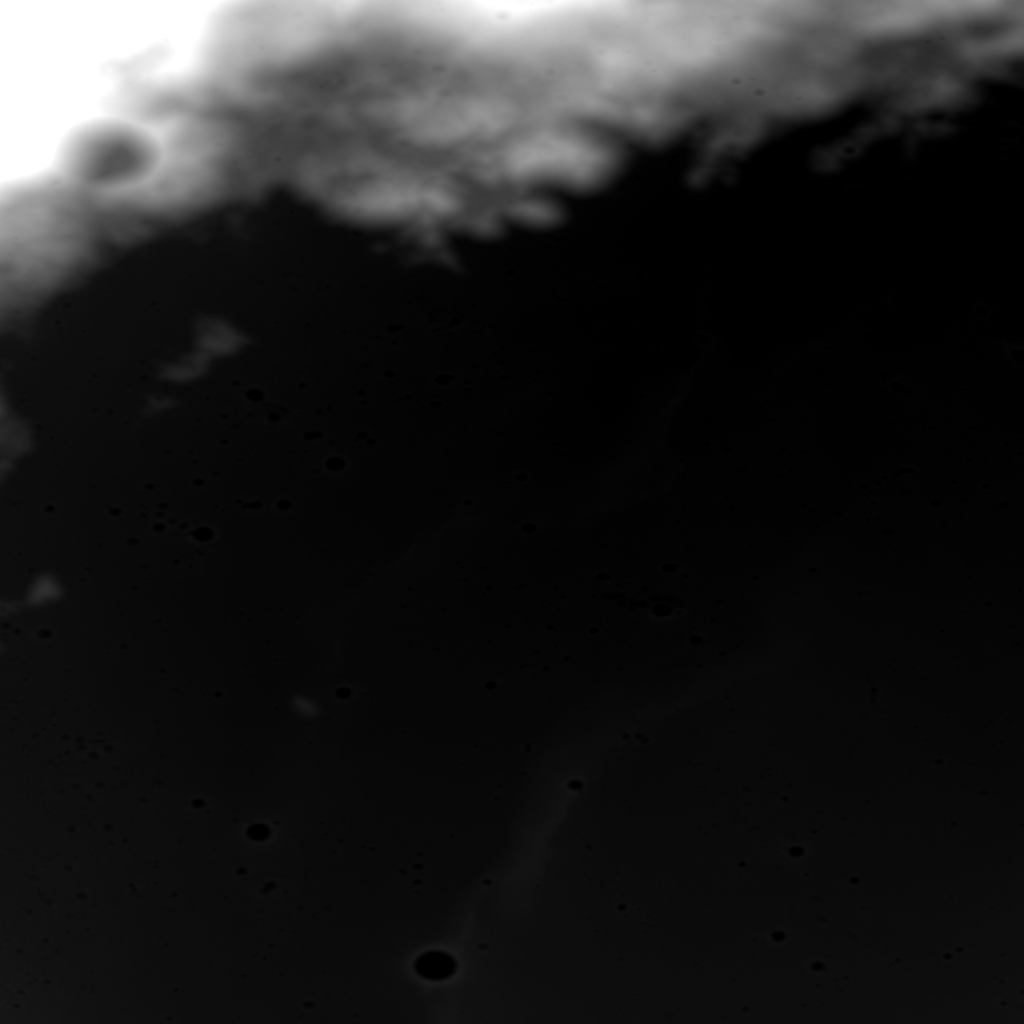

Supplement: Supplementary file 1 [file sensors-26-04344-s001.zip › data/images/test/tile_00528_lon-36.0_lat45.0.png]

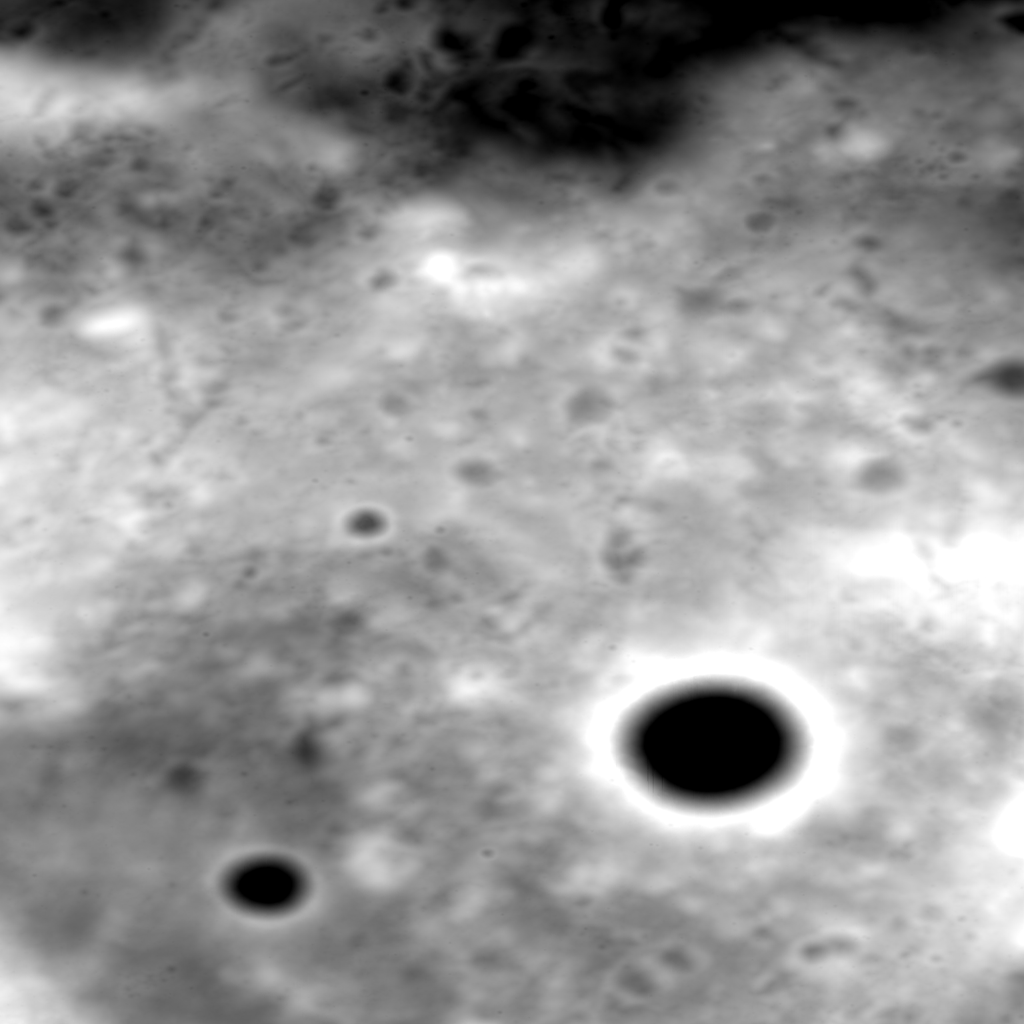

Supplement: Supplementary file 1 [file sensors-26-04344-s001.zip › data/images/test/tile_00546_lon18.0_lat45.0.png]

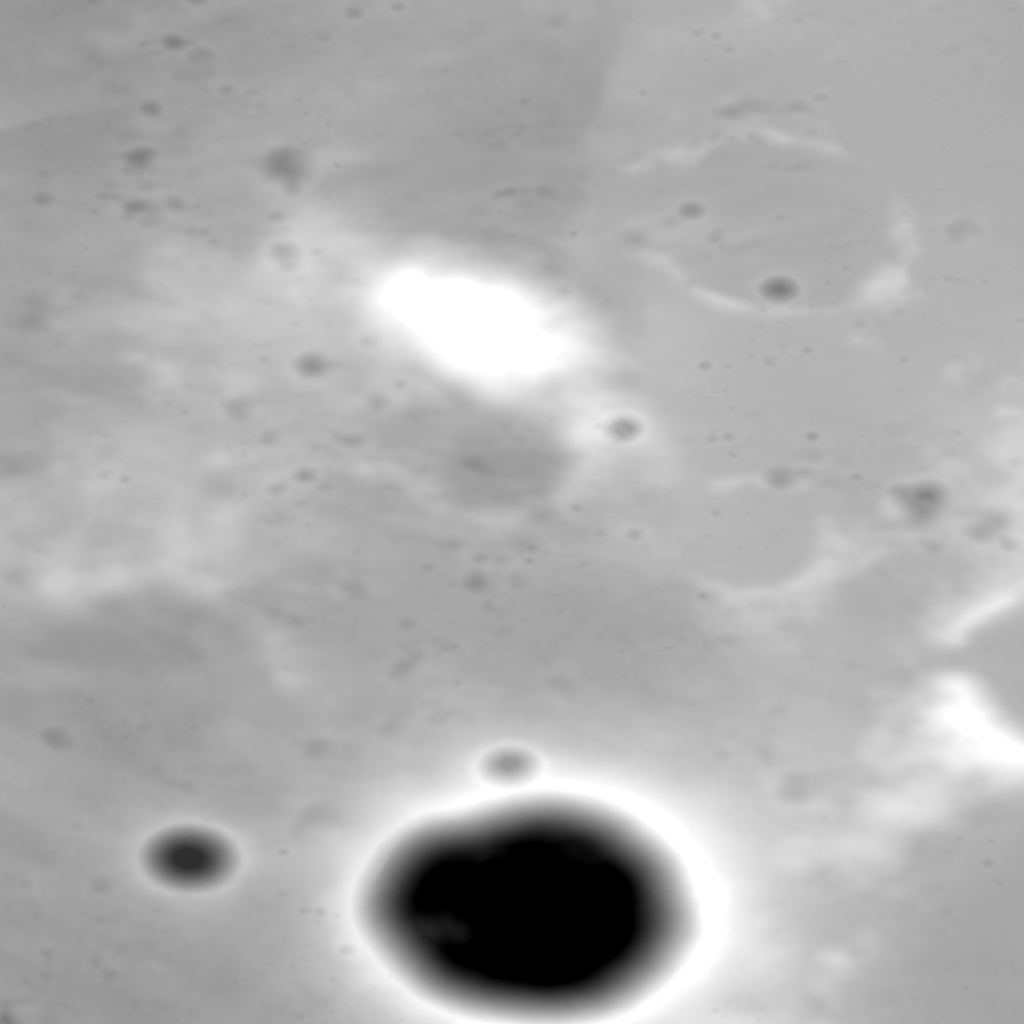

Supplement: Supplementary file 1 [file sensors-26-04344-s001.zip › data/images/test/tile_00556_lon48.0_lat45.0.png]

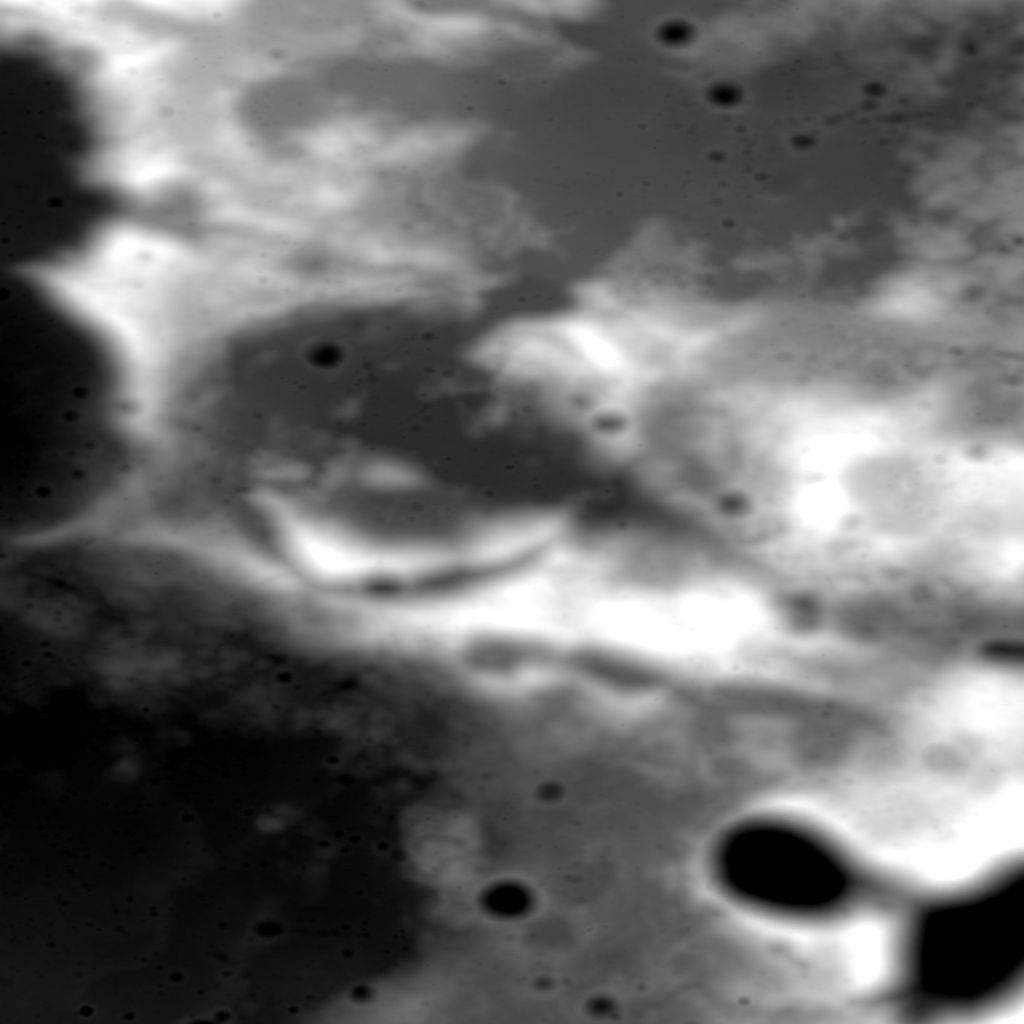

Supplement: Supplementary file 1 [file sensors-26-04344-s001.zip › data/images/test/tile_00560_lon60.0_lat45.0.png]

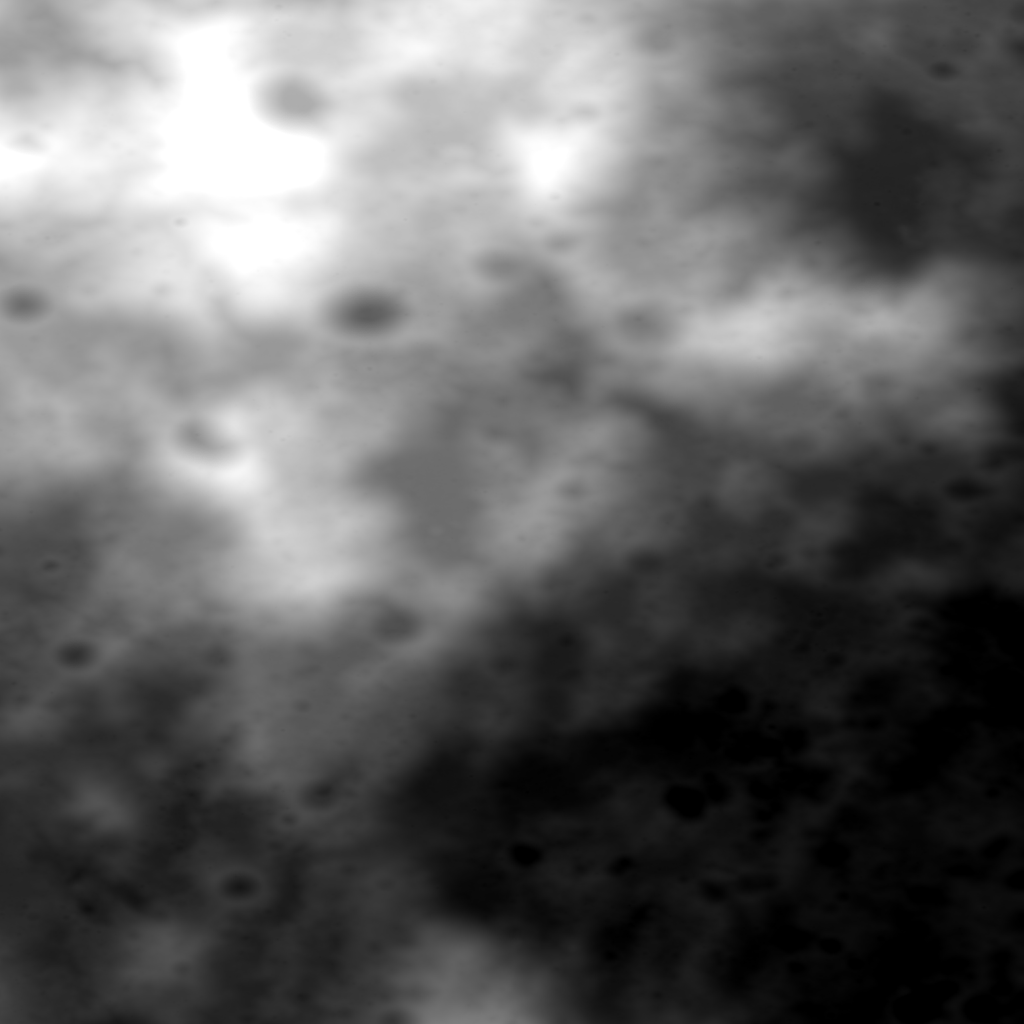

Supplement: Supplementary file 1 [file sensors-26-04344-s001.zip › data/images/test/tile_00565_lon75.0_lat45.0.png]

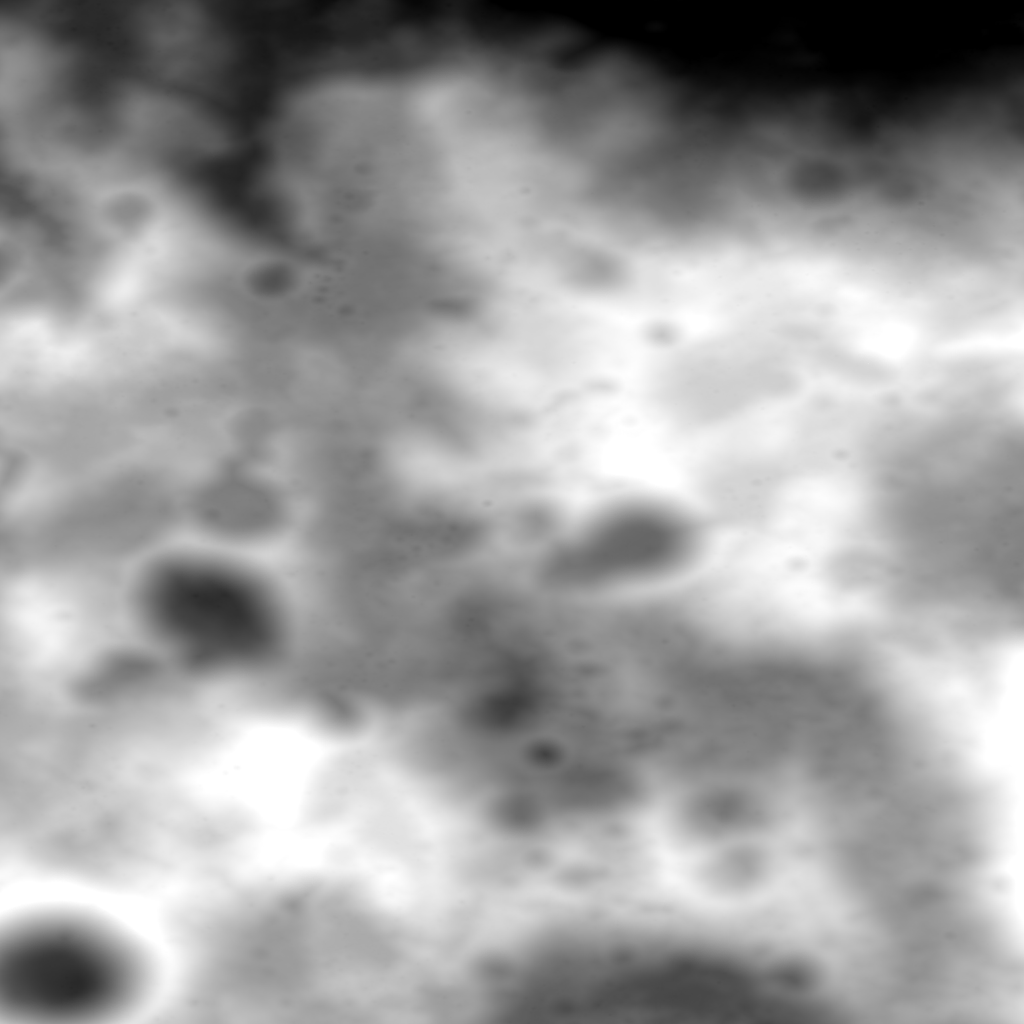

Supplement: Supplementary file 1 [file sensors-26-04344-s001.zip › data/images/test/tile_00567_lon81.0_lat45.0.png]

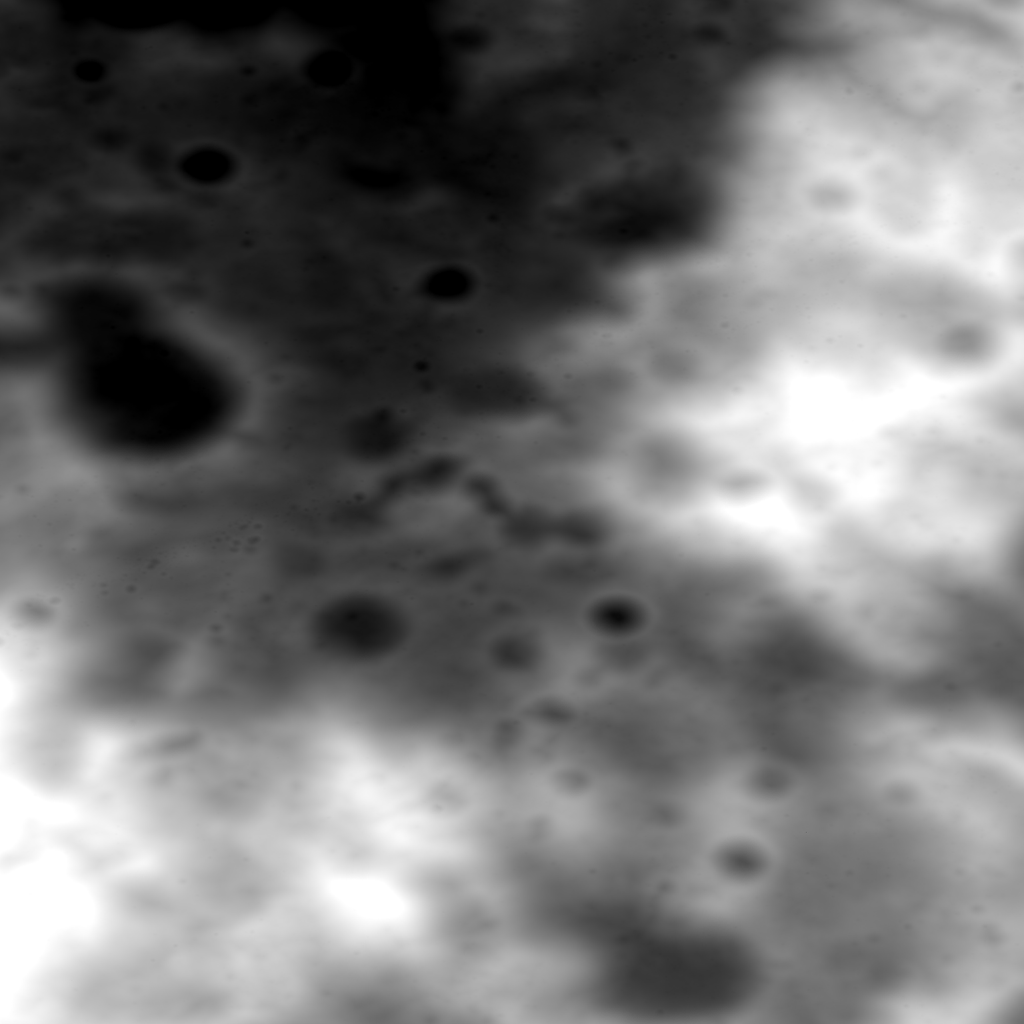

Supplement: Supplementary file 1 [file sensors-26-04344-s001.zip › data/images/test/tile_00570_lon90.0_lat45.0.png]

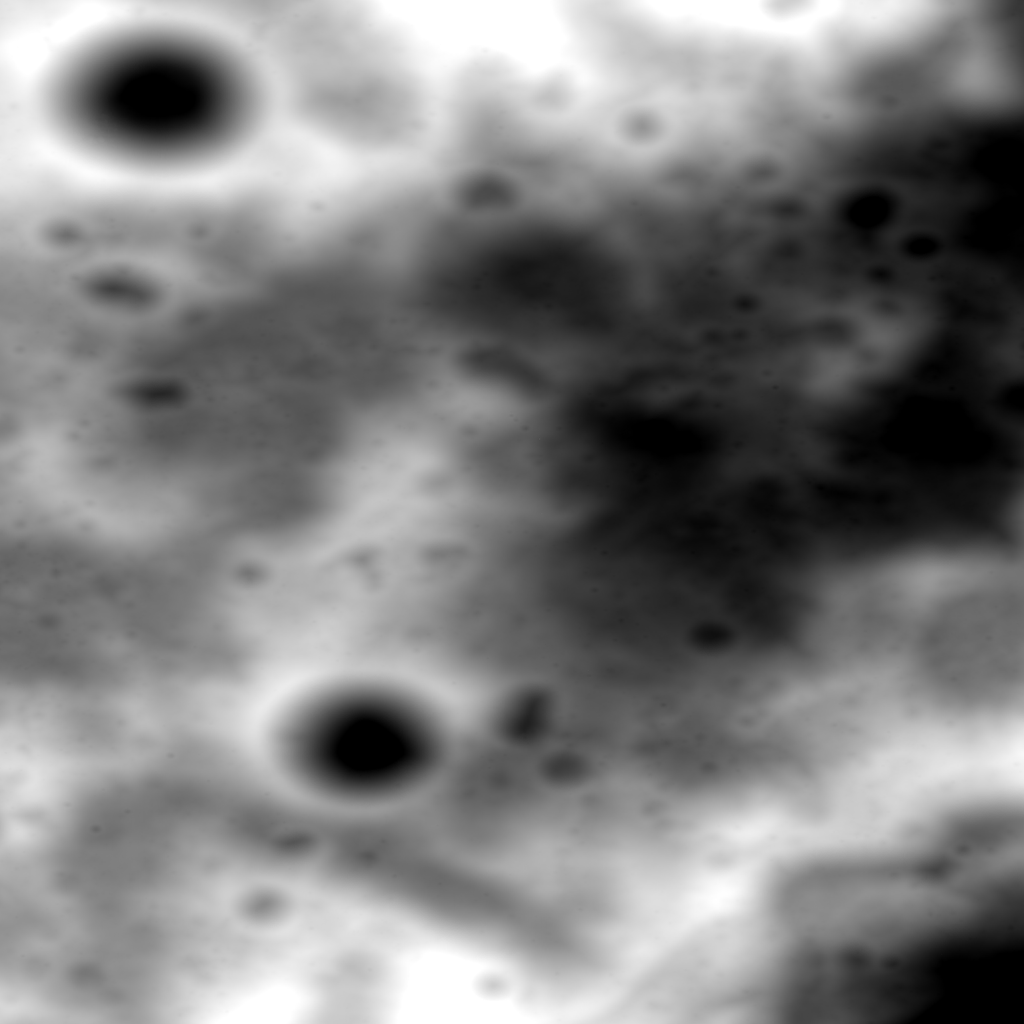

Supplement: Supplementary file 1 [file sensors-26-04344-s001.zip › data/images/test/tile_00572_lon96.0_lat45.0.png]

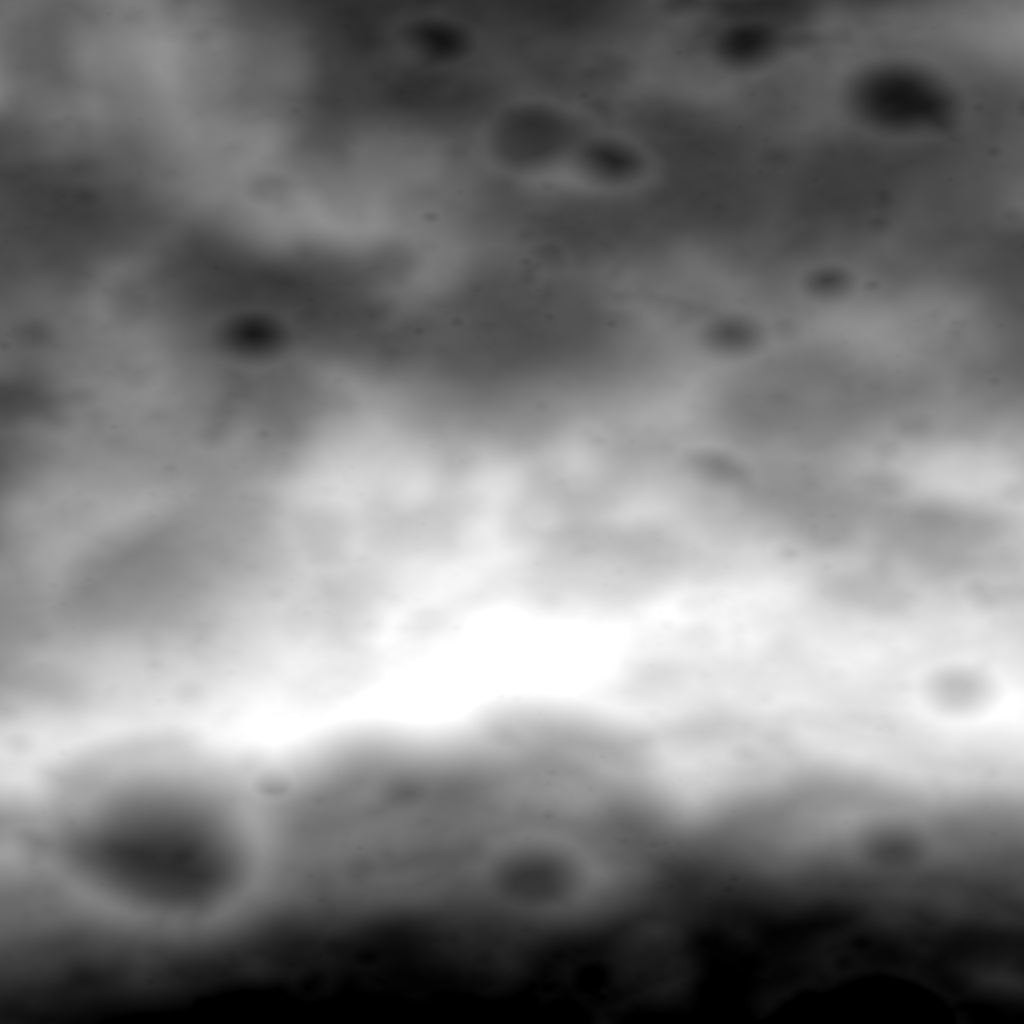

Supplement: Supplementary file 1 [file sensors-26-04344-s001.zip › data/images/test/tile_00573_lon99.0_lat45.0.png]

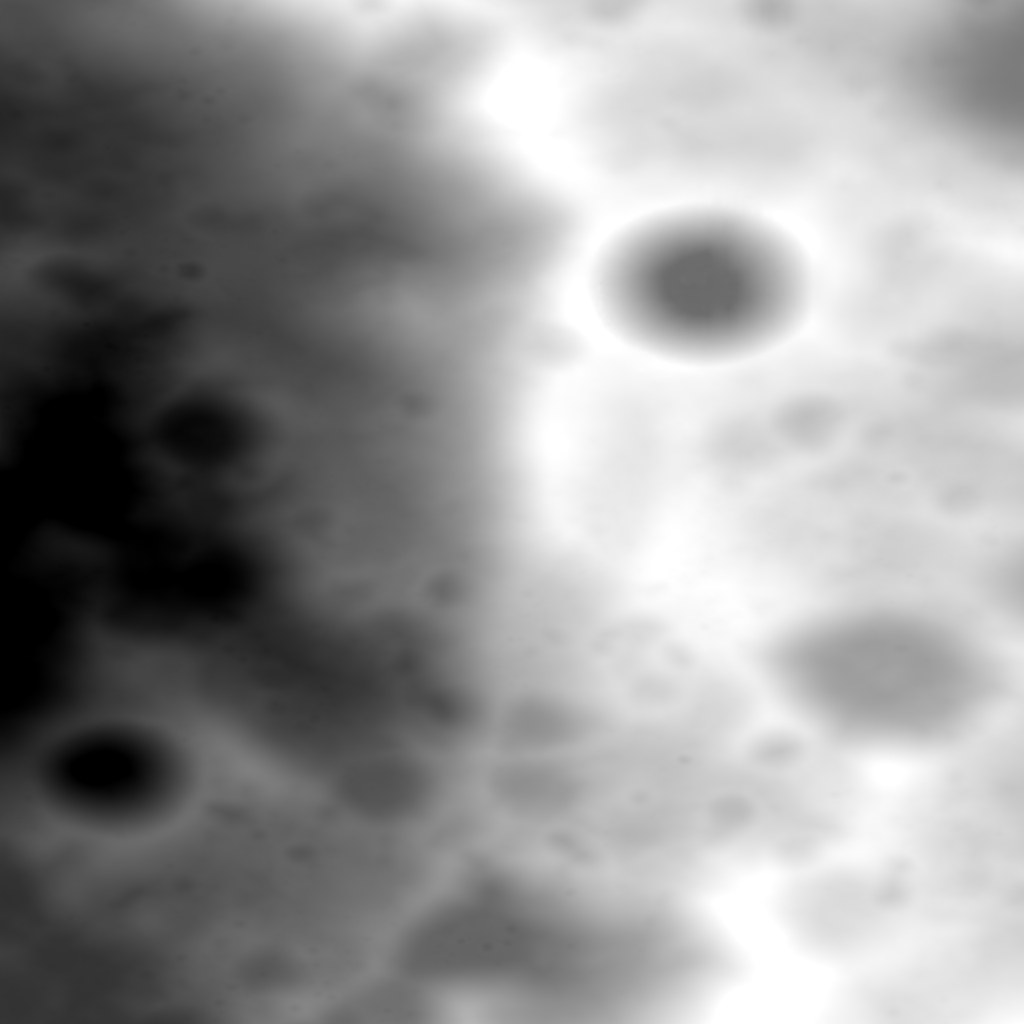

Supplement: Supplementary file 1 [file sensors-26-04344-s001.zip › data/images/test/tile_00577_lon111.0_lat45.0.png]

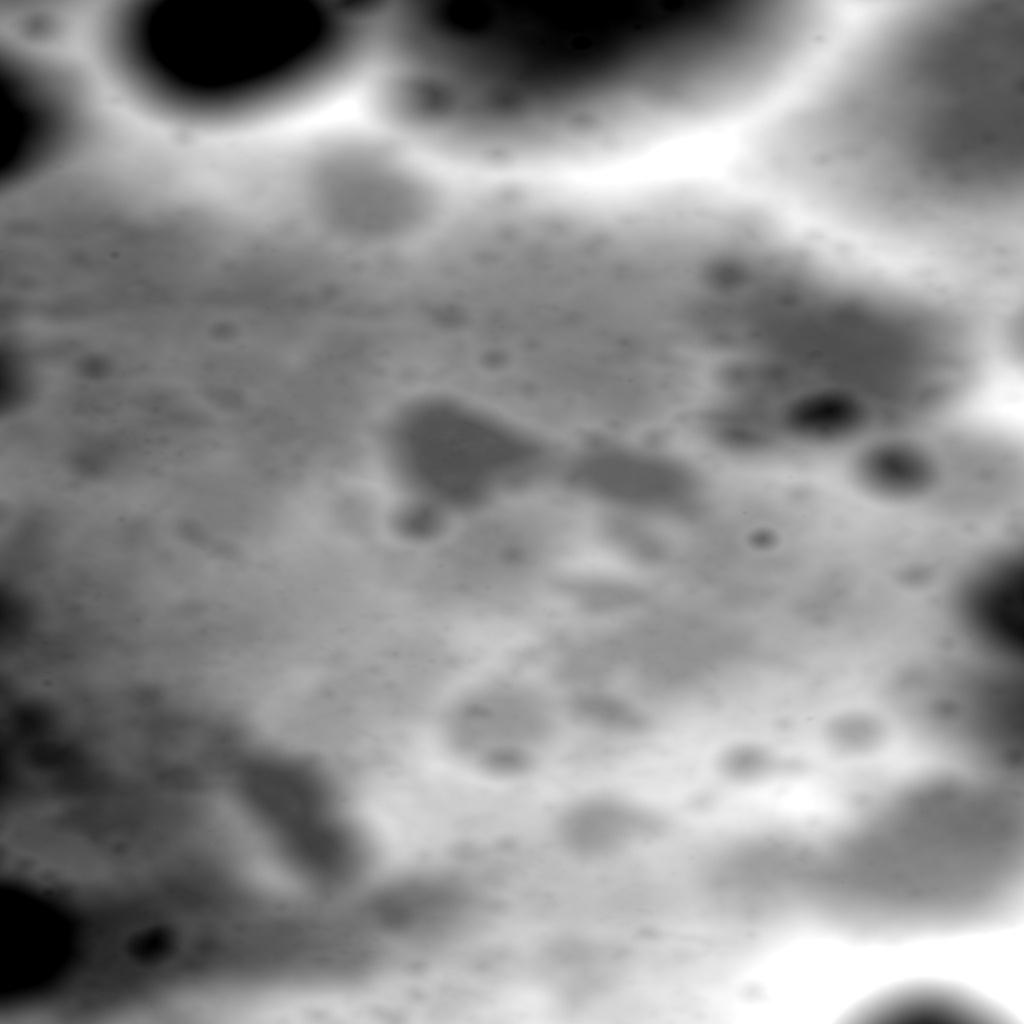

Supplement: Supplementary file 1 [file sensors-26-04344-s001.zip › data/images/test/tile_00584_lon132.0_lat45.0.png]

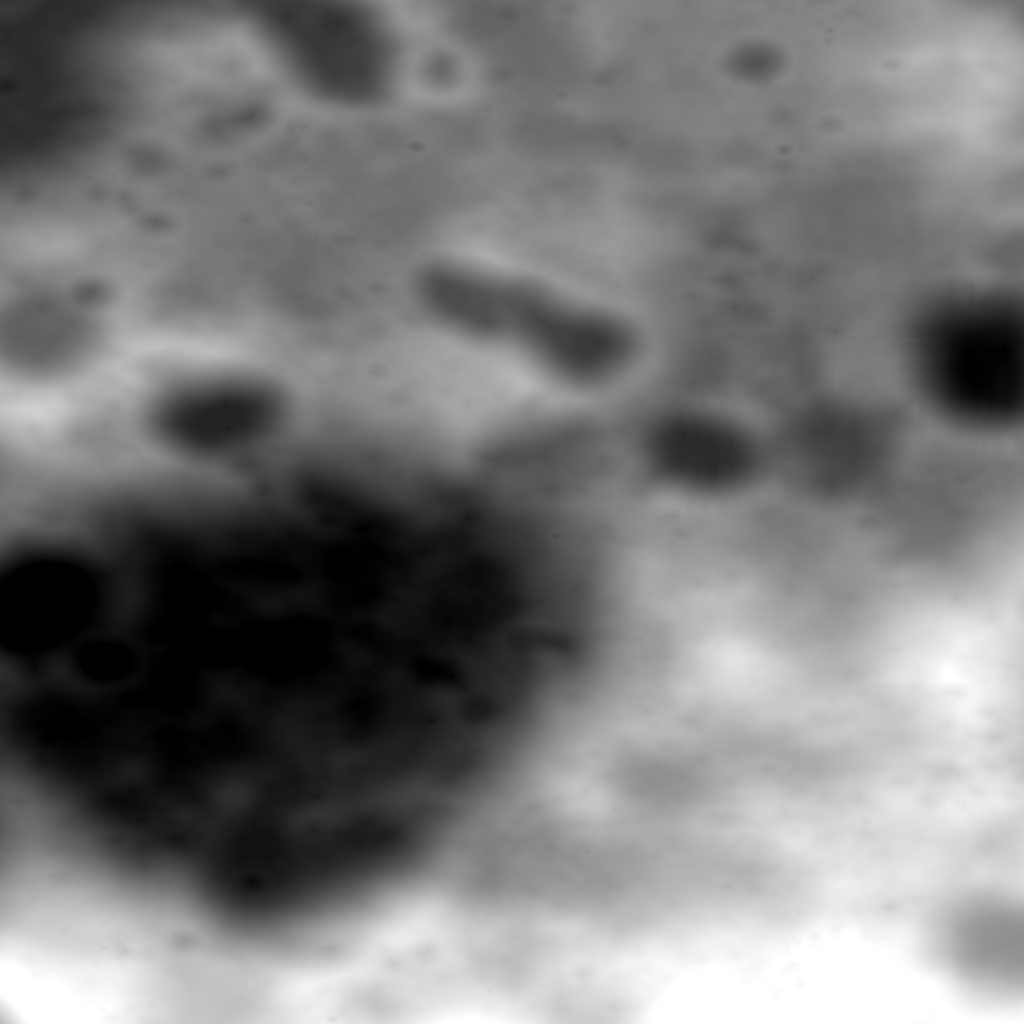

Supplement: Supplementary file 1 [file sensors-26-04344-s001.zip › data/images/test/tile_00585_lon135.0_lat45.0.png]

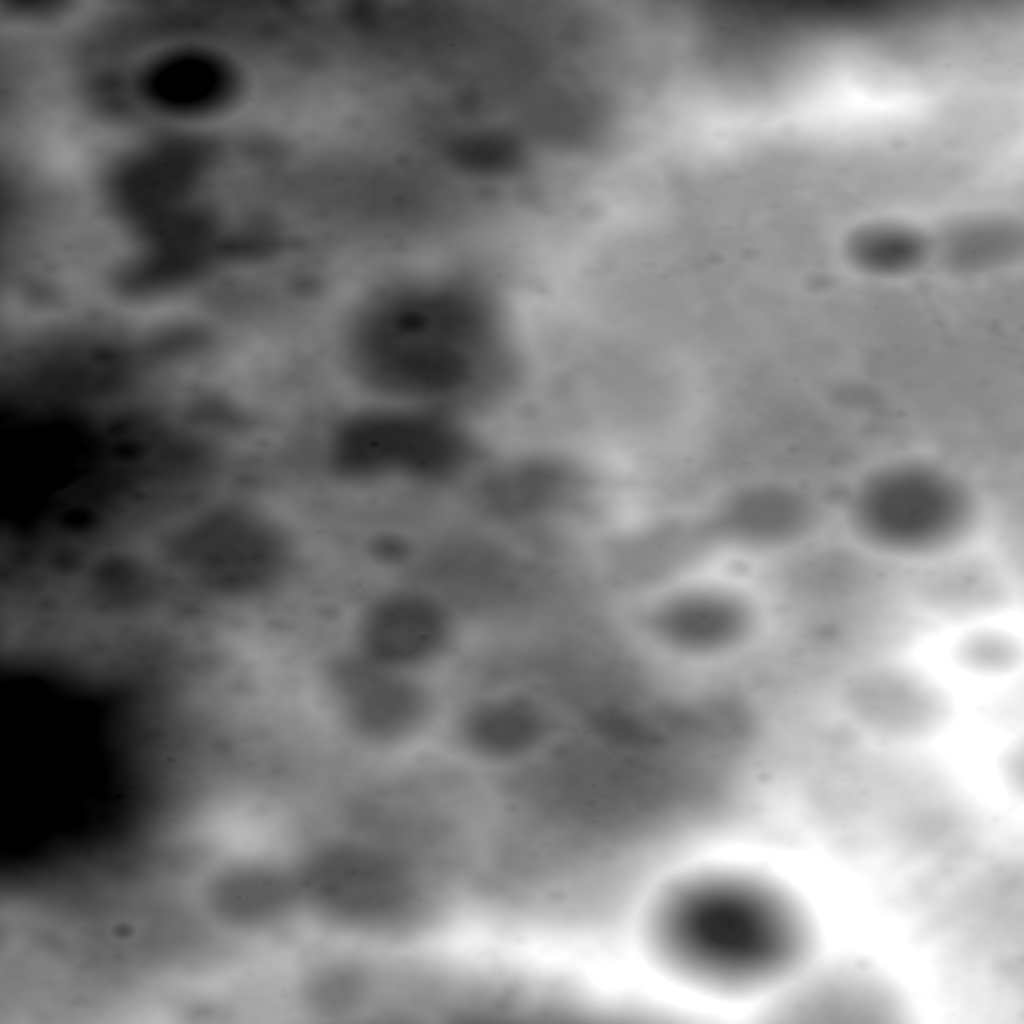

Supplement: Supplementary file 1 [file sensors-26-04344-s001.zip › data/images/test/tile_00588_lon144.0_lat45.0.png]

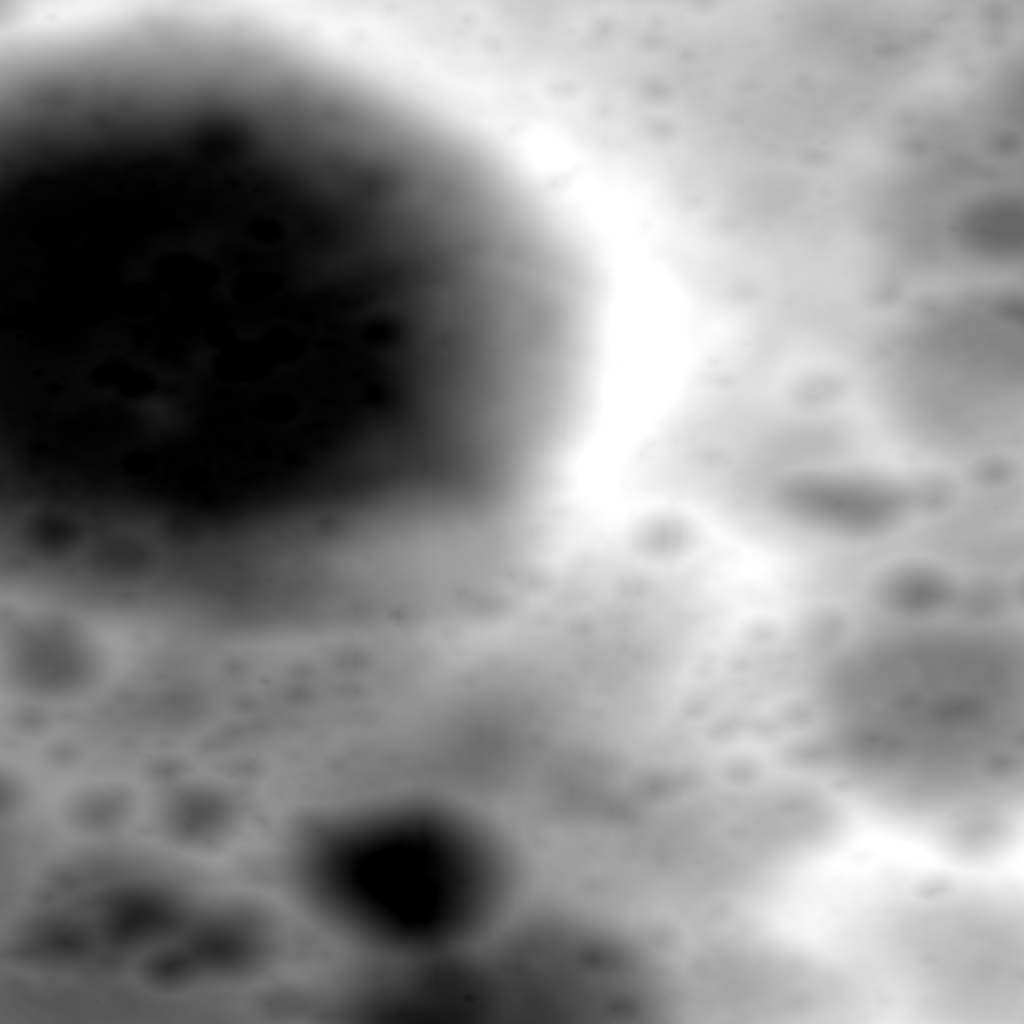

Supplement: Supplementary file 1 [file sensors-26-04344-s001.zip › data/images/test/tile_00598_lon174.0_lat45.0.png]

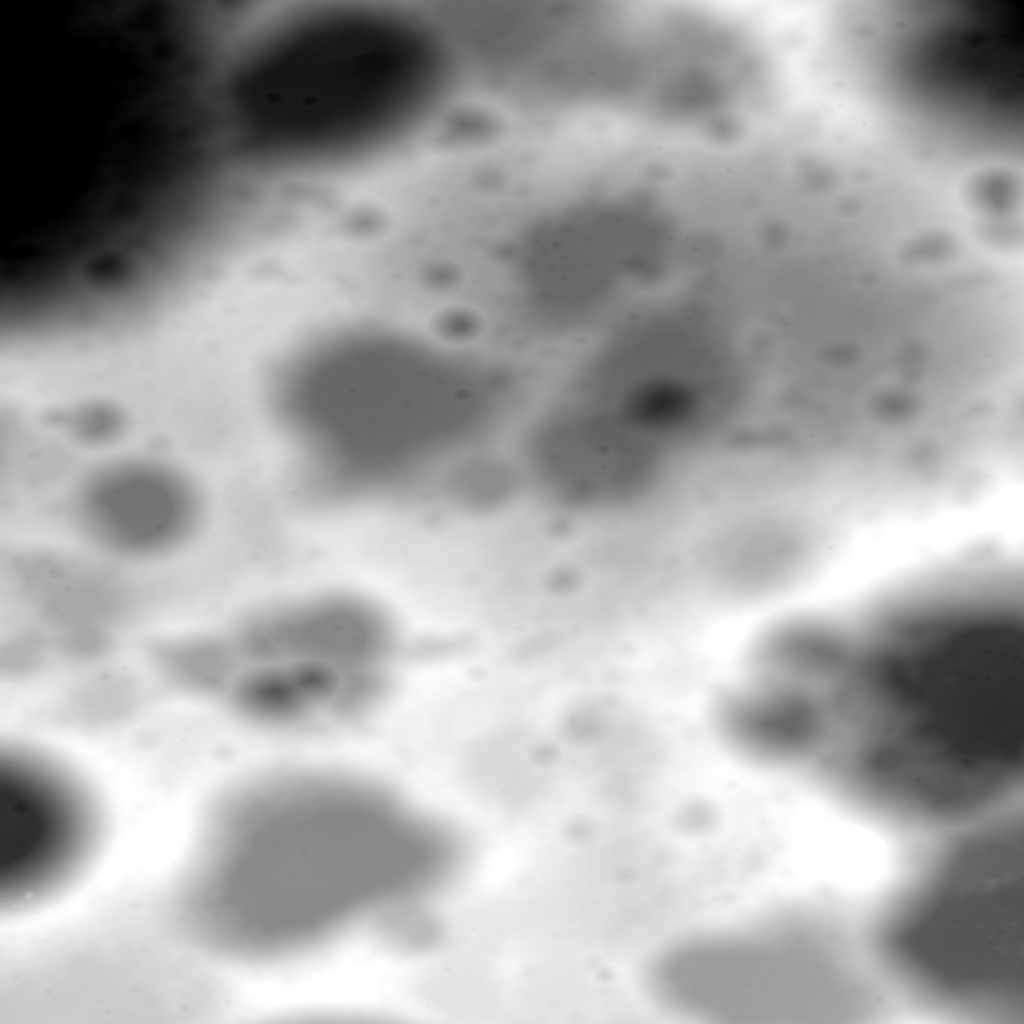

Supplement: Supplementary file 1 [file sensors-26-04344-s001.zip › data/images/test/tile_00601_lon-177.0_lat42.0.png]

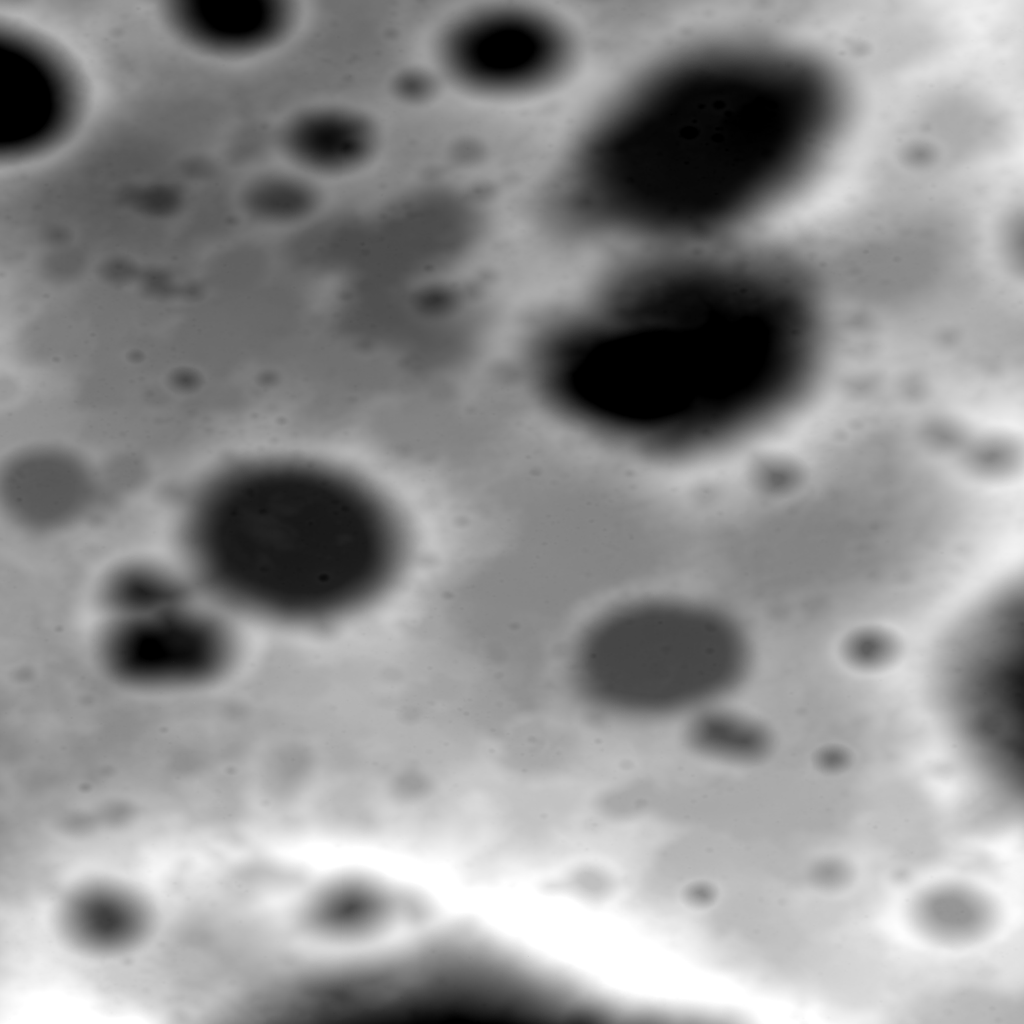

Supplement: Supplementary file 1 [file sensors-26-04344-s001.zip › data/images/test/tile_00605_lon-165.0_lat42.0.png]

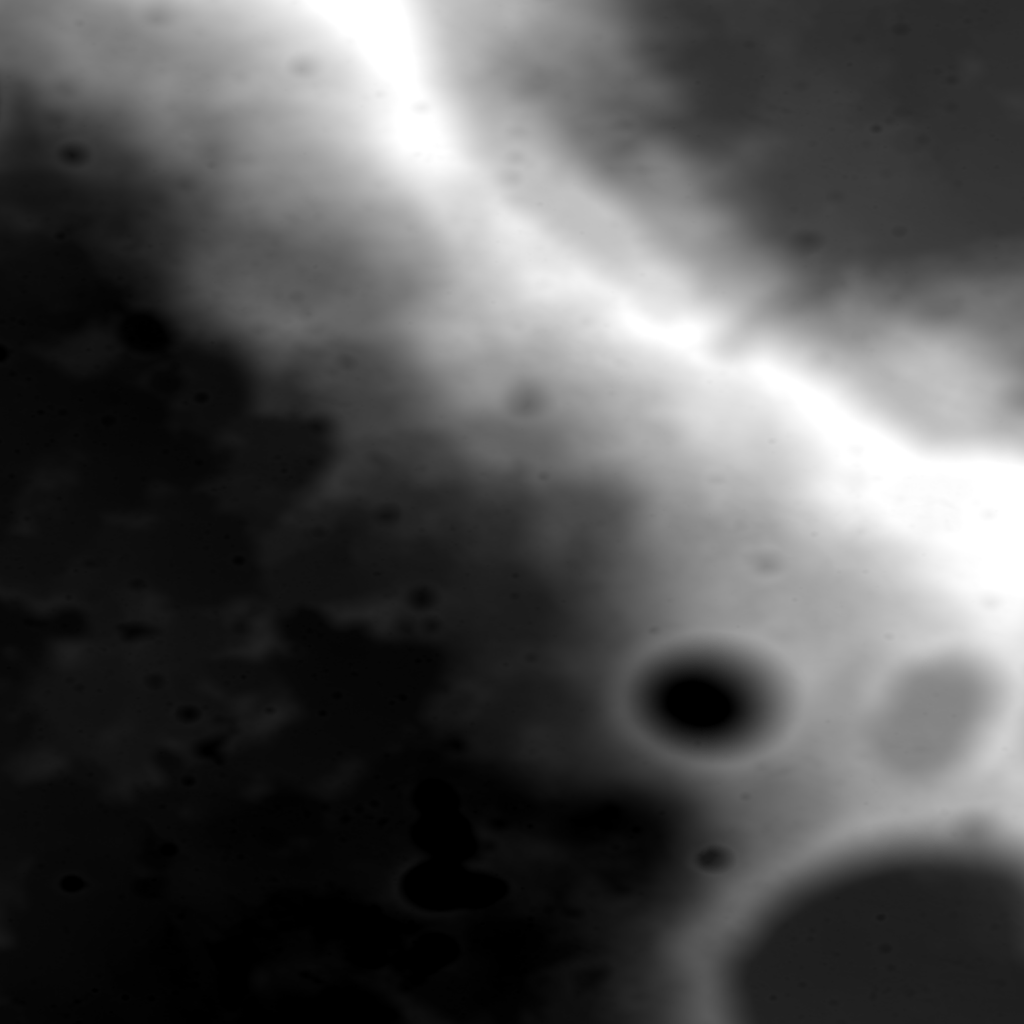

Supplement: Supplementary file 1 [file sensors-26-04344-s001.zip › data/images/test/tile_00621_lon-117.0_lat42.0.png]

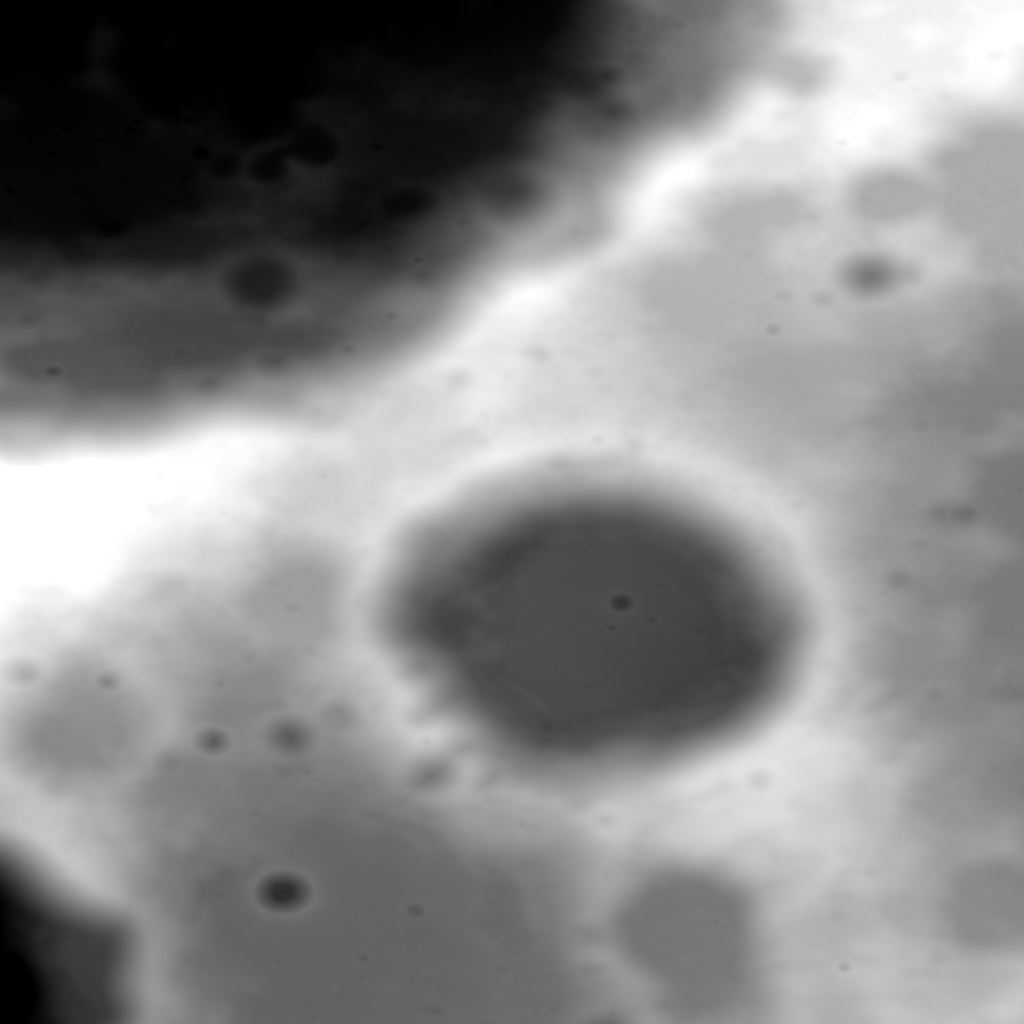

Supplement: Supplementary file 1 [file sensors-26-04344-s001.zip › data/images/test/tile_00622_lon-114.0_lat42.0.png]

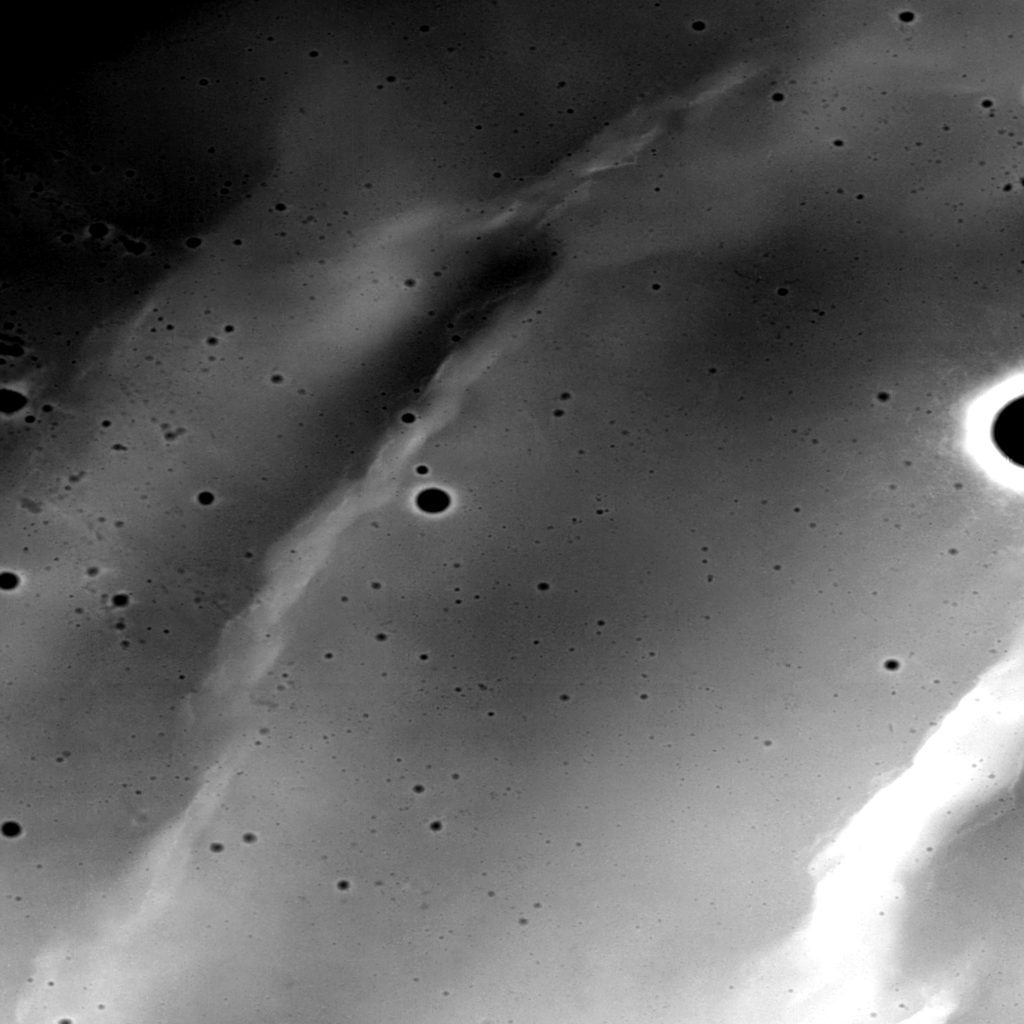

Supplement: Supplementary file 1 [file sensors-26-04344-s001.zip › data/images/test/tile_00650_lon-30.0_lat42.0.png]

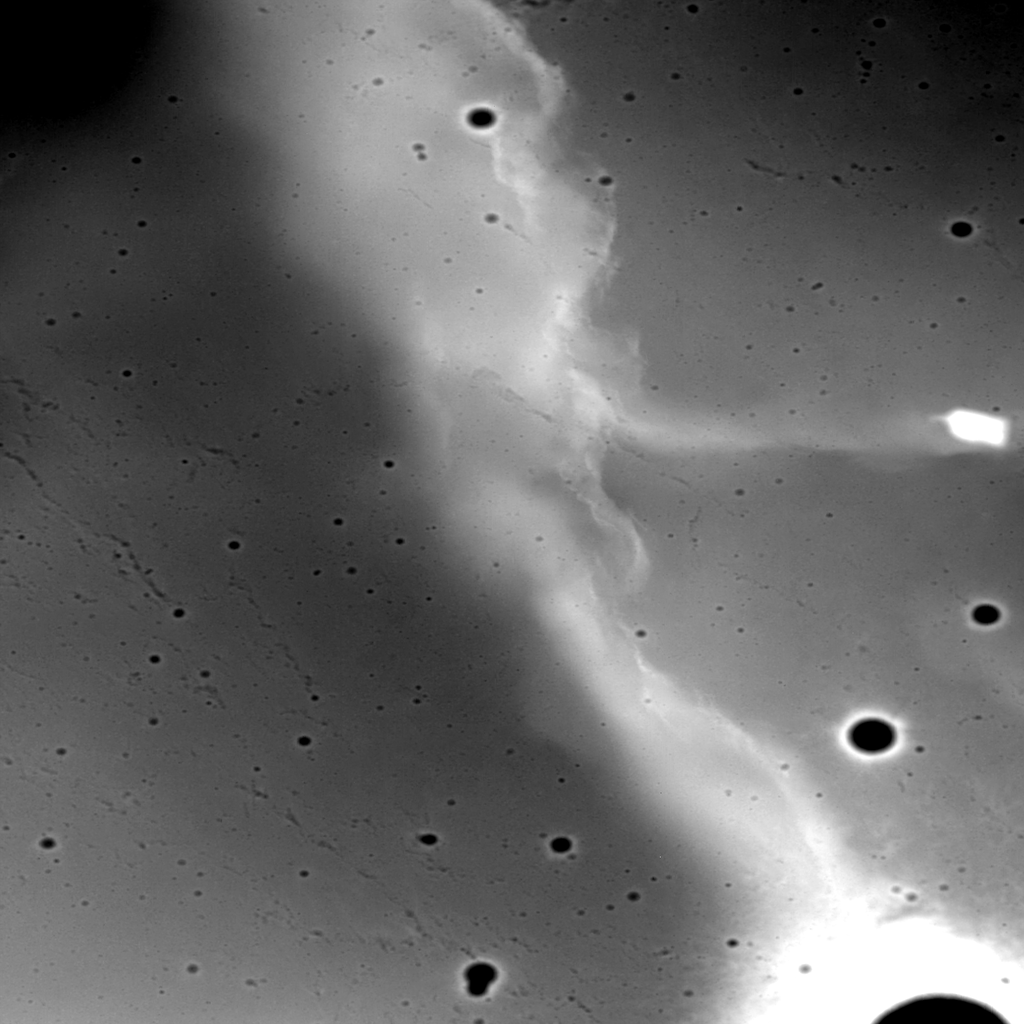

Supplement: Supplementary file 1 [file sensors-26-04344-s001.zip › data/images/test/tile_00658_lon-6.0_lat42.0.png]

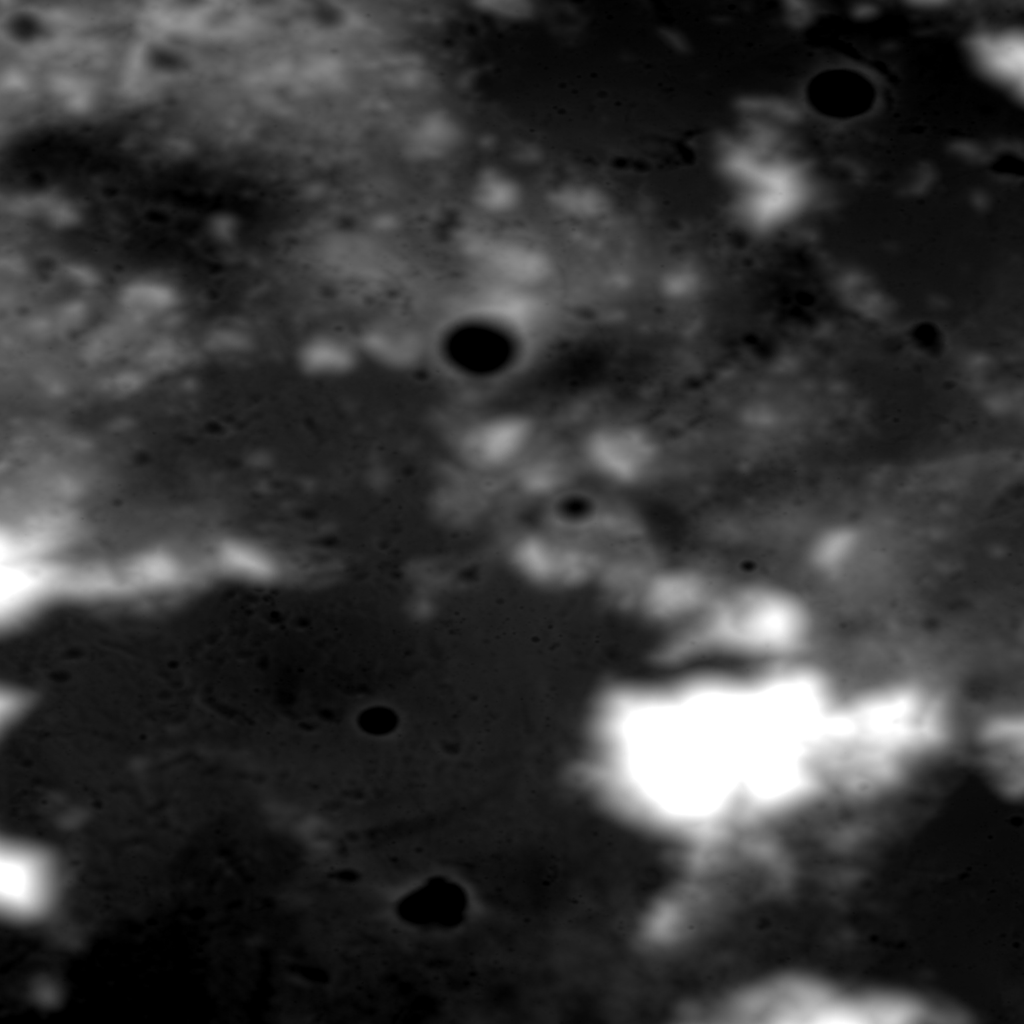

Supplement: Supplementary file 1 [file sensors-26-04344-s001.zip › data/images/test/tile_00661_lon3.0_lat42.0.png]

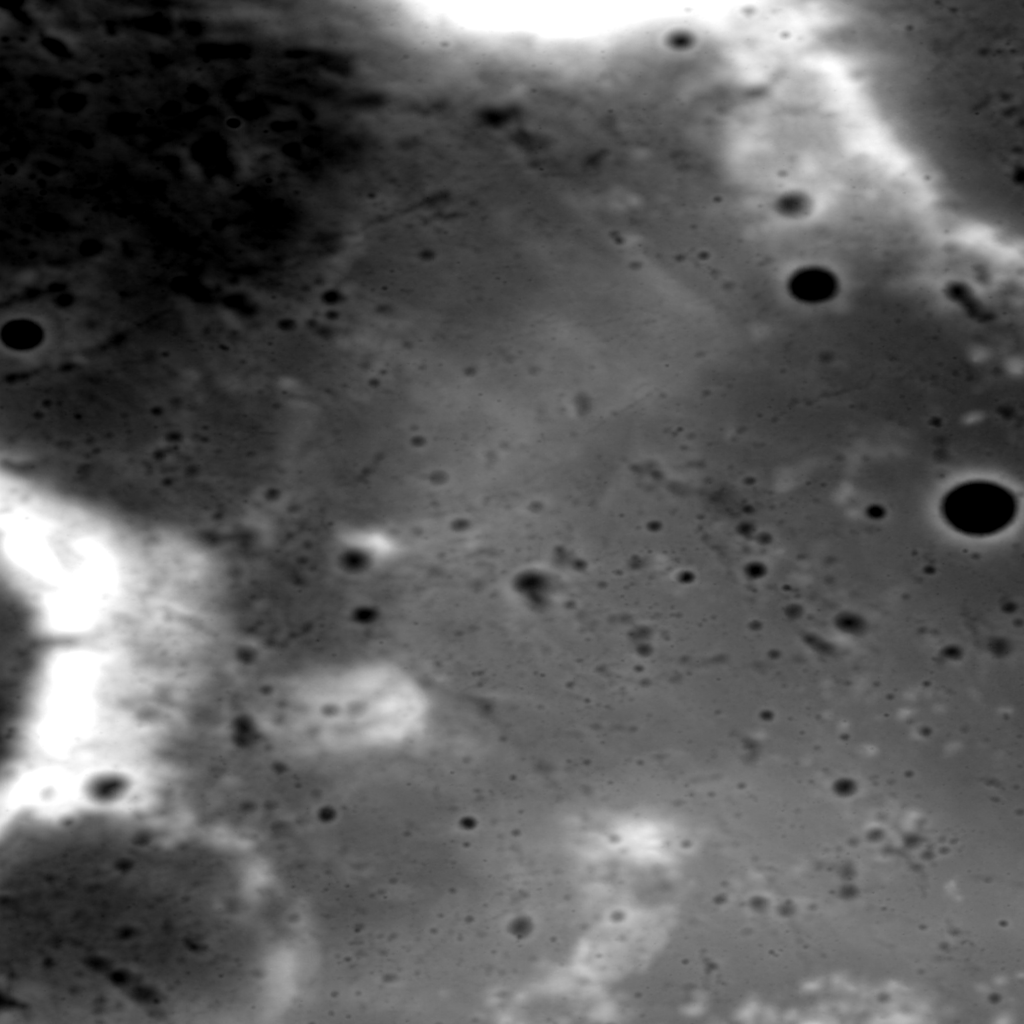

Supplement: Supplementary file 1 [file sensors-26-04344-s001.zip › data/images/test/tile_00676_lon48.0_lat42.0.png]

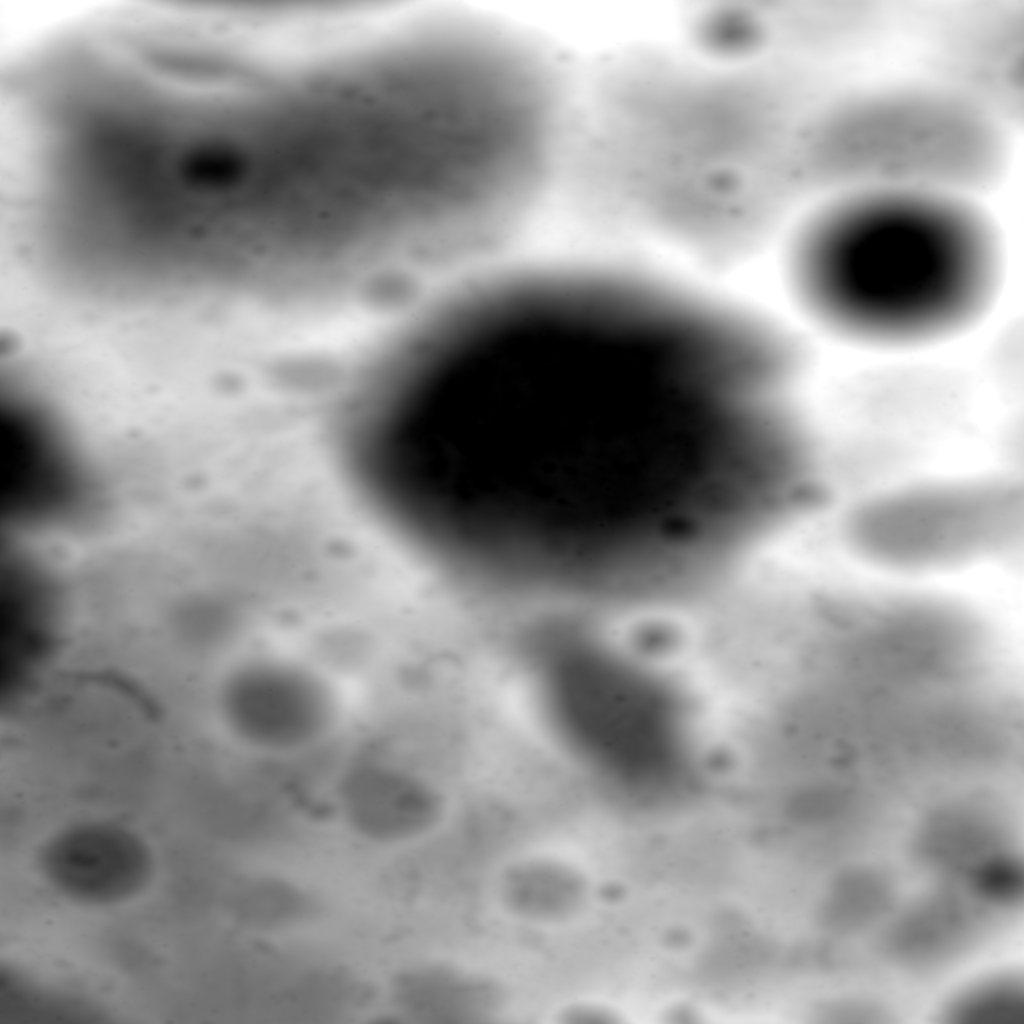

Supplement: Supplementary file 1 [file sensors-26-04344-s001.zip › data/images/test/tile_00699_lon117.0_lat42.0.png]

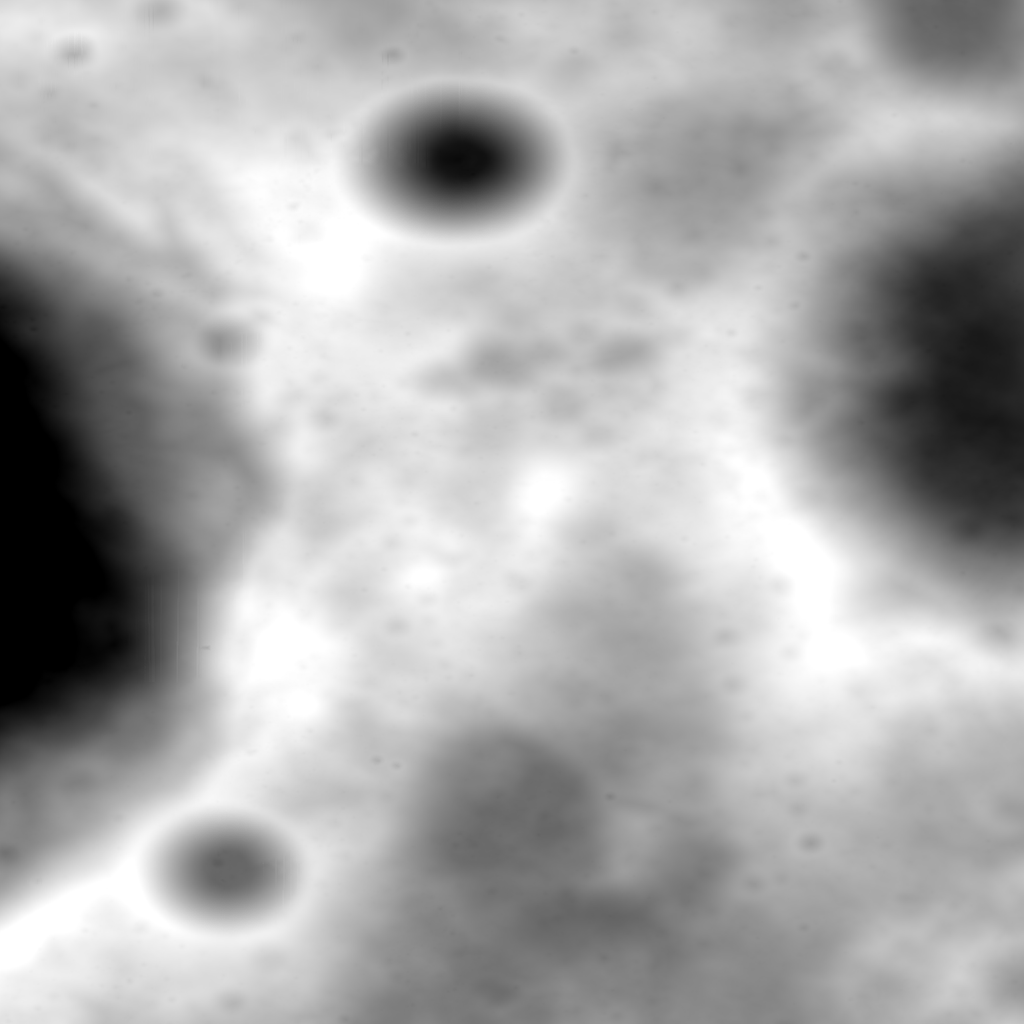

Supplement: Supplementary file 1 [file sensors-26-04344-s001.zip › data/images/test/tile_00706_lon138.0_lat42.0.png]

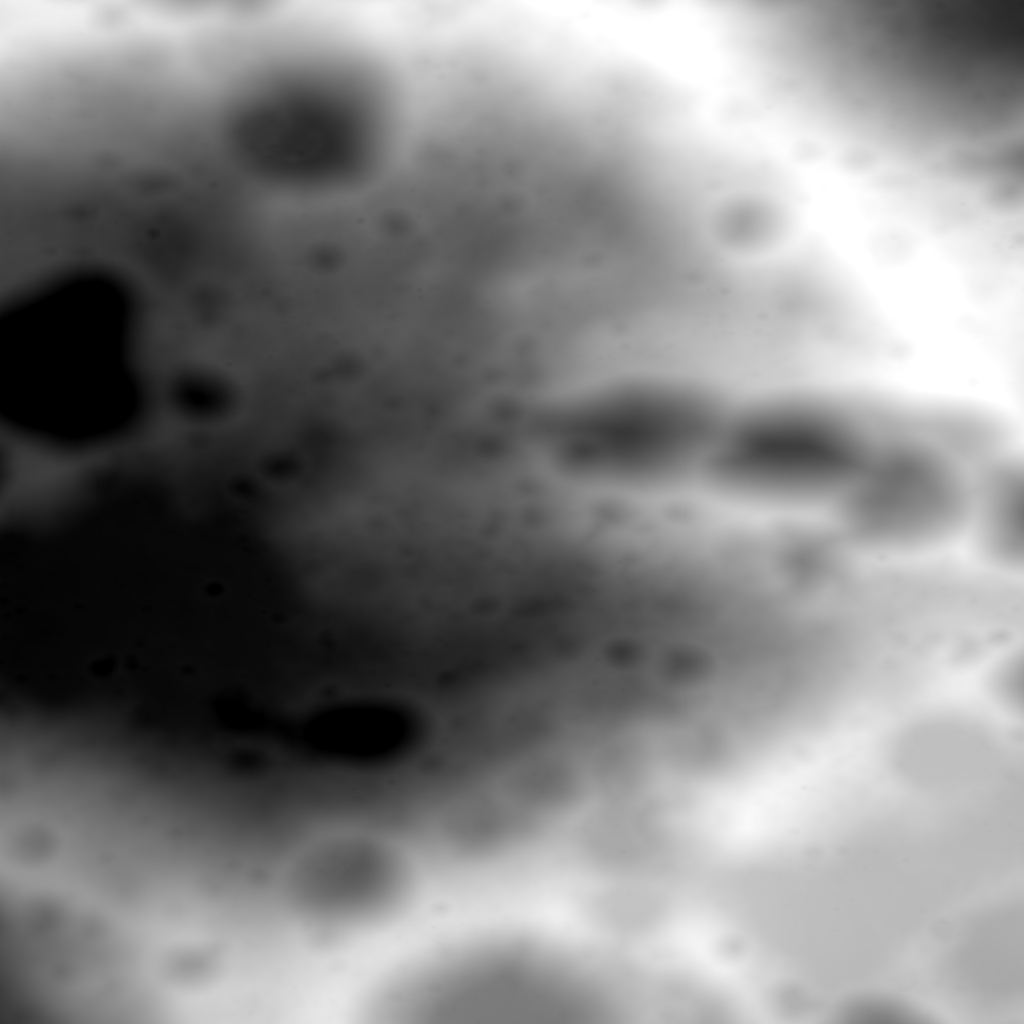

Supplement: Supplementary file 1 [file sensors-26-04344-s001.zip › data/images/test/tile_00717_lon171.0_lat42.0.png]

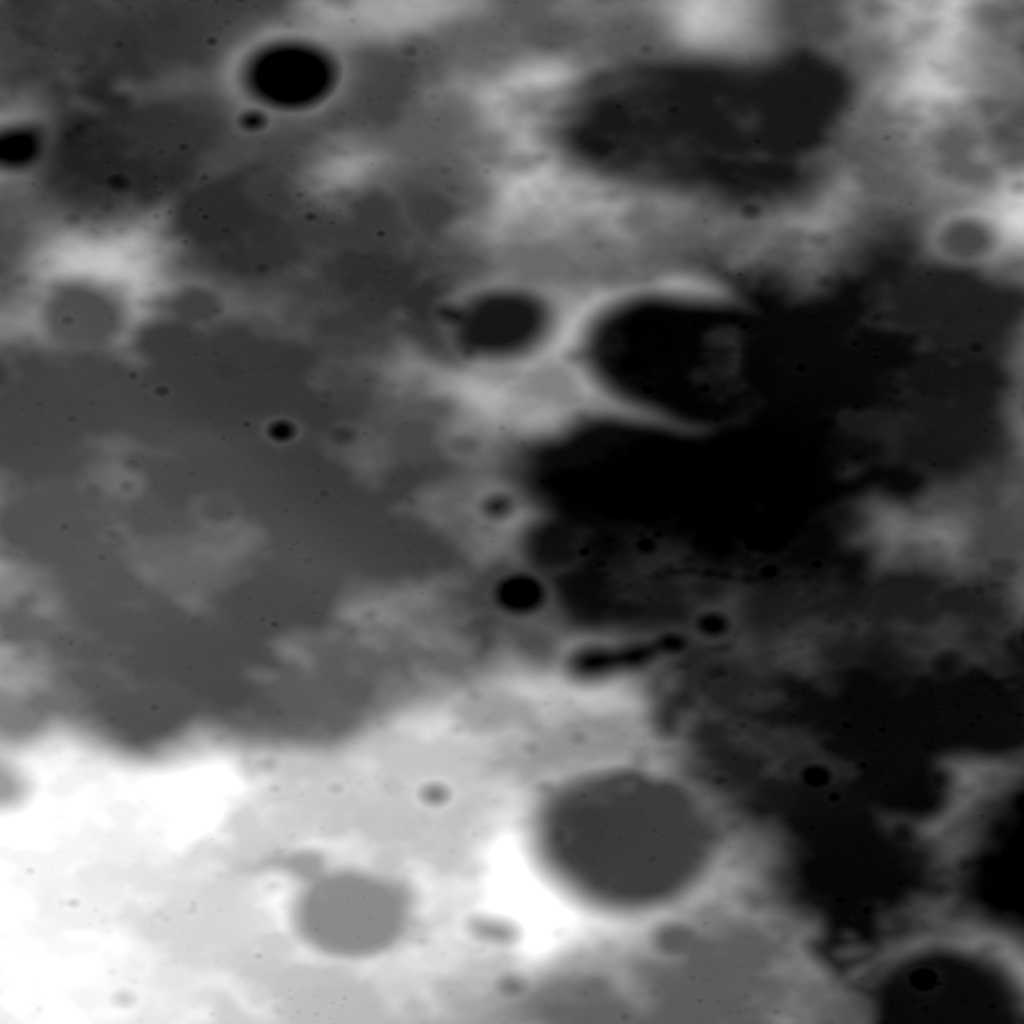

Supplement: Supplementary file 1 [file sensors-26-04344-s001.zip › data/images/test/tile_00750_lon-90.0_lat39.0.png]

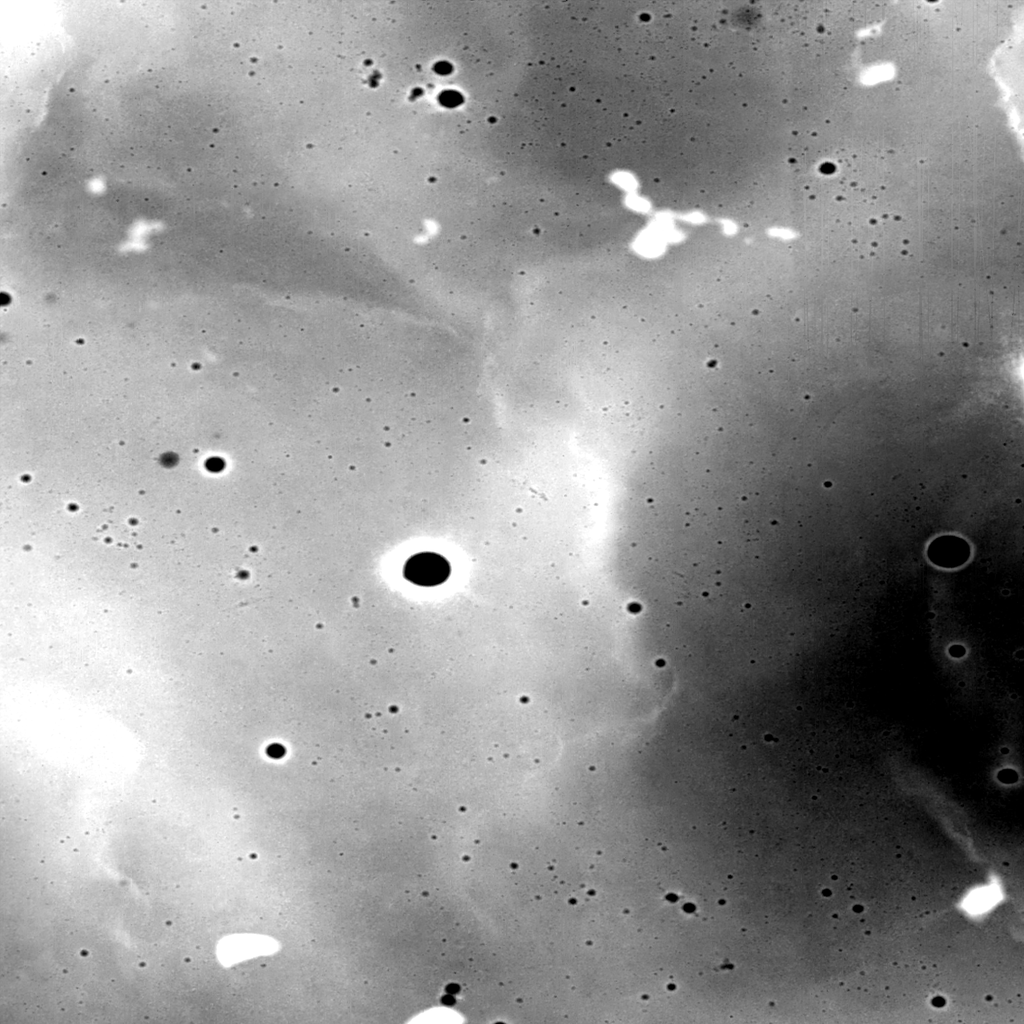

Supplement: Supplementary file 1 [file sensors-26-04344-s001.zip › data/images/test/tile_00762_lon-54.0_lat39.0.png]

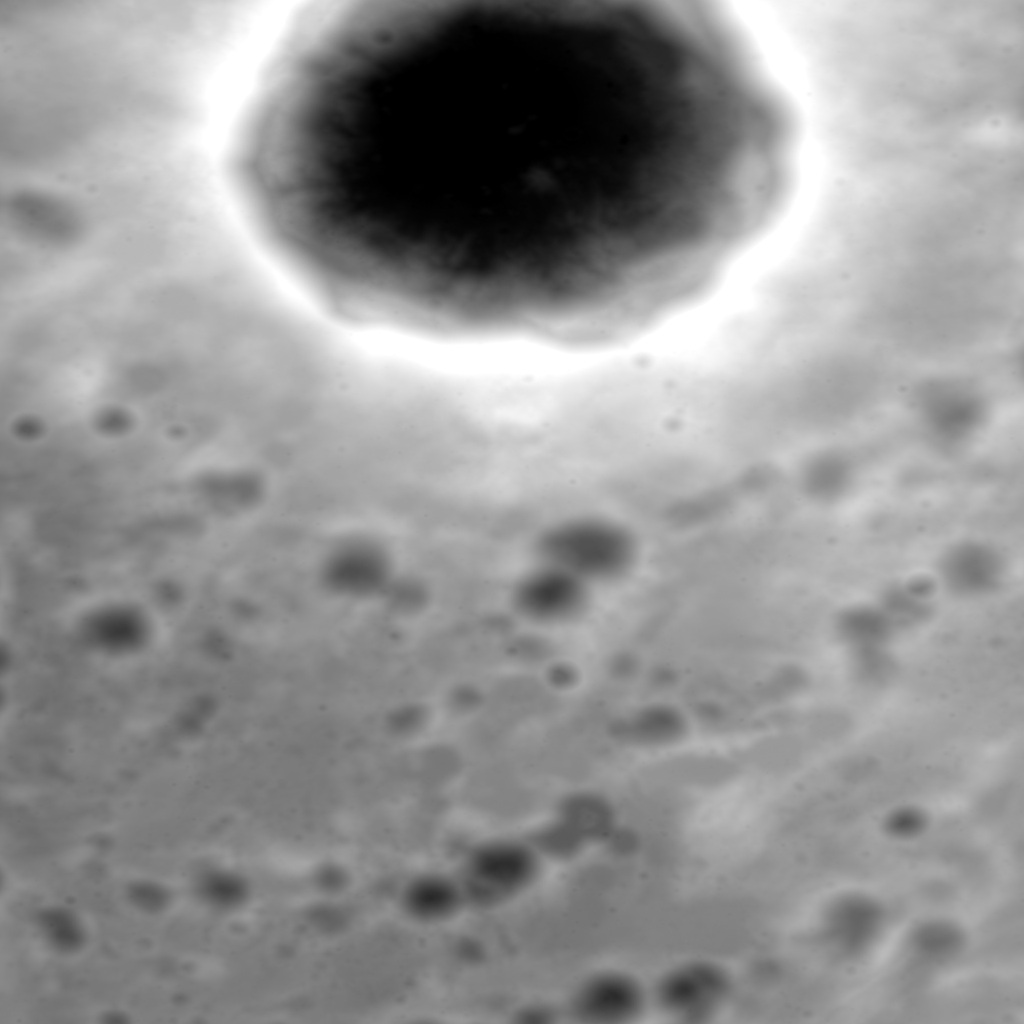

Supplement: Supplementary file 1 [file sensors-26-04344-s001.zip › data/images/test/tile_00765_lon-45.0_lat39.0.png]

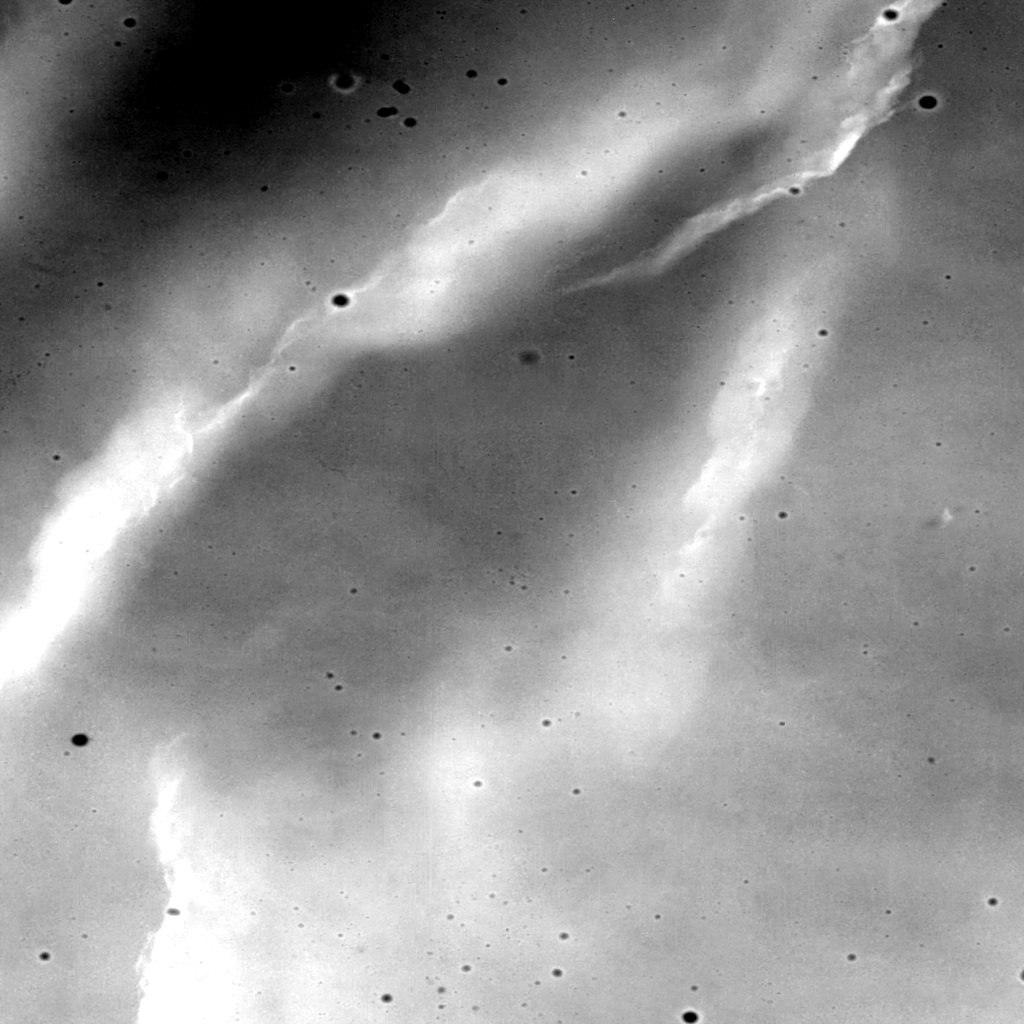

Supplement: Supplementary file 1 [file sensors-26-04344-s001.zip › data/images/test/tile_00770_lon-30.0_lat39.0.png]

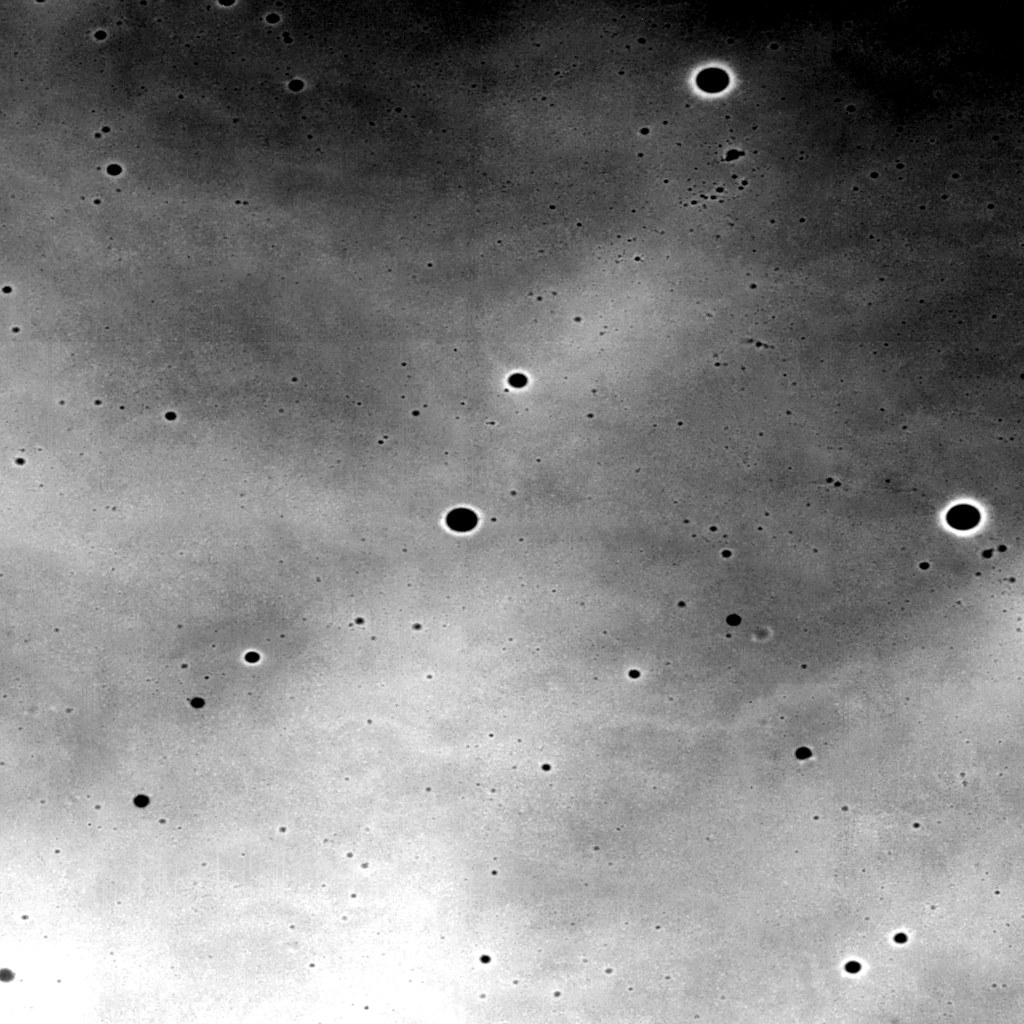

Supplement: Supplementary file 1 [file sensors-26-04344-s001.zip › data/images/test/tile_00771_lon-27.0_lat39.0.png]

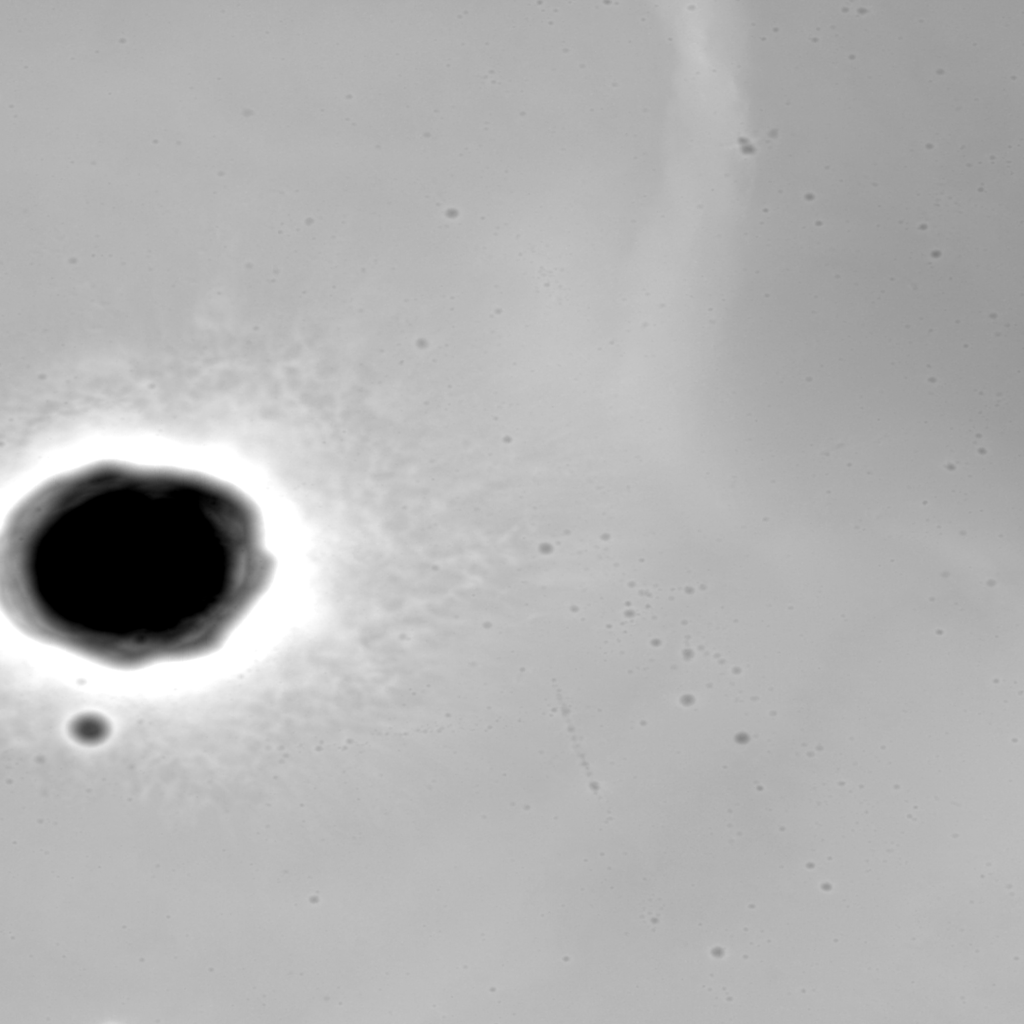

Supplement: Supplementary file 1 [file sensors-26-04344-s001.zip › data/images/test/tile_00773_lon-21.0_lat39.0.png]

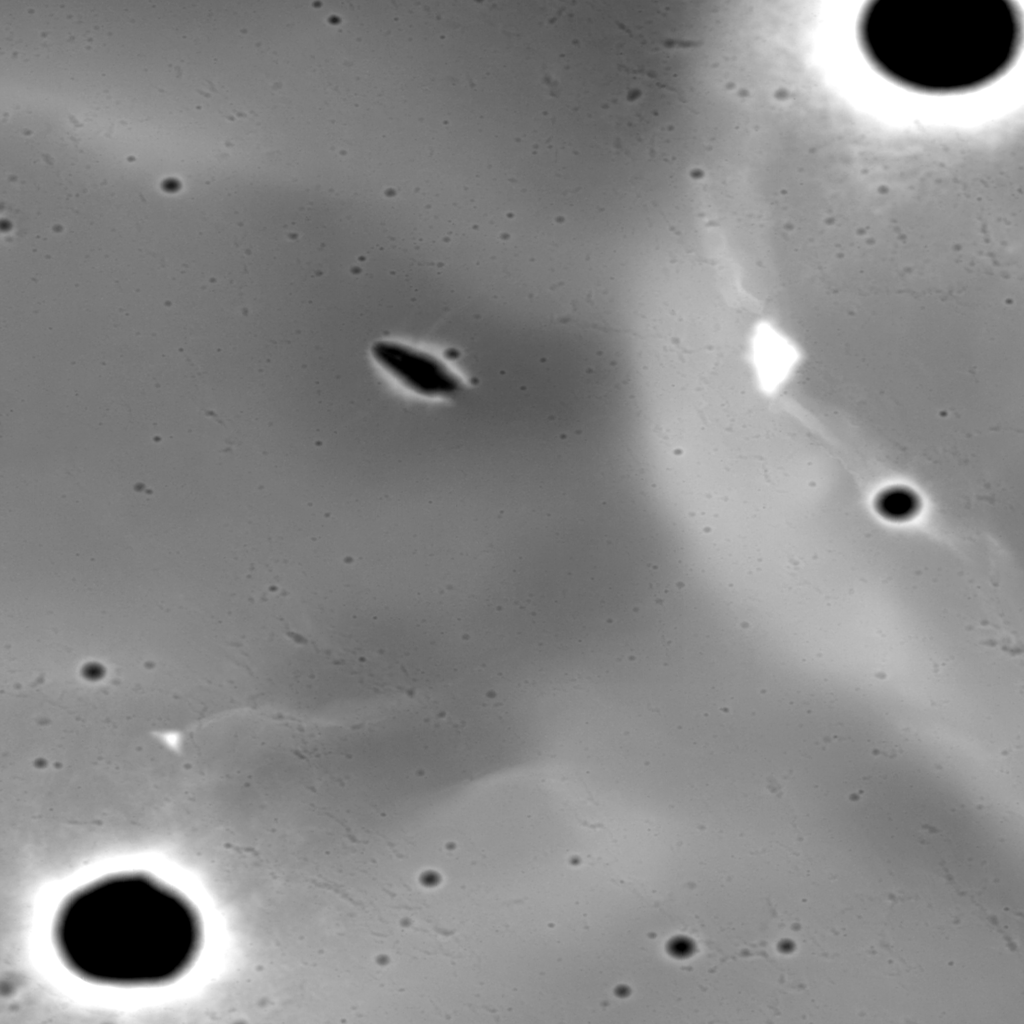

Supplement: Supplementary file 1 [file sensors-26-04344-s001.zip › data/images/test/tile_00778_lon-6.0_lat39.0.png]

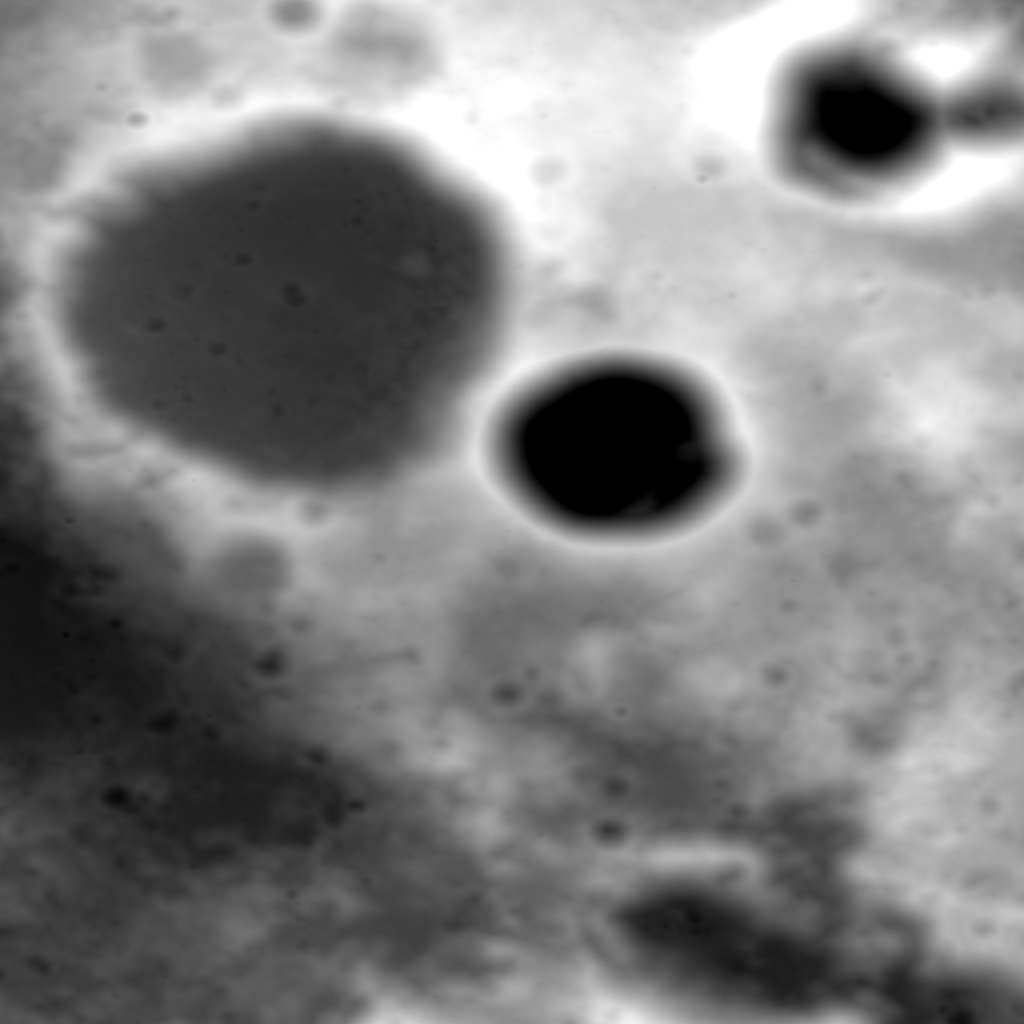

Supplement: Supplementary file 1 [file sensors-26-04344-s001.zip › data/images/test/tile_00798_lon54.0_lat39.0.png]

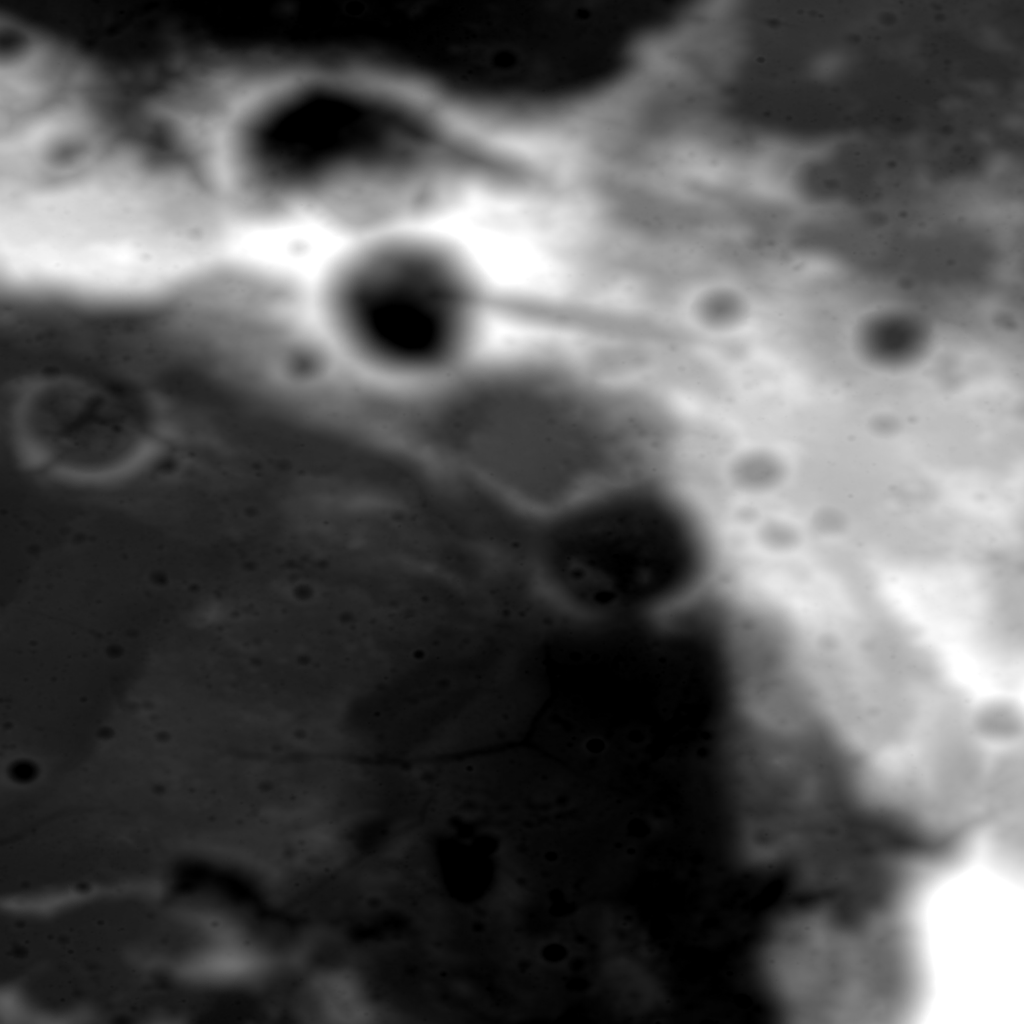

Supplement: Supplementary file 1 [file sensors-26-04344-s001.zip › data/images/test/tile_00800_lon60.0_lat39.0.png]

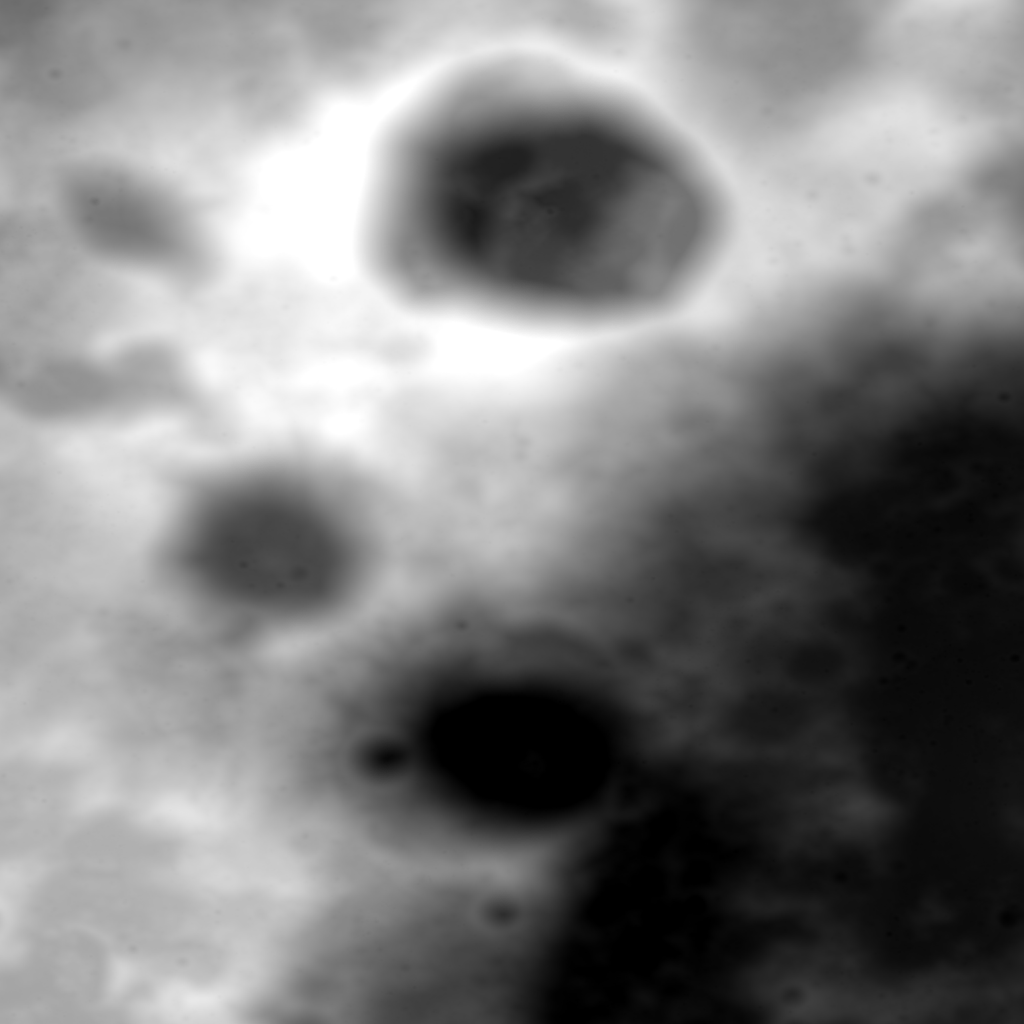

Supplement: Supplementary file 1 [file sensors-26-04344-s001.zip › data/images/test/tile_00808_lon84.0_lat39.0.png]

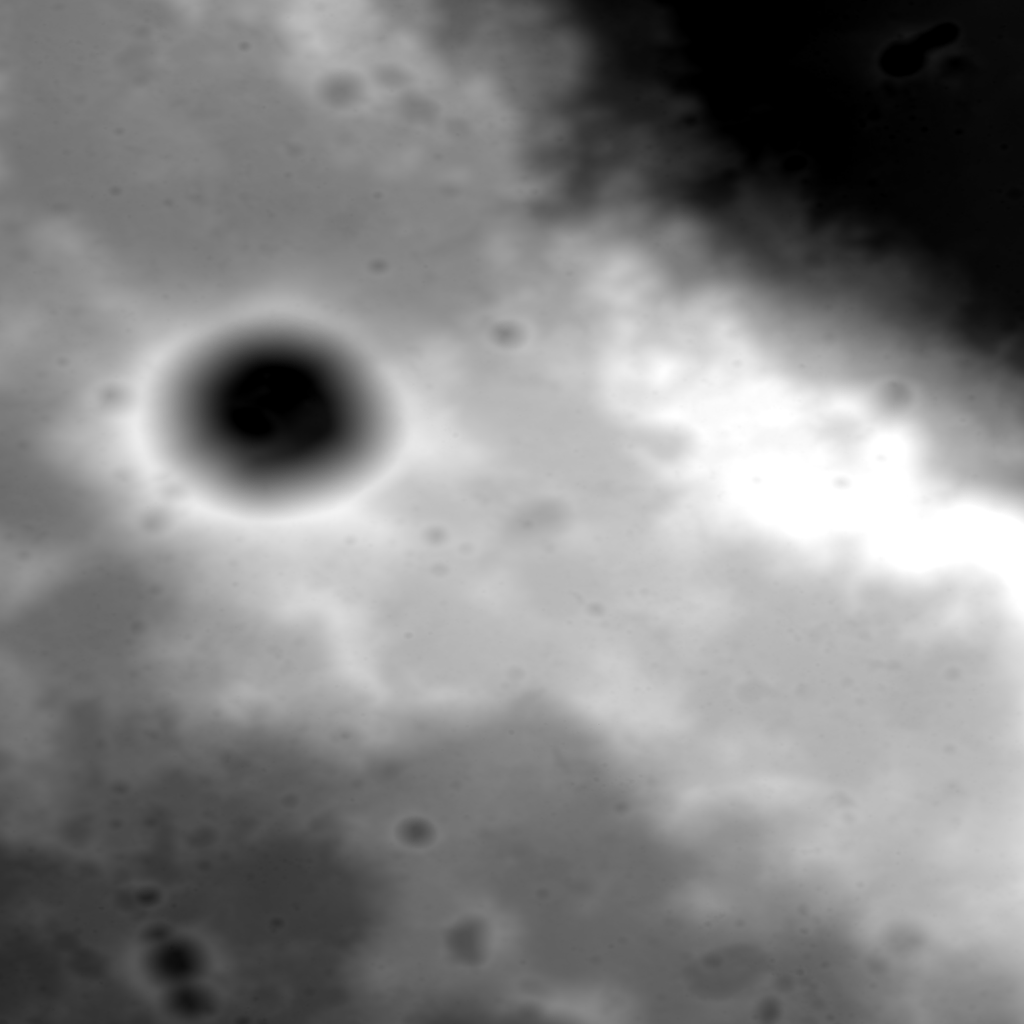

Supplement: Supplementary file 1 [file sensors-26-04344-s001.zip › data/images/test/tile_00812_lon96.0_lat39.0.png]

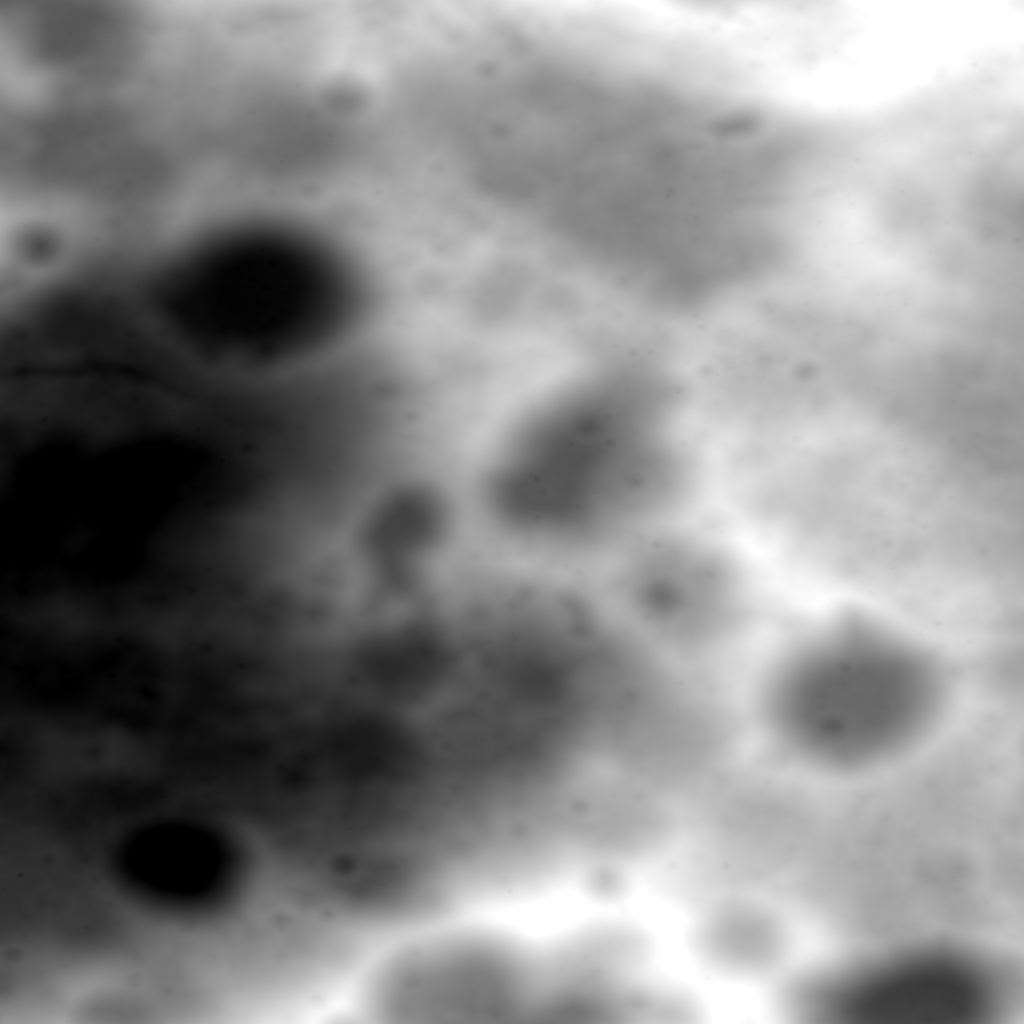

Supplement: Supplementary file 1 [file sensors-26-04344-s001.zip › data/images/test/tile_00825_lon135.0_lat39.0.png]

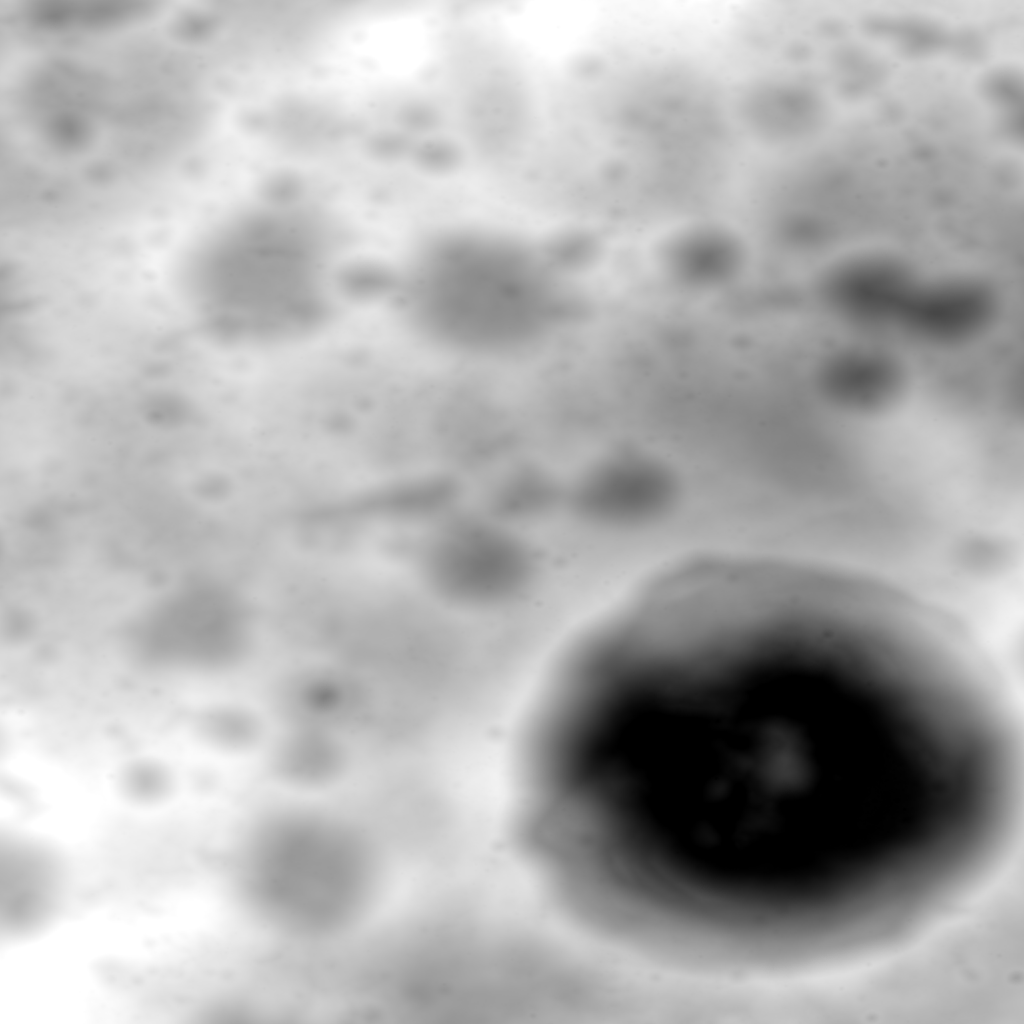

Supplement: Supplementary file 1 [file sensors-26-04344-s001.zip › data/images/test/tile_00833_lon159.0_lat39.0.png]

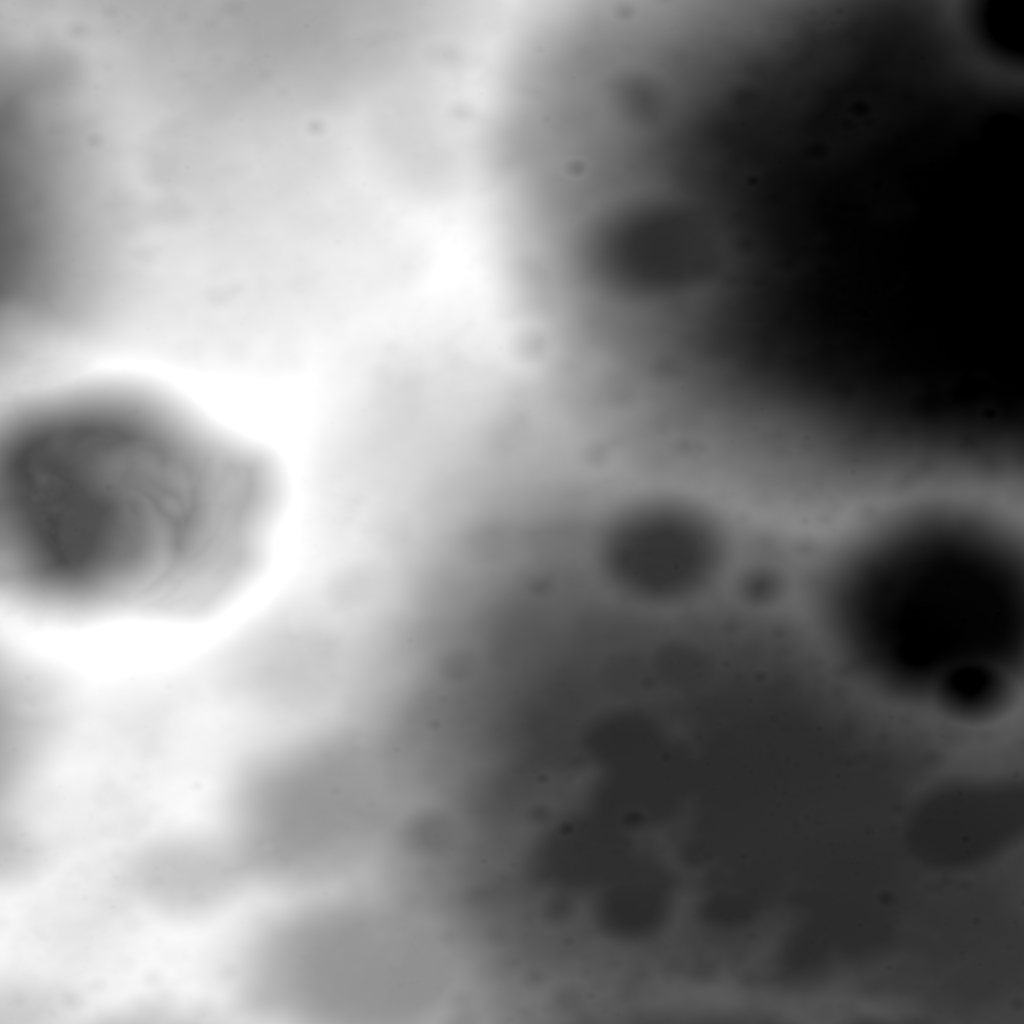

Supplement: Supplementary file 1 [file sensors-26-04344-s001.zip › data/images/test/tile_00844_lon-168.0_lat36.0.png]

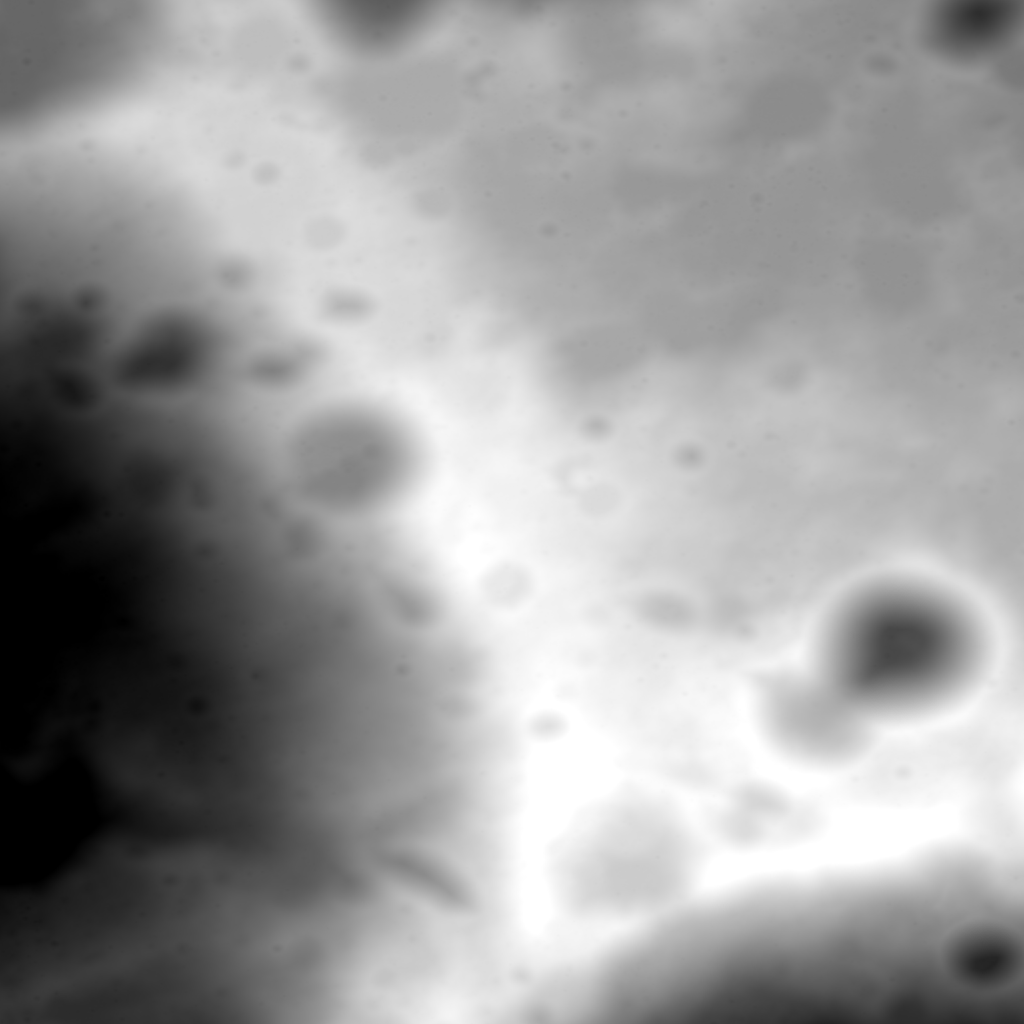

Supplement: Supplementary file 1 [file sensors-26-04344-s001.zip › data/images/test/tile_00846_lon-162.0_lat36.0.png]

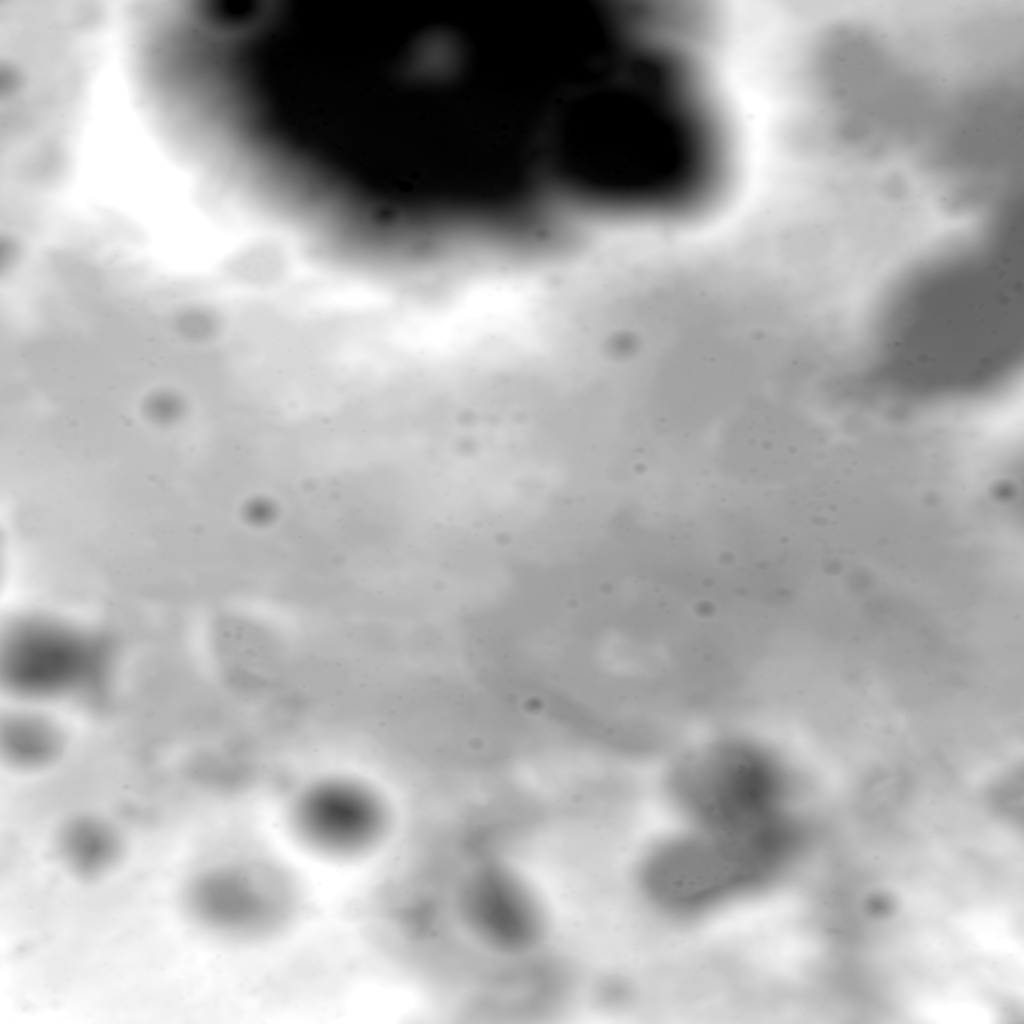

Supplement: Supplementary file 1 [file sensors-26-04344-s001.zip › data/images/test/tile_00849_lon-153.0_lat36.0.png]

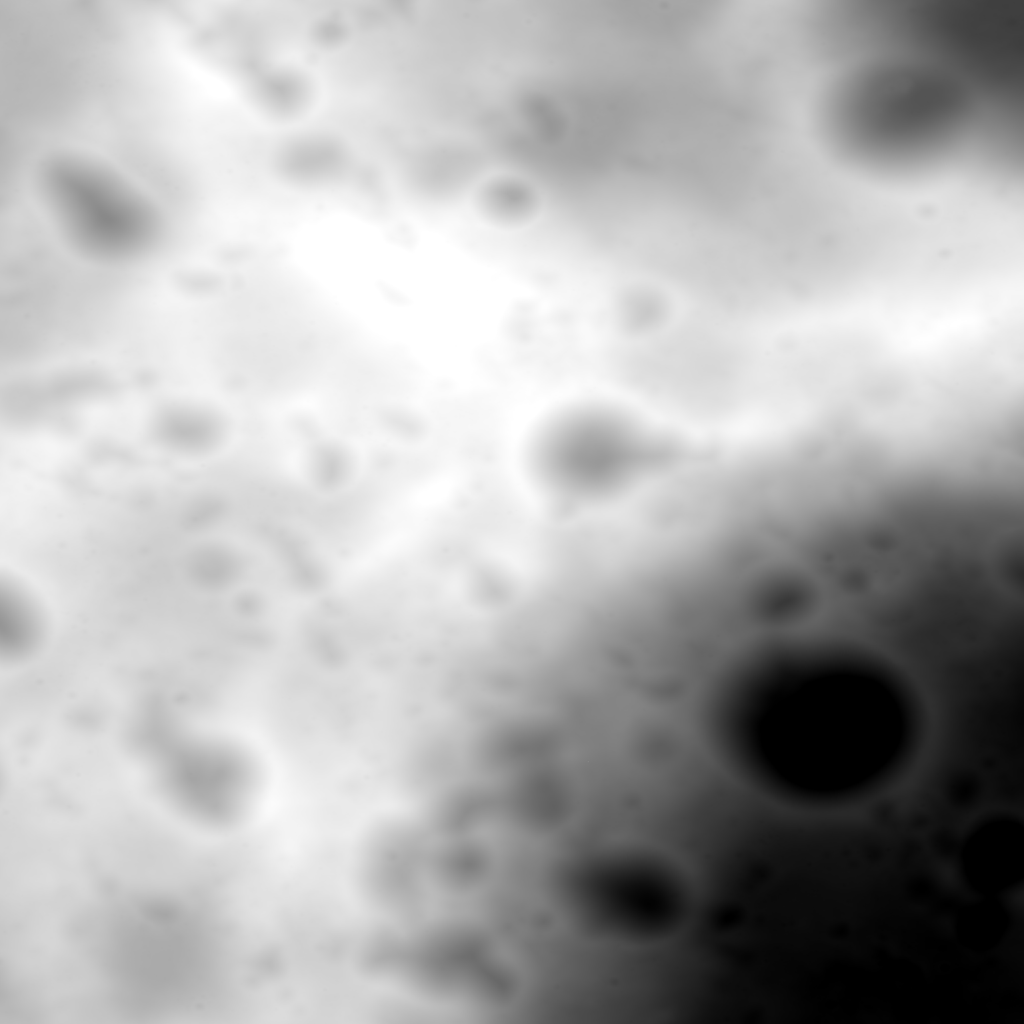

Supplement: Supplementary file 1 [file sensors-26-04344-s001.zip › data/images/test/tile_00855_lon-135.0_lat36.0.png]

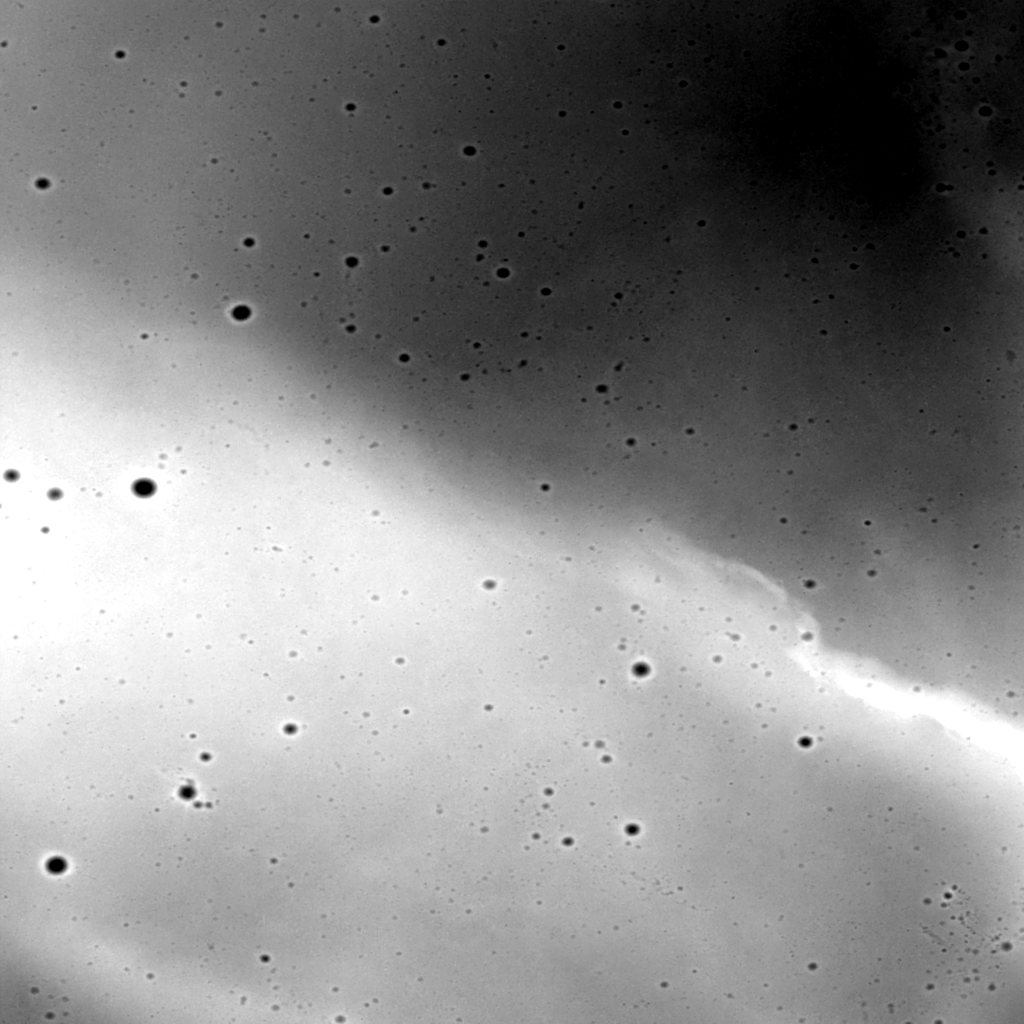

Supplement: Supplementary file 1 [file sensors-26-04344-s001.zip › data/images/test/tile_00878_lon-66.0_lat36.0.png]

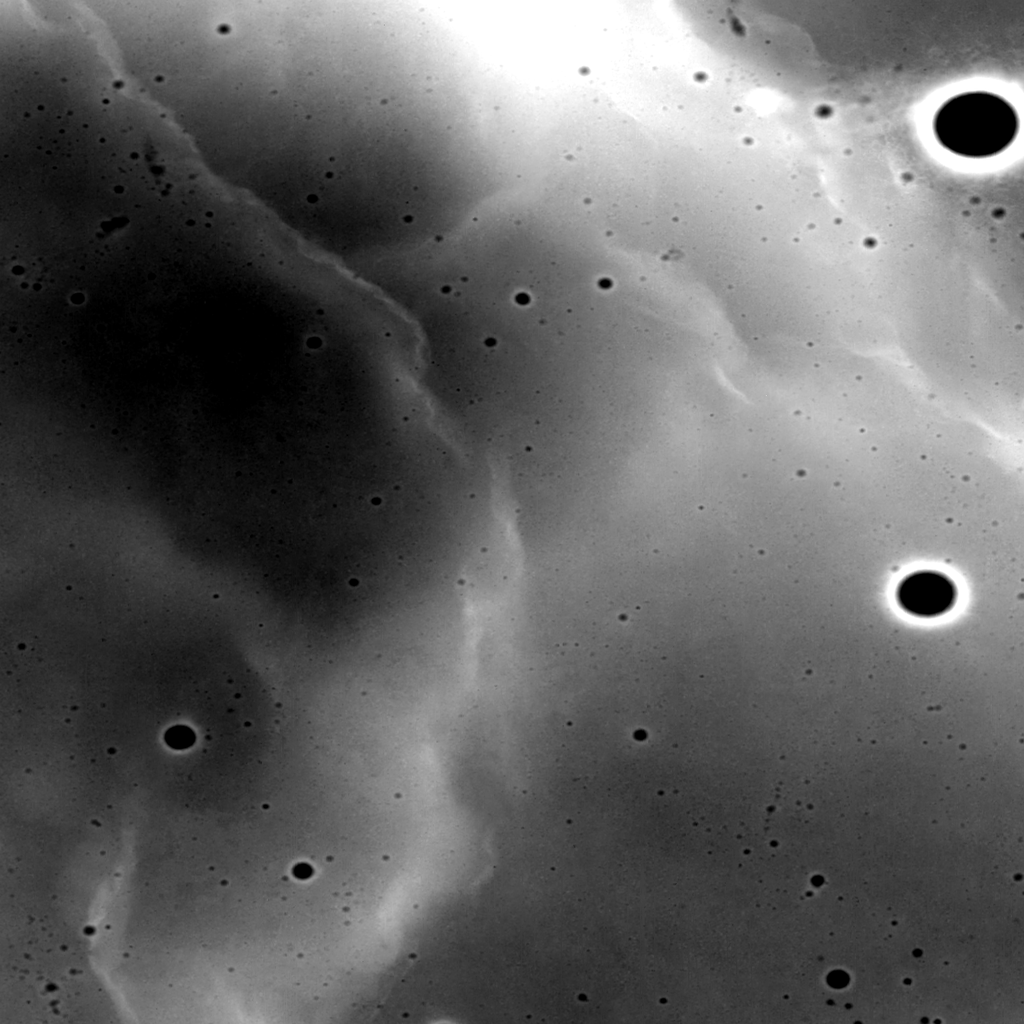

Supplement: Supplementary file 1 [file sensors-26-04344-s001.zip › data/images/test/tile_00880_lon-60.0_lat36.0.png]

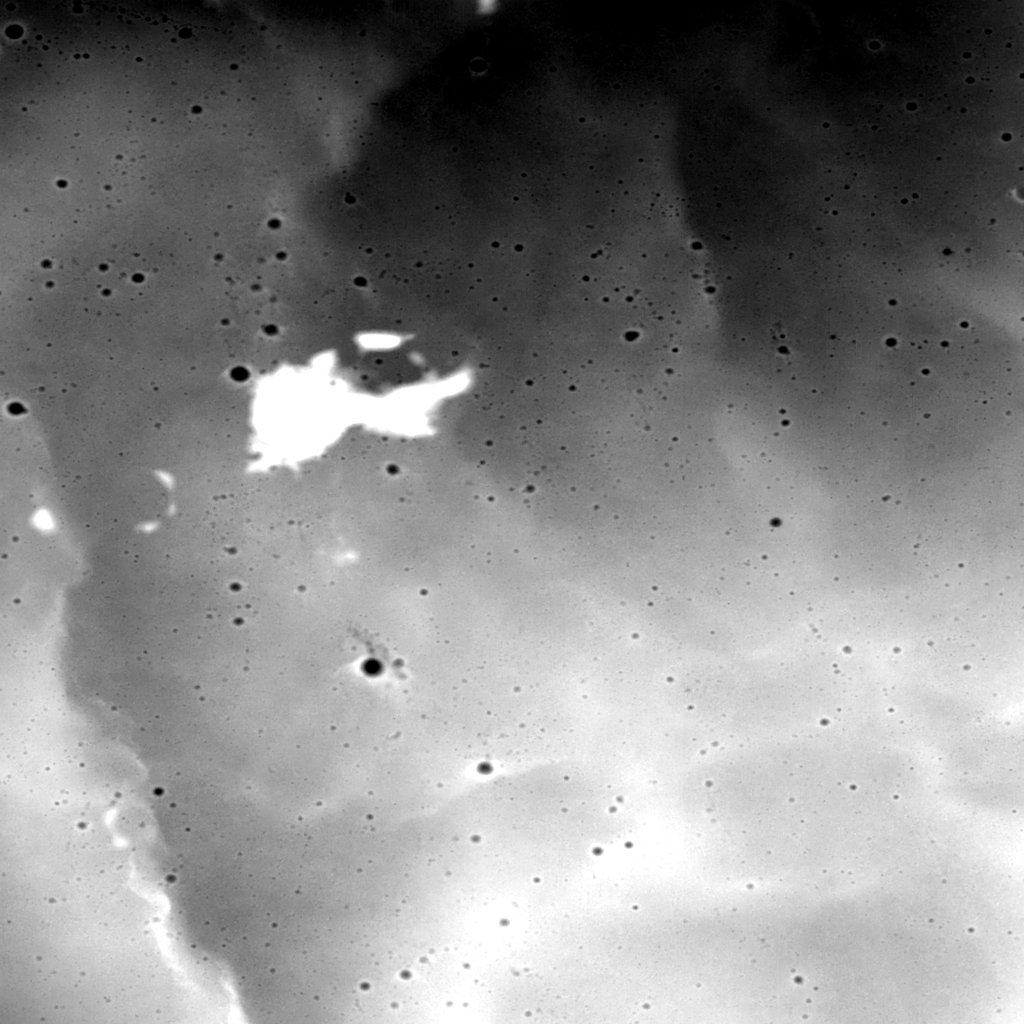

Supplement: Supplementary file 1 [file sensors-26-04344-s001.zip › data/images/test/tile_00883_lon-51.0_lat36.0.png]

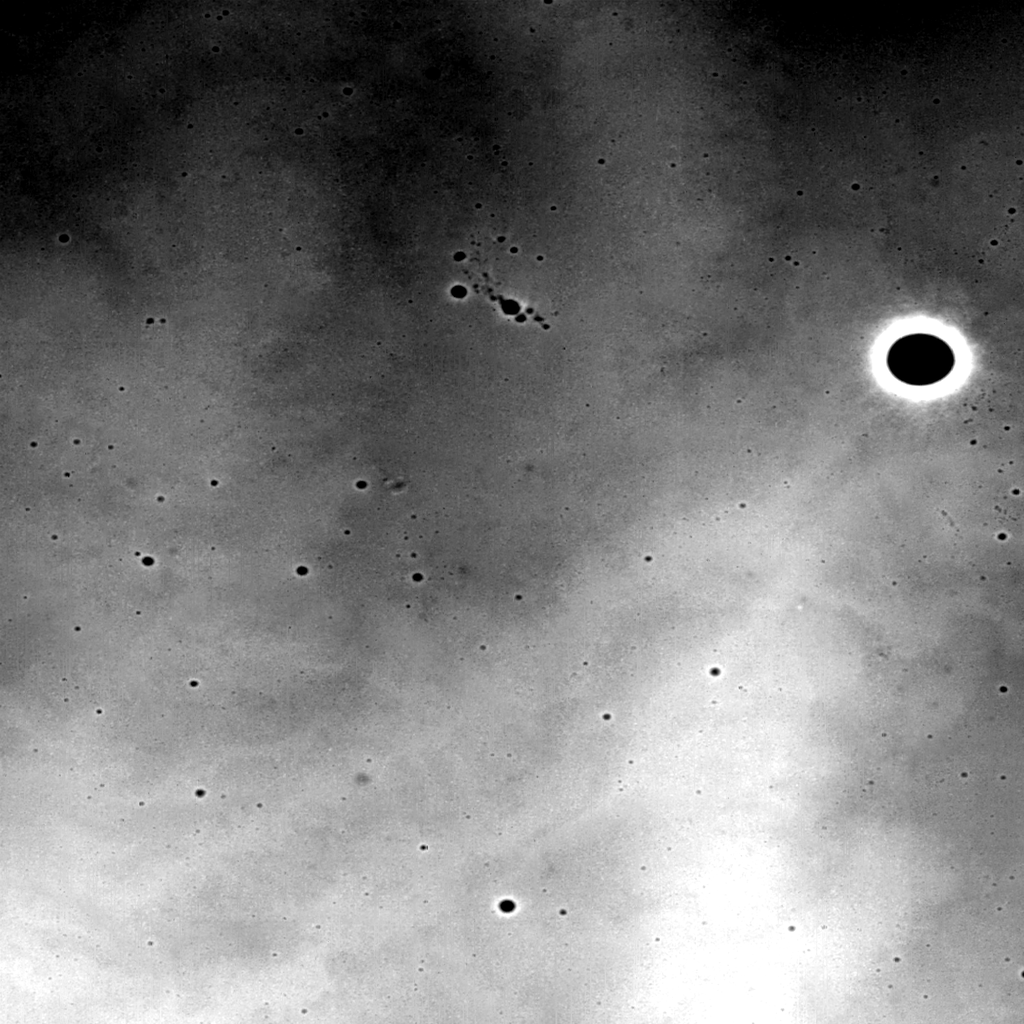

Supplement: Supplementary file 1 [file sensors-26-04344-s001.zip › data/images/test/tile_00892_lon-24.0_lat36.0.png]

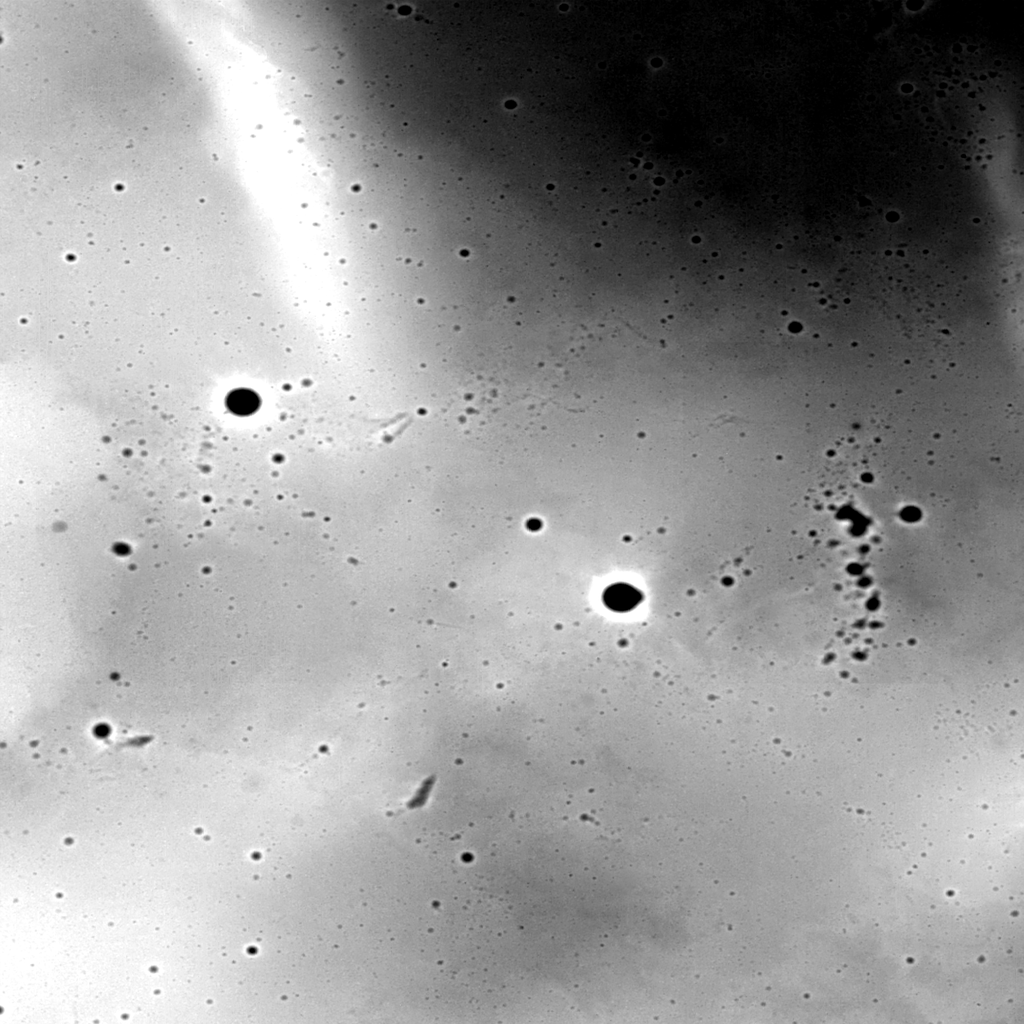

Supplement: Supplementary file 1 [file sensors-26-04344-s001.zip › data/images/test/tile_00895_lon-15.0_lat36.0.png]

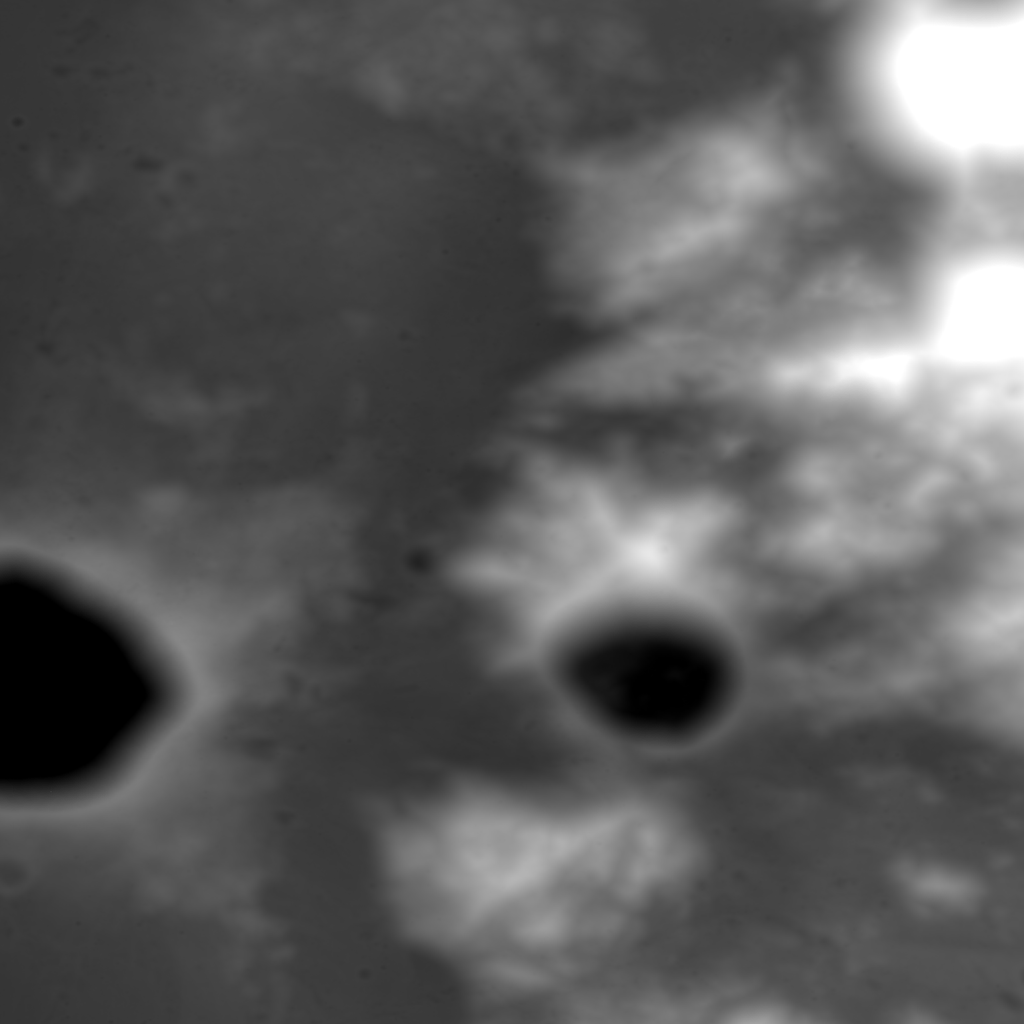

Supplement: Supplementary file 1 [file sensors-26-04344-s001.zip › data/images/test/tile_00902_lon6.0_lat36.0.png]
